# Optimization of University Counseling Consent Forms With Large Language Models: Multidimensional Comparative Evaluation

**DOI:** 10.2196/86502

**Published:** 2026-04-01

**Authors:** Jianchen Luo, Jing Ma, Danni Zhan, Yuhong Zhou, Jiayu Li, Lan Zhang, Wentao Wang

**Affiliations:** 1 Department of Liver Surgery, West China Hospital of Sichuan University, 37 Guoxue Alley, Wuhou District, Chengdu, 610041, China, 86 18980601895; 2 West China School of Clinical Medicine, Sichuan University, Chengdu, China; 3 Mental Health Center, West China Hospital of Sichuan University, Chengdu, China; 4 Department of Vocational Education, The Open University of Mianyang, Mianyang, China; 5 Psychological Research and Counseling Center, Southwest Jiaotong University, Chengdu, China; 6 Department of Psychology, Hebei Normal University, Shijiazhuang, China

**Keywords:** large language models, informed consent forms, university counseling, mental health accessibility, higher education

## Abstract

**Background:**

Mental health problems among university students are a growing global concern, yet limited counseling resources and inadequate understanding of counseling procedures often delay timely help-seeking. Informed consent forms (ICFs) are essential for safeguarding autonomy and clarifying counseling procedures, but many universities’ counseling ICFs are incomplete, ambiguous, or overly technical. Large language models (LLMs) may offer scalable assistance for improving clarity and accessibility.

**Objective:**

This study aimed to evaluate whether LLM-based rewriting could improve the structure, readability, content quality, and comprehensibility of university counseling ICFs, and compared 2 advanced models (ChatGPT [GPT-5] and Grok-4).

**Methods:**

We conducted a comparative evaluation of counseling ICFs collected from 33 Chinese universities (original texts) and generated 2 rewritten versions for each ICF using ChatGPT (GPT-5) and Grok-4. A multidimensional framework assessed (1) textual structure and readability, (2) expert-rated content quality from a counselor perspective, and (3) volunteer-rated reading comprehension from a client perspective. Comparisons between original and rewritten texts were performed using Wilcoxon signed rank tests, with linear mixed-effects models used to validate results while accounting for rater variability.

**Results:**

Compared with the originals, both LLM-rewritten ICFs showed significant improvements across all evaluated dimensions. The mean Lee-Yang Readability Index decreased from 28.68 (SD 5.69) to 22.39 (SD 2.13) with ChatGPT (GPT-5) and 24.37 (SD 2.32) with Grok-4 (both *P*<.001), and mean tone friendliness increased from 2.57 (SD 0.29) to 2.67 (SD 0.12) and 2.67 (SD 0.13), respectively. The mean expert-rated content quality improved from 45.33 (SD 8.74) to 52.54 (SD 7.92) and 55.49 (SD 7.81) (*P*<.001), driven mainly by higher completeness and specificity of key information. The mean volunteer-rated reading comprehension scores increased from 19.02 (SD 1.32) to 22.33 (SD 0.81) and 22.05 (SD 0.90) (*P*<.001), indicating improved clarity, readability, and acceptability. Across structural features, Grok-4 tended to produce longer rewritten forms than the originals, highlighting a potential trade-off between added informational content and document length.

**Conclusions:**

In this comparative evaluation of 33 Chinese university counseling ICFs, LLM-based rewriting was associated with improved readability, expert-rated content quality, and volunteer-rated comprehension relative to original forms. These findings suggest that LLMs can support the optimization of counseling documentation; however, implementation should consider practical constraints (eg, document length) and retain human oversight.

## Introduction

Mental health problems among adolescents and young adults represent a growing global public health concern, particularly in the context of higher education [1]. Estimates suggest that up to 50% of college freshmen worldwide experience at least 1 mental health problem during their time at university [2]. With the rising prevalence of mental health challenges in this population, psychological counseling services have become an indispensable component of campus mental health systems [3]. As the initial step in delivering such services, informed consent forms (ICFs) not only embody fundamental ethical and legal principles but also serve as a critical safeguard for students’ right to information and autonomy. Nevertheless, substantial variability exists in the structure, content, and language of ICFs across universities [4]: some are excessively lengthy and filled with technical jargon, making them difficult for students to understand, while others are incomplete or ambiguously worded, undermining their protective function [5,6].

The clarity and standardization of ICFs are directly linked to recipients’ levels of understanding and the quality of their decision-making [5]. Although relevant efforts have been made in the medical and mental health fields, systematic research specifically targeting ICFs for university counseling services remains scarce [7]. At the institutional level in higher education, many universities lack the capacity to continuously refine these forms due to constraints in manpower and resources, as well as the absence of unified standards and practical technical approaches [8,9].

In recent years, large language models (LLMs) have demonstrated notable strengths in text generation and rewriting [10-13]. Their natural language processing capabilities not only enhance readability and structural clarity but also offer universities lacking professional support a low-cost and scalable auxiliary approach to gradually establish more standardized consent form systems [14-16]. However, empirical evidence remains limited regarding whether LLMs may inadvertently omit critical information during rewriting [17], how to balance professionalism with comprehensibility [18], and whether they can genuinely contribute to the standardization of such documents [19].

To address these gaps, we collected counseling consent forms from 33 universities in China and applied 2 state-of-the-art LLMs, ChatGPT (GPT-5) and Grok-4, to optimize and standardize the texts. The evaluation was conducted across three dimensions: textual structure and readability, content quality, and reading comprehension. By comparing original and LLM-optimized versions, this study aims to examine the feasibility and limitations of using LLMs to improve counseling consent forms and to provide insights for strengthening youth mental health services and exploring human-artificial intelligence (AI) collaboration in clinical communication.

## Methods

### Study Materials and Sources

This study collected 33 psychological counseling ICFs, including 32 from the official websites of Chinese universities and 1 template issued by the Clinical Psychology Registration Work Committee of the Chinese Psychological Society [20]. The template, initially used for pilot testing of LLM rewriting and prompt design, was later included in the final analysis. The sampled universities covered China’s eastern, central, western, and northeastern regions, ensuring geographic representativeness, and encompassed national key, provincial key, and local undergraduate institutions, reflecting variation across higher education tiers. To maintain objectivity and validity, all original documents were analyzed without modification, other than the removal of university identifiers. A full list of universities is provided in Table S1 (see Multimedia Appendix 1).

### Model Selection and Text Generation

Two representative LLMs were examined: ChatGPT (GPT-5) (OpenAI; released August 7, 2025) and Grok-4 (xAI; released July 9, 2025) [21,22]. Both are recently released, widely adopted, and high-performing closed-source systems with strong capabilities in Chinese language processing and text generation [23,24]. All model outputs were generated using the publicly available versions of these models accessible during a defined time window (August 21-September 8, 2025), and the reported results reflect model behavior at the time of data generation. Prior to formal experimentation, we developed and tested a structured prompt defining requirements for contextual framing, preservation and supplementation of key information, linguistic style, and textual organization (Supplementary Information S1 in Multimedia Appendix 2). The structured prompts were natively drafted in Chinese to align with the language of the source documents, and no cross-linguistic translation was involved. The template ICF was used for preliminary trials to verify that the models generated content that was both complete and structurally consistent. Following this procedure, each original text was optimized independently by ChatGPT (GPT-5) and Grok-4, yielding three versions—Original, ChatGPT (GPT-5), and Grok-4—for subsequent comparison and evaluation (all documents are provided in Multimedia Appendix 3). When generating optimized ICFs, both ChatGPT (GPT-5) and Grok-4 were prompted using the same instruction template to ensure fair comparison. The prompts required adherence to both international ethical guidelines and domestic regulations relevant to counseling practice, with Chinese laws prioritized in cases of discrepancy. Retrieval-augmented generation or external document retrieval was not used; all outputs were generated through the models’ standard web-based interfaces.

### Textual Structure and Readability Analysis

To evaluate differences among the 3 ICF versions in structural complexity and linguistic expression, 8 metrics were applied. Five basic structural features were measured: character count, word count, sentence count, words per sentence, and characters per word [4,6,25,26]. Syntactic complexity was captured by the nested sentence ratio [27], reading difficulty by the Lee-Yang Readability Index [28], and tone friendliness was added to reflect approachability and acceptability [29]. All features were derived through a standardized processing pipeline.

The Lee-Yang Readability Index is a weighted composite of average sentence length, word length, and lexical complexity, widely used to assess the readability of Chinese texts [30], especially in science communication, education, and health contexts [28,31]. Tone friendliness was operationalized using a lexical counting approach. Two predefined dictionaries—politeness- or empathy-related words and harsh words—were applied (Supplementary Information S2 in Multimedia Appendix 2) [32]. For each text, the normalized frequency difference between the 2 word categories (adjusted by sentence count) was linearly mapped from −1 to 1 onto a 0 to 5 scale, with values truncated if outside this range. This metric was intended as a surface-level lexical proxy of linguistic tone rather than a contextual or pragmatic assessment of emotional friendliness. Higher scores indicate a greater relative use of polite or empathic wording and fewer harsh or legalistic terms. The method is interpretable, reproducible, and adaptable through dictionary refinement [33].

### Content Quality Assessment

To assess the completeness and standardization of ICFs, we drew on major international and national ethical guidelines. These included the American Psychological Association Ethical Principles and Code of Conduct (2016) [34], the British Psychological Society Code of Ethics and Conduct (2018) [35], the Code of Ethics for Clinical and Counseling Psychology (2018) [36], and the World Health Organization Comprehensive Mental Health Action Plan 2013‐2030 (2021) [37]. Together, these documents provided the ethical and methodological foundation for the content quality evaluation framework.

Building on this foundation, we established an evaluation framework of 20 core indicators widely considered essential for ICFs: confidentiality; exceptions to confidentiality; client rights; guardian consent; goals and scope; format and frequency; fees and cancelation policy; recording methods; authorization and revocation; crisis procedures; complaints and appeals; data protection; disclaimer of boundaries; language clarity; voluntariness; client obligations; counseling limitations; counselor qualifications; counseling modalities; target population (detailed descriptions and rationales in Multimedia Appendix 1, Table S2). Each indicator was assessed across three dimensions: existence (0‐1), defined as whether the element was explicitly presented as an independent item (eg, a distinct heading or clearly delineated paragraph); specificity (0‐2), reflecting whether concrete details were provided; and operability (0‐2), indicating whether actionable procedures were specified (detailed in Multimedia Appendix 1**,** Table S3). When a relevant indicator was mentioned within other sections without being explicitly listed as an independent item, existence was scored as 0, while specificity and operability were still assessed based on the content provided. Each indicator had a maximum of 5 points, for a total of 100 across all 20 indicators, with subscores of 20 for existence, 40 for specificity, and 40 for operability.

Prior to formal application, 3 senior experts in psychology and ethics reviewed and revised the proposed framework to ensure scientific rigor, comprehensiveness, and operability. A pilot test using the template ICF was then conducted to examine applicability and discriminative capacity. Once feasibility was confirmed, the finalized framework was applied to the remaining 32 ICFs. Scoring was performed independently by 5 experts with backgrounds in psychology or mental health. To maximize fairness and consistency, all texts were randomized and anonymized so that raters were blinded to both the source and version.

### Reading Comprehension Assessment

To assess differences in comprehensibility among ICF versions, 10 university volunteers from nonpsychology and nonhealth-related majors participated as readers simulating clients; none had received formal training in counseling or mental health disciplines. They evaluated the texts using a 6D framework: comprehensibility—ease of reading and understanding [8,38]; clarity—whether key information was clearly and unambiguously expressed [39,40]; trustworthiness—perceived reliability of content [41,42]; friendliness—supportive, respectful, and approachable tone [32]; professionalism—appropriate formality and expertise [43]; and acceptability—whether content and style were acceptable to clients [44]. Each dimension was rated on a 5-point Likert scale (1=strongly disagree, 5=strongly agree) [45,46]. The maximum per-dimension score was 5, yielding a total score of 6‐30. A seventh composite dimension (“dimension means”), defined as the average of the six dimensions (range 1–5), was calculated. Prior to the formal study, a pilot test with the template ICF was conducted to examine the scale’s applicability and discriminative capacity. During formal rating, all texts were randomized and anonymized to blind raters to both source and version, ensuring objectivity.

### Statistical Analysis

Differences among the 3 ICF versions were examined across textual structure and readability, content quality, and reading comprehension. For textual structure and readability, values for 8 indicators were calculated for each document. For content quality and reading comprehension, ratings from multiple evaluators were averaged per document and then summarized by version. In all cases, means and SDs were reported.

All between-version comparisons were conducted using the Wilcoxon signed rank test. Effect sizes (*r*) were reported, and *P*-values were adjusted for multiple testing using the false discovery rate approach (Benjamini-Hochberg procedure). Interrater reliability for total scores of content quality and reading comprehension was assessed using intraclass correlation coefficients (ICCs), based on ratings from 5 experts and 10 readers, respectively.

In addition, to account for individual differences among raters and documents, linear mixed-effects models (LMMs) were fitted with “version” as a fixed effect and “document ID” and “rater” as random effects, allowing robust estimation of version effects under a repeated-measures design. Because content quality and reading comprehension were evaluated by different groups, separate models were constructed for each outcome. Estimation used the MixedLM module. All tests were 2-tailed with significance set at *P*<.05. *P* values from the LMMs were not adjusted for multiple comparisons; therefore, the possibility of type I error due to multiple testing cannot be excluded. Analyses were performed in Python 3.11 using pandas (v2.2.2; pandas development team), NumPy (v1.26.4; NumPy developers), SciPy (v1.11.4; SciPy developers), jieba (v0.42.1; Jieba development team), statsmodels (v0.14.1; statsmodels developers), scikit-learn (v1.4.0; scikit-learn developers), and Pingouin (v0.5.4; Pingouin developers). All codes are available in Multimedia Appendix 4 for reproducibility.

### Ethical Considerations

This study involved human participants and was reviewed and approved by the Ethics Committee of West China Hospital, Sichuan University (Approval 2024‐1431). The study was classified as minimal risk and conducted in accordance with the Declaration of Helsinki. Written informed consent was obtained from all participants prior to participation, including expert raters and student volunteers who evaluated deidentified university counseling ICFs; no clinical intervention or therapeutic interaction was involved. To protect privacy and confidentiality, all documents were anonymized before analysis, with institutional identifiers removed, and no personally identifiable information was collected or analyzed. Participants received no financial or material compensation, and the manuscript and multimedia appendices do not contain any images or content that could enable identification of individual participants.

## Results

### Textual Structure and Readability

Compared with the original texts, ChatGPT (GPT-5)–optimized ICFs showed more sentences (mean 51.30, SD 12.27 vs mean 36.79, SD 13.29; *P*<.001), fewer words per sentence (mean 20.23, SD 2.29 vs mean 28.05, SD 6.45; *P*<.001), more characters per word (mean 1.16, SD 0.04 vs mean 1.12, SD 0.03; *P*<.001), and a lower nested sentence ratio (mean 0.21, SD 0.06 vs mean 0.32, SD 0.14; *P*<.001). Character count (mean 1206.42, SD 353.00 vs mean 1117.61, SD 376.58; *P*=.17) and word count (mean 1039.33, SD 297.52 vs mean 996.24, SD 338.14; *P*=.44) did not differ significantly. The Lee-Yang Readability Index was lower (mean 22.39, SD 2.13 vs mean 28.68, SD 5.69; *P*<.001), while tone friendliness was higher (mean 2.67, SD 0.12 vs mean 2.57, SD 0.29; *P*=.04).

Grok-4–optimized ICFs were significantly longer, with higher character counts (mean 1669.52, SD 234.09; *P*<.001) and word counts (mean 1393.21, SD 208.82; *P*<.001). Sentence count also increased (mean 65.33, SD 10.15; *P*<.001), words per sentence decreased (mean 21.49, SD 2.56; *P*<.001), characters per word increased (mean 1.20, SD 0.04; *P*<.001), and the nested sentence ratio decreased (mean 0.23, SD 0.06; *P*<.001). The Lee-Yang Index also decreased (mean 24.37, SD 2.32; *P*<.001), and tone friendliness improved (mean 2.67, SD 0.13; *P*=.02).

**Figure 1.**
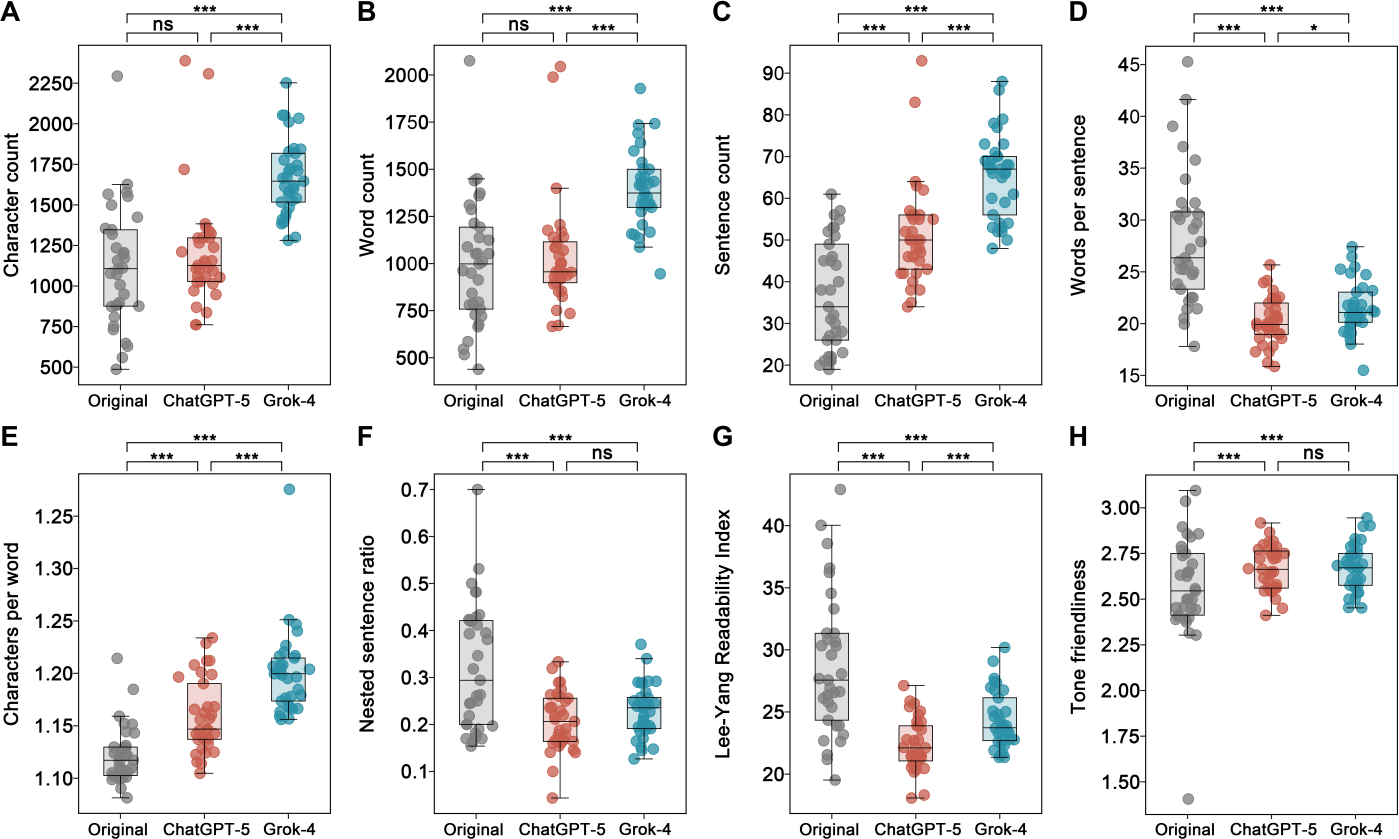
Text structure and readability indicators across original and large language model–optimized university counseling informed consent forms. Box plots compare 8 structural and readability indicators across original and ChatGPT-5– and Grok-4–rewritten counseling informed consent forms (n=33). Dots represent individual documents. Statistical comparisons were performed using Wilcoxon signed rank tests. Significance thresholds: *P*<.05 (*), *P*<.01 (**), *P*<.001 (***), not significant (ns). (A) Character count, (B) word count, (C) sentence count, (D) words per sentence, (E) characters per word, (F) nested sentence ratio, (G) Lee-Yang Readability Index, and (H) tone friendliness.

Direct comparisons showed that Grok-4 had higher character counts, word counts, sentence counts, and characters per word (*P*<.001). Differences in words per sentence were modest (*P*=.02), nested sentence ratios did not differ (*P*=.09), and Grok-4 had a higher Lee-Yang Readability Index (*P*<.001). Tone friendliness was comparable (*P*=.76). Full results are shown in Table 1 and Figure 1. Detailed statistical analysis results are provided in Table S4 (Multimedia Appendix 1).

**Table 1.** Comparative analysis of multidimensional evaluation results across all versions.

Indicator	Original, mean (SD)	ChatGPT-5, mean (SD)	Grok-4, mean (SD)	ChatGPT-5 versus original (*P* value)^a^	Grok-4 versus original (*P* value)	Grok-4 versus ChatGPT-5 (*P* value)
Text structure						
Character count	1117.61 (376.58)	1206.42 (353.00)	1669.52 (234.09)	.17	<.001	<.001
Word count	996.24 (338.14)	1039.33 (297.52)	1393.21 (208.82)	.44	<.001	<.001
Sentence count	36.79 (13.29)	51.30 (12.27)	65.33 (10.15)	<.001	<.001	<.001
Words per sentence	28.05 (6.45)	20.23 (2.29)	21.49 (2.56)	<.001	<.001	.02
Characters per word	1.12 (0.03)	1.16 (0.04)	1.20 (0.04)	<.001	<.001	<.001
Nested sentence ratio	0.32 (0.14)	0.21 (0.06)	0.23 (0.06)	<.001	<.001	.09
Readability						
Lee-Yang Readability Index	28.68 (5.69)	22.39 (2.13)	24.37 (2.32)	<.001	<.001	<.001
Tone friendliness	2.57 (0.29)	2.67 (0.12)	2.67 (0.13)	.04	.02	.76
Content quality						
Existence	11.03 (2.18)	12.72 (1.26)	13.55 (1.24)	<.001	<.001	.004
Specificity	11.55 (3.08)	16.51 (3.38)	18.25 (2.38)	<.001	<.001	.02
Operability	22.75 (5.73)	23.31 (6.12)	23.69 (6.46)	.31	.10	.60
Total	45.33 (8.74)	52.54 (7.92)	55.49 (7.81)	<.001	<.001	.01
Reading comprehension						
Comprehensibility	2.93 (0.45)	3.76 (0.30)	3.69 (0.35)	<.001	<.001	.19
Clarity	2.98 (0.47)	3.78 (0.28)	3.74 (0.28)	<.001	<.001	.32
Trustworthiness	3.30 (0.30)	3.72 (0.26)	3.61 (0.32)	<.001	<.001	.12
Friendliness	3.35 (0.35)	3.75 (0.32)	3.73 (0.27)	<.001	<.001	.79
Professionalism	3.25 (0.25)	3.55 (0.32)	3.64 (0.30)	<.001	<.001	.21
Acceptability	3.20 (0.40)	3.77 (0.37)	3.65 (0.33)	<.001	<.001	.06
Total^b^	19.02 (1.32)	22.33 (0.81)	22.05 (0.90)	<.001	<.001	.11
Dimension means (total^c^/6)	3.17 (0.22)	3.72 (0.13)	3.68 (0.15)	<.001	<.001	.11

a *P* values were adjusted for multiple comparisons using the false discovery rate.

b Total score of the 3 dimensions in content quality evaluation.

c Total score of the 6 dimensions in reading comprehension evaluation.

### Content Quality

Interrater reliability among 5 expert raters across all documents was high (ICC [2,k]=0.83; Table S5 in Multimedia Appendix 1). Compared with the original texts, ChatGPT (GPT-5)–optimized ICFs showed higher existence (mean 12.72, SD 1.26 vs mean 11.03, SD 2.18; *P*<.001) and specificity scores (mean 16.51, SD 3.38 vs mean 11.55, SD 3.08; *P*<.001), while operability did not differ (mean 23.31, SD 6.12 vs mean 22.75, SD 5.73; *P*=.31). The total score increased significantly (mean 52.54, SD 7.92 vs mean 45.33, SD 8.74; *P*<.001). Grok-4 also showed marked gains, with higher existence (mean 13.55, SD 1.24; *P*<.001) and specificity (mean 18.25, SD 2.38; *P*<.001). Operability increased (mean 23.69, SD 6.46) but was not significant (*P*=.09). The total score rose to a mean of 55.49 (SD 7.81; *P*<.001). Direct comparisons showed Grok-4 scored higher than ChatGPT (GPT-5) in existence (*P*=.004) and specificity (*P*=.02), with no difference in operability (*P*=.60). The overall score was also higher for Grok-4 (*P*=.01) (Table 1). Detailed statistical analysis results are provided in Multimedia Appendix 1, Table S6.

**Figure 2.**
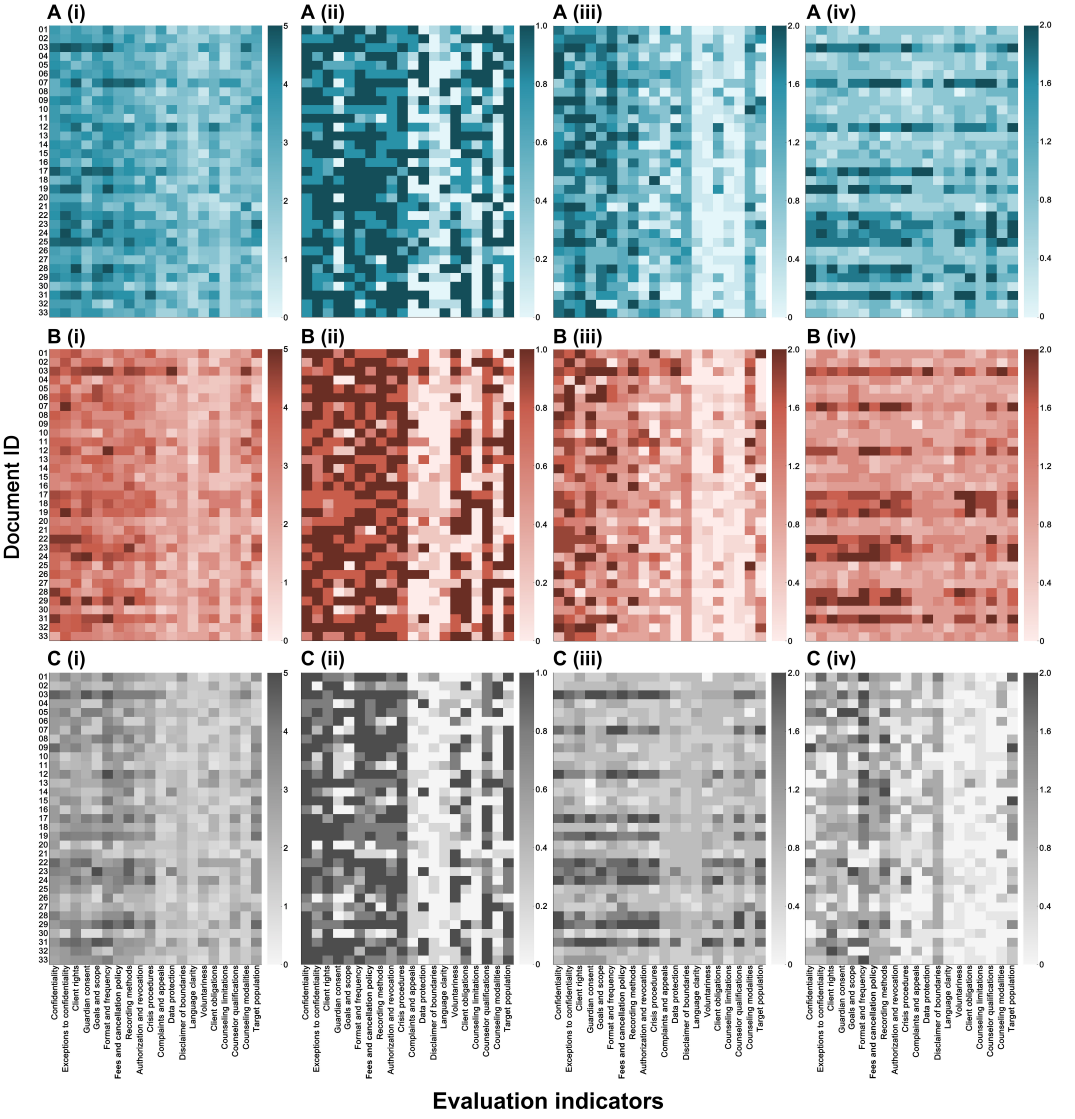
Expert-rated content quality profiles of original and large language models–optimized university counseling informed consent forms. Heatmaps display document-level content quality scores for 33 counseling informed consent forms, evaluated across 20 predefined indicators grouped into three dimensions (existence, specificity, and operability), as well as the total score. Darker colors indicate higher scores. (A) Grok-4–rewritten versions, (B) ChatGPT (GPT-5)–rewritten versions, and (C) original documents. Dimension-level summaries include (i) total score, (ii) existence score, (iii) specificity score, and (iv) operability score.

To illustrate performance across the 20 core indicators, a heatmap was generated (Figure 2). The analyses showed that several indicators—confidentiality, exceptions to confidentiality, goals and scope, authorization and revocation, complaints and appeals, disclaimer of boundaries, and counseling modalities—improved significantly under both LLM-optimized versions. These gains were mainly in specificity, with few improvements in existence or operability. Between the 2 LLMs, significant differences appeared only in client rights and language clarity; all other indicators showed no differences. Full statistical results are provided in Tables S7-S11, and rater-level scores in Supplementary Data S1-S5 (Multimedia Appendix 5).

### Reading Comprehension

Interrater reliability among 10 readers across all documents was fair (ICC [2,k]=0.59; Table S12 in Multimedia Appendix 1). LLM-optimized ICFs outperformed the originals across all dimensions and in total scores (Table 1). For ChatGPT (GPT-5), scores improved in comprehensibility (mean 3.76, SD 0.30 vs mean 2.93, SD 0.45; *P*<.001), clarity (mean 3.78, SD 0.28 vs mean 2.98, SD 0.47; *P*<.001), trustworthiness (mean 3.72, SD 0.26 vs mean 3.30, SD 0.30; *P*<.001), friendliness (mean 3.75, SD 0.32 vs mean 3.35, SD 0.35; *P*<.001), professionalism (mean 3.55, SD 0.32 vs mean 3.25, SD 0.25; *P*<.001), and acceptability (mean 3.77, SD 0.37 vs mean 3.20, SD 0.40; *P*<.001). The total score also rose (mean 22.33, SD 0.81 vs mean 19.02, SD 1.32; *P*<.001).

Grok-4 showed similar mean gains: comprehensibility 3.69 (SD 0.35; *P*<.001), clarity 3.74 (SD 0.28; *P*<.001), trustworthiness 3.61 (SD 0.32; *P*<.001), friendliness 3.73 (SD 0.27; *P*<.001), professionalism 3.64 (SD 0.30; *P*<.001), and acceptability 3.65 (SD 0.33; *P*<.001), with a total of 22.05 (SD 0.90; *P*<.001).

**Figure 3.**
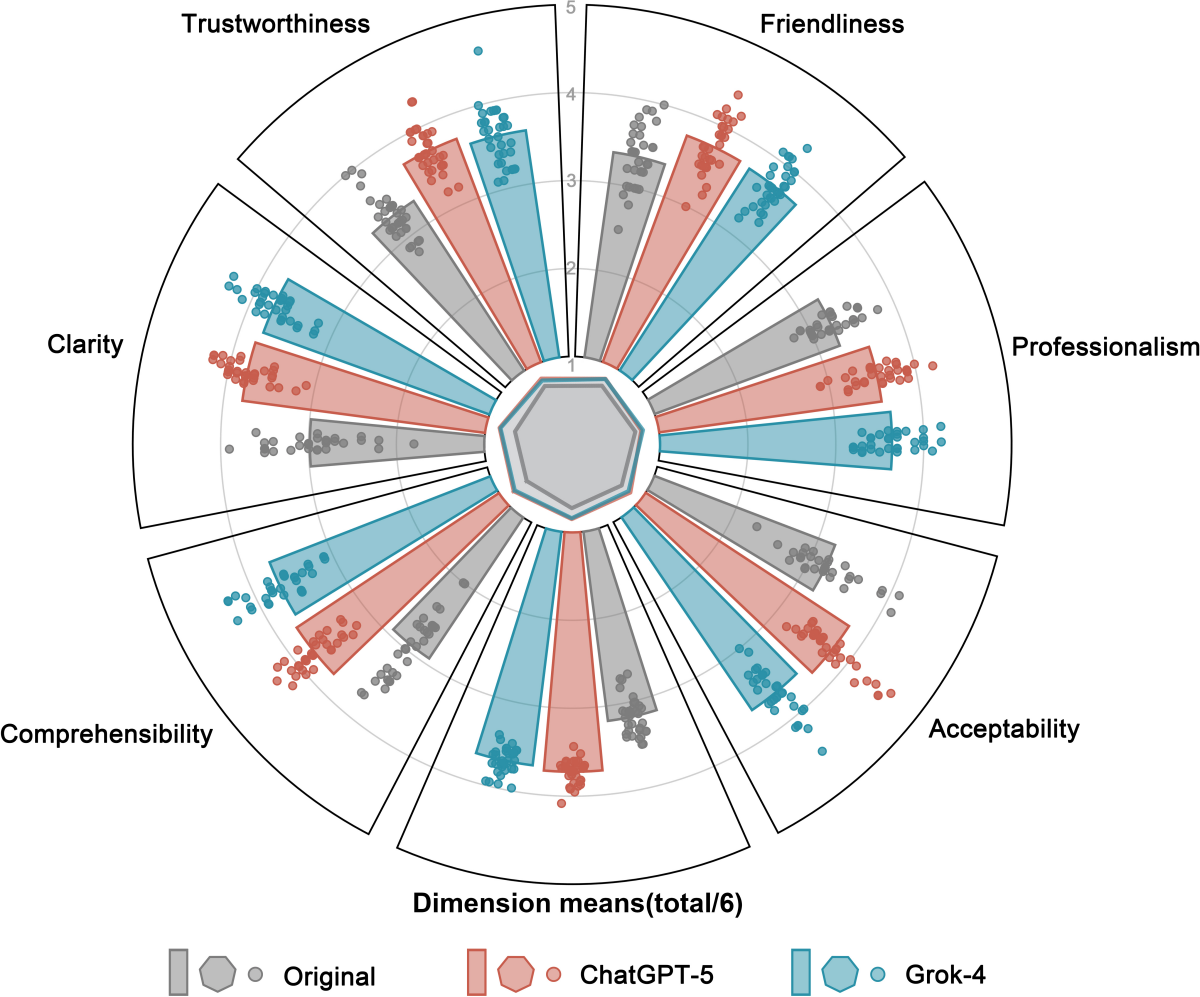
Volunteer-rated reading comprehension of original and large language model–optimized university counseling informed consent forms.

In direct comparison, ChatGPT (GPT-5) had higher means across most dimensions and the total score, but none reached significance (comprehensibility: *P*=.19; clarity: *P*=.32; trustworthiness: *P*=.12; friendliness: *P*=.79; professionalism: *P*=.21; and acceptability: *P*=.06). For visualization, dimension means were plotted instead of totals (Figure 3). Detailed statistical analysis results are provided in Table S13 in Multimedia Appendix 1. Complete dimension-level ratings are in Supplementary Data S6-S15 in Multimedia Appendix 5.

The figure compares reading comprehension scores across original, ChatGPT (GPT-5)–rewritten, and Grok-4–rewritten counseling ICFs (n=33). Mean scores across 6 comprehension dimensions and the total score are shown, with dots indicating document-level distributions. A central radar plot summarizes dimension-level means for comparison.

### Linear Mixed-Effects Model

**Figure 4.**
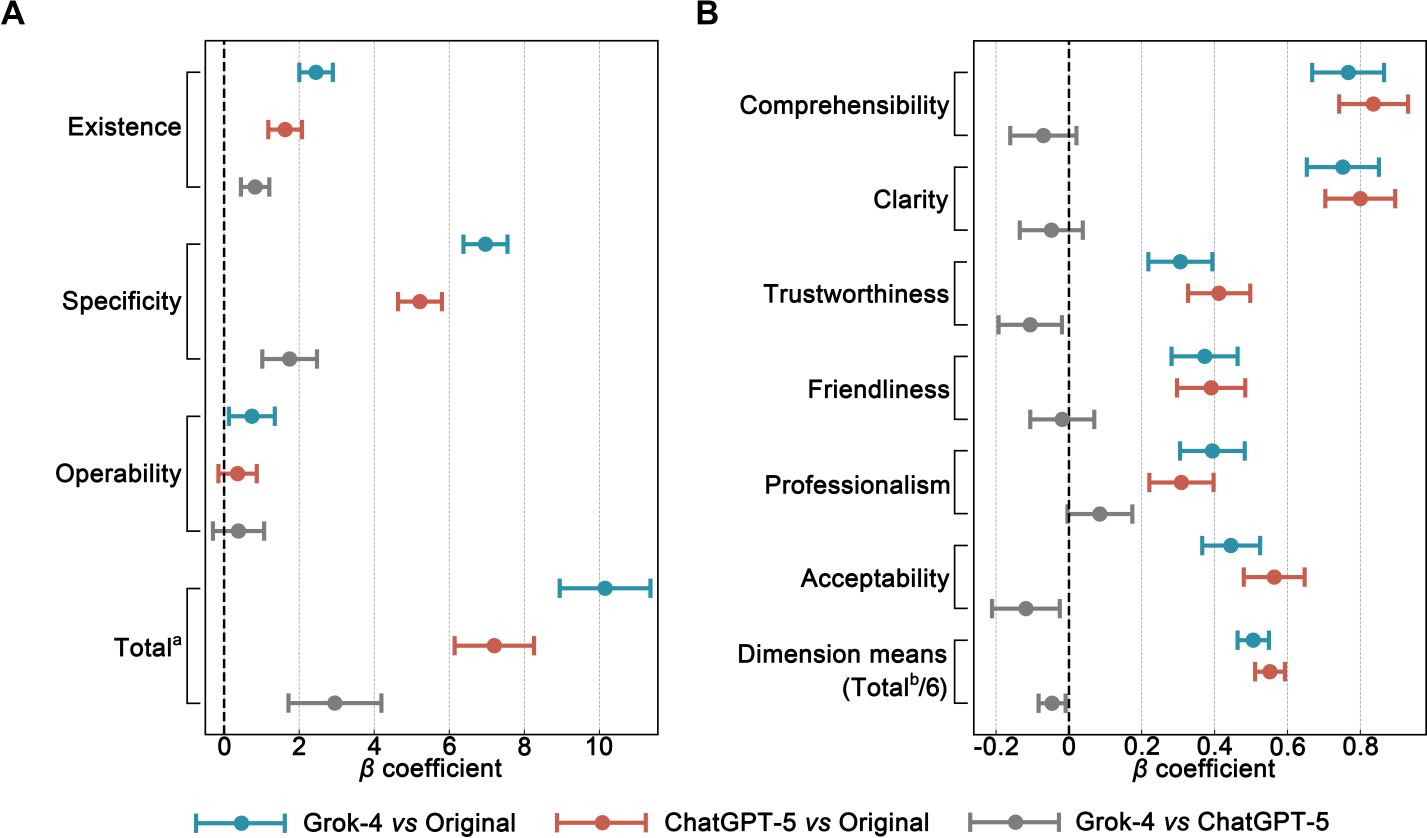
Linear mixed-effects model results for (A) content quality and (B) reading comprehension across original and large language model–optimized university counseling informed consent forms.

To account for individual differences among raters and texts, LMMs with crossed random effects were constructed, specifying “version” as a fixed effect and including random intercepts for rater and document ID. Separate models were fitted for content quality and reading comprehension (Figure 4). In the content quality model, ChatGPT (GPT-5) yielded higher total scores than the original texts (*β*=7.20, *P*<.001), with significant gains in existence (*β*=1.62; *P*<.001) and specificity (*β*=5.22; *P*<.001), but not operability (*β*=.36; *P*=.17). Grok-4 also showed significant improvements over the originals in total scores (*β*=10.15; *P*<.001), existence (*β*=2.45; *P*<.001), specificity (*β*=6.96; *P*<.001), and operability (*β*=.74; *P*=.02). Direct comparison showed Grok-4 scored higher than ChatGPT (GPT-5) in total scores (*β*=2.95; *P*<.001), existence (*β*=0.82; *P*<.001), and specificity (*β*=1.75; *P*<.001), with no difference in operability (*β*=.38; *P*=.27).

The figure shows LMM estimates comparing original, ChatGPT (GPT-5)–rewritten, and Grok-4–rewritten counseling ICFs (n=33). Models were fitted separately for content quality and reading comprehension outcomes. Positive *β* coefficients indicate higher scores for the first group in each comparison. The vertical dashed line at *β*=0 represents no difference between groups. CIs that do not cross the zero line indicate statistically significant differences at *P*<.05.

In the reading comprehension model, ChatGPT (GPT-5) showed significant improvements over the original texts in comprehensibility (*β*=.84; *P*<.001), clarity (*β*=.80; *P*<.001), trustworthiness (*β*=.41; *P*<.001), friendliness (*β*=.39; *P*<.001), professionalism (*β*=.31; *P*<.001), acceptability (*β*=.56; *P*<.001), and dimension means (*β*=.55; *P*<.001). Grok-4 likewise outperformed the originals, with gains in comprehensibility (*β*=.77; *P*<.001), clarity (*β*=.75; *P*<.001), trustworthiness (*β*=.31; *P*<.001), friendliness (*β*=.37; *P*<.001), professionalism (*β*=.39; *P*<.001), acceptability (*β*=.45; *P*<.001), and dimension means (*β*=.51; *P*<.001). In direct comparison, Grok-4 scored lower than ChatGPT (GPT-5) in trustworthiness (*β*=−.11; *P*=.02), acceptability (*β*=−.12; *P*=.01), and dimension means (*β*=−.05; *P*=.01). Details are reported in Table 2, with random effects and model fit indices in Multimedia Appendix 1, Tables S14 and S15.

**Table 2.** Results of linear mixed-effects model analysis on content quality and reading comprehension.

Dimension and comparison	*β* (95% CI)	SE	*P* value^a^
Content quality			
Existence			
Grok-4 vs original	2.45 (2.00 to 2.90)	0.23	<.001
ChatGPT-5 vs original	1.62 (1.18 to 2.07)	0.23	<.001
Grok-4 vs ChatGPT-5	0.82 (0.45 to 1.20)	0.19	<.001
Specificity			
Grok-4 vs original	6.96 (6.38 to 7.55)	0.30	<.001
ChatGPT-5 vs original	5.22 (4.63 to 5.80)	0.30	<.001
Grok-4 vs ChatGPT-5	1.74 (1.02 to 2.47)	0.37	<.001
Operability			
Grok-4 vs original	0.74 (0.13 to 1.35)	0.31	.02
ChatGPT-5 vs original	0.36 (–0.15 to 0.87)	0.26	.17
Grok-4 vs ChatGPT-5	0.38 (–0.30 to 1.06)	0.35	.27
Total^b^			
Grok-4 vs original	10.15 (8.94 to 11.36)	0.62	<.001
ChatGPT-5 vs original	7.20 (6.14 to 8.26)	0.54	<.001
Grok-4 vs ChatGPT-5	2.95 (1.71 to 4.19)	0.63	<.001
Reading comprehension			
Comprehensibility			
Grok-4 vs original	0.77 (0.67 to 0.86)	0.05	<.001
ChatGPT-5 vs original	0.84 (0.74 to 0.93)	0.05	<.001
Grok-4 vs ChatGPT-5	–0.07 (–0.16 to 0.02)	0.05	.13
Clarity			
Grok-4 vs original	0.75 (0.65 to 0.85)	0.05	<.001
ChatGPT-5 vs original	0.80 (0.70 to 0.90)	0.05	<.001
Grok-4 vs ChatGPT-5	–0.05 (–0.14 to 0.04)	0.04	.27
Trustworthiness			
Grok-4 vs original	0.31 (0.22 to 0.39)	0.05	<.001
ChatGPT-5 vs original	0.41 (0.33 to 0.50)	0.04	<.001
Grok-4 vs ChatGPT-5	–0.11 (–0.19 to 0.02)	0.04	.02
Friendliness			
Grok-4 vs original	0.37 (0.28 to 0.46)	0.05	<.001
ChatGPT-5 vs original	0.39 (0.30 to 0.48)	0.05	<.001
Grok-4 vs ChatGPT-5	–0.02 (–0.11 to 0.07)	0.05	.69
Professionalism			
Grok-4 vs original	0.39 (0.30 to 0.48)	0.05	<.001
ChatGPT-5 vs original	0.31 (0.22 to 0.40)	0.05	<.001
Grok-4 vs ChatGPT-5	0.08 (0.00 to 0.17)	0.05	.06
Acceptability			
Grok-4 vs original	0.44 (0.37 to 0.53)	0.04	<.001
ChatGPT-5 vs original	0.56 (0.48 to 0.65)	0.04	<.001
Grok-4 vs ChatGPT-5	–0.12 (–0.21 to 0.03)	0.05	.01
Total^c^			
Grok-4 vs original	3.04 (2.78 to 3.30)	0.13	<.001
ChatGPT-5 vs original	3.31 (3.07 to 3.56)	0.13	<.001
Grok-4 vs ChatGPT-5	–0.28 (–0.50 to 0.06)	0.11	.01
Dimension means (Total^c^/6)			
Grok-4 vs original	0.51 (0.46 to 0.55)	0.02	<.001
ChatGPT-5 vs original	0.55 (0.51 to 0.59)	0.02	<.001
Grok-4 vs ChatGPT-5	–0.05 (–0.08 to 0.01)	0.02	.01

a *P* values from the linear mixed-effects models were not adjusted for multiple comparisons.

b Total score of the 3 dimensions in content quality evaluation.

c Total score of the 6 dimensions in reading comprehension evaluation.

## Discussion

### Principal Results

This study systematically evaluated the performance of LLMs in rewriting university counseling ICFs. It focused on 2 state-of-the-art closed-source models, ChatGPT (GPT-5) and Grok-4. Both achieved significant improvements over the originals in textual structure and readability, content quality, and reading comprehension. LLM-based rewriting reduced linguistic complexity, enhanced the completeness and specificity of key information, and improved clarity and acceptability, making ICFs easier to understand and apply. Such improvements may help lower barriers to accessing counseling, thereby supporting more timely use of mental health services among young people. LMMs confirmed these advantages across multiple dimensions. This study is also the first to design and apply a multidimensional evaluation framework for university counseling ICFs. The framework deliberately distinguished evaluation perspectives: content quality was assessed by psychology or mental health experts from a counselor’s standpoint, while reading comprehension was evaluated by university volunteers simulating clients. This dual-perspective design enabled the assessment of both professional and ethical adequacy and actual acceptability and comprehensibility. Together, the findings underscore the value of LLMs in optimizing counseling ICFs and offer a methodological reference for future research.

### Comparison With Prior Work

In terms of textual structure and readability, LLM-based rewriting substantially altered the linguistic features of ICFs. ChatGPT (GPT-5)–generated texts showed shorter words per sentence and lower nested sentence ratios, indicating improved clarity and comprehensibility through sentence splitting and syntactic simplification [47]. The increase in characters per word likely reflects the use of more standardized, professional vocabulary to ensure accuracy while simplifying syntax [4]. The lower Lee-Yang Readability Index and higher tone friendliness suggest reduced overall difficulty with a more approachable style [28]. It should be noted that tone friendliness, as operationalized here, reflects surface-level lexical tendencies and may not fully capture context-dependent or pragmatically appropriate expressions; for example, legally protective wording may serve a positive ethical function despite being classified as strict. Although statistically significant, the absolute increase was small (approximately 0.1 on a 5-point scale), and the linear mapping approach may amplify minor lexical frequency differences. These findings should therefore be interpreted as indicative of modest stylistic shifts rather than substantive changes in empathetic communication. In addition, sentence counts rose significantly, with modest increases in character and word counts, likely reflecting supplementation of missing content [26]. Grok-4 followed a similar strategy but produced longer texts with larger increases in length and sentence count, indicating more active supplementation of absent material, while still improving readability and tone friendliness. However, increased length may also impose practical burdens in counseling settings, highlighting a trade-off between informational completeness and usability. The lack of significant differences between the models in nested sentence ratio and tone friendliness suggests convergence in syntactic and stylistic adjustments [48]. These findings align with prior research. Ali et al [49] reported that GPT-4 simplification of surgical consent forms lowered reading difficulty and enhanced readability, while Beattie et al [50] found LLM-optimized trial consent forms easier to understand. Mirza et al [26] further showed that US trial consent forms are written at overly high reading levels, with each grade-level increase linked to ~16% participant attrition, underscoring the value of simplification. The present study confirms that LLM-based rewriting reduces syntactic complexity and improves readability, with ChatGPT (GPT-5) emphasizing clarity through conciseness and Grok-4 expanding coverage through longer texts, highlighting differences in generation strategies that may shape readers’ experiences.

The analysis of content quality indicates that LLM-based rewriting effectively supplemented key information and enhanced the concreteness of items, particularly in the specificity dimension. However, improvements in operability were relatively limited, suggesting that models still face challenges in generating explicit execution pathways and actionable requirements. Conceptually, operability requires specifying not only what should be done, but also who is responsible, when actions occur, and under what conditions they apply. Such institution-specific procedural details are difficult for LLMs to generate reliably without access to local workflows. Consequently, operability may be less suitable for fully automated optimization and better addressed through human-AI collaboration, with LLMs supporting structural drafting and clinicians refining actionable details. This limited improvement may also reflect the absence of institution-specific “ground truth” information in the source texts and prompts. Because no retrieval-augmented generation or context injection was used, the models lacked access to concrete local procedural details (eg, office hours or emergency contacts), which inherently constrained operability-related gains. Grok-4 showed a more proactive optimization pattern. Taken together, these perspectives were further supported by the findings of the LMM analyses. Previous studies have similarly highlighted the potential of LLMs to improve the quality of ICFs and health-related documents. Shi et al [51] confirmed that LLMs significantly enhanced completeness and specificity in clinical trial consent forms. Oh et al [52] showed that LLMs simplified surgical consent forms while preserving expert-endorsed quality. Rust et al [53] and Romoff et al [54] reported in cardiology discharge summaries and orthopedic educational materials that LLM-based rewriting not only improved readability but also consistently received positive evaluations in content quality. These findings align with the present results, suggesting that LLMs have stable advantages in reinforcing information coverage and refining provisions, while limitations in operability-oriented expressions warrant further investigation.

With regard to practical implementation, operability emerged as a particularly challenging dimension. Indicator-level analyses further revealed that LLM optimization was concentrated on information presentation and provision refinement, with vague or missing clauses supplemented and clarified after rewriting. By contrast, operability and implementation pathways showed only modest gains. While this can be regarded as a limitation at the rewriting level, it also reflects the models’ cautious tendency to avoid generating potentially erroneous or misleading operational details in high-risk contexts, thereby reducing the risk of hallucinations. This trend echoes prior research: Alber et al [55] warned of data poisoning risks in medical corpora, underscoring the need for prudence, while Sebastian et al [56] systematically documented hallucinations in LLM-generated medical texts and emphasized risk evaluation mechanisms. Collectively, these observations suggest that the latest generation of models may exhibit enhanced stability in sensitive contexts. Moreover, this pattern leaves scope for improvement through prompt engineering or human-AI collaboration to guide models toward generating more detailed and personalized operational provisions.

It is noteworthy that cases were observed in which existence was scored as 0, while specificity or operability received nonzero scores. This pattern reflects a distinction between structural explicitness and semantic presence incorporated in the evaluation framework. In the present study, the existence dimension was intentionally defined to capture whether information was explicitly listed as an independent item, rather than whether related content appeared anywhere in the document. This design choice was motivated by the intended audience of counseling ICFs, which primarily consists of nonprofessional readers who rely on clear, structured, and itemized presentation to accurately identify key information. Accordingly, information that was mentioned within other sections without explicit listing could still be evaluated for its specificity and operability once identified, but such nonexplicit presentation may reduce retrievability and comprehensibility, as relevant details—even if included—may not be readily recognized without clear structural cues [57]. Although LLM-based optimization improved overall performance in existence and specificity, fully explicit and well-structured presentation was not consistently achieved across all items [49]. These findings underscore the importance of structural clarity in counseling ICFs and suggest that further improvements may benefit from the integration of professional human review and standardized templates to enhance explicitness and ease of information retrieval [26].

With respect to reading comprehension, LLM-based rewriting significantly outperformed the original texts across all 6 dimensions and the total score, indicating that syntactic simplification and structural optimization enhanced clients’ understanding of ICFs. The 2 LLMs showed broadly similar patterns, with ChatGPT (GPT-5) slightly higher than Grok-4 on most dimensions. Notably, discrepancies arose between the Wilcoxon test and the LMM in model-to-model comparisons: Wilcoxon signed rank tests suggested no significant differences, whereas LMMs indicated that ChatGPT-5 was significantly superior in trustworthiness and acceptability. This discrepancy may reflect the small sample size (10 readers) and only moderate interrater reliability, which limited the power of nonparametric tests to detect differences. In interpreting these findings, we primarily relied on the LMM results, as they explicitly account for both document- and rater-level variability and are better suited to the hierarchical structure of the data. These observations accord with prior research. Will et al [58] reported that LLM-based rewriting improved the readability of online patient education materials without compromising accuracy, while Ali et al [49] found that optimized surgical consent forms enhanced comprehensibility while preserving professionalism and trustworthiness. Together with the present findings, this evidence suggests that LLMs can refine the structure and language of counseling ICFs to improve client comprehension while maintaining credibility and professional integrity.

Overall, this study is the first to systematically validate the feasibility and value of applying LLMs to optimize ICFs for university mental health counseling. The results demonstrated consistent improvements in text structure, content quality, and comprehensibility, with recognition from both professional evaluators and simulated clients. Notably, the baseline content quality scores of the original documents were relatively low (approximately mid-40s out of 100), and the observed gains therefore represent relative improvements rather than the attainment of an optimal standard. Importantly, the magnitude of these improvements was reflected in the *β* coefficients derived from the LMMs, which provide interpretable estimates of practical effect size beyond statistical significance. These improvements suggest that LLMs can function as auxiliary tools in resource-limited higher education settings, enabling the rapid generation of standardized and accessible consent forms to address shortages of trained personnel and professional resources. More importantly, by lowering barriers to comprehension and engagement, LLM-based optimization may facilitate earlier help-seeking, promote more equitable access to counseling services, and strengthen the overall efficiency of youth mental health systems. While rewriting consent forms represents only an initial step, the strong performance observed here underscores the broader potential of LLMs to support standardized [49,51], equitable, and client-centered mental health services for adolescents and young adults worldwide.

In addition, the multidimensional evaluation framework developed in this study offers a methodological reference for future digital mental health research and highlights the value of prompt design and human-AI collaboration in sensitive contexts. Specifically, human-AI collaboration may help address key limitations identified in this study by allowing LLMs to support drafting and structural standardization, while human experts provide contextual judgment, ethical oversight, legal review, and final validation prior to real-world implementation. From a governance perspective, LLM-generated consent materials should therefore be regarded as assistive drafts rather than autonomous outputs, with institutional mechanisms such as mandatory human review and version control playing a central role in mitigating ethical, legal, and accountability risks. In practice, such collaboration may follow a structured workflow, in which LLMs generate an initial draft, counselors or mental health professionals review and refine content for contextual appropriateness and ethical compliance, and institutions provide final oversight and approval prior to deployment.

### Limitations

Several limitations should be acknowledged. First, study materials were limited to Chinese-language ICFs from universities in China, which may constrain generalizability. Direct transfer to other settings may be limited by differences in language structure and readability conventions, as well as jurisdiction-specific legal and ethical requirements (eg, confidentiality exceptions, consent norms, and institutional procedures) and broader cultural expectations regarding counseling relationships. Future research should test applicability in English and other languages and adapt the evaluation framework through context-specific calibration of readability metrics and locally relevant ethical and regulatory standards to support cross-cultural use. Second, despite anonymization and randomization, rater blinding may not have been fully preserved. The substantially longer Grok-4–optimized texts may have been visually distinguishable from the originals, potentially introducing unconscious bias. Although efforts were made to reduce this risk, residual expectancy effects cannot be excluded. In addition, potential rater fatigue and sequence bias should be considered. Each expert evaluated 99 documents across 20 indicators, which may have imposed substantial cognitive load. Although the document order was randomized, fatigue-related effects cannot be fully excluded. Third, the reading comprehension assessment included only 10 volunteers, with moderate interrater consistency and no actual clients, underscoring the need for larger, more representative samples, including real audiences. This small sample size also limited statistical power, particularly for detecting subtle differences between LLM models. Future studies should further validate this evaluation framework against real client decision-making processes and counseling outcomes in applied settings. Moreover, university students may not represent individuals with lower health literacy or acute psychological distress, and documents understandable to college volunteers may still pose barriers to more vulnerable populations. Fourth, although our expert ratings captured multidimensional aspects of content quality (eg, completeness, specificity, and operability), the current evaluation did not systematically verify the factual and legal accuracy of LLM-generated content; given the known risk of hallucinations, optimized forms may inadvertently introduce legally incorrect statements, misinterpret jurisdiction-specific regulations (eg, provisions under the Chinese Mental Health Law), or omit critical ethical requirements despite fluent wording. Because no explicit legal texts were provided to the models, such outputs rely entirely on internal training data, which may be outdated or incomplete. This underscores the necessity of mandatory legal and ethical expert review before any real-world implementation. In addition, aggregate mean scores may obscure isolated but clinically significant errors. For example, LLM-generated drafts could omit mandatory clauses or include contextually inaccurate procedural details despite otherwise strong performance. Future studies should incorporate structured qualitative audits to complement quantitative evaluation. Fifth, this study evaluated only 2 closed-source models, ChatGPT (GPT-5) and Grok-4, without considering open-source systems or alternative architectures. In addition, model outputs were generated within a fixed time window, and future updates to these rapidly evolving systems may lead to different results even under identical prompts. As closed-source commercial systems undergo continuous updates, output behavior may shift over time, and future replications may yield different results due to model drift. Moreover, because outputs were generated via consumer-facing web interfaces rather than application programming interfaces with fixed parameters, unrecorded variables may have limited exact replication of the generation environment and further compounded model drift. Future work should therefore broaden model comparisons and incorporate transparent reporting of model versions and access dates to enhance reproducibility. Sixth, although a multidimensional evaluation framework was applied, this study did not conduct an in-depth analysis of specific textual revisions at the case level; future research could incorporate representative before and after examples to complement the quantitative findings. Finally, improvements in operability-related items were limited, suggesting current models remain insufficient in generating explicit execution pathways; this points to directions such as prompt engineering or human-AI collaboration to enhance operability in future work.

### Conclusion

This study is the first to systematically evaluate the application of LLMs in optimizing ICFs for university mental health counseling. We found significant improvements in structure, readability, content quality, and comprehension, with consistent recognition from both experts and simulated clients. These findings suggest that LLMs can serve as tools to improve currently deficient counseling consent documentation by enhancing clarity and standardization. However, they may not yet produce fully comprehensive or high-quality consent forms without significant human refinement. For universities or primary institutions with limited professional resources, LLMs may serve as practical auxiliary tools to generate accessible, high-quality materials that facilitate early engagement with counseling. Further research is required to evaluate their safety, accuracy, and practical feasibility in real-world counseling workflows.

## Supplementary material

10.2196/86502 Multimedia Appendix 1 Detailed results of the analyses.

10.2196/86502 Multimedia Appendix 2 Supplementary information including prompt instructions, vocabulary lists, and bilingual (Chinese-English) design details.

10.2196/86502 Multimedia Appendix 3 Complete set of documents in 3 versions (Original, ChatGPT5-generated, and Grok4-generated).

10.2196/86502 Multimedia Appendix 4 Source code used for data processing and statistical analysis.

10.2196/86502 Multimedia Appendix 5 Supplementary data including all scoring results across documents and versions.

## Abbreviations

AI: artificial intelligence
ICC: intraclass correlation coefficient
ICF: informed consent form
LLM: large language model
LMM: linear mixed-effects model
