# Supplementary material for: Optimization of University Counseling Consent Forms With Large Language Models: Multidimensional Comparative Evaluation
Source: J Med Internet Res. 2026 Apr 1;28:e86502. doi: 10.2196/86502 (PMC13043017; doi:10.2196/86502)
Supplement: Multimedia Appendix 1 [file jmir-v28-e86502-s001.pdf]

Supplementary Table S1. Document sources

Institution	URL
Central China Normal University	https://www.doc88.com/p-43473276893638.html
University of Science and Technology Beijing	https://xinli.ustb.edu.cn/guizhangzhidu/2022-08-29/189.html
Beijing Normal University at Zhuhai (BNU Zhuhai)	https://www.wjx.cn/vm/hhGOd0x.aspx
Jiangsu University of Science and Technology	https://xsc.just.edu.cn/_t32/2021/0302/c1364a276541/page.htm
Yanshan University	https://xinli.ysu.edu.cn/zxjj/lfzzxxzjlc.htm
Zhengzhou University of Light Industry	https://students.zzuli.edu.cn/2025/0304/c22594a329136/page.htm
Chongqing University of Technology	https://xljk.cqut.edu.cn/info/1075/1030.htm
North China University of Science and Technology	https://mp.weixin.qq.com/s/iwq6Pv-jYhJO_WVFdslGyg
Peking University	https://mp.weixin.qq.com/s/UBx-enTP-3R1PdMU-PItBg
Guangdong Vocational and Technical University of Business and Technology	https://mp.weixin.qq.com/s/FjA2Qz-0PrCdStls2D6z8Q
Chongqing University of Education	https://www.cque.edu.cn/
Southwest University	https://international.swu.edu.cn/
Taiyuan University of Technology	https://student.tyut.edu.cn/info/1224/5018.htm
Northeast Normal University	https://innenu.com/apartment/psychology/agreement.html
Sichuan University	https://en.scu.edu.cn/
Shenyang Medical College	https://dxsxljkjyzx.symc.edu.cn/info/1078/3944.htm
Hefei University	https://www.hfuu.edu.cn/xljk/7a/ee/c9384a97006/page.htm
Henan University of Economics and Law	https://xlzxzx.hafu.edu.cn/info/1015/1672.htm
Kunlun Tourism College, Heilongjiang Institute of Technology	https://www.hljkltc.edu.cn/xsc/info/1391/2152.htm
Jilin International Studies University	https://xgc.jlsu.edu.cn/info/1164/1641.htm
Xuchang University	https://xueshengchu.xcu.edu.cn/info/1040/4137.htm
Shanghai Ocean University	https://xcb.shou.edu.cn/_upload/article/files/2f/82/000690d64dbb8f6fb29ab5d03452/0efea2fb-11ae-47ec-a837-3a21e730adbd.pdf
Mianyang City College	https://xlzx.mycc.edu.cn/info/1033/1134.htm
Huangshan University	https://www.hsu.edu.cn/bb/f5/c18a179189/page.htm
Shandong Agriculture and Engineering University	https://www.sdaeu.edu.cn/xljk/info/1045/1763.htm

Inner Mongolia Agricultural University	https://xinchuang.imau.edu.cn/info/1023/4187.htm
Chongqing Normal University	https://xlzx.cqnu.edu.cn/nr.jsp?urltype=news.NewsContentUrl&wbnewsid=5153&wbtreeid=1457
China University of Petroleum (Beijing)	https://www.cupk.edu.cn/xlzx/c/2017-12-25/492209.shtml
Anhui University of Arts (AHUA)	https://www.ahua.edu.cn/xsc/2022/0706/c762a20049/page.htm
Communication University of Shanxi	https://www.sxcast.edu.cn/uploadfile/ueditor/file/202302/16775540914c2a69.docx
Changchun University	https://xl.ccu.edu.cn/__local/6/7A/31/3085D5C9EBC3111814146BE9294_BA3A0E2F_1955C.pdf?e=.pdf&utm_source=chatgpt.com
Bozhou Vocational and Technical College	https://www.bzy.edu.cn/xsc/xljkjy/gzzd2/content_19085
Chinese Psychological Society – Clinical and Counseling Psychology Registration System (CCPRS)	None (text form)

Supplementary Table S2. Introduction and rationale for the 20 indicators

No.	Indicator	Brief description	APA Ethical Principles and Code of Conduct (2002; revised 2010, 2016; 2025 revision under consultation)	BPS Code of Ethics and Conduct (2018 edition)	Code of Ethics for Clinical and Counseling Psychology, Chinese Psychological Society (2nd edition, Chinese Psychological Society, 2018)	WHO Comprehensive Mental Health Action Plan 2013–2030(2013–2020, extended to 2030 in 2019, revised in 2021)
1	Confidentiality	Counseling content must remain strictly confidential	Standard 4.01 (Maintaining Confidentiality)	Principle of Respect	Article 3.2 Confidentiality Principle	3.3.1 Protection of Privacy and Human Rights
2	Exceptions to confidentiality	Exceptions include self-harm, harm to others, abuse, or legal obligations	4.05(b) Disclosures	Exceptions to Confidentiality	Article 3.3 Exceptions to Confidentiality	3.3.1 Exceptional Circumstances
3	Client rights	Rights include informed consent, withdrawal, and the option to seek a second opinion	3.10 Informed Consent; 10.01	Autonomy	Article 2.2 Right to Informed Consent and Withdrawal	2.1.2 Core Principle of Informed Consent
4	Guardian consent	Requires written consent from guardian	3.10(b)	Consent with minors	Article 2.3 Provisions for Minors	2.1.2 Vulnerable Groups
5	Goals and scope	Clarify counseling objectives and boundaries	3.10(c)	Clarity of role	Article 1.1 Scope of Services	2.1.1 Definition of Service Recipients
6	Format and frequency	Specify mode (in-person/online), duration, and frequency	10.01	Good Practice Guidelines	Article 2.5 Counseling Arrangements	3.2.2 Service Modalities
7	Fees and cancellation policy	Policy on fees, free services, or rescheduling	6.04 Fees	Transparency	Article 2.5	3.2.2 Resource Allocation
8	Recording methods	Whether sessions are recorded; methods of storage	3.10(c); 6.01	Records & confidentiality	Article 3.4 Counseling Records	3.2.3 Data Management
9	Authorization and revocation	Authorization of interns / withdrawal of consent	8.02(b)	Consent and withdrawal	Article 2.6 Authorization and Withdrawal	2.1.2 Informed Consent
10	Crisis procedures	Emergency contacts and crisis intervention procedures	10.10 Termination	Good practice in risk	Article 5.1 Crisis Intervention	3.3.1 Emergency Response

11	Complaints and appeals	Right to submit complaints and feedback	1.04; 1.05	Complaints procedure	Complaint Handling Procedures	3.4.2 Complaint and Appeal Mechanism
12	Data protection	Data retention and destruction procedures	6.01 Documentation	Data Protection Guidance	Article 3.5 Data Security	3.2.3 Data Protection
13	Disclaimer of boundaries	Counseling is not a substitute for medical or legal services	3.10(c)	Clarity of role	Article 1.2	2.1.1 Scope of Services
14	Language clarity	Plain and easily understandable language	3.10(c)	Respect principle	Article 2.1 Linguistic Comprehensibility	WHO Informed Consent Guidelines (2021)
15	Voluntariness	Explicit emphasis on voluntariness	3.10(a)	Autonomy	Article 2.1 Principle of Voluntariness	2.1.2 Voluntariness
16	Client obligations	Obligation to provide accurate information and adhere to agreements	3.1	Responsibilities	Article 2.4 Client Responsibilities	3.2.2 Service Participation
17	Counseling limitations	No guarantee of immediate effectiveness	10.01(c)	Good practice	Article 2.5 Limitations and Disclaimers	3.2.1 Realism and Practical Considerations
18	Counselor qualifications	Counselor must be professionally trained and qualified	2.01 Boundaries of Competence	Professional competence	Article 1.3 Counselor Qualifications	3.1.1 Capacity Building
19	Counseling modalities	Description of methods and psychological assessments	9.03 Assessment	Testing guidance	Article 3.7 Psychological Testing Guidelines	3.2.2 Service Models
20	Target population	Target population: student group, non-medical in nature	3.1	Clarity of services	Preface to the Second Edition	2.1.1 Service Definition

Supplementary Table S3. Assessment of Three dimensions

Dimension	Score range (each indicator)	Brief description	Example
Existence	0-1	Whether the element is present	-
Specificity	0-2	Whether specific details are provided	For confidentiality exceptions, explicitly enumerating "risk to life / legal requirements / abuse" counts as 2 points; only a vague description counts as 1 point; absence counts as 0
Operability	0-2	Whether executable actions or pathways are specified	To withdraw recording authorization, please email ××, template available in the appendix"; "crisis hotline contact (24/7 / campus security / emergency department)

Supplementary Table S4. Complete statistical analysis results for text structure and readability

Indicator	Comparison	*W* statistic	*Z*	*P*-raw	*P*-FDR	*r*	Effect size
Character count	Original vs ChatGPT-5	200.5	-1.43	.16	.17	0.25	small
Word count	Original vs ChatGPT-5	235	-0.81	.43	.44	0.14	small
Sentence count	Original vs ChatGPT-5	10	-4.83	<.001	<.001	0.84	large
Words per sentence	Original vs ChatGPT-5	5	-4.92	<.001	<.001	0.86	large
Characters per word	Original vs ChatGPT-5	24	-4.58	<.001	<.001	0.80	large
Nested sentence ratio	Original vs ChatGPT-5	48	-4.15	<.001	<.001	0.72	large
Lee-Yang Readability Index	Original vs ChatGPT-5	17	-4.71	<.001	<.001	0.82	large
Tone friendliness	Original vs ChatGPT-5	149	-2.35	.03	.04	0.41	medium
Character count	Original vs Grok-4	5	-4.92	<.001	<.001	0.86	large
Word count	Original vs Grok-4	17	-4.71	<.001	<.001	0.82	large
Sentence count	Original vs Grok-4	0	-5.01	<.001	<.001	0.87	large
Words per sentence	Original vs Grok-4	16	-4.73	<.001	<.001	0.82	large
Characters per word	Original vs Grok-4	0	-5.01	<.001	<.001	0.87	large
Nested sentence ratio	Original vs Grok-4	77	-3.64	<.001	<.001	0.63	large
Lee-Yang Readability Index	Original vs Grok-4	68	-3.80	<.001	<.001	0.66	large
Tone friendliness	Original vs Grok-4	144	-2.44	.01	.02	0.42	medium
Character count	ChatGPT-5 vs Grok-4	40	-4.30	<.001	<.001	0.75	large
Word count	ChatGPT-5 vs Grok-4	48	-4.15	<.001	<.001	0.72	large
Sentence count	ChatGPT-5 vs Grok-4	35	-4.39	<.001	<.001	0.76	large
Words per sentence	ChatGPT-5 vs Grok-4	147	-2.39	.02	.02	0.42	medium
Characters per word	ChatGPT-5 vs Grok-4	34	-4.40	<.001	<.001	0.77	large
Nested sentence ratio	ChatGPT-5 vs Grok-4	181	-1.78	.08	0.09	0.31	medium
Lee-Yang Readability Index	ChatGPT-5 vs Grok-4	82	-3.55	<.001	<.001	0.62	large
Tone friendliness	ChatGPT-5 vs Grok-4	263	-0.31	.76	.76	0.05	negligible

W statistic, Wilcoxon signed-rank test statistic.

Z, standardized statistic.

P-raw, unadjusted *p*-value.
P-FDR, *P*-value adjusted by the False Discovery Rate (FDR) method.
r, effect size.
Effect size, interpretative measure of the magnitude of difference.

Supplementary Table S5. Complete consistency analysis results of ratings by 5 experts

Version	Indicator	ICC[a]	95% CI	*F* statistic	*df1*	*df2*	*p*	Consistency level
Original	ICC(2,1)	0.58	0.41-0.73	8.41	32	0	<.001	fair
Original	ICC(2,k)	0.90	0.80-0.94	8.41	32	128	<.001	good
ChatGPT-5	ICC(2,1)	0.35	0.19-0.52	3.66	32	128	<.001	fair
ChatGPT-5	ICC(2,k)	0.74	0.56-0.84	3.66	32	128	<.001	moderate
Grok-4	ICC(2,1)	0.50	0.33-0.65	5.95	32	128	<.001	fair
Grok-4	ICC(2,k)	0.83	0.71-0.90	5.95	32	128	<.001	good
ALL documents	ICC(2,1)	0.50	0.39-0.59	5.95	98	392	<.001	fair
ALL documents	ICC(2,k)	0.83	0.76-0.88	5.95	98	392	<.001	good

ICC[a], ICC < 0.40 (poor), 0.40–0.59 (fair), 0.60–0.75 (moderate), > 0.75 (good).

ICC(2,1) was used for single-rater and ICC(2,k) for average-rater reliability.

F statistic, the test statistic in ANOVA or regression.

df1, numerator df

df2, denominator df

Supplementary Table S6. Complete statistical analysis results for content quality

Dimension	Comparison	*W* statistic	*Z*	*P*-raw	*P*-FDR	*r*	Effect size
Existence	Original vs ChatGPT-5	50.5	-4.11	<.001	<.001	0.72	large
Specificity	Original vs ChatGPT-5	0	-5.01	<.001	<.001	0.87	large
Operability	Original vs ChatGPT-5	211.5	-1.23	.23	.31	0.21	small
Total	Original vs ChatGPT-5	2	-4.98	<.001	<.001	0.87	large
Existence	Original vs Grok-4	30.5	-4.47	<.001	<.001	0.78	large
Specificity	Original vs Grok-4	0	-5.01	<.001	<.001	0.87	large
Operability	Original vs Grok-4	176	-1.87	.06	.10	0.33	medium
Total	Original vs Grok-4	2	-4.98	<.001	<.001	0.87	large
Existence	ChatGPT-5 vs Grok-4	110	-3.05	.002	.004	0.53	large
Specificity	ChatGPT-5 vs Grok-4	134.5	-2.61	.009	.02	0.45	medium
Operability	ChatGPT-5 vs Grok-4	240	-0.72	.48	.60	0.13	small
Total	ChatGPT-5 vs Grok-4	131.5	-2.66	.007	.01	0.46	medium

W statistic, Wilcoxon signed-rank test statistic.

Z, standardized statistic.

P-raw, unadjusted *p*-value.

P-FDR, *P*-value adjusted by the False Discovery Rate (FDR) method.

r, effect size.

Effect size, interpretative measure of the magnitude of difference.

Supplementary Table S7. Complete scores of the 20 indicators across the all versions of documents in 3 dimensions

Indicator	Mean(SD) of Original				Mean(SD) of ChatGPT-5				Mean(SD) of Grok-4			
	Total	Existence	Specificity	Operability	Total	Existence	Specificity	Operability	Total	Existence	Specificity	Operability
Confidentiality	2.92 (0.71)	0.95 (0.10)	0.70 (0.59)	1.27 (0.44)	3.59 (0.69)	0.87 (0.16)	1.47 (0.48)	1.25 (0.46)	3.65 (0.59)	0.90 (0.11)	1.44 (0.49)	1.32 (0.44)
Exceptions to confidentiality	2.75 (0.98)	0.72 (0.34)	0.80 (0.51)	1.24 (0.39)	3.67 (0.77)	0.88 (0.15)	1.51 (0.48)	1.28 (0.41)	3.65 (0.84)	0.87 (0.16)	1.52 (0.43)	1.27 (0.44)
Client rights	3.11 (0.68)	0.90 (0.15)	0.92 (0.52)	1.30 (0.38)	3.38 (0.70)	0.88 (0.15)	1.24 (0.55)	1.26 (0.42)	3.88 (0.66)	0.90 (0.15)	1.73 (0.28)	1.25 (0.44)
Guardian consent	2.69 (0.99)	0.56 (0.43)	1.01 (0.42)	1.12 (0.47)	3.34 (1.00)	0.72 (0.34)	1.46 (0.47)	1.16 (0.42)	2.93 (0.94)	0.55 (0.44)	1.26 (0.46)	1.12 (0.37)
Goals and scope	2.45 (1.22)	0.56 (0.44)	0.69 (0.63)	1.21 (0.42)	3.13 (0.95)	0.77 (0.34)	1.10 (0.55)	1.26 (0.37)	3.58 (0.75)	0.92 (0.12)	1.43 (0.49)	1.23 (0.47)
Format and frequency	3.44 (0.93)	0.82 (0.22)	1.34 (0.63)	1.27 (0.41)	3.38 (0.90)	0.90 (0.13)	1.15 (0.61)	1.33 (0.47)	3.73 (0.92)	0.85 (0.23)	1.58 (0.50)	1.30 (0.46)
Fees and cancellation policy	2.91 (0.80)	0.79 (0.28)	0.82 (0.42)	1.30 (0.41)	3.11 (0.55)	0.88 (0.21)	0.96 (0.17)	1.27 (0.44)	3.16 (0.59)	0.90 (0.15)	0.97 (0.30)	1.29 (0.40)
Recording methods	3.04 (1.07)	0.75 (0.33)	1.11 (0.50)	1.18 (0.49)	3.31 (0.80)	0.85 (0.25)	1.20 (0.48)	1.25 (0.41)	3.39 (0.90)	0.82 (0.28)	1.35 (0.47)	1.22 (0.44)
Authorization and revocation	2.32 (0.76)	0.72 (0.35)	0.39 (0.41)	1.21 (0.45)	2.84 (0.84)	0.82 (0.22)	0.78 (0.50)	1.23 (0.48)	2.78 (0.72)	0.87 (0.15)	0.73 (0.49)	1.18 (0.44)
Crisis procedures	2.59 (0.83)	0.91 (0.12)	0.52 (0.48)	1.17 (0.51)	2.79 (0.80)	0.88 (0.14)	0.72 (0.60)	1.19 (0.43)	2.86 (0.81)	0.87 (0.16)	0.83 (0.55)	1.16 (0.53)
Complaints and appeals	1.46 (0.52)	0.15 (0.19)	0.35 (0.39)	0.96 (0.22)	1.96 (0.64)	0.25 (0.30)	0.78 (0.39)	0.93 (0.24)	2.15 (0.88)	0.41 (0.43)	0.66 (0.45)	1.08 (0.38)
Data protection	1.64 (0.71)	0.24 (0.38)	0.38 (0.37)	1.02 (0.24)	1.96 (0.88)	0.33 (0.38)	0.56 (0.55)	1.06 (0.32)	2.52 (1.01)	0.61 (0.42)	0.76 (0.63)	1.15 (0.39)
Disclaimer of boundaries	1.91 (0.39)	0.07 (0.13)	0.92 (0.33)	0.92 (0.12)	2.12 (0.40)	0.09 (0.11)	1.04 (0.22)	0.98 (0.14)	2.16 (0.36)	0.12 (0.15)	1.10 (0.15)	0.94 (0.18)
Language clarity	1.21 (0.24)	0.12 (0.14)	0.13 (0.14)	0.96 (0.13)	1.19 (0.37)	0.14 (0.21)	0.11 (0.14)	0.95 (0.24)	1.57 (0.68)	0.31 (0.36)	0.19 (0.19)	1.07 (0.30)
Voluntariness	1.66 (0.67)	0.47 (0.41)	0.13 (0.16)	1.06 (0.31)	2.05 (0.63)	0.73 (0.32)	0.16 (0.20)	1.16 (0.39)	1.93 (0.82)	0.60 (0.43)	0.13 (0.22)	1.20 (0.45)
Client obligations	1.76 (0.78)	0.48 (0.43)	0.19 (0.26)	1.09 (0.38)	2.22 (0.98)	0.62 (0.45)	0.43 (0.48)	1.16 (0.43)	2.48 (0.91)	0.72 (0.32)	0.53 (0.55)	1.23 (0.39)
Counseling limitations	1.20 (0.34)	0.08 (0.15)	0.15 (0.14)	0.96 (0.18)	1.33 (0.48)	0.18 (0.29)	0.14 (0.13)	1.01 (0.25)	1.52 (0.72)	0.30 (0.41)	0.21 (0.26)	1.01 (0.30)
Counselor qualifications	2.10 (0.65)	0.77 (0.30)	0.06 (0.13)	1.27 (0.40)	2.25 (0.54)	0.88 (0.16)	0.12 (0.14)	1.25 (0.45)	2.36 (0.57)	0.87 (0.20)	0.13 (0.18)	1.35 (0.43)
Counseling modalities	1.52 (0.70)	0.25 (0.35)	0.27 (0.40)	1.00 (0.26)	2.15 (0.97)	0.28 (0.37)	0.85 (0.61)	1.02 (0.25)	2.45 (0.76)	0.28 (0.38)	1.11 (0.31)	1.06 (0.28)
Target population	2.65 (1.04)	0.73 (0.38)	0.68 (0.63)	1.24 (0.44)	2.77 (1.20)	0.75 (0.38)	0.72 (0.66)	1.30 (0.45)	2.74 (0.87)	0.87 (0.25)	0.60 (0.62)	1.27 (0.44)

SD, standard deviation

Supplementary Table S8. Complete statistical analysis results of the total score for 20 content quality indicators

Indicator	Comparison	*W* statistic	*Z*	*P*-raw	*P*-FDR	*r*	Effect size
Confidentiality	Original vs ChatGPT-5	57.5	-3.98	<.001	<.001	0.69	large
Exceptions to confidentiality	Original vs ChatGPT-5	40	-4.30	<.001	<.001	0.75	large
Client rights	Original vs ChatGPT-5	163	-2.10	.049	.09	0.37	medium
Guardian consent	Original vs ChatGPT-5	72	-3.73	<.001	0.001	0.65	large
Goals and scope	Original vs ChatGPT-5	118.5	-2.89	.004	.01	0.50	large
Format and frequency	Original vs ChatGPT-5	244	-0.65	.69	.75	0.11	small
Fees and cancellation policy	Original vs ChatGPT-5	185	-1.71	.09	.16	0.30	medium
Recording methods	Original vs ChatGPT-5	192	-1.58	.12	.19	0.28	small
Authorization and revocation	Original vs ChatGPT-5	109	-3.06	.002	.009	0.53	large
Crisis procedures	Original vs ChatGPT-5	221	-1.06	.33	.45	0.19	small
Complaints and appeals	Original vs ChatGPT-5	75.5	-3.66	<.001	.001	0.64	large
Data protection	Original vs ChatGPT-5	180	-1.80	.09	.15	0.31	medium
Disclaimer of boundaries	Original vs ChatGPT-5	117	-2.92	.006	.02	0.51	large
Language clarity	Original vs ChatGPT-5	226	-0.97	.51	.64	0.17	small
Voluntariness	Original vs ChatGPT-5	147	-2.39	.02	.052	0.42	medium
Client obligations	Original vs ChatGPT-5	150	-2.33	.02	.052	0.41	medium
Counseling limitations	Original vs ChatGPT-5	227.5	-0.95	.44	.56	0.16	small
Counselor qualifications	Original vs ChatGPT-5	204.5	-1.36	.24	.35	0.24	small
Counseling modalities	Original vs ChatGPT-5	23.5	-4.59	<.001	<.001	0.80	large
Target population	Original vs ChatGPT-5	232	-0.87	.42	.54	0.15	small
Confidentiality	Original vs Grok-4	40	-4.30	<.001	<.001	0.75	large
Exceptions to confidentiality	Original vs Grok-4	39	-4.32	<.001	<.001	0.75	large
Client rights	Original vs Grok-4	30.5	-4.47	<.001	<.001	0.78	large
Guardian consent	Original vs Grok-4	217	-1.13	.28	.40	0.20	small
Goals and scope	Original vs Grok-4	49	-4.14	<.001	<.001	0.72	large
Format and frequency	Original vs Grok-4	175	-1.89	.07	.12	0.33	medium

Fees and cancellation policy	Original vs Grok-4	185	-1.71	.12	.18	0.30	medium
Recording methods	Original vs Grok-4	175	-1.89	.07	.13	0.33	medium
Authorization and revocation	Original vs Grok-4	121.5	-2.84	.004	.013	0.49	medium
Crisis procedures	Original vs Grok-4	174.5	-1.89	.07	.12	0.33	medium
Complaints and appeals	Original vs Grok-4	73	-3.71	<.001	.002	0.65	large
Data protection	Original vs Grok-4	74	-3.69	<.001	.001	0.64	large
Disclaimer of boundaries	Original vs Grok-4	132.5	-2.64	.02	.046	0.46	medium
Language clarity	Original vs Grok-4	163.5	-2.09	.049	.09	0.36	medium
Voluntariness	Original vs Grok-4	179.5	-1.80	.10	.17	0.31	medium
Client obligations	Original vs Grok-4	60	-3.94	<.001	<.001	0.69	large
Counseling limitations	Original vs Grok-4	166.5	-2.04	.04	.09	0.35	medium
Counselor qualifications	Original vs Grok-4	157.5	-2.20	.04	.09	0.38	medium
Counseling modalities	Original vs Grok-4	28	-4.51	<.001	<.001	0.79	large
Target population	Original vs Grok-4	247	-0.60	.61	.69	0.10	small
Confidentiality	ChatGPT-5 vs Grok-4	248	-0.58	.58	.68	0.10	small
Exceptions to confidentiality	ChatGPT-5 vs Grok-4	257	-0.42	.77	.82	0.07	negligible
Client rights	ChatGPT-5 vs Grok-4	87.5	-3.45	<.001	0.004	0.60	large
Guardian consent	ChatGPT-5 vs Grok-4	150.5	-2.32	.03	.06	0.40	medium
Goals and scope	ChatGPT-5 vs Grok-4	165.5	-2.05	.045	.09	0.36	medium
Format and frequency	ChatGPT-5 vs Grok-4	151.5	-2.30	.02	.052	0.40	medium
Fees and cancellation policy	ChatGPT-5 vs Grok-4	238.5	-0.75	.61	.69	0.13	small
Recording methods	ChatGPT-5 vs Grok-4	223	-1.03	.33	.45	0.18	small
Authorization and revocation	ChatGPT-5 vs Grok-4	236.5	-0.79	.55	.66	0.14	small
Crisis procedures	ChatGPT-5 vs Grok-4	226	-0.97	.38	.50	0.17	small
Complaints and appeals	ChatGPT-5 vs Grok-4	250.5	-0.54	.61	.69	0.09	negligible
Data protection	ChatGPT-5 vs Grok-4	151.5	-2.30	.02	.053	0.40	medium
Disclaimer of boundaries	ChatGPT-5 vs Grok-4	230.5	-0.89	.76	.82	0.16	small
Language clarity	ChatGPT-5 vs Grok-4	90	-3.40	.001	.005	0.59	large

Voluntariness	ChatGPT-5 vs Grok-4	223.5	-1.02	.35	.47	0.18	small
Client obligations	ChatGPT-5 vs Grok-4	197.5	-1.48	.15	.22	0.26	small
Counseling limitations	ChatGPT-5 vs Grok-4	246	-0.62	.55	.66	0.11	small
Counselor qualifications	ChatGPT-5 vs Grok-4	204	-1.37	.20	.30	0.24	small
Counseling modalities	ChatGPT-5 vs Grok-4	146.5	-2.39	.02	.052	0.42	medium
Target population	ChatGPT-5 vs Grok-4	260	-0.37	.78	.82	0.06	negligible

W statistic, Wilcoxon signed-rank test statistic

Z, standardized statistic

P-raw, unadjusted *P*-value

P-FDR, *P*-value adjusted by the False Discovery Rate (FDR) method

r, effect size

Effect size, interpretative measure of the magnitude of difference.

Supplementary Table S9. Complete statistical analysis results of the scores of the 20 content quality indicators in the existence dimension

Indicator	Comparison	*W* statistic	*Z*	*P*-raw	*P*-FDR	*r*	Effect size
Confidentiality	Original vs ChatGPT-5	135	-2.60	.08	.28	0.45	medium
Exceptions to confidentiality	Original vs ChatGPT-5	151.5	-2.30	.07	.24	0.40	medium
Client rights	Original vs ChatGPT-5	213.5	-1.20	.69	.80	0.21	small
Guardian consent	Original vs ChatGPT-5	134.5	-2.61	.049	.22	0.45	medium
Goals and scope	Original vs ChatGPT-5	158.5	-2.18	.09	.28	0.38	medium
Format and frequency	Original vs ChatGPT-5	160.5	-2.14	.11	.32	0.37	medium
Fees and cancellation policy	Original vs ChatGPT-5	205.5	-1.34	.34	.53	0.23	small
Recording methods	Original vs ChatGPT-5	145.5	-2.41	.12	.32	0.42	medium
Authorization and revocation	Original vs ChatGPT-5	187	-1.67	.15	.32	0.29	small
Crisis procedures	Original vs ChatGPT-5	133.5	-2.63	.51	.67	0.46	medium
Complaints and appeals	Original vs ChatGPT-5	194	-1.55	.19	.36	0.27	small
Data protection	Original vs ChatGPT-5	160.5	-2.14	.17	.35	0.37	medium
Disclaimer of boundaries	Original vs ChatGPT-5	155.5	-2.23	.34	.53	0.39	medium
Language clarity	Original vs ChatGPT-5	212.5	-1.22	.88	.95	0.21	small
Voluntariness	Original vs ChatGPT-5	110.5	-3.04	.005	.046	0.53	large
Client obligations	Original vs ChatGPT-5	184	-1.72	.14	.32	0.30	medium
Counseling limitations	Original vs ChatGPT-5	162	-2.12	.22	.39	0.37	medium
Counselor qualifications	Original vs ChatGPT-5	70.5	-3.75	.02	.12	0.65	large
Counseling modalities	Original vs ChatGPT-5	192	-1.58	.95	.998	0.28	small
Target population	Original vs ChatGPT-5	159	-2.17	.48	.64	0.38	medium
Confidentiality	Original vs Grok-4	128.5	-2.72	.11	.32	0.47	medium
Exceptions to confidentiality	Original vs Grok-4	123.5	-2.81	.03	.14	0.49	medium
Client rights	Original vs Grok-4	196	-1.51	.75	.86	0.26	small
Guardian consent	Original vs Grok-4	132	-2.65	.22	.39	0.46	medium
Goals and scope	Original vs Grok-4	49.5	-4.13	<.001	.005	0.72	large
Format and frequency	Original vs Grok-4	223	-1.03	.47	.64	0.18	small

Fees and cancellation policy	Original vs Grok-4	167	-2.03	.14	.32	0.35	medium
Recording methods	Original vs Grok-4	197	-1.49	.35	.53	0.26	small
Authorization and revocation	Original vs Grok-4	158.5	-2.18	.10	.32	0.38	medium
Crisis procedures	Original vs Grok-4	173	-1.92	.21	.39	0.33	medium
Complaints and appeals	Original vs Grok-4	115.5	-2.95	.02	.10	0.51	large
Data protection	Original vs Grok-4	72	-3.73	<.001	.02	0.65	large
Disclaimer of boundaries	Original vs Grok-4	177	-1.85	.230	.40	0.32	medium
Language clarity	Original vs Grok-4	132.5	-2.64	.06	.22	0.46	medium
Voluntariness	Original vs Grok-4	187	-1.67	.32	.52	0.29	small
Client obligations	Original vs Grok-4	83	-3.53	.001	.02	0.61	large
Counseling limitations	Original vs Grok-4	136	-2.58	.05	.22	0.45	medium
Counselor qualifications	Original vs Grok-4	161.5	-2.13	.14	.32	0.37	medium
Counseling modalities	Original vs Grok-4	190.5	-1.61	.67	.79	0.28	small
Target population	Original vs Grok-4	163.5	-2.09	.15	.32	0.36	medium
Confidentiality	ChatGPT-5 vs Grok-4	161	-2.14	.32	.52	0.37	medium
Exceptions to confidentiality	ChatGPT-5 vs Grok-4	204	-1.37	.87	.95	0.24	small
Client rights	ChatGPT-5 vs Grok-4	203.5	-1.38	.65	.77	0.24	small
Guardian consent	ChatGPT-5 vs Grok-4	116	-2.94	.03	.14	0.51	large
Goals and scope	ChatGPT-5 vs Grok-4	74.5	-3.68	.01	.09	0.64	large
Format and frequency	ChatGPT-5 vs Grok-4	207	-1.31	.60	.73	0.23	small
Fees and cancellation policy	ChatGPT-5 vs Grok-4	195.5	-1.52	.54	.69	0.26	small
Recording methods	ChatGPT-5 vs Grok-4	192.5	-1.57	.26	.44	0.27	small
Authorization and revocation	ChatGPT-5 vs Grok-4	207	-1.31	.46	.64	0.23	small
Crisis procedures	ChatGPT-5 vs Grok-4	206	-1.33	.59	.73	0.23	small
Complaints and appeals	ChatGPT-5 vs Grok-4	166.5	-2.04	.14	.32	0.35	medium
Data protection	ChatGPT-5 vs Grok-4	142	-2.47	.02	.10	0.43	medium
Disclaimer of boundaries	ChatGPT-5 vs Grok-4	171.5	-1.95	.53	.68	0.34	medium
Language clarity	ChatGPT-5 vs Grok-4	100	-3.23	.01	.09	0.56	large

Voluntariness	ChatGPT-5 vs Grok-4	188	-1.65	.18	.36	0.29	small
Client obligations	ChatGPT-5 vs Grok-4	210.5	-1.25	.44	.64	0.22	small
Counseling limitations	ChatGPT-5 vs Grok-4	189	-1.63	.47	.64	0.28	small
Counselor qualifications	ChatGPT-5 vs Grok-4	208	-1.30	.93	.99	0.23	small
Counseling modalities	ChatGPT-5 vs Grok-4	185	-1.71	.84	.94	0.30	small
Target population	ChatGPT-5 vs Grok-4	132	-2.65	.12	.32	0.46	medium

W statistic, Wilcoxon signed-rank test statistic

Z, standardized statistic

P-raw, unadjusted *P*-value

P-FDR, *P*-value adjusted by the False Discovery Rate (FDR) method

r, effect size

Effect size, interpretative measure of the magnitude of difference.

Supplementary Table S10. Complete statistical analysis results of the scores of the 20 content quality indicators in the specificity dimension

Indicator	Comparison	*W* statistic	*Z*	*P*-raw	*P*-FDR	*r*	Effect size
Confidentiality	Original vs ChatGPT-5	18.5	-4.68	<.001	<.001	0.81	large
Exceptions to confidentiality	Original vs ChatGPT-5	16	-4.73	<.001	<.001	0.82	large
Client rights	Original vs ChatGPT-5	115.5	-2.95	0.005	0.017	0.51	large
Guardian consent	Original vs ChatGPT-5	54	-4.05	<.001	<.001	0.70	large
Goals and scope	Original vs ChatGPT-5	110.5	-3.04	0.005	0.017	0.53	large
Format and frequency	Original vs ChatGPT-5	145.5	-2.41	0.078	0.147	0.42	medium
Fees and cancellation policy	Original vs ChatGPT-5	168.5	-2.00	0.105	0.192	0.35	medium
Recording methods	Original vs ChatGPT-5	222	-1.05	0.387	0.521	0.18	small
Authorization and revocation	Original vs ChatGPT-5	84	-3.51	0.001	0.004	0.61	large
Crisis procedures	Original vs ChatGPT-5	199.5	-1.45	0.256	0.375	0.25	small
Complaints and appeals	Original vs ChatGPT-5	37	-4.35	<.001	<.001	0.76	large
Data protection	Original vs ChatGPT-5	191.5	-1.59	0.358	0.493	0.28	small
Disclaimer of boundaries	Original vs ChatGPT-5	96	-3.30	0.026	0.062	0.57	large
Language clarity	Original vs ChatGPT-5	194	-1.55	0.444	0.555	0.27	small
Voluntariness	Original vs ChatGPT-5	207.5	-1.30	0.605	0.726	0.23	small
Client obligations	Original vs ChatGPT-5	147.5	-2.38	0.027	0.062	0.41	medium
Counseling limitations	Original vs ChatGPT-5	225	-0.99	0.955	1.000	0.17	small
Counselor qualifications	Original vs ChatGPT-5	115	-2.96	0.061	0.121	0.51	large
Counseling modalities	Original vs ChatGPT-5	0	-5.01	<.001	<.001	0.87	large
Target population	Original vs ChatGPT-5	215.5	-1.16	0.718	0.817	0.20	small
Confidentiality	Original vs Grok-4	18.5	-4.68	<.001	<.001	0.81	large
Exceptions to confidentiality	Original vs Grok-4	22	-4.62	<.001	<.001	0.80	large
Client rights	Original vs Grok-4	5	-4.92	<.001	<.001	0.86	large
Guardian consent	Original vs Grok-4	146.5	-2.39	0.026	0.062	0.42	medium
Goals and scope	Original vs Grok-4	48.5	-4.15	<.001	<.001	0.72	large
Format and frequency	Original vs Grok-4	164.5	-2.07	0.058	0.120	0.36	medium

Fees and cancellation policy	Original vs Grok-4	176.5	-1.86	.14	.24	0.32	medium
Recording methods	Original vs Grok-4	147	-2.39	.02	.06	0.42	medium
Authorization and revocation	Original vs Grok-4	103	-3.17	.003	.01	0.55	large
Crisis procedures	Original vs Grok-4	136.5	-2.57	.014	.04	0.45	medium
Complaints and appeals	Original vs Grok-4	76	-3.65	<.001	.004	0.64	large
Data protection	Original vs Grok-4	125	-2.78	.009	.03	0.48	medium
Disclaimer of boundaries	Original vs Grok-4	104.5	-3.14	.011	.03	0.55	large
Language clarity	Original vs Grok-4	166	-2.05	.24	.37	0.36	medium
Voluntariness	Original vs Grok-4	187	-1.67	.44	.56	0.29	small
Client obligations	Original vs Grok-4	125	-2.78	.011	.03	0.48	medium
Counseling limitations	Original vs Grok-4	186	-1.69	.30	.43	0.29	small
Counselor qualifications	Original vs Grok-4	102	-3.19	.03	.07	0.56	large
Counseling modalities	Original vs Grok-4	2.5	-4.97	<.001	<.001	0.86	large
Target population	Original vs Grok-4	253.5	-0.48	.82	.91	0.08	negligible
Confidentiality	ChatGPT-5 vs Grok-4	256.5	-0.43	.91	>.99	0.07	negligible
Exceptions to confidentiality	ChatGPT-5 vs Grok-4	274.5	-0.11	.99	>.99	0.02	negligible
Client rights	ChatGPT-5 vs Grok-4	69	-3.78	<.001	.001	0.66	large
Guardian consent	ChatGPT-5 vs Grok-4	191.5	-1.59	.20	.31	0.28	small
Goals and scope	ChatGPT-5 vs Grok-4	149	-2.35	.02	.052	0.41	medium
Format and frequency	ChatGPT-5 vs Grok-4	90	-3.40	<.001	.004	0.59	large
Fees and cancellation policy	ChatGPT-5 vs Grok-4	260.5	-0.36	.97	>.99	0.06	negligible
Recording methods	ChatGPT-5 vs Grok-4	174	-1.90	.07	.15	0.33	medium
Authorization and revocation	ChatGPT-5 vs Grok-4	231.5	-0.88	.46	.56	0.15	small
Crisis procedures	ChatGPT-5 vs Grok-4	189.5	-1.63	.13	.23	0.28	small
Complaints and appeals	ChatGPT-5 vs Grok-4	186.5	-1.68	.12	.22	0.29	small
Data protection	ChatGPT-5 vs Grok-4	196.5	-1.50	.16	.25	0.26	small
Disclaimer of boundaries	ChatGPT-5 vs Grok-4	152.5	-2.29	.64	.75	0.40	medium
Language clarity	ChatGPT-5 vs Grok-4	169	-1.99	.15	.24	0.35	medium

Voluntariness	ChatGPT-5 vs Grok-4	165	-2.06	.15	.24	0.36	medium
Client obligations	ChatGPT-5 vs Grok-4	191.5	-1.59	.42	.56	0.28	small
Counseling limitations	ChatGPT-5 vs Grok-4	195	-1.53	.28	.41	0.27	small
Counselor qualifications	ChatGPT-5 vs Grok-4	200	-1.44	.70	.81	0.25	small
Counseling modalities	ChatGPT-5 vs Grok-4	160.5	-2.14	.03	.07	0.37	medium
Target population	ChatGPT-5 vs Grok-4	233	-0.85	.45	.56	0.15	small

W statistic, Wilcoxon signed-rank test statistic

Z, standardized statistic

P-raw, unadjusted *P*-value

P-FDR, *P*-value adjusted by the False Discovery Rate (FDR) method

r, effect size

Effect size, interpretative measure of the magnitude of difference.

Supplementary Table S11. Complete statistical analysis results of the scores of the 20 content quality indicators in the operability dimension

Indicator	Comparison	*W* statistic	*Z*	*P*-raw	*P*-FDR	*r*	Effect size
Confidentiality	Original vs ChatGPT-5	210	-1.26	.49	.81	0.22	small
Exceptions to confidentiality	Original vs ChatGPT-5	236.5	-0.79	.50	.81	0.14	small
Client rights	Original vs ChatGPT-5	198	-1.47	.49	.81	0.26	small
Guardian consent	Original vs ChatGPT-5	244.5	-0.64	.61	.82	0.11	small
Goals and scope	Original vs ChatGPT-5	205	-1.35	.30	.81	0.23	small
Format and frequency	Original vs ChatGPT-5	210.5	-1.25	.44	.81	0.22	small
Fees and cancellation policy	Original vs ChatGPT-5	222	-1.05	.57	.81	0.18	small
Recording methods	Original vs ChatGPT-5	203	-1.38	.32	.81	0.24	small
Authorization and revocation	Original vs ChatGPT-5	236.5	-0.79	.93	.99	0.14	small
Crisis procedures	Original vs ChatGPT-5	236	-0.80	.83	.96	0.14	small
Complaints and appeals	Original vs ChatGPT-5	190	-1.62	.40	.81	0.28	small
Data protection	Original vs ChatGPT-5	172	-1.94	.36	.81	0.34	medium
Disclaimer of boundaries	Original vs ChatGPT-5	143.5	-2.45	.03	.59	0.43	medium
Language clarity	Original vs ChatGPT-5	222	-1.05	.72	.86	0.18	small
Voluntariness	Original vs ChatGPT-5	227	-0.96	.41	.81	0.17	small
Client obligations	Original vs ChatGPT-5	224.5	-1.00	.54	.81	0.17	small
Counseling limitations	Original vs ChatGPT-5	194	-1.55	.45	.81	0.27	small
Counselor qualifications	Original vs ChatGPT-5	196	-1.51	.64	.83	0.26	small
Counseling modalities	Original vs ChatGPT-5	199	-1.46	.69	.837	0.25	small
Target population	Original vs ChatGPT-5	228	-0.94	.53	.81	0.16	small
Confidentiality	Original vs Grok-4	239	-0.74	.67	.83	0.13	small
Exceptions to confidentiality	Original vs Grok-4	214.5	-1.18	.43	.81	0.21	small
Client rights	Original vs Grok-4	185	-1.71	.25	.81	0.30	small
Guardian consent	Original vs Grok-4	250.5	-0.54	.89	.99	0.09	negligible
Goals and scope	Original vs Grok-4	261	-0.35	.92	.99	0.06	negligible
Format and frequency	Original vs Grok-4	221.5	-1.05	.57	.81	0.18	small

Fees and cancellation policy	Original vs Grok-4	252.5	-0.50	.92	.99	0.09	negligible
Recording methods	Original vs Grok-4	239.5	-0.73	.58	.81	0.13	small
Authorization and revocation	Original vs Grok-4	194	-1.55	.28	.81	0.27	small
Crisis procedures	Original vs Grok-4	205.5	-1.34	.51	.81	0.23	small
Complaints and appeals	Original vs Grok-4	150.5	-2.32	.09	.69	0.40	medium
Data protection	Original vs Grok-4	152.5	-2.29	.06	.60	0.40	medium
Disclaimer of boundaries	Original vs Grok-4	224	-1.01	.66	.83	0.18	small
Language clarity	Original vs Grok-4	167	-2.03	.17	.81	0.35	medium
Voluntariness	Original vs Grok-4	149	-2.35	.048	.60	0.41	medium
Client obligations	Original vs Grok-4	113.5	-2.98	.008	.28	0.52	large
Counseling limitations	Original vs Grok-4	212	-1.22	.59	.81	0.21	small
Counselor qualifications	Original vs Grok-4	189.5	-1.63	.21	.81	0.28	small
Counseling modalities	Original vs Grok-4	201.5	-1.41	.40	.81	0.25	small
Target population	Original vs Grok-4	219.5	-1.09	.36	.81	0.19	small
Confidentiality	ChatGPT-5 vs Grok-4	206.5	-1.32	.25	.81	0.23	small
Exceptions to confidentiality	ChatGPT-5 vs Grok-4	236.5	-0.79	.76	.90	0.14	small
Client rights	ChatGPT-5 vs Grok-4	221.5	-1.05	.63	.83	0.18	small
Guardian consent	ChatGPT-5 vs Grok-4	220.5	-1.07	.55	.81	0.19	small
Goals and scope	ChatGPT-5 vs Grok-4	190.5	-1.61	.22	.81	0.28	small
Format and frequency	ChatGPT-5 vs Grok-4	201	-1.42	.39	.81	0.25	small
Fees and cancellation policy	ChatGPT-5 vs Grok-4	204.5	-1.36	.49	.81	0.24	small
Recording methods	ChatGPT-5 vs Grok-4	226	-0.97	.56	.81	0.17	small
Authorization and revocation	ChatGPT-5 vs Grok-4	141.5	-2.48	.08	.67	0.43	medium
Crisis procedures	ChatGPT-5 vs Grok-4	225	-0.99	.55	.81	0.17	small
Complaints and appeals	ChatGPT-5 vs Grok-4	132.5	-2.64	.04	.59	0.46	medium
Data protection	ChatGPT-5 vs Grok-4	226.5	-0.96	.43	.81	0.17	small
Disclaimer of boundaries	ChatGPT-5 vs Grok-4	184.5	-1.72	.35	.81	0.30	small
Language clarity	ChatGPT-5 vs Grok-4	51	-4.10	.003	.21	0.71	large

Voluntariness	ChatGPT-5 vs Grok-4	227	-0.96	.57	.81	0.17	small
Client obligations	ChatGPT-5 vs Grok-4	196	-1.51	.26	.81	0.26	small
Counseling limitations	ChatGPT-5 vs Grok-4	241	-0.71	.99	>.99	0.12	small
Counselor qualifications	ChatGPT-5 vs Grok-4	171	-1.96	.14	.81	0.34	medium
Counseling modalities	ChatGPT-5 vs Grok-4	250.5	-0.54	.89	.99	0.09	negligible
Target population	ChatGPT-5 vs Grok-4	198.5	-1.47	.58	.81	0.26	small

W statistic, Wilcoxon signed-rank test statistic

Z, standardized statistic

P-raw, unadjusted *P*-value

P-FDR, *P*-value adjusted by the False Discovery Rate (FDR) method

r, effect size

Effect size, interpretative measure of the magnitude of difference.

Supplementary Table S12. Complete consistency analysis results of ratings by 10 readers

Version	Indicator	ICC[a]	95% CI	*F* statistic	*df1*	*df2*	*p*	Consistency level
Original	ICC(2,1)	0.06	[0.00, 0.15]	1.58	32	288	.02	poor
Original	ICC(2,k)	0.36	[0.00, 0.58]	1.58	32	288	.01	poor
ChatGPT-5	ICC(2,1)	0.15	[0.05, 0.26]	2.53	32	288	<.001	poor
ChatGPT-5	ICC(2,k)	0.62	[0.34, 0.76]	2.53	32	288	<.001	moderate
Grok-4	ICC(2,1)	0.09	[0.01, 0.20]	1.87	32	288	.002	poor
Grok-4	ICC(2,k)	0.48	[0.10, 0.67]	1.87	32	288	.002	fair
ALL documents	ICC(2,1)	0.14	[0.07, 0.21]	2.60	98	882	<.001	poor
ALL documents	ICC(2,k)	0.59	[0.42, 0.70]	2.60	98	882	<.001	fair

ICC[a], ICC < 0.40 (poor), 0.40–0.59 (fair), 0.60–0.75 (moderate), > 0.75 (good).
ICC(2,1) was used for single-rater and ICC(2,k) for average-rater reliability.
F statistic, the test statistic in ANOVA or regression.
df1, numerator df
df2, denominator df

Supplementary Table S13. Complete statistical analysis results for reading comprehension

Indicator	Comparison	*W* statistic	*Z*	*P*-raw	*P*-FDR	*r*	Effect size
Comprehensibility	Original vs ChatGPT-5	1	-4.99	<.001	<.001	0.87	large
Clarity	Original vs ChatGPT-5	0	-5.01	<.001	<.001	0.87	large
Trustworthiness	Original vs ChatGPT-5	18.5	-4.68	<.001	<.001	0.81	large
Friendliness	Original vs ChatGPT-5	40	-4.30	<.001	<.001	0.75	large
Professionalism	Original vs ChatGPT-5	22	-4.62	<.001	<.001	0.80	large
Acceptability	Original vs ChatGPT-5	0	-5.01	<.001	<.001	0.87	large
Total	Original vs ChatGPT-5	0	-5.01	<.001	<.001	0.87	large
Dimension means (Total/6)	Original vs ChatGPT-5	0	-5.01	<.001	<.001	0.87	large
Comprehensibility	Original vs Grok-4	1	-4.99	<.001	<.001	0.87	large
Clarity	Original vs Grok-4	0	-5.01	<.001	<.001	0.87	large
Trustworthiness	Original vs Grok-4	29	-4.49	<.001	<.001	0.78	large
Friendliness	Original vs Grok-4	30.5	-4.47	<.001	<.001	0.78	large
Professionalism	Original vs Grok-4	4	-4.94	<.001	<.001	0.86	large
Acceptability	Original vs Grok-4	4	-4.94	<.001	<.001	0.86	large
Total	Original vs Grok-4	0	-5.01	<.001	<.001	0.87	large
Dimension means (Total/6)	Original vs Grok-4	0	-5.01	<.001	<.001	0.87	large
Comprehensibility	ChatGPT-5 vs Grok-4	198	-1.47	.15	.19	0.26	small
Clarity	ChatGPT-5 vs Grok-4	212	-1.22	.27	.32	0.21	small
Trustworthiness	ChatGPT-5 vs Grok-4	181.5	-1.77	.09	.12	0.31	medium
Friendliness	ChatGPT-5 vs Grok-4	245.5	-0.63	.71	.79	0.11	small
Professionalism	ChatGPT-5 vs Grok-4	197	-1.49	.17	.21	0.26	small
Acceptability	ChatGPT-5 vs Grok-4	163	-2.10	.04	.06	0.37	medium
Total	ChatGPT-5 vs Grok-4	179.5	-1.80	.08	.11	0.31	medium
Dimension means (Total/6)	ChatGPT-5 vs Grok-4	179.5	-1.80	.08	.14	0.31	medium

W statistic, Wilcoxon signed-rank test statistic

Z, standardized statistic

P-raw, unadjusted *P*-value
P-FDR, *P*-value adjusted by the False Discovery Rate (FDR) method
r, effect size
Effect size, interpretative measure of the magnitude of difference.

Supplementary Table S14. Results of the linear mixed-effects model analysis for content quality

Dimension	Comparison	*β*	SE	95% CI	*P*	Variance (rater)	Variance (text)	Residual variance	ICC	Marginal R^2	Conditional R^2
Existence	Grok-4 vs Original	2.45	0.23	2.00-2.90	<.001	<0.001	0.010	4.296	<0.001	0.35	0.35
	ChatGPT-5 vs Original	1.62	0.23	1.18-2.07	<.001	0.072	0.002	4.329	0.016	0.15	0.17
	Grok-4 vs ChatGPT-5	0.82	0.19	0.45-1.20	<.001	<0.001	0.009	3.063	<0.001	0.06	0.06
Specificity	Grok-4 vs Original	6.96	0.30	6.38-7.55	<.001	0.007	1.841	7.362	0.001	1.32	1.52
	ChatGPT-5 vs Original	5.22	0.30	4.63-5.80	<.001	0.243	4.302	7.347	0.020	0.57	0.96
	Grok-4 vs ChatGPT-5	1.75	0.37	1.02-2.47	<.001	0.004	0.002	11.375	<0.001	0.07	0.07
Operability	Grok-4 vs Original	0.74	0.31	0.13-1.35	.02	0.005	32.643	8.013	<0.001	0.00	0.81
	ChatGPT-5 vs Original	0.36	0.26	-0.15-0.87	.17	0.016	33.255	5.609	<0.001	0.00	0.86
	Grok-4 vs ChatGPT-5	0.38	0.35	-0.30-1.06	.27	<0.001	32.471	9.991	<0.001	0.00	0.77
Total	Grok-4 vs Original	10.15	0.62	8.94-11.36	<.001	<0.001	45.191	31.303	<0.001	0.34	0.93
	ChatGPT-5 vs Original	7.20	0.54	6.14-8.26	<.001	<0.001	53.678	23.959	<0.001	0.17	0.86
	Grok-4 vs ChatGPT-5	2.95	0.63	1.71-4.19	<.001	0.135	41.379	33.016	0.002	0.03	0.59

β, regression coefficient

SE, standard error

95% CI, 95% confidence interval

Variance (rater), variance attributable to raters

Variance (text), variance attributable to texts

ICC, intraclass correlation coefficient

Marginal R^2, proportion of variance explained by fixed effects

Conditional R^2, proportion of variance explained by both fixed and random effects.

Supplementary Table S15. Results of the linear mixed-effects model analysis for reading comprehension

Dimension	Comparison	β	SE	95% CI	*p*	Variance (rater)	Variance (text)	Residual variance	ICC	Marginal R^2	Conditional R^2
Comprehensibility	Grok-4 vs Original	0.77	0.05	0.67-0.87	<.001	0.084	<0.001	0.419	0.168	0.29	0.46
	ChatGPT-5 vs Original	0.84	0.05	0.74-0.93	<.001	0.080	<0.001	0.383	0.173	0.38	0.55
	Grok-4 vs ChatGPT-5	-0.07	0.05	-0.16-0.02	.13	0.081	<0.001	0.357	0.185	0.00	0.19
Clarity	Grok-4 vs Original	0.75	0.05	0.65-0.85	<.001	0.056	<0.001	0.421	0.118	0.30	0.41
	ChatGPT-5 vs Original	0.80	0.05	0.70-0.90	<.001	0.068	<0.001	0.395	0.147	0.35	0.49
	Grok-4 vs ChatGPT-5	-0.05	0.04	-0.14-0.04	.27	0.057	<0.001	0.325	0.149	0.00	0.15
Trustworthiness	Grok-4 vs Original	0.31	0.05	0.22-0.39	<.001	0.032	<0.001	0.330	0.089	0.07	0.15
	ChatGPT-5 vs Original	0.41	0.04	0.33-0.50	<.001	0.044	<0.001	0.314	0.122	0.12	0.24
	Grok-4 vs ChatGPT-5	-0.11	0.04	-0.19--0.02	.02	0.028	<0.001	0.324	0.079	0.01	0.09
Friendliness	Grok-4 vs Original	0.37	0.05	0.28-0.46	<.001	0.040	<0.001	0.353	0.101	0.09	0.19
	ChatGPT-5 vs Original	0.39	0.05	0.30-0.48	<.001	0.046	<0.001	0.376	0.109	0.09	0.20
	Grok-4 vs ChatGPT-5	-0.02	0.05	-0.11-0.07	.69	0.061	<0.001	0.334	0.155	<0.01	0.16
Professionalism	Grok-4 vs Original	0.39	0.05	0.31-0.48	<.001	0.052	<0.001	0.338	0.134	0.10	0.23
	ChatGPT-5 vs Original	0.31	0.05	0.22-0.40	<.001	0.075	<0.001	0.333	0.183	0.06	0.24
	Grok-4 vs ChatGPT-5	0.09	0.05	-0.004-0.17	.06	0.041	<0.001	0.341	0.107	0.01	0.11
Acceptability	Grok-4 vs Original	0.45	0.04	0.37-0.53	<.001	0.048	0.114	0.269	0.112	0.12	0.49
	ChatGPT-5 vs Original	0.56	0.04	0.48-0.65	<.001	0.046	0.098	0.302	0.104	0.18	0.50
	Grok-4 vs ChatGPT-5	-0.12	0.05	-0.21--0.03	.013	0.039	<0.001	0.372	0.095	0.01	0.10
Total	Grok-4 vs Original	3.04	0.13	2.78-3.30	<.001	0.541	0.001	2.878	0.158	0.67	0.83
	ChatGPT-5 vs Original	3.31	0.13	3.07-3.56	<.001	0.537	0.002	2.615	0.170	0.87	1.04
	Grok-4 vs ChatGPT-5	-0.28	0.11	-0.50--0.06	.014	0.491	0.001	2.082	0.191	0.01	0.20
Dimension means	Grok-4 vs Original	0.51	0.02	0.46-0.55	<.001	0.015	<0.001	0.080	0.158	0.67	0.83
(Total/6)	ChatGPT-5 vs Original	0.55	0.02	0.51-0.59	<.001	0.015	<0.001	0.073	0.170	0.87	1.04
	Grok-4 vs ChatGPT-5	-0.05	0.02	-0.08--0.01	.014	0.014	<0.001	0.058	0.191	0.01	0.20

β, regression coefficient

SE, standard error

95% CI, 95% confidence interval
Variance (rater), variance attributable to raters
Variance (text), variance attributable to texts
ICC, intraclass correlation coefficient
Marginal R^2, proportion of variance explained by fixed effects
Conditional R^2, proportion of variance explained by both fixed and random effects.
