# Supplementary material for: Optimization of University Counseling Consent Forms With Large Language Models: Multidimensional Comparative Evaluation
Source: J Med Internet Res. 2026 Apr 1;28:e86502. doi: 10.2196/86502 (PMC13043017; doi:10.2196/86502)
Supplement: Multimedia Appendix 2 [file jmir-v28-e86502-s002.pdf]

Supplementary Information S1. Chinese prompts and their corresponding English versions

English version

Assume you are a mental health professional with more than 20 years of experience. Please strictly follow the latest international and domestic guidelines (In case of conflict, the domestic version shall prevail) on psychological counseling ethics and practice to optimize the following informed consent form for psychological counseling.

Requirements:

1. Do not delete any existing core content; add missing elements if necessary.
2. Use clear and concise language that is understandable for university students.
3. Ensure structural standardization with hierarchical numbering and bullet-point formatting to avoid information overload.
4. Maintain a professional, formal, yet friendly tone.

Chinese version

我将给你一份心理咨询咨询同意书，假设你是一位从业 20 年以上的心理咨询工作从业者，请严格依照国际与国内（冲突时以国内为准）最新的心理咨询伦理与实践指南，对以下心理咨询知情同意书进行优化。

要求：

1. 不得删减已有核心内容，必要时补充缺失部分；
2. 语言简洁清晰，适合高校学生群体理解；
3. 结构规范化，采用统一的分级编号与条目式表述，避免信息堆积；
4. 保持专业、正式且友好的语气。

Supplementary Information S2. Table of polite/empathetic words and strict/legalistic words

Polite/empathetic words		Strict/legalistic words	
Chinese version	English version	Chinese version	English version
请	please	必须	must
感谢	thank you	不得	prohibited
谢谢	thanks	严禁	strictly forbidden
欢迎	welcome	否则	otherwise
如有疑问请	if you have questions please	追究	be held accountable
如需帮助	if you need help	终止服务	termination of service
联系我们	contact us	责令	ordered
敬请	kindly	强制	mandatory
歉意	apologies	保留权利	reserve the right
抱歉	sorry	责任自负	at your own risk
理解	understanding	违者	violator
支持	support	处罚	punishment
