# Supplementary material for: Optimization of University Counseling Consent Forms With Large Language Models: Multidimensional Comparative Evaluation
Source: J Med Internet Res. 2026 Apr 1;28:e86502. doi: 10.2196/86502 (PMC13043017; doi:10.2196/86502)
Supplement: Multimedia Appendix 3 [file jmir-v28-e86502-s003.pdf]

Document 01 Original

心理咨询知情同意书

XX 学院大学生心理健康教育与咨询中心是为全校学生提供心理咨询、心理训练、心理健康教育的服务机构，旨在帮助来访者实现自我成长、人格完善、潜能开发健康生活的目标。

为保障心理咨询顺利进行，维护来访者和心理咨询师的权利和义务，对有关事项说明如下:

1.大学生心理健康教育与咨询中心是一个非医疗性质的咨询服务单位，对本校在校学生提供心理咨询，不收取任何费用。

2.心理咨询师须对来访者咨询的内容严格保密，未经来访者的同意不能泄露
给第三方。但以下情况例外:

(1) 来访者有伤害自身或他人的严重危险时:

(2) 来访者有致命的传染性疾病等且可能危及他人时:

(3) 未成年人在受到性侵犯或虐待时

(4) 法律规定需要披露时。

3.心理咨询师为了更有效地协助来访者解决问题，或为接受专业督导或教学与科研的需要，可能要求录音(像)，但在录音(像)之前，必须征求来访者的意见，在征得来访者的书面同意后方可录音(像)，来访者有同意或拒绝的权利。

4.心理咨询每次会谈时间约 50 分钟，每周一次，若有特殊情况经中心同意可增加咨询频次。心理咨询需要有一个过程，一次咨询不一定能解决所有问题，通常需要持续而定期的连续咨询，咨询次数由来访者和咨询师协商。知来访者，因不可抗力因素导致咨询师迟到除外。

5.接受连续咨询的来访者需在每次咨询完后直接和咨询师预约下次的咨询时间。来访者需在预约的时间准时到达，在未提前通知的情况下迟到，咨询师将不相应延长咨询时间。若来访者因故不能按时咨询，请提前一个工作日告知中心改期或取消。咨询师若有事需要改变咨询时间，需提前与中心联系通知来访者，因不可抗力因素导致咨询师迟到除外。

6.来访者有权寻求其他心理咨询师的意见或变更心理咨询师，但需经中心同意。原则上一段时间内，来访者只能与一位心理咨询师咨询，若想转介至其他心理咨询师，应先结束与当前心理咨询师的咨询关系。若咨询师基于理论取向、业务学习等理由需要两位及以上咨询师在场时，需首先征得来访者的同意并报由中心审核决定。

7.来访者在咨询期间，若欲做出重大决定，如自杀、自伤、伤人、出走、离婚、退学、休学等，请务必告知心理咨询师协商处理并签定个人生命安全契约书。否则，将视为咨询关系的自然终止。

8 若来访者或心理咨询师感觉咨询效果不佳，可协商转介事宜。

9.来访者有权接受或停止咨询，也有权决定是否参与咨询过程中的有关活动。

10.来访者在两次咨询的间隔期，如有紧急情况，无须提前预约，可拨打本班辅导员电话，由辅导员进行协助预约。

11.大学生心理健康指导中心仅提供涉及个人成长和自我发展类相关问题的心理咨询，不提供包括精神鉴定、心理诊断、开具精神类药物、出具资格证明、对严重心理障碍患者的长程心理治疗等在内的服务内容。

12.本人已认真阅读并清楚协议内容，同意接受/提供心理中心安排的咨询服务。

签名:________________

________年______月______日

Document 02 Original

为了帮助来访者和咨询师明确各自的权力和责任，为了咨询过程更为顺畅，请您仔细阅读以下内容：

本中心心理咨询师的权利与义务：

1. ***竭诚为来访者提供专业性服务：***心理咨询是咨询师与来访者共同工作的过程，其效果取决于双方的配合等诸多因素。本中心心理咨询师应竭诚为来访者提供高质量专业性服务，态度端正，不得迟到早退无故缺诊。但**不承诺**心理咨询能彻底解决来访者所有问题、或完全达到来访者期待。

2. ***遵守职业伦理：***本中心咨询师应遵守相关国家法律法规和中国心理学会制定的《中国心理学临床与咨询心理工作伦理守则》

3. ***遵守保密原则：***来访者的预约信息、咨询记录、测验结果等所有相关资料均属保密信息，由专人负责保管在中心的专柜里，咨询师不得将咨询记录带出中心。咨询师不得在中心以外的场合（私人或媒体等）谈论来访者信息，若出于专业要求必须进行督导，需要征得来访者同意，并在督导时隐去来访者姓名以及有可能暴露身份的细节。咨询师不可以在咨询时录音录像照相，若出于某种专业需求，需要录音录像，必须征得本中心同意，而且征得来访者在“知情同意书”上签署同意并签名。咨询师不可接触其他咨询师的个案记录。

4. ***打破保密原则的条件：***接待员和咨询师在工作中评估来访者存在或已经存在危害自己或他人生命安全的风险时，有权打破保密原则，应向中心主任、相关主管部门或公安机关报告。有关部门（如公安局、法院等）出示公函的情况下，有权打破保密原则，为其提供来访者相关信息。

5. ***来访者迟到或缺诊时的做法：***来访者迟到或者临时不能来诊而按照中心规定又必须缴费时，咨询师必须在该咨询时段待在诊室里、研究思考该来访者的咨询记录，不得做其他事情。不得为来访者任意延时，必须遵守设置，这是咨询的一部分。

6. ***不得提供心理治疗及精神障碍诊断：***严格按照《中华人民共和国精神卫生法》的相关规定执行，不得接待精神障碍患者。若咨询师评估来访者可能患有精神障碍，建议其到正规医疗机构就诊。

7. ***转介：***若咨询师评估自己不适合该来访者，应该在征得来访者同意以及中心同意之后，负责任地及时**转介**到中心其他咨询师或者其他正规心理咨询或心理治疗机构。

8. ***终止咨询关系：***若来访者连续三次请假，本中心以及咨询师有权自动终止咨询关系。

本中心来访者的权利与义务：

1. ***本中心来访者是指：***在本中心完成了登记、缴费、来访者信息登记表和来访者知情同意书的填写，欲在本中心寻求心理咨询专业服务的人员，包括本人及其监护人。

2. ***来访者应在预约时间内就诊：***若来访者迟到或者早退，咨询师只能在预约时段内提供专业服务。

3. ***来访者应提供本人相关真实信息：***精神障碍患者及家属预约时有义务告知咨询师真实精神障碍的症状及诊断；来访者应提供真实信息，若提供不实信息或有意隐瞒而造成任何损失、伤害或法律后

果，将自行承担后果和相应责任。

4.　　***来访者在咨询期间不做重大决定***：包括自杀、伤害他人、离婚等，若不得不做上述重大决定，必须提前告知咨询师。

5.　　***来访者遵守预约制度：*** 每次咨询后需要缴费方可预约下一个时段。

6.　　***咨询时段调整：***若来访者因故需临时取消预约或更改咨询时间，必须***在前一个工作日的该预约时段之前***通知本中心，费用可以顺延或者退费。若在前一个工作日的该预约时段之后才通知本中心，***咨询费用必须照付***。

违约补偿：为了尊重来访者和咨询师双方的权益，如果咨询师迟到，来访者将免费获得与迟到时间相当的咨询服务；咨询师因故需临时取消预约或更改咨询时间，必须***在前一个工作日的该预约时段之前***通知本中心和来访者。若不能在规定时间内通知来访者，咨询师将为来访者免费提供一次咨询。

7.　来访者或家属须自行记住预约时间，***本中心无提前提醒业务***，如不清楚可自行联系中心确定。

8.　　***终止咨询关系及退费：***咨询关系结束后，若来访者已缴纳咨询费用且***按照制度取消咨询***后尚未接受心理咨询服务，请来访者本人（或费用缴纳者）于***费用缴纳之日起 3 个月内***前来医院退费。

Document 03 Original

心理咨询知情同意书

为保证心理咨询工作的专业性和效果，更好地开展心理咨询工作和保护来访者权益，特提出关于心理咨询的一些说明。请仔细阅读并理解下列项目后，在最后面签上你的姓名。

1. 来访者可以自由选择是否开始或者维持一段咨询关系，且有权充分了解关于专业工作的过程和心理咨询师的专业资质及理论取向。

2. XX 大学心理素质教育中心（以下简称“心理中心”）对在校学生提供免费的心理咨询服务。凡 XX 大学在校学生，原则上每学期可接受不多于 8 次咨询（面对面咨询与远程网络咨询均计入次数），一般情况下，频率为每周 1 次，每次咨询时间 50 分钟，若有特殊情况可与咨询师讨论更改咨询设置。

3. 咨询地点为 XX 大学心理素质教育中心（7 斋东侧一层心理素质教育中心）。

4. 心理咨询师在咨询工作中收集的来访者的个人资料，包括个案记录、测验资料、信件、录音、录像等资料，均属专业信息，咨询师及其他能接触到来访者此类资料的心理中心工作人员，须严格遵守保密原则，但下列几种情况属于保密例外：（1）来访者可能存在自杀、自伤、杀人、伤人等风险；（2）不具备完全民事行为能力的未成年人、精神病人等受到虐待、忽视等；（3）法律规定需要披露的其他情况。

5. 咨询师出于保障服务质量或提升专业胜任力需要进行督导、案例研讨、写作或科研研究等时，须事先与来访者讨论，在获取来访者同意后进行，且必须隐去可能暴露来访者身份的个人信息。无特别约定，咨询师和来访者均不得对心理咨询会谈进行录音录像。咨询师只有在征得来访者书面同意的前提下，才能对咨询过程进行录音或录像。未经书面许可，来访者不得对咨询过程进行录音录像，否则咨询师有权立刻终止咨询，并保留依法申诉的权利。

6. 预约咨询成功后，来访者应记住咨询时间并按时前来，如无变更，将不再有通知和提醒。来访者须在预约时间前到达心理中心接待室，由接待员引导进入咨询室接受咨询。来访者如果在未取消预约时间的情况下迟到，咨询师将不相应延长咨询时间。

7. 来访者如需改变或取消已经预约好的咨询时间，必须提前 24 小时及以上告知心理中心（62335225）。24 小时之内改变咨询时间或无故缺席记为一次临时未到，连续两次或者累计三次临时未到则取消咨询，一个月内无法预约咨询，一个月后方可重新进入预约队列。

8. 咨询师如果要改变或取消已经预约好的咨询时间，必须提前 24 小时及以上通知心理中心，由心理中心及时通知来访者。

9. 咨询师与来访者共同努力建立平等、尊重、合作的专业关系，不发展咨询以外的任何私人关系。来访者有权在任何时候终止咨询或更换咨询师，但出于对来访者有益的考虑，应尽可能与当前咨询师讨论之后再结束咨询或更换咨询师。

10. 心理咨询师为来访者提供的是心理咨询服务。经过咨询师评估后，如果认定来访者的问题不属于心理咨询范围，咨询师有责任终止咨询关系，告知来访者及其亲属或监护人，转介相关医院或机构。若来访者放弃就医或转介，则需了解并承担咨询效果无法达到预期的情况。如因来访者故意隐瞒或提供虚假信息导致延误就医，咨询师与心理中心不承担延误就医的责任。如果就医后医院根据来访者的情况建议辅助支持性心理辅导，且咨询师本人同意的情况下，咨询师可以进行支持性心理辅导。

11. 来访者在两次咨询的间隔期，如遇到特殊情况，工作时间可以拨打办公电话（62335225）；非工作时间紧急情况可以拨打心理援助热线（可通过“北科心理”公众号，点击“专业服务”-“转介信息”获取援助热线信息）

12. 本协议一式两份，来访者签字后生效，请妥善保管。

我已经阅读并同意上述条款。（请在下方横线上抄写左侧句子并签名。）

我是否同意咨询师将我的案例在符合伦理条例的前提下进行督导?
同意　不同意

来访者签名:　　　　　　　　　　日　　　期:

咨询师签名:　　　　　　　　　　日　　　期:

Document 04 Original

心理咨询知情同意书

亲爱的同学:

你好，欢迎你来到心理咨询室。每个人都希望自己拥有更多的快乐和成功，你的到来表明了你对自己的负责态度。研究证明，心理咨询有助于帮助个体更好的自我认识、自我发展，咨询的效果有赖于你和咨询师的共同努力。

为了维护你的权益，协助你拥有更合适的咨询师，请认真阅读:

我的权利：

我可享受免费咨询。咨询师的工作报酬由**学校承担**。**咨询内容**主要是环境适应、学业、情绪、人际关系、恋爱、婚姻和性心理、自我发展、家庭关系、职业生涯规划、身心疾病、压力调节等。

个人隐私均被保密。我知道我的个人信息、所述经历、体验等内容将严格保密。但如果我的叙述涉及个人、他人、社会安全时，咨询师有义务将有关信息依法报告给相关部门。为提高咨询水平，保证我接受高质量的咨询，有时需要在咨询过程中录音、录像，用于督导、研讨或教学研究。如有录音、录像的要求，咨询师会事先征得我的同意，隐去我的背景资料，并签署保密协议。我知道所有资料只限于专业人员使用，绝不会有非专业人员涉及。

我有权选择、更换咨询师。每个咨询师的咨询风格有所不同，我可以根据我的意愿选择合适的咨询师。如果我感到咨询师的方法并不适合我，我可以要求再选择其他更合适的咨询师，进行深入的谈话。

咨询既有收益又有风险。咨询的收益是通过咨询排解心理困扰、开发心理潜能、促进个人成长。咨询的风险包括谈起不愉快的往事，激发起强烈的情绪等。在咨询过程中保持积极、开放和诚实的态度非常重要。

我有权中止咨询。如果在咨询过程中，我感觉到受侵犯、伤害或感觉不舒服、无需再进行咨询时，我可以提出中止咨询，也可以提出更换咨询师的要求。

我需要做的：

专业设置。我知道咨询一般须提前预约，预约时间为工作日的 10：00-13：00，16：00-19：00。我将根据约定时间提前 10 分钟来到咨询室，如果有特殊原因不能前来，我将至少提前 24 小时告知咨询师，取消预约。我知道咨询是一个过程，不能一蹴而就。每次咨询时间为 50 分钟，可以多次预约，时间间隔为每周一次，或与咨询师共同商量而定。如果我处于紧急状态，学工部会立即为我安排合适的咨询师。

信息登记。为了帮助我整理思路，以及在最短时间内，了解我的基本情况，在**第一次**咨询开始前，我知道要填写信息登记表的各项内容，并交给咨询师。

反馈问卷。为了让我和其他同学获得高质量的咨询，**咨询结束后一周内**，我愿意填写匿名的《咨询反馈问卷》，我填写的反馈问卷将被保密，并且只用于改进和提升咨询工作。我愿意填写真实的感受，以促进心理咨询质量的提高。

我已认真阅读上述内容，并对整个过程中我的权利、义务有清楚地认识和了解。

Document 05 Original

知情同意书

欢迎您来到我中心参与进行心理咨询,为明确您与咨询师各自的权利与责任,在咨询开始前,请您认真阅读以下内容:

本协议为 XX 大学心理服务中心的电子知情同意书，与纸质知情同意书具有同时等效力。

一、本研究为北京师范大学临床与咨询心理方向研究生的毕业设计项目,咨询全程公益免费。

二、本协议中的咨询双方应均为完全行为能力人,未成年人应委托监护人履行协议认同手续。本协议不针对无行为能力人。来访者或其监护人在本知情同意书中应以真实姓名签署，并在咨询申请表单等材料中提供真实、正确信息（包括但不限于联系电话、出生年份、紧急联系人、是否接受过精神科相关的诊断或治疗、是否有过自伤/自杀的想法或行为等），若来访者或监护人刻意隐瞒或虚构上述信息事实，则来访者自行承担由此导致的全部责任。

三、根据《中华人民共和国精神卫生法》等法律法规，本中心不提供心理治疗及精神障碍诊断、治疗等相关服务；如果咨询过程中，咨询师发现您可能存在上述情况，将建议到法定医疗机构就诊。

四、心理咨询是来访者与咨询师共同工作的过程，其效果取决于双方配合等多种因素。本中心咨询师均遵循相关法律规定和工作伦理，使用有临床研究基础、有专业共识并证实有效的流派技术，但也无法承诺必将解决您的问题。请您与咨询师一齐努力，制定并达成合理的咨询目标。

五、中心的实习咨询师为我部经过系统化专业培养的应用心理学专业硕士临床与咨询心理方向在读研究生，通过考核后在督导师的监督保障下提供咨询服务。

六、您在使用中心提供的各项免费服务的同时，应承诺接受并遵守各项相关规则的规定。中心有权根据需要不时地制定、修改本协议和/或各类规则，如有任何变更，中心将以公示的方式进行公告，不再单独通知每个人。变更后的协议和规则一经公布即自动生效，成为本协议的一部分。如您不同意相关变更，应当立即以书面通知的方式终止本协议；任何修订和新规则一经公布即自动生效，成为本协议的一部分。

七、关于保密性

通常情况，只有您的咨询师、个案督导老师、论文指导老师及论文答辩委员会成员才能接触到您相关的咨询信息,任何涉及可能显示您个人身份、咨询情况的信息,都将受到严格保护,包括在下述情况:

1. 根据咨询设置，为保证咨询效果，实习咨询师须接受督导（例如：个体督导、团体督导）。我们对您的个人信息严格保密，经过您的书面同意后，您个人信息的查看范围仅限于咨询师本人、个案督导老师、论文指导老师及论文答辩委员会成员，相关人员均承诺对相关内容严格保密，并仅用于学术和督导，绝不对外泄露。

2. 咨询师可能会使用个体咨询的过程和结果作为撰写硕士学位论文的一部分，在论文中不会出现您的任何个人身份信息。您对此项研究结果享有知情权，如您想了解，可与咨询师联系。

3.上述全部工作人员将签署保密协议，不外泄您个人身份、预约情况、咨询情况在内的任何咨询信息。

4.只有在中心与您双方协商一致、经您书面授权的情况下，中心方可将您授权的信息告知给指定第三方。

八、保密例外

1.经咨询师评估，发现有精神疾病倾向，可能对您自身或他人的生命安全构成严重危害的情况；不具备完全民事行为能力的未成年人等遭受或者疑似遭受不法侵害以及面临不法侵害危

险的情况，咨询师有权利直接联系对您及他人生命安全负责的相关人员或相关机构。
2. 司法机构强制要求配合。
九、录音录像
本中心提倡对咨询过程录音录像，以协助咨询师反思咨询过程，提高督导效率，保证咨询效果；由此为确保咨询质量，所有实习咨询师的咨询都需要全程录像（音），实习咨询师的督导师将定期使用录像（音）进行督导。咨询师个人承担录音录像文件的保密保管责任，咨询师将严格保管您的录音/像资料，但录像（音）并非咨询记录或医疗记录的一部分，保密原则同样遵照上述条款。无特别约定或者另签订协议，来访者不得对咨询过程进行录音或者录像。
十、咨询设置
1. 遵照心理咨询设置要求，咨询通常一周一次，每次咨询时间为 50 分钟（家庭咨询时间可为 80 分钟/次），填写量表的时间不包含在咨询时间内。请按约定的时间登录网络账号或到达约定的咨询地点。如您迟到，咨询仍在原定时间结束；如咨询师迟到，将补足迟到的咨询时间。
2.预/续约成功后，您和咨询师双方均可提出更改咨询。如您有要事与咨询冲突，请务必在咨询前 24 小时提出更改；如您在咨询前 24 小时之内更改咨询，您只能将咨询更改至后天或之后的时间。
3.如您连续两次 24 小时内主动取消预约或爽约达到两次，在 3 个月内将不能再在中心预约咨询。
4.您和咨询师双方均有权在咨询过程中的任何时间提出终止咨询，但我们希望您务必和您的咨询师妥善商量此事，至少进行一次结束性会谈后，不再续约。
5.请您实名签署该协议；并承诺在接受中心服务期间，只同时与一位咨询师工作；若发现您可能同时预约了其他咨询师，中心将视情况决定是否再向您提供咨询服务。
6.如您选择网络咨询方式，中心无法保证网络服务的及时性、安全性、准确性。不论在何种情况下，均不对由于网络连接故障，电脑，通讯或其他系统的故障，电力故障，罢工，劳动争议，暴乱，起义，骚乱，生产力或生产资料不足，火灾，洪水，风暴，爆炸，不可抗力，战争，政府行为，国际、国内法院的命令或第三方的不作为而造成的不能服务或延迟服务承担责任。
十、其他
本协议纸质及电子形式具有同等法律效力，来访者签字提交后生效。电子知情同事书可在提交前使用预览模式打印保留（PC 端）/或截图保存（移动端），请妥善保管。如来访者是未成年人，须同时由监护人签字，否则在未得到其监护人签字的情况下咨询师有权拒绝或终止咨询。成年来访者如果两人（及以上）一同前来，请同时签署。在来访者退出咨询后，协议终止。

Document 06 Original

心理咨询服务相关说明（知情同意书）

亲爱的同学，欢迎你选择我院心理健康教育中心为你提供心理咨询服务，请在正式咨询前阅读下列说明：

1.学院心理咨询以助人自助为基本原则，适合诉求缓解心理困扰、帮助自我成长的健康人群。咨询师们持证上岗，采用心理咨询的专业技术并通过以谈话为主的方式，帮助来访者认识和应对生活中的困扰。

2.来访者的个人信息及咨询内容将严格保密，未经本人同意，咨询师不得公布或传播来访者个人信息。但下述情况保密例外：(1) 来访者有伤害自身或伤害他人的严重危险时；(2) 未成年人在受到性侵犯或虐待时；（3）法律规定需要披露时。

3.心理咨询服务有时间限制，一般一次咨询为 50 分钟，通常需要持续而定期进行，咨询频率一般为每周一次，在每次咨询结束后预约下次咨询的时间。请你在预约时间内准时到达心理咨询室接受咨询。如果要改变或取消已经预约好的咨询时间，敬请提前 24 小时电话通知。

4.来访者进入心理咨询室的求助属自愿行为，咨询过程中应尽可能提供真实、完整的求助信息，提供虚假或失真信息，对咨询效果和来访者本人可能存有不利影响。

5.心理咨询不能替代心理治疗,对于精神分裂症、情感障碍等精神疾病，心理咨询师有建议就诊和转介的告知义务。来访者有义务真实、全面地告知咨询师既往咨询和治疗经历，以便于咨询师对来访者的情况进行适切的评估。咨询师结合综合评估来决定你的情况是否需要医疗诊治。

6.你有权在任何时候终止咨询，并请及时告知咨询师。如果你愿意，咨询师可以为你推荐其他心理咨询师。

7.关于上述设定，如果存有任何疑问，你可以向咨询师提出并充分讨论，并在此基础上决定是否开始或继续咨询。

我已经阅读并理解了上述信息，我选择 XX 大学心理健康教育中心的心理咨询服务。

来访者签名：

日期：　　　　年　　月　　日

Document 07 Original

学生心理发展指导中心(以下简称“中心”)是为全体在校本科生提供心理健康教育与咨询的服务机构，旨在帮助学生追求自我成长、人格完善、潜能开发、快乐生活。

所有前来寻求心理咨询服务的学生均享有下述的权利与义务:

1. 中心对学生提供的心理咨询服务，不收取任何费用。
2. 心理咨询师对学生咨询的内容保密，在征得学生同意后才会告知相关人士。但下列特殊 情况例外: (1)学生有伤害自身或他人的严重危险时；(2)法律规定心理咨询师有通报责任 时。
3. 心理咨询师为了有效地协助学生解决问题，或为接受专业督导以及教学与科研的需要， 可能要求录音(像)。这必须征求学生的意见，学生有同意或拒绝的权利。
4. 心理咨询以每周晤谈一次为原则，每次约 50 分钟。若有特殊情况经中心同意可增加咨询 次数。若学生因故不能按时咨询，必须提前 24 小时告知中心改期或取消。
5. 学生有权寻求其他心理咨询师的意见或更换心理咨询师，但需经中心同意。原则上一段 时间内，学生只能与一位心理咨询师咨询。若想寻求中心其他心理咨询师的协助，应先 结束与当前心理咨询师的咨询关系。
6. 学生在咨询期间，若欲做出重大决定，需告知心理咨询师以寻求专业意见后再做决定，这将使学生的决定更为合理。
7. 只进行一次心理咨询难以解决所有的问题，可能需要一段时间的连续咨询。
8. 学生有权接受或停止咨询，也有权决定是否参与咨询过程中所进行的有关活动。
9. 学生在两次咨询之间的间隔期，如遇到紧急情况，可拨打中心电话: 85099129，每天(8:30 - 11:00、13:30 - 20:00)；在上述这些时间之外，可拨打长春市心理援助热线: 12320，0431-89685000，0431-89685333。也可拨打全国心理援助热线: 800-810- 1117(仅限固定电话拨打)；010-82951332(固定电话、移动电话均可拨打)。
10. 中心仅提供涉及学习生活、个人成长和自我发展类的相关问题心理评估和咨询，不提供 包括精神鉴定、诊断、处方精神类药物、出具资格证明、对严重心理障碍患者的长程心 理治疗等在内的服务内容。(如果学生可能患有某种精神障碍，请先去相关专科医院就诊。

Document 08 Original

心理咨询来访者知情同意书

亲爱的来访同学:

你好!

心理咨间是心理咨询师与来访者之间建立的一种专业助人关系，其目的是使来访者从咨询师提供的专业服务中获益。本中心希望通过知情同意的过程，明确双方的权利与义务，以保障心理咨询顺利开展。

本中心向本校正式注册的学生提供专业心理咨间服务，不提供精神障碍的诊断、治疗处方开具、医疗证明开具和心理治疗等服务。

以下是本中心；希望您在接受心理咨询之前需要了解的信息:

一、心理咨询设置

（一）心理咨询预约。原则上需要有来访意愿的同学主动提前预約。

（二）心理咨询地点。所有咨询均在本中心指定地点进行。

（三）心理咨询时间。按本中心提供的咨询时间范围内预约，一般情况下每周一次，面谈心理咨询的时间为 50-60 分钟。如果咨询情况较为复杂，可以适当延长至 90 分钟。

二、咨询师的责任与义务

（一）保密

凡是能接触到来访者的个人资料和会谈信息的咨询师及中心工作人员，都必须严格遵守保密原则。为保障服务质里，在隐去可识别身份的信息后，来访者的案例信息可能被用于本中心研讨和督导，所有参与人会承诺保密：所有案例相关信息运用都需遵守专业伦理规范。

（二）保密例外

1.来访者有伤害自身或伤害他人的严重危险时;

2.不具备完全民事行为能力的未成年人等受到性侵犯或虐待时;

3.法律规定需要披露时。

（三）录音录像

无特别约定，咨询师和来访者皆不得对心理咨询会谈进行录音录像。为保障服务质里，提升专业胜任力，咨询师如需录音录像需要和来访者另签知情同意协议。

（四）缺席

资访双方均需遵守咨询设置。如咨询师未提前 24 小时告知中心的缺席，应为来访者补足咨询时间。

三、来访者的权利与义务

心理咨询是来访者与咨询师共同工作的过程，其效果取决于双方配合等多种因素。来访者应向咨询师告知其心理问题的真实情况，包括其他专业机构对其心理问题的评估诊断：积极主动与咨询师一起探

索解决问题的方法，完成双方商定的作业。来访者应自行承担未如实告知个人真实情况所造成的不良后果。

（一）来访者有权提出更换咨询师。

（二）来访者有权终止咨询，但建议在咨询中与咨询师充分讨论后，由双方协商决定。

（三）改期或取消均需至少提前 24 小时告知中心办理。

来访者未有明确原因取消次数一学期累计达到 3 次，须重新预约等候。如来访者迟到，咨询时间不予补足或延长，如来访者退到时间超过 30 分钟或缺席，当次咨询取消。

四、转介

初始访谈评估后，如来访者的情况不属于本中心工作范畴，咨询师根据本中心规定予以解释并告知合适的求助资源；如果来访者的咨询议题超出了咨询师的工作能力，咨询师应与来访者协商并妥善安排转介咨询事宜。

五、费用

本中心对在校学生提供免费的心理咨询服务。

六、其他情况

本文件一式一份，由心理健康教育与咨询中心保存。

希望你能在咨询前充分了解上述情况。

我已经阅读并充分理解了以上内容。我愿意在广东工商职业技术大学心理健康教育与咨询中心接受心理咨询服务:

来访者签字:

日期:

咨询师签字:

日期:

Document 09 Original

心理咨询知情同意书

为了给来访者提供专业、有效、安全的心理咨询服务，维护来访者和心理咨询师的权益，保证心理咨询的顺利进行，现就心理咨询的基本原则、来访者和心理咨询师的权利和义务等做如下说明：

1. XX 大学大学生心理健康教育与咨询中心是一个非医疗性质的咨询服务单位，对本校在校学生提供心理咨询，不收取任何费用。

2. 心理咨询保密原则：保密是心理咨询的重要工作原则。心理咨询师须对来访者咨询的内容严格保密，一般来说，来访者是否接受过心理咨询以及咨询的内容都不会被透漏给中心以外的非专业人员。但下述几种情况除外：

1）来访者出现自我伤害或伤害他人的倾向；

2）来访者的问题涉及法律责任；

3. 心理咨询师为了更有效地协助来访者解决问题，或为接受专业督导或教学与科研的需要，可能要求录音（像），但在录音（像）之前，必须征求来访者的意见，在征得来访者的书面同意后方可录音（像）， 来访者有同意或拒绝的权利。

4. 为了能够更好地帮助来访者，心理咨询师提出个案讨论或申请督导，但仅限于专业场合，同时须隐去来访者的个人信息。

5. 心理咨询师与来访者避免双重关系。咨询师不得与来访者有不符合咨询原则的接触，不得向咨询学生收取费用。

6. 心理咨询每次会谈时间约 50 分钟，每周一次，若有特殊情况经中心同意可增加咨询频次。心理咨询需要有一个过程，一次咨询不一定能解决所有问题，通常需要持续而定期的连续咨询，咨询次数由来访者和咨询师协商。

7. 为了方便后续的跟踪咨询服务，来访者须提供其真实姓名及联系方式，这些资料仅用于心理咨询中心的档案管理，不会透漏给其他任何单位和个人。

8. 来访者有权寻求其他心理咨询师的意见或变更心理咨询师，但需经中心同意。原则上一段时间内，来访者只能与一位心理咨询师咨询，若想转介至其他心理咨询师，应先结束与当前心理咨询师的咨询关系。

9. 来访者向校心理健康教育与咨询中心求助属自愿行为，咨询过程中应自愿提供尽可能真实的求助信息，对于提供虚假或失真信息者，心理咨询师对该情况下的咨询结果及其它相应后果不负责任。

10. 来访者有义务提供专业医疗机构作出的精神疾病相关诊断，不能隐瞒精神类相关病史。

11. 来访者要安心配合咨询员的咨询辅导。在一定的时间里，严格按照心理咨询师的建议，耐心主动地接受辅导。有义务按照咨询师要求到专门医疗机构进行精神疾病鉴别诊断后，在精神科医生指导下通过心理咨询进行辅助治疗。

12. 来访者在咨询期间，若欲做出重大决定，如自杀、自伤、伤人、出走、退学、休学等，请务必告知心理咨询师协商处理。否则，将视为咨询关系的自然终止。

13. 来访者应允许心理咨询师把咨询过程中的相关谈话记录整理成文本档案形式予以存档。

14. XX 大学心理健康教育与咨询中心仅提供涉及个人成长和自我发展类相关问题的心理咨询，不提供包括精神鉴定、心理诊断、开具精神类药物、出具资格证明、对严重心理障碍患者的长程心理治疗等在内的服务内容。

本人已认真阅读并清楚协议内容，同意接受提供心理健康教育与咨询中心安排的咨询服务。

Document 10 Original

心理咨询知情同意书

以下是关于心理咨询的一些基本约定，请仔细阅读并在下面签字表明你已了解相关规定，是自愿参加咨询，并能为自己的行为负责。

1、心理咨询时间每次约为 50 分钟，通常需要持续而定期进行，每周一次。

2、来访者在心理咨询中提供的信息将严格保密，未经来访者书面同意不会泄露给第三者。但是如果出现涉及来访者自身或他人的生命安全的情况（比如自杀倾向，伤害他人的倾向等）保密制度将不能保证。

3、学校咨询室对本校学生免费咨询。

4、长期接受咨询的来访者需在每次咨询完后预约下次的咨询时间。来访者需在预约的时间准时到达。在未提前通知的情况下迟到，咨询师将不相应延长咨询时间。

5、来访者如有特殊情况，需要临时取消或更改已经预约好的咨询时间，请至少提前一天通知咨询师并进行协商。若没有通知而未到，将按来访者主动放弃咨询处理。下周原定咨询时间将不再保留，中心将根据需要安排其他来访者的咨询工作。若仍需咨询，则需要重新预约。

6、咨询师如有特殊情况，需要临时取消或更改已经预约好的咨询时间，同样需要至少提前一天与来访者协商。

7、来访者有权利在任何时候中止咨询。

8、在取得来访者同意后，面谈可能会被录音、录像或被观察，作为督导咨询师的一部分。所有录音或录像内容，除咨询师督导外，在未征得你同意之前，决不会向任何人披露。

我已阅读并理解了上述的信息，且知道我可以询问有关的问题。我同意参加心理咨询。

来访者姓名：__________　　　　咨询师姓名：__________

日期：__________

Document 11 Original

心理咨询知情同意书

以下是关于心理咨询的一些基本约定，请仔细阅读并在下面签字表明你了解相关规定，是自愿参加咨询，并能为自己的行为负责。

1.心理咨询每次约为 50 分钟，通常需要持续而定期进行。

2.你在心理咨询中提供的信息将严格保密,未经你的书面同意不会泄露给第三者。但是如果出现涉及你自己或他人的生命安全的情况(比如自杀倾向，伤害他人的倾向等)保密制度将不能保证。

3.学校咨询室对本校学生免费咨询。长期接受咨询的来访者需在
每次咨询完后预约下次的咨询时间。来访者需在预约的时间准确到达。在未提前通知的情况下迟到，咨询师将不相应延长咨询时间。

4.来访者如果要临时取消已经预约好的咨询时间,请至少提前一天通知我们。若没有通知而未到，我们将按放弃咨询处理。下次你的咨询时间，我们将安排其他人的咨询。你若仍需咨询，需重新预约。

5.咨询师若有事，需要改变咨询时间，会提前告知。

6.你有权利在任何时候中止咨询；但是来访者如果要改变咨询时间设置，请尽早与咨询师协商。

我已阅读并理解了上述的信息，且知道我可以询问有关的问题。我同意参加心理咨询。

来访者姓名:______性别:____年龄:____班级：________

联系电话:____________

咨询师:________日期:__________

Document 12 Original

心理咨询来访者知情同意书

亲爱的来访同学:

您好!

心理咨询是心理咨询师与来访者之间建立的一种专业助人关系，其目的是使来访者从咨询师提供的专业服务中获益。华北理工大学大学生心理健康教育与咨询中心希望通过知情同意的过程，明确双方的权利、责任与义务，以保障心理咨询顺利开展。

本中心向本校学生提供免费的专业心理咨询服务，按照《中华人民共和国精神卫生法》第二十三条的规定，本中心不具备心理、精神疾病的诊断、治疗、处方开具、医疗证明开具和心理治疗等资质。如出现以上可能，本中心将建议学生到符合精神卫生法规定的医疗机构就诊，并将相关信息告知学生所在的学院及辅导员老师，以便于学院给与学生相应的督促和支持。

一、心理咨询设置

心理咨询需要学生提前预约，并在约定的时间准时到咨询中心。提供的咨询时间一般情况下每周一次、每次咨询时间为 50 分钟。

二、咨询师的责任与义务

（一）保密

凡是能接触到来访者的个人资料和会谈信息的咨询师及中心人员，都必须严格遵守保密原则。为保障服务质量，在隐去可识别身份的信息后，来访者的案例信息可能被用于案例研讨和督导，所有参与人会承诺保密；所有案例相关信息运用都需遵守专业伦理规范。

（二）保密例外，下列特殊情况心理咨询师可以突破保密例外:

1.经过评估发现学生有伤害自身、伤害他人人身安全和伤害社会的倾向。

2.经过评估，发现学生有自杀的可能性。

3.来访者有致命的传染性疾病且可能危及他人时。

4.来访者受到性侵犯或虐待以及虐待他人时。

5.法律规定需要披露时。

（三）录音录像

无特别约定，咨询师和来访者皆不得对心理咨询会谈进行录音录像。为保障服务质量，提升专业胜任力，咨询师如需录音录像需要和来访者另签录音录像知情同意协议。

三、来访者的权利与义务

1.心理咨询是来访者与咨询师共同工作的过程，其效果取决于双方配合等多种因素。来访者应向咨询师告知其心理问题的真实情况，包括其他医疗专业机构对其心理问题的评估诊断；积极主动与咨询师一起探索解决问题的方法，完成双方商定的作业。来访者应自行承担未如实告知个人真实情况所造成的不良后果。

2.来访者有权终止咨询，但建议在咨询中与咨询师充分讨论后，由双方协商决定。

3.改咨询时间或取消咨询均需至少提前 24 小时电话告知咨询中心。来访者未有明确原因取消或迟到次数一学期累计达到 3 次，须重新预约等候。如来访者迟到，咨询时间不予补足或延长，如来访者迟到时间超过 30 分钟，本次咨询取消。

四、紧急情况处理

来访者在两次咨询的间隔期，如遇到特殊紧急危机情况，可以拨打河北省 24 小时心理援助热线，热线号码为：0312-96312，0311—68052995，北京市 24 小时心理援助热线，热线号码为：010-82951332，010-58303286；010-58340263。

我已经阅读并充分理解了以上内容。我愿意在 XX 大学大学生心理健康教育与咨询中心接受心理咨询服务。

来访者签名：　　　　　　　　　　咨询师签名：

年　月　日　　　　　　　　　　年　月　日

Document 13 Original

心理咨询知情同意书

亲爱的同学们：您们好！欢迎您的到来！感谢您对我们中心的信任，愿意将您的心灵之声与我们分享，我们将共同经历生命中一段调整方向、触及灵魂的探索旅程。这一程的前方可以部分预期，但又充满未知，结果如何将取决于你我共同的努力，还有社会环境的影响。

为保证咨询服务的顺利进行，保障您的权益不受侵害，我们将从心理咨询基本原则、来访者和咨询师的责任和义务等方面一一为您详述：

一、咨询说明

1、服务对象本中心主要以_为服务对象，从事专业的心理咨询与治疗工作，心理咨询师将会运用专业的心理咨询技术与方法对来访者进行咨询与治疗。

2、收费标准

本中心的心理咨询师免费本校学生提供心理咨询服务。

3、时间准时赴约是咨询的一个良好的开端，您与心理咨询师都应遵守守时原则。一般情况下，心理咨询的时间为 50-60 分钟/次，每周 1-2 次。具体次数与时间安排需要视具体情况由您和您的咨询师双方协商约定。咨询过程中，如您需改变或取消咨询时间，必须提前至少 5 小时通知。咨询师如要改变或取消约定，也必须提前通知您。

4、保密原则

保密是心理咨询的工作原则之一，也是职业道德的集中体现。在咨询过程中，咨询师将在严格保密的情况下保存您的有关资料，包括个案记录、测验资料、信件等。咨询师在获得您允许的情况下可对咨询进行录音及录像。但未经允许不得 将咨询过程中来访者的经历、体验及相关文字、影音文件公开或传播。整个咨询过程将完全保密，但是以下几种情况例外：

A 已经获得您的披露信息授权，心理咨询师应该严格按照约定范围使用该授权；

B 如果您出现严重自我伤害或伤害他人的倾向，为了避免伤害，咨询师有权打破保密原则，通知相关部门及您的家人，但应将有关保密信息的暴露程度限制在 最低范围之内。当然咨询师会在采取任何行动之前，尽量先与您进行协商；

C 如果您的问题涉及法律责任或法庭取证，法律要求咨询师披露的不在保密之列，如杀人事实、谋杀计划、危害公共安全等；

D 为了能更好的帮助您，咨询师有时会进行个案讨论或申请督导，在涉及业内交流时会事先征求您的意见并隐去身份信息。

E 未成年来访者其监护人可以向咨询师申请调阅咨询记录。

二、来访者的权利、责任与义务

1. 您需要以积极的态度对待咨询，尊重咨询师，坦诚地向咨询师提供与心理问题有关的真实资料，完成双方商定的作业。

2. 自愿。您有权决定中止或结束咨询，可以自主选择咨询师，与咨询师协商修正咨询的方向及方法。

3. 自主。你需要积极主动地与咨询师一起探索解决问题的方法，努力实现自我成长，为咨询负责。

三、咨询师的权利、责任与义务

1. 咨询师有权利了解与来访者心理问题有关的个人资料。

2. 咨询师应遵守国家有关的法律法规及职业道德，遵守咨询机构的有关规定。

3. 真诚。热情诚恳地接待每一位来访者，耐心倾听，建立互相信任的咨访关系。

4. 保密。咨询师要严格遵守保密原则，并说明保密例外。对来访者咨询访谈内容严格保密，对有关资料妥善保管，不在咨询室以外的地方随便谈论来访者的相关事宜。
5. 尊重。尊重来访者的思想和意愿，接受来访者的情绪情感。遵守预约时间，如有特殊情况提前告知来访者。
6. 当咨询师个人有限制时，本着对来访者负责的态度，咨询师有权利提出转介或中止咨询，坦诚地告诉来访者并及时转给相关机构。

我已经阅读并理解以上内容，愿意与______心理咨询师建立职业性心理咨询关系，并在咨询过程中履行相应的权利和义务。

谢谢阅读！若您已确定知晓，请在下方签字。

来访者:　　　　　　　　　　　　咨询师:

联系电话:　　　　　　　　　　　联系电话:

Document 14 Original

知情同意书

亲爱的_____同学，你好:

1、协议双方通过签署本协议以规范心理咨询的过程，保证心理咨询的有效进行。

2、咨询地点为学生心理健康教育与咨询中心，每次咨询_90_分钟，每周咨询_1 次，每个学期咨询限 6 次。

3、咨询对象为全日制在读学生，不含休学中的学生。

4、来访者请准时在预约时间到达心理咨询室接受咨询，迟到不延迟咨询时间。

5、来访者如要改变或取消咨询时间，必须提前一个工作日通知中心，否则计入六次咨询之中；如果缺席次数超过 2 次（包括无故不来，临时请假，提前请假），将取消本阶段咨询资格，可重新预约等待；如果无故不来且失联 48 小时，中心会联系院系确保学术安全。

6、来访者可并仅可在第一次咨询结束后可以提出更换咨询师的要求。

7、为尽可能保证大多数同学收益，默认每学期每位同学享有六次咨询权利，如果超过六次，须经评估判断后才可继续咨询。

8、中心具有跟诊规则，前来预约的同学默认统一跟诊，如不同意，咨询将酌情后排。

9、咨询师如需改变或取消已经预约好的咨询时间，须提前一个工作日通知中心。

10、在心理咨询过程中，咨询师要为来访者的隐私保密，来访者在咨询过程中所叙述的经历，体验，未经其本人同意，咨询师不得公布或传播这些信息，如确因学术交流或其他因素的需要，要报告该紧例，需隐去可能辦认出来访者的个人信息。

11、如来访者的行为可能对自己或他人构成严重伤害，或出于司法原国作为呈堂证供，则咨询师可以突破保留原则，问有关单位报告来访者的情况，除此之外，咨询记录将妥善保存在本中心，不提供给包括来访者在内的任何机构与个人。

12、心理咨询中心仅提供涉及学习生活、个人成长和自我发展类的相关问题心理评估和咨询，不提供包括精神鉴定、诊断、处方精神类药物、出具资格证明、对严重心理障碍患者的长程心理治疗等在内的服务内容，如果来访者可能患有某种精神障碍，请先去相关专科医院就诊就治。

13，心理咨询中心承担着对临床专业研究生的培养和教学任务，在日常的咨询中不定期会有研究调查与跟诊的情况，希望得到同掌的支持和协助。

以上全部规定，本人已经阅读，并愿意遵守!

我已阅读并同意

姓名:

Document 15 Original

心理咨询知情同意书

亲爱的来访同学:

您好!

大学生心理健康中心是一个非医疗性质的咨询服务单位，对本校在校学生提供心理咨询，不收取任何费用。不提供精神障碍的诊断、治疗、处方开具、医疗证明开具和心理治疗等服务。

以下是本中心希望您在接受心理咨询之前需要了解的信息:

一、心理咨询概况

（一）心理咨询预约。原则上需要有来访意愿的同学主动提前预约。

（二）心理咨询地点。所有咨询均在本中心指定地点教学楼 104 室进行。

（三）心理咨询时间。按本中心提供的咨询时间范围内预约，一般情况下每周一次、每次咨询时间为 50 分钟。心理咨询需要有一个过程，一次咨询不一定能解决所有问题，通常需要持续而定期的连续咨询，咨询次数由来访者和咨询师协商。原则上每位学生最多可接受 8 次咨询。

二、咨询师的责任与义务

（一）保密

凡是能接触到来访者的个人资料和会谈信息的咨询师及中心工作人员，都必须严格遵守保密原则。所有案例相关信息运用都需遵守专业伦理规范。

（二）保密例外

1.来访者有伤害自身或伤害他人的严重危险时;

2.法律规定需要披露时。

（三）迟到与缺席

咨访双方均需遵守咨询时间。如有特殊情况，咨询师需提前 24 小时告知中心，并与来访者重新约定咨询时间。

三、来访者的权利与义务

心理咨询是来访者与咨询师共同工作的过程，其效果取决于双方配合等多种因素。来访者应向咨询师告知其心理问题的真实情况，包括其他专业机构对其心理问题的评估诊断;积极主动与咨询师一起探索解决问题的方法，完成双方商定的作业。来访者应自行承担未如实告知个人真实情况所造成的不良后果。

（一）来访者有权寻求其他心理咨询师的意见或变更心理咨询师，但需经中心同意。原则上一段时间内，来访者只能与一位心理咨询师咨询，若想转介至其他心理咨询师，应先结束与当前心理咨询师的咨询关系。

（二）来访者有权终止咨询，但建议在咨询中与咨询师充分讨论后，由双方协商决定。

（三）改期或取消均需至少提前 24 小时告知中心。如来访者迟到，咨询时间不予补足或延长，如来访者迟到时间超过 15 分钟或缺席，本次咨询取消。

（四）来访者在咨询期间，若欲做出重大决定，如自杀、自伤、伤人、出走、退学、休学等，请务必告知心理咨询师协商处理，否则将视为咨询关系的自然终止。

四、转介

初始访谈评估后，中心根据来访者情况合理安排后续咨询。如来访者的情况不属于本中心工作范畴，咨询师根据本中心规定予以解释并告知合适的求助资源;如果来访者的咨询议题超出了咨询师的工作能力，咨询师应与来访者协商并妥善安排转介咨询事宜。

希望您能在咨询前充分了解上述情况。

我已经阅读并充分理解了以上内容。我愿意在吉林体育学院心理健康教育中心接受心理咨询服务。

来访者签字:　　　　日期:

咨询师签字:　　　　日期:

Document 16 Original

心理咨询知情同意书

尊敬的来访者:

您好! 江苏科技大学心理健康教育中心提供的心理咨询的服务对象是心理正常的在校大学生，心理咨询是针对大学生在生活适应、学业科研、人际交往、恋爱情感、家庭关系、人生价值、发展规划、压力情绪等方面出现的烦恼与困惑提供帮助的一种方式。本着“以人为本，助人自助”的原则，为了更好地维护来访者和咨询师的权益，提高咨询的质量，现就相关内容做如下说明:

一、心理咨询的设置

1.咨询保密原则：保密是心理咨询的工作原则之一，也是职业道德的集中体现。咨询师对来访者的个人信息及咨询的相关内容严格保守秘密。但下述几种情况除外：（1）来访者出现自我伤害或伤害他人的倾向。（2）来访者的问题涉及法律责任。（3）来访者因心理失常而需要就医。

2.学校心理中心对本校学生免费咨询。1 次咨询时长为 50 分钟，频率一般为 1 周 1 次。咨询次数由来访者和咨询师共同协商。如有特殊原因不能如期咨询，双方都至少提前半天告知心理中心预约人员，并由专人通知对方。

3.一般情况下，一位来访者一周只能预约 1 次咨询，如有特殊情况，需和咨询师进行协商。

4.原则上同一时期只预约一名咨询师，咨询关系结束后，可预约其他咨询师。

5.如果咨询师需要将来访者的咨询进行专业的个案督导，需征得来访者的同意，同时须隐去可辨识身份的个人信息。

6.如果咨询师需要对会谈进行录音或录像，需与来访者另行签订咨询录音/录像知情同意书。

7.为避免双重关系对咨询的影响，咨询师不得接受来访者的礼物，且不得建立咨访关系以外的其他关系。

8.咨询师个人有限制时，须坦诚地告诉来访者并及时转介。

二、来访者的权利与义务

1.自愿。来访者需要主动预约心理咨询，可以自主选择咨询师，并有权决定是否继续咨询或更换咨询师。

2.积极。以积极的态度对待咨询，坦诚地向咨询师表露自己，不掩饰或伪装；努力实现自我成长，为咨询负责。

3.尊重。尊重咨询师，严格遵守约定的咨询时间。

4.坚持。心理咨询不是简单的问题解决。来访者带着自己个人成长中经年累月形成的问题而来，这就决定了心理咨询也需要一个过程。

三、咨询师的权利与义务

1.真诚。真诚对待每一位求助者，耐心倾听，建立互相信任的咨访关系。

2.保密。对来访者的咨询访谈内容严格保密。

3.尊重。尊重来访者的思想和意愿，接受来访者的情绪情感。

四、其他需要约定的内容

1.来访者如有伤害自己及他人的想法或行为，须及时告诉咨询师。

2.在咨询过程中，咨询师应与来访者对工作的重点进行讨论并达成一致意见，必要时应与来访者达成书面协议。

3.知情同意书签订后由咨询师自行保存，并注意保密。

咨询师签名:　　　　　　　　　　　　　　来访者签名:

年　月　日　　　　　　　　　　　　　　年　月　日

Document 17 Original

心理咨询知情同意书

亲爱的来访同学：您好！

心理咨询是心理咨询师与来访者之间建立的一种专业助人关系，其目的是使来访者从咨询师提供的专业服务中获益。本中心希望通过知情同意的过程，明确双方的权利与义务，以保障心理咨询顺利开展。

本中心向本校正式注册的学生提供免费的心理咨询服务，不提供精神障碍的诊断、治疗、处方开具、医疗证明开具和心理治疗等服务。

以下是本中心希望您在接受心理咨询之前需要了解的信息：

一、心理咨询设置

（一）心理咨询预约：原则上需要有来访意愿的同学主动提前预约。

（二）心理咨询地点：所有咨询均在本中心指定地点进行。

（三）心理咨询时间：按本中心提供的咨询时间范围内预约，一般情况下每周一次、每次咨询时间为 50 分钟。

二、咨询师的责任与义务

（一）保密

凡是能接触到来访者的个人资料和会谈信息的咨询师及中心工作人员，都必须严格遵守保密原则。为保障服务质量，在隐去可识别身份的信息后，来访者的案例信息可能被用于本中心研讨和督导，所有参与人会承诺保密；所有案例相关信息运用都需遵守专业伦理规范。

（二）保密例外

1、来访者有伤害自身或伤害他人的严重危险时；

2、不具备完全民事行为能力的未成年人等受到性侵犯或虐待时；

3、法律规定需要披露时。

（三）录音录像

无特别约定，咨询师和来访者皆不得对心理咨询会谈进行录音录像。为保障服务质量，提升专业胜任力，咨询师如需录音录像需要和来访者另签知情同意协议。

（四）迟到与缺席

咨访双方均需遵守咨询设置。如咨询师未提前告知中心的迟到或缺席，应为来访者补足咨询时间。

三、来访者的权利与义务

心理咨询是来访者与咨询师共同工作的过程，其效果取决于双方配合等多种因素。来访者应向咨询师告知其心理问题的真实情况，包括其他专业机构对其心理问题的评估诊断；积极主动与咨询师一起探索解决问题的方法，完成双方商定的作业。来访者应自行承担未如实告知个人真实情况所造成的不良后果。

（一）来访者有权提出更换咨询师，但在一学期内最多可申请更换一次。

（二）来访者有权终止咨询，但建议在咨询中与咨询师充分讨论后，由双方协商决定。

（三）改期或取消均需至少半天提告知中心办理。

来访者未有明确原因取消或迟到次数累计达到 3 次，须重新预约等候。如来访者迟到，咨询时间不予补足或延长，如来访者迟到时间超过 30 分钟或缺席，本次咨询取消。

四、转介

初始访谈评估后，中心根据来访者情况合理安排后续咨询。如来访者的情况不属于本中心工作范畴，咨询师根据本中心规定予以解释并告知合适的求助资源；如果来访者的咨询议题超出了咨询师的工作能力，咨询师应与来访者协商并妥善安排转介咨询事宜。

五、其他情况

来访者在两次咨询的间隔期，如有紧急情况，请拨打中心电话：0816-6905269（游仙）或 0816-3963291（安州），每周一至周五，上午：9:00-12:00，下午：14:30-17:30，晚上：19:00-21:00。或拨打绵阳市 24 小时公益心理援助热线电话：0816-2424666、0816-2268885。

希望您能在咨询前充分了解上述情况。

我已经阅读并充分理解了以上内容，我愿意在 XX 学院大学生心理健康教育与发展服务中心接受心理咨询服务。

来访者签字：　日期：

咨询师签字：　日期：

Document 18 Original

个体心理咨询知情同意书

亲爱的来访同学：

您好!

心理咨询是心理咨询师与来访者之间建立的一种专业助人关系，其目的是使来访者从咨询师提供的专业服务中获益。本中心希望通过知情同意的过程，明确双方的权利与义务，以保障心理咨询顺利开展。

本中心向本校正式注册的学生提供专业心理咨询服务，不提供精神障碍的诊断、治疗、处方开具、医疗证明开具和心理治疗等服务。

以下是本中心希望您在接受心理咨询之前需要了解的信息：

一、心理咨询设置

（一）心理咨询预约。原则上需要有来访意愿的同学主动提前预约。

（二）心理咨询地点。所有咨询均在本中心指定地点进行。

（三）心理咨询时间。按本中心提供的咨询时间范围内预约，一般情况下每周一次、每次咨询时间为 50 分钟。

二、咨询师的责任与义务

（一）保密

凡是能接触到来访者的个人资料和会谈信息的咨询师及中心工作人员，都必须严格遵守保密原则。为保障服务质量，在隐去可识别身份的信息后，来访者的案例信息可能被用于本中心研讨和督导，所有参与人会承诺保密；所有案例相关信息运用都需遵守专业伦理规范。

（二）保密例外

1. 来访者有伤害自身或伤害他人的严重危险时；
2. 不具备完全民事行为能力的未成年人等受到性侵犯或虐待时；
3. 法律规定需要披露时。

（三）录音录像

无特别约定，咨询师和来访者皆不得对心理咨询会谈进行录音录像。为保障服务质量，提升专业胜任力，咨询师如需录音录像需要和来访者另签知情同意协议。

（四）迟到与缺席

咨访双方均需遵守咨询设置。如咨询师未提前告知中心的迟到或缺席，应为来访者补足咨询时间。

三、来访者的权利与义务

心理咨询是来访者与咨询师共同工作的过程，其效果取决于双方配合等多种因素。来访者应向咨询师告知其心理问题的真实情况，包括其他专业机构对其心理问题的评估诊断；积极主动与咨询师一起探索解决问题的方法，完成双方商定的作业。来访者应自行承担未如实告知个人真实情况所造成的不良后果。

（一）来访者有权提出更换咨询师。

（二）来访者有权终止咨询，但建议在咨询中与咨询师充分讨论后，由双方协商决定。

（三）改期或取消均需提前告知中心办理。

如来访者迟到，咨询时间不予补足或延长，如来访者迟到时间超过 10 分钟或缺席，本次咨询取消。

四、转介

如来访者的情况不属于本中心工作范畴，咨询师根据本中心规定予以解释并告知合适的求助资源；如果来访者的咨询议题超出了咨询师的工作能力，咨询师应与来访者协商并妥善安排转介咨询事宜。

五、费用

本中心对在校学生提供免费的心理咨询服务。

六、其他情况

来访者在两次咨询的间隔期，如遇到特殊情况，工作时间可以拨打办公电话（0471）4305710；非工作时间紧急情况可以拨打中心 24 小时心理援助热线（0471）4306364。

希望您能在咨询前充分了解上述情况。

我已经阅读并充分理解了以上内容。我愿意在 XX 大学大学生心理辅导与服务中心接受心理咨询服务。

Document 19 Original

编号：____________

心理咨询知情同意书

亲爱的来访同学:

您好!

心理咨询是心理咨询师与来访者之间建立的一种专业助人关系，其目的是使来访者从咨询师提供的专业服务中获益。山东农业工程学院大学生心理健康教育中心（以下简称：本中心）希望通过知情同意的过程，明确双方的权利与义务，以保障心理咨询顺利开展。

本中心向本校正式注册的学生提供专业心理咨询服务，不提供精神障碍的诊断、治疗、处方开具、医疗证明开具和心理治疗等服务。

以下是本中心希望您在接受心理咨询之前需要了解的信息。其中可能有您不理解的术语，您可以向心理咨询教师要求解释任何您不清楚的术语或资料。

一、心理咨询设置

（一）心理咨询预约。原则上需要有来访意愿的同学主动提前预约。

（二）心理咨询地点。所有咨询均在本中心指定地点进行。

（三）心理咨询时间。在本中心提供的咨询时间范围内预约，一般情况下每周一次、每次咨询时间为 50 分钟。

二、咨询师的责任与义务

（一）保密

凡是能接触到来访者的个人资料和会谈信息的咨询师及中心工作人员，都必须严格遵守保密原则。为保障服务质量，在隐去可识别身份的信息后，来访者的案例信息可能被用于本中心研讨和督导，所有参与人会承诺保密；所有案例相关信息运用都需遵守专业伦理规范。

（二）保密例外

1.来访者有伤害自身或伤害他人的严重危险时；

2.不具备完全民事行为能力的未成年人等受到性侵犯或虐待时；

3.法律规定需要披露时。

（三）录音录像

无特别约定，咨询师和来访者皆不得对心理咨询会谈进行录音录像。为保障服务质量，提升专业胜任力，咨询师如需录音录像需要和来访者另签知情同意协议。

（四）迟到与缺席

咨访双方均需遵守咨询设置。如咨询师未提前 24 小时告知中心的迟到或缺席，应为来访者补足咨询时间。

三、来访者的权利与义务

心理咨询是来访者与咨询师共同工作的过程，其效果取决于双方配合等多种因素。来访者应向咨询师告知其心理问题的真实情况，包括其他专业机构对其心理问题的评估诊断；积极主动与咨询师一起探索解决问题的方法，完成双方商定的作业。来访者应自行承担未如实告知个人真实情况所造成的不良后果。

（一）来访者有权提出更换咨询师，但在一学期内最多可申请更换 1 次。

（二）来访者有权终止咨询，但建议在咨询中与咨询师充分讨论后，由双方协商决定。

（三）改期或取消均需至少提前 24 小时告知中心办理。

来访者未有明确原因取消或迟到次数一学期累计达到 3 次，须重新预约等候。如来访者迟到，咨询时间不予补足或延长，如来访者迟到时间超过 30 分钟或缺席，本次咨询取消。

四、转介

初始访谈评估后，中心根据来访者情况合理安排后续咨询。如来访者的情况不属于本中心工作范畴，咨询师根据本中心规定予以解释并告知合适的求助资源；如果来访者的咨询议题超出了咨询师的工作能力，咨询师应与来访者协商并妥善安排转介咨询事宜。

五、费用

本中心对山东农业工程学院正式注册的在校学生提供免费的心理咨询服务。

六、可能的突发情况

被咨询学生在心理咨询过程中，因本身心理状况的复杂性和严重性，有可能出现以下情况：

1.有精神病性症状发作；2.有自杀、自虐或伤害他人、危害社会的行为。

以上情况，心理咨询教师不负任何责任。

七、其他情况

来访者在两次咨询的间隔期，如遇到特殊情况，工作时间可以拨打办公电话 0531-88117870；非工作时间紧急情况可以拨打心理援助热线，如：全国生命危机干预 24 小时热线：4001619995 （1 学生专线，2 抑郁专线，3 生命专线）；济南市医学心理咨询中心热线：0531-85713287/85713289 。

希望您能在咨询前充分了解上述情况。

我已经阅读并充分理解了以上内容，有不明白的地方已经向我的心理咨询教师提出，并获得满意的解释。我愿意在山东农业工程学院大学生心理健康教育中心接受心理咨询服务。

来访者签字：________________ 日期：______年___月___日

咨询师签字：________________ 日期：______年___月___日

Document 20 Original

心理咨询知情同意书

以下是关于心理咨询的一些基本约定，请仔细阅读并在下面签字表明你了解相关规定，并能为自己的行为负责。

1.为了充分尊重和保护来访者以及咨询师的权益，特拟定《知情同意书》。

2.咨询保密原则：保密是心理咨询的工作原则之一，也是职业道德的集中体现。来访者的个人信息及咨询的相关问题不会被随意谈论，来访者的信息登记表不会被带出咨询室之外的任何地方。一般来说，来访者是否接受过咨询以及咨询的内容都不会被透漏给中心以外的非专业人员。**但下述几种情况除外**：（1）来访者出现自我伤害或伤害他人的倾向。（2）来访者的问题涉及法律责任。（3）为了能更好的帮助来访者，咨询师提出个案讨论或申请督导，但仅限专业场合，同时须隐去来访者的个人化信息。

3.为了方便后续的跟踪咨询服务，需要把来访者的真实姓名联系方式及紧急联系人等信息登记在案，这些个人资料只用于心理咨询中的管理，不会透漏给其他任何单位和个人。

4.如果咨询中心或咨询师需要对会谈进行录音或录像时，需征得来访者同意。

5.每次会谈时间一般为 50 分钟。会谈次数由来访者和咨询师协商，双方严格遵守。

6.心理咨询不能代替药物治疗，请来访者严格按照医嘱进行药物使用。

7.心理咨询机构及咨询师，只承担对自杀、精神分裂、抑郁症等危险症状对家属的告知义务，不承担其他责任。对由于来访者及家人隐瞒诊断和症状造成的后果，不承担责任。

8.来访者有权利在任何时候中止咨询。

我已经阅读并理解了上述信息，而且知道我可以询问有关的问题。我同意参加心理咨询。

来访者：　　　　　　咨询师：

年　　月　　日

Document 21 Original

心理辅导与咨询知情同意书
亲爱的来访者:

感谢来访者对本中心的信任,请按照本中心的工作流程,在助理的安排下,完成 预约登记、心理测试等环节。请仔细阅读以下说明并在下面签字表明你了解相关规定, 并且能为自己的行为负责。

一、心理辅导和咨询的目的是提升心理健康水平,尽早发现心理不适并加以预防和干预。

二、进入本中心的求助属自愿行为。咨询过程中应提供真实的相关信息,提供失真或隐瞒重要信息将不利于心理干预的效果,其咨询结果及其它相应后果由来访者本人承担。

三、咨询师与来访者双方在相互了解信任的基础上开展会谈辅导,咨询方案由双方协商确定,来访者有权利选择或更换咨询师,有权利和咨询师谈论任何问题。来访者也有权在必要的时候,主动中止咨询。咨询师亦有权利出于来访者福祉的考虑 提出转介或中止咨询。

四、对来访者在咨询中提供的信息,中心将严格遵守保密原则。若中心工作人员和咨询师团队基于专业工作的需要接触到来访者的档案,均会遵循保密原则。下列为 保密原则中的保密例外情形:

1)来访者有伤害自身或伤害他人的严重危险时;
2)来访者有致命的传染疾病等可能危及他人时;
3)未成年人受到性侵犯或虐待时;
4)法律规定需要披露时:
5)来访者的问题超出心理咨询的服务范围时。

中心以专业科学的方式与来访者的亲属或辅导员联系,以尽到最大限度为来访 者寻求帮助资源的责任。

Document 22 Original

心理咨询知情同意书

根据《中华人民共和国心理咨询师职业标准》以及服务行业的通用法规规定，沈阳医学院大学生心理健康教育中心 心理咨询中心（以下简称心理中心）与来访学生本着平等自愿协商一致原则，就心理中心咨询师为来访学生提供心理咨询进行协商，达成如下协议：

一、保密原则

心理中心须对来访学生的咨询内容严格保密，未经来访学生同意不能泄露给第三方。以下情况例外：

来访者有伤害自身或他人的严重危险时；

来访者有致命的传染性疾病等且可能危及他人时；

未成年人在受到性侵犯或虐待时；

法律规定需要披露时。

经心理中心咨询师观察，认为来访学生有可能出现行为失控，并影响校园安全与教学秩序时，属于保密例外情况，心理中心咨询师有权利突破保密协议通知来访学生辅导员或紧急联系人。

心理咨询师为了更有效地协助来访学生解决问题，或为接受专业督导或教学与科研的需要，可能要求录音（像），但在录音（像）之前，必须征求来访学生的意见，在征得来访学生的书面同意后方可录音（像）， 来访学生有同意或拒绝的权利。

二、咨询时间

每次咨询时间为 45 分钟，请勿迟到或缺席。如需请假或取消咨询，应至少提前一天告知心理中心，且每人每学期咨询请假次数不超过 2 次；如未提前告知，将视作无故失约，无故失约 1 次，心理中心将取消后续咨询安排。

由于心理议题的复杂性，咨询很难一次完成，具体咨询次数可由来访学生与咨询师商议后决定；来访学生应在咨询结束前与咨询师商定咨询时间后再离开。

因咨询资源有限，每人每学期常规咨询次数（含请假）原则上不超过 8 次。

三、咨询终止

达成咨询目标后，终止咨询。

若来访学生或心理咨询师感觉咨询效果不佳，可协商转介事宜。

来访学生有权接受或停止咨询，也有权决定是否参与咨询过程中的有关活动。

四、咨询过程

来访学生在咨询时，有义务提供咨询师因咨询所需的真实个人资料，以保证更好的咨询效果。

来访学生须保证在接受心理咨询期间不发生任何故意伤害自己或故意危害他人人身安全的行为。

有些不属于心理咨询范围的患者，为配合其他精神科的药物治疗，在其本人有能力接受心理咨询的情况下，如果家属或者本人希望进行心理咨询的，咨询师本人也愿意为其咨询的，可以进行心理咨询。

心理咨询师与来访学生之间不得产生和建立咨询以外的任何关系，原则上咨询师不和来访学生做咨询外沟通（不互相留微信、QQ、手机号等），如需确定咨询时间、改变时间、临时需求增加咨询议题应及时拨打 62216906 沟通。

五、其他说明

心理中心为沈阳医学院全体学生提供个人成长和自我发展类相关问题的免费心理咨询，

不提供精神鉴定、心理诊断、开具精神类药物、出具资格证明等服务内容。

未尽事宜，　双方协商后补充如下：

心理咨询师：

来访者：　电话：

紧急联系人：　电话：

年 月 日

Document 23 Original

知情同意书
亲爱的同学，你好：
亲爱的来访者：
感谢您的信任。
心理咨询是咨询师与来访者之间建立的一种专业助人关系，其目的是使来访者从咨询师提供的专业服务中获益。为了维护您和咨询师的权益，保证心理咨询的顺利进行，在预约开始之前，请仔细阅读下列事项并确认。
1、本中心向本校全日制在读学生（不含休学中的学生）提供免费的心理咨询服务，不提供精神障碍的诊断、治疗、处方开具、医疗证明开具和心理治疗等服务。
2、咨询地点为三个校区的心理咨询室。一般情况下每周 1 次、每次咨询时间为 50 分钟。
3、为尽可能保证大多数同学权益，原则上每位来访者在一学期内最多可接受 8 次咨询，且不能预约同一天的多个时段。如有特殊情况，须经评估后才可继续咨询。
4、来访者应按预约时间提前十分钟到达咨询室。
如不能如期来访，应提前在 APP 上取消预约。如来访者迟到，咨询时间不予补足或延长，如来访者迟到时间超过 30 分钟或缺席，本次咨询取消且计入 8 次咨询之中。如来访者一学期内未有明确原因取消或迟到次数达到 3 次，将暂停本学期预约资格。
5、来访者有权提出更换咨询师，但在一学期内最多可更换一次咨询师。如因咨询师未结案无法预约新的咨询师，请拨打 85463928 告知情况。
6、咨询师如因个人原因需取消或更改已预约的咨询时间，需提前告知来访者并约定新的咨询时间。
7、咨询师尊重和保护来访者的个人隐私，咨询相关个人信息都在严格保密的情况下进行保存，不会被泄露。为更好地帮助来访者，在隐去可识别身份的信息后，来访者的案例信息可能被用于本中心研讨和督导，所有参与人会承诺保密，且所有案例相关信息的运用都需遵守专业伦理规范。咨询过程中，来访者未经同意不可进行录音录像。
8、如来访者的行为可能对自己或他人构成严重伤害，或出于司法原因作为呈堂证供，则咨询师可以突破保密原则，向有关单位报告来访者的情况。除此之外，咨询记录将妥善保存在本中心，不提供给包括来访者在内的任何机构与个人。
9、如来访者的情况不属于本中心工作范畴，咨询师会予以解释并告知合适的求助资源。
10、如在咨询后希望更改咨询师，但系统显示无法预约新的咨询师，请联系 85463928，我们会手动进行处理。
11、如没有可预约时段，可以先拨打 85401212 进行电话咨询。
以上全部规定，本人已经阅读，并愿意遵守！
姓名：

Document 24 Original

学生心理健康指导中心知情同意书

心理咨询知情同意书

同学您好，欢迎来学校心理中心进行心理咨询。

心理咨询是心理咨询师与来访者之间建立的一种专业助人关系，其目的是使来访者从咨询师提供的专业服务中获益。本中心向本校学籍的学生提供专业心理咨询服务，不提供精神障碍的诊断、治疗、处方开具、医疗证明开具和心理治疗等服务。

为维护您的权利，保障心理咨询顺利有效开展，本中心希望通过知情同意的过程，使您明确该工作相关内容。因此，请您认真阅读以下内容，如无异议，请在最后签字。

以下是本中心希望您在接受心理咨询之前需要了解的信息：

一、心理咨询设置

（一）心理咨询预约。原则上需要有咨询意愿的同学亲自主动提前预约。若他人代为预约，来访同学未明确否定，则视为本人同意预约。

（二）心理咨询地点。所有咨询均在本中心指定地点进行。

（三）心理咨询时间。按本中心提供的咨询时间进行咨询。首次后的咨询时间，由咨询师与来访学生协商确定。一般情况下每周一次，每次咨询时长为 50 分钟。每位学生在一学期内最多可接受 10 次咨询，特殊情况需与咨询师协商处理。

（四）心理咨询中的迟到与缺席。咨访双方均需遵守咨询设置。改期或取消均需至少提前 24 小时告知中心办理（紧急情况除外）。咨询师在未提前告知的情况下迟到或缺席，应为来访者补足咨询次数与时长。来访者迟到，咨询时长不予补足或延时；来访者迟到时间超过 30 分钟或缺席，本次咨询取消。来访者在未结案咨询期间没有明确原因取消或迟到次数累计达到 3 次，须重新预约等候。

二、中心的责任与义务

（一）保密

凡是能接触到来访者的个人资料和会谈信息的咨询师及中心工作人员，都必须严格遵守保密原则。为保障服务质量，在隐去可识别身份的信息后，来访者的案例信息可能被用于本中心研讨和督导，所有参与人会承诺保密；所有案例相关信息运用都需遵守专业伦理规范。

（二）保密例外

1.来访者有伤害自身或伤害他人的严重危险时；

2.不具备完全民事行为能力的未成年人等受到性侵犯或虐待时；

3.法律规定需要披露时。

（三）录音录像

无特别约定，咨询师和来访者皆不得对心理咨询会谈进行录音录像。为保障服务质量，提升专业胜任力，咨询师如需录音录像需要和来访者另签知情同意协议。

三、来访者的权利与义务

心理咨询是来访者与咨询师共同工作的过程，其效果取决于双方配合等多种因素。

（一）来访者应向咨询师告知其心理问题的真实情况，包括其他专业机构对其心理问题的评估诊断，否则应自行承担由此引发的不良后果。

（二）积极主动与咨询师一起探索解决问题的方法，完成双方商定的作业。

（三）来访者有权提出更换咨询师，但在一学期内最多可申请更换一次。

（四）来访者有权终止咨询，但建议在咨询中与咨询师充分讨论后，由双方协商决定。

四、转介

咨询师对来访者进行评估后，如来访者的情况不属于本中心工作范畴，咨询师根据本中心规定予以解释并告知合适的求助资源。如果来访者的咨询议题超出了咨询师的工作能力，咨询师应与来访者协商并妥善安排转介咨询事宜。

五、费用

本中心为在校学生提供免费的心理咨询服务。

六、其他情况

来访者在两次咨询的间隔期，如遇到特殊情况，工作时间可以拨打办公电话：0351-3176212（明向校区），0351-6010260（迎西校区）；非工作时间紧急情况可以拨打心理援助热线，热线号码为：0351-4639459（山西医科大学第一医院 24 小时心理热线）、4006-525-521（16:30—22:30）（清华大学珍惜生命大学生心理热线）。

我已经阅读并充分理解以上内容，愿意在 XX 大学心理中心接受心理咨询服务

来访者签字:　　　　咨询师签字:　　　日期:

（此文件一式两份，一份交予来访同学，一份心理中心留存）

Document 25 Original

心理学部心理咨询研究与培训中心

心理咨询知情同意书

欢迎您来到 XX 大学心理学部心理咨询研究与培训中心接受心理咨询服务。本心理咨询服务由受到专业系统培训的**应用心理临床与咨询方向专业硕士**作为实习咨询师免费提供。为保证咨询更加科学、规范、有效，现将咨询的相关信息做出如下说明:

1、保密原则

本咨询室郑重承诺，遵守保密原则，未经您同意，不会向第三方透露您的个人信息，您的咨询情况不会进入学籍档案和职员档案，不影响学业成绩和社交关系。咨询师将您的案例与督导讨论时，会隐去您的个人信息。但发生以下情况我们不会保密：（1）当您自己的生命受到威胁（如自伤、自残、自杀），或因您而导致他人的生命安全受到威胁时；（2）涉及相关法律法规问题时；（3）如果您是未成年人、患有严重精神障碍或丧失民事行为能力，我们会与监护人、家属等相关人员进行沟通。以上保密原则的突破都是为了给您提供更加专业有效的服务。

2、关于咨询安排

1) **时间**：一般情况下每周一次，每人每次咨询时间约为 50 分钟，涉及婚姻家庭等多成员的问题，或者某些咨询方法的需要，时间会有所延长，但原则上不超过 90 分钟。每次咨询请至少提前 10 分钟到达咨询室或线上平台。如果您在未取消预约时间的情况下迟到，咨询师不相应延长咨询时间。

2) **取消**：如果您无法参加预约好的咨询,请您提前 24 小时通知我们,以便安排等待咨询的人。如果咨询师遇出差、开会等无法参加咨询,我们会提前通知您。

3) **测试**：根据您的情况,可能会进行一些心理测试，有助于咨询师对您情况的评估。

4) **录音录像**：由于咨询过程均在本中心专家咨询师的督导下进行，所以咨询师将进行录音或录像，以便于督导时进行讨论。录音录像等将严格遵守保密原则。

5) **督导**：督导将严格遵守保密原则，您的个人信息仅限于咨询师、督导师、团体督导小组可以知晓，并且了解您信息的各方将签署保密协议，并接受该协议的约束。

6) **本校学生**：如果您是西南大学学生，将通过西南大学大学生心理健康教育与服务中心进行预约报备，如果涉及到危机状况（自身或他人的生命安全受到威胁、严重精神障碍等），我们会将情况告知大学生心理健康教育与服务中心。

3、转介原则

1) 若您的问题涉及严重心理问题,我们会为您推荐更适合的治疗师或治疗机构,进行转介。

2) 若咨询师无法给您提供较好心理咨询时,与您协商后可向其他咨询师转介。

来访者确认：来访者已阅读、同意以上所有内容,自愿接受心理咨询。

来访者签名：________________

时间：　　年　　月　　日

XX 大学心理学部心理咨询研究与培训中心

Document 26 Original

心理咨询知情同意书

为切实贯彻“以人为本，助人自助”的原则，维护来访者和咨询师的利益，保证心理咨询的顺利进行，就心理咨询基本原则、来访者和咨询师的责任和义务做如下说明：

一、咨询说明

1. 咨询保密原则：保密是心理咨询的工作原则之一，也是职业道德的集中体现。来访者的个人信息及咨询的相关问题不会被随意谈论，来访者的信息登记表不会被带出咨询室之外的任何地方。一般来说，来访者是否接受过咨询以及咨询的内容都不会被透漏给中心以外的非专业人员。

但下述几种情况除外：

（1）来访者出现自我伤害或伤害他人的倾向。

（2）来访者的问题涉及法律责任。

（3）为了能更好的帮助来访者，咨询师提出个案讨论或申请督导，但仅限专业场合，同时须隐去来访者的个人化信息。

2. 为了方便后续的跟踪咨询服务，需要把来访者的真实姓名联系方式及紧急联系人等信息登记在案，这些个人资料只用于心理咨询中的管理，不会透漏给其他任何单位和个人。

3. 如果咨询中心或咨询师需要对会谈进行录音或录像时，需征得来访者同意。

4. 每次会谈时间一般为 50 分钟。会谈次数由来访者和咨询师协商，双方严格遵守。

5.心理咨询不能代替药物治疗，请来访者严格按照医嘱进行药物使用。对由于来访者及家人隐瞒诊断和症状造成的后果，不承担法律责任。

6.心理咨询机构及咨询师，对自杀观念、精神分裂、抑郁症等危险症状可能造成的风险和后果有对告知的义务，不承担法律或其他责任。如果您在此前有过自杀冲动或者自杀行为，并且是带着这样的困扰寻求帮助的，我们会尽所能提供帮助，但不能保证咨询期间您的冲动马上减缓，也不能保证您今后不会再有自杀冲动和行为。请您在有自杀冲动和行为之前告知心理咨询师和我们，我们一起探讨更有建设性的问题解决之道。但如果您最终选择自杀，您做出了决定，您将承担全部责任和个人行为后果。

二、来访者的权利和义务

1. 以积极的态度对待咨询，坦诚地向咨询师表露自己，不掩饰不伪装。

2. 自愿的原则。来访者有权决定中止或结束咨询，可以自主选择咨询师，与咨询师协商修正咨询的方向及方法。

3. 自主。努力实现自我成长，为咨询负责，不期待咨询师帮你做决定。

Document 27 Original

心理咨询知情同意书

为保护咨询教师与来访学生（简称“咨访”）双方的权益，明确双方的权利、义务，保证心理咨询的有效性和专业性，来访学生须仔细阅读和了解以下内容。

一、来访学生的义务与权利

1.咨询规范

来访学生应准时赴约，如迟到或取消预约，应告知心理中心；每次咨询约 50 分钟，两次咨询间隔宜超过 1 周；请出示个人有效证件。

2.坦诚沟通

来访学生应当如实填写个人信息，坦诚表达真实感受，并且承担隐瞒、掩饰等带来的不良影响。

3.咨询认知

来访学生应合理看待心理咨询。来访学生积极的互动、参与和体验是成长的关键，咨询教师的角色是倾听者、陪伴者、助人者，而不是决策者。咨询是一个渐进的过程，一次咨询不能解决所有问题。心理测验是一种辅助性工具，目的是进一步了解学生具体状况，不具有诊断意义。

4.来访学生权利

来访学生有权更换咨询教师、终止心理咨询，并承担可能给咨询带来的不良影响。

二、咨询教师的义务与权利

1.严格保密

咨询教师应当保护学生的隐私权，严格为学生的个人信息和叙述内容保守秘密。如需在科研、教学或督导等活动中采用咨询案例，应隐去可能辨认出的来访学生相关信息。

2.保密例外

心理咨询的保密原则是有限度的，保密原则例外的情况如下：（1）来访学生有伤害自身或他人的危险；（2）来访学生有疑似心理障碍；（3）来访学生有 A 类、B 类等危及生命健康的传染性疾病；（4）来访学生近期遭受严重侵害；（5）法律规定需要披露的其他情况。

3.专业规范

咨询教师应耐心倾听、真诚帮助来访学生。

4.咨询范围

咨询教师仅提供心理咨询服务，不提供精神障碍的诊疗和心理治疗等服务。

5.咨询教师权利

在充分考虑的情况下，本着对来访学生负责的态度，咨询教师可以转介或终止咨询。

三、咨访双方共同遵守的约定

1.咨询目标

咨访双方通过商讨，共同确定具体、可行、积极、具备心理学性质的咨询目标。

2.避免多重关系

除咨询关系外，咨访双方应避免多重关系，比如经济关系、商业关系等。

3.联系方式

咨访双方宜通过办公电话联系。手机、微信等不能保证及时回复。

以上全部内容，本人已阅读，并愿意遵守！

来访学生：

年 月 日

XX 大学学生工作处心理健康教育服务中心

Document 28 Original

个体心理咨询知情同意书
亲爱的来访者:
为了充分尊重和保护您以及咨询师的权益， 规范心理咨询过程，保证心理咨询有效进行， 特拟定本《个体心理咨询知情同意书》。请仔细阅读下文，并在相应位置签上姓名，以表明仔细阅读过，且同意这些条款。
1.心理健康教育中心免费为全校学生提供心理咨询服务。每次咨询时间 50 分钟，每周咨询 1 次，每学期咨询限 8 次。咨询时间: ________________________，咨询地点: ____________________________。
2.来访者应在预约时间准时到达心理咨询室，如需要改变或取消咨询，请提前一个工作日通知中心，如无故连续两次未参加咨询，将取消全部咨询。来访者如果迟到，将不相应延长咨询时间，仍在约定时间结束咨询。
3.来访者有权在任何时候终止咨询，但我们建议在正式退出咨询之前与您的咨询师讨论您的想法。
4.咨询师会对您在咨询中提供的信息严格保密，如确因学术、督导或其他需要，将隐去可能辨认出来访者的个人信息。 如来访者的行为可能对自己或他人构成伤害，或出于司法原因，咨询师可以突破保密原则。
5.心理咨询仅提供涉及学习生活、个人成长和自我发展相关问题的心理测评和咨询，不提供包括精神鉴定、诊断、处方精神类药物、出具资格证明、对严重精神障碍患者的长程心理治疗等在内的服务内容。如来访者可能患有某种精神障碍，应到相关专科医院就诊治疗。
6.若您来访前曾有过专业医疗机构作出的精神疾病相关诊断，您有义务提供诊断书，不能隐瞒精神类相关病史。对于您隐瞒诊断和症状造成的后果，中心和咨询师不承担责任。心理咨询不能代替药物治疗，请严格按照医嘱进行药物使用。
7.如果咨询任一方需要对会谈进行录音或录像，需先征得对方的同意。
8.咨询师不得接受您的礼物，且不在咨询之外与您进行咨询性质的交流。
9.来访者在两次咨询的间隔期，如遇特殊情况，工作时间可拨打办公电话: 85250525; 非工作时间紧急情况可拨打长春市心理援助热线: 12320,0431-89685000,0431-89685333。也可拨打全国心理援助热线: 800-810-1117（仅限固定电话拨打）;010-82951332（固定电话、 移动电话均可拨打）。
以上全部条款本人已经阅读， 并愿意遵守!
来访者签名:　　　　　　　日期:
咨询师签名:　　　　　　　日期:

Document 29 Original

心理咨询知情同意书

亲爱的来访同学：

您好！

心理咨询是心理咨询师与来访者之间建立的一种专业助人关系，其目的是使来访者从咨询师提供的专业服务中获益。本中心希望通过知情同意的过程，明确双方的权利与义务，以保障心理咨询顺利开展。

本中心向本校正式注册的学生提供专业心理咨询服务，不提供精神障碍的诊断、治疗、处方开具、医疗证明开具和心理治疗等服务。

以下是本中心希望您在接受心理咨询之前需要了解的信息：

一、心理咨询设置

（一）心理咨询预约。原则上需要有来访意愿的同学主动提前预约。

（二）心理咨询地点。所有咨询均在本中心指定地点进行。

（三）心理咨询时间。按本中心提供的咨询时间范围内预约，一般情况下每周一次、每次咨询时间为 50 分钟。原则上每位学生在一学期内最多可接受 8 次咨询。

二、咨询师的责任与义务

（一）保密

凡是能接触到来访者的个人资料和会谈信息的咨询师及中心工作人员，都必须严格遵守保密原则。为保障服务质量，在隐去可识别身份的信息后，来访者的案例信息可能被用于本中心研讨和督导，所有参与人会承诺保密；所有案例相关信息运用都需遵守专业伦理规范。

（二）保密例外

1. 来访者有伤害自身或伤害他人的严重危险时；
2. 不具备完全民事行为能力的未成年人等受到性侵犯或虐待时；
3. 法律规定需要披露时。

（三）录音录像

无特别约定，咨询师和来访者皆不得对心理咨询会谈进行录音录像。为保障服务质量，提升专业胜任力，咨询师如需录音录像需要和来访者另签知情同意协议。

（四）迟到与缺席

咨访双方均需遵守咨询设置。如咨询师未提前 24 小时通过中心告知来访学生的迟到或缺席，应为来访者补足咨询时间。

三、来访者的权利与义务

心理咨询是来访者与咨询师共同工作的过程，其效果取决于双方配合等多种因素。来访者应向咨询师告知其心理问题的真实情况，包括其他专业机构对其心理问题的评估诊断；积极主动与咨询师一起探索解决问题的方法，完成双方商定的作业。来访者应自行承担未如实告知个人真实情况所造成的不良后果。

（一）来访者有权提出更换咨询师，但在一学期内最多可申请更换一次。

（二）来访者有权终止咨询，但建议在咨询中与咨询师充分讨论后，由双方协商决定。

（三）改期或取消均需至少提前 24 小时告知中心办理。

来访者未有明确原因取消或迟到次数一学期累计达到 2 次，须重新预约等候。如来访者迟到，咨询时间不予补足或延长，如来访者迟到时间超过 20 分钟或缺席，本次咨询取消。

四、转介

初始访谈评估后，中心根据来访者情况合理安排后续心理服务工作。如来访者的情况不属于本中心工作范畴，咨询师根据本中心规定予以解释并告知合适的求助资源；如果来访者的咨询议题超出了咨询师的工作能力，咨询师应与来访者协商并妥善安排转介咨询事宜。

五、费用

本中心对在校学生提供免费的心理咨询服务。

六、其他情况

来访者在两次咨询的间隔期，如遇到特殊情况，工作时间可以拨打办公电话86608122/86601122；非工作时间紧急情况可以拨打当地适用的心理援助热线，热线号码为：【白班】0371-86169595、【晚班】0371-22993442，并及时和辅导员沟通。

本协议由 XX 大学心理健康教育中心代为保存。

希望您能在咨询前充分了解上述情况。

我已经阅读并充分理解了以上内容。我愿意在 XX 大学心理健康教育中心接受心理咨询服务。

Document 30 Original

来访者须知

亲爱的同学:

你好，欢迎你接受重庆第二师范学院心理健康教育与咨询中心提供的心理咨询服务，谢谢你的信任！在正式开始咨询之前，请仔细阅读以下内容:

一、心理咨询的服务对象为全体在校学生。

二、心理咨询的服务内容主要为有心理困扰的来访者进行鉴别、疏导、调整。

三、心理咨询遵循以下原则:

（一）重庆第二师范学院学生心理健康教育与咨询中心是组织实施全校心理健康教育和心理咨询工作的专门机构，我们的服务理念是尊重、平等、真诚、保密。

（二）心理问题与困扰的解决通常需要一段时间，一般情况下心理咨询不可能一次就会取得明显的效果或者彻底解决问题，而是一个循序渐进的过程，请您要有耐心和信心。

（三）平等。您有权力询问有关心理咨询老师、服务内容、咨询时间、服务方式等方面的情况，以便根据自己具体情况选择咨询老师、方式及时间，如对咨询进程不满意可随时中断咨询。

（四）保密。心理咨询将严格遵守保密原则，对来访者与咨询师的谈话及相应资料保密。但遇特殊情况如来访者有伤害他人或伤害自己危险或来访者的情况需要转介时，心理咨询师将突破本原则。

（五）自主。心理咨询的理念是“助人自助”，只有您自己才能真正解决您的问题，不能过分依赖心理咨询师替您做决定。

（六）坦诚。在咨询室请坦城地表露自己，为了达到更好的咨询效果，来访者应及时把内心真正的困惑或咨询过程中产生的问题与心理咨询师沟通。

（七）自愿。是否开始或终止接受心理咨询都由来访者本人决定，老师只能提出建议，无权强硬要求。相应地，随意终止咨询由此带来的不良影响由来访者本人承担。咨询过程中，若对咨询方向或方法有异议，可与心理咨询师进行必要的讨论并修正。

四、咨询设置

心理咨询采取预约制，每次咨询时间为 40-60 分钟，一次咨询不能完成者，可在面谈结束后再约下一次咨询时间。如无法按期咨询，请提前 24 小时联系中心说明情况，并确定新的咨询时间。

我已认真阅读并理解了《来访者须知》,同意并接受以上内容。

签名:

日期:　　　年　　月　　日

Document 31 Original

心理咨询知情同意书

亲爱的来访同学：

您好！心理咨询是心理咨询师与来访者之间建立的一种专业助人关系，其目的是使来访者从咨询师提供的专业服务中获益。本中心希望通过知情同意的过程，明确双方的权利与义务，以保障心理咨询顺利开展。

本中心向本校正式注册的学生提供专业心理咨询服务，不提供精神障碍的诊断、治疗、处方开具、医疗证明开具和心理治疗等服务。

以下是本中心希望您在接受心理咨询之前需要了解的信息：

一、心理咨询设置

（一）心理咨询预约。原则上需要有来访意愿的同学主动提前预约。

（二）心理咨询地点。花溪校区：学生活动中心 3 楼大学生心理健康与成才服务中心；

两江校区：学生公寓 2 栋 112-114 室。

（三）心理咨询时间。按本中心提供的咨询时间范围内预约，一般情况下每周一次、每次咨询时间为 50 分钟。原则上每位学生在一学年内最多可接受 8 次咨询。

二、咨询师的责任与义务

（一）保密

凡是能接触到来访者的个人资料和会谈信息的咨询师及中心工作人员，都必须严格遵守保密原则。为保障服务质量，在隐去可识别身份的信息后，来访者的案例信息可能被用于本中心研讨和督导，所有参与人会承诺保密；所有案例相关信息运用都需遵守专业伦理规范。

（二）保密例外

1.来访者有伤害自身或伤害他人的危险时；咨询师必须采取必要的措施，防止意外发生，必要时通知有关部门或家属，或与其他心理咨询师磋商，作为学校的心理咨询师需要按照《重庆理工大学心理危机干预流程》处理。

2.不具备完全民事行为能力的未成年人等受到性侵犯或虐待时。

3.当受卫生、司法或有关公安机关询问时，法律规定需告知的情况。

（三）录音录像

无特别约定，咨询师和来访者皆不得对心理咨询会谈进行录音录像。为保障服务质量，提升专业胜任力，咨询师如需录音录像需要和来访者另签知情同意协议。

（四）迟到与缺席

咨访双方均需遵守咨询设置。如咨询师未提前 24 小时告知中心的迟到或缺席，应为来访者补足咨询时间。

三、来访者的权利与义务

心理咨询是来访者与咨询师共同工作的过程，其效果取决于双方配合等多种因素。来访者应向咨询师告知其心理问题的真实情况，包括其他专业机构对其心理问题的评估诊断；积极主动与咨询师一起探索解决问题的方法，完成双方商定的作业。来访者应自行承担未如实告知个人真实情况所造成的不良后果。

（一）来访者有权提出更换咨询师，但在一学期内最多可申请更换一次。

（二）来访者有权终止咨询，但建议在咨询中与咨询师充分讨论后，由双方协商决定。

（三）改期或取消均需至少提前 24 小时告知中心办理。

来访者未有明确原因取消或迟到次数一学期累计达到 3 次，须重新预约等候。如来访者迟到，咨询时间不予补足或延长，如来访者迟到时间超过 30 分钟或缺席，本次咨询取

消。

四、转介

初始访谈评估后，中心根据来访者情况合理安排后续咨询。如来访者的情况不属于本中心工作范畴，咨询师根据本中心规定予以解释并告知合适的求助资源；如果来访者的咨询议题超出了咨询师的工作能力，咨询师应与来访者协商并妥善安排转介咨询事宜。在和其他治疗机构接触的过程中产生任何的纠纷或其他的事件属于来访者个人行为范畴。

五、费用

本中心对在校学生提供免费的心理咨询服务。

六、其他情况

来访者需要联系中心对咨询进行调整或遇特殊情况，工作时间（上午 10:00-12:00，下午 14:00-17:00，晚上 19:00-22:00）可以拨打 62563208； 非工作时间紧急情况可以拨打重庆市 12320-1 心理援助热线，热线号码为：023-12320-1。

七、紧急联系人

来访者需要提供一位紧急联系人信息，并保证信息的真实、准确、有效性。在紧急情况下（如来访者存在危及自身或他人生命的情况等），心理中心可能与该联系人取得联系，通报来访者的状况，但不会将来访者的隐私内容透露给该联系人。如果来访者提供的联系人信息为无效信息，由此导致的一切后果将有来访者本人承担，与咨询师、心理中心及学校无关。

我已读过，也同意以上所注明的，自愿参加咨询，并能为自己的行为负责。

Document 32 Original

咨询知情同意书

根据《中华人民共和国精神卫生法》、《中国心理学会临床与咨询心理学工作伦理守则(第二版)》中“善行、责任、诚信、公正、尊重”的总则，为保障来访者和咨询师的权益，并籍此保证和提升重庆师范大学学生心理健康教育与咨询中心的心理咨询专业服务的水准，现就有关咨询事项协议如下：

心理咨询是指在良好的咨询关系基础上，由经过专业训练并拥有相关资质的咨询师运用咨询心理学的有关理论和技术，对有心理困扰的来访者进行帮助，以消除或缓解来访者的心理困扰，促进其心理健康与自我发展的过程。

本中心尊重来访者的个人隐私，将严格按照《中国心理学会临床与咨询心理学工作伦理守则（第二版)》遵守“保密原则”。另外，为提高咨询水平，保障来访者接受高质量的咨询服务，有时需在咨询过程中录音或录像，用于个案督导、个案研讨或教学研究。如有录音、录像的需求，本中心会事前征得来访者的书面同意。但下列情况为保密原则的例外，本中心有权与有关方面联系：

（1）咨询师发现寻求专业服务者有伤害自身或伤害他人的严重危险；

（2）未成年人等不具备完全民事行为能力的人受到性侵犯或虐待；

（3）法律规定需要披露的其他情况。

来访者首次来访，请务必携带本人学生证，并按预约时间准时到达，若不能如期到达应提前打电话说明。本中心咨询服务不收取任何费用，但来访者应保持积极、开放和诚实的态度，向着双方共同制定的咨询目标而努力，并配合中心规定填写相关文件。在咨询过程中，来访者有权随时提出中断咨询或更换咨询师，中心将及时安排新的咨询师或帮助来访者寻求合适的转介服务。来访者如需续约，须与本中心预约确认。

咨询过程须在咨询室内完成，来访者不得要求在咨询室以外进行心理咨询。在等待咨询的

过程中应保持安静；爱护咨询室的公共设施。

我已知晓并同意以上内容。

来访者签名：

日期：

_年_月_日

Document 33 Original

中国心理学会临床心理学注册工作委员会制

高校心理咨询知情同意书

亲爱的来访同学：您好！

心理咨询是心理咨询师与来访者之间建立的一种专业助人关系，其目的是使来访者从咨询师提供的专业服务中获益。本中心希望通过知情同意的过程，明确双方的权利与义务，以障心理咨询顺利开展。

本中心向本校正式注册的学生提供专业心理咨询服务，不提供精神障碍的诊断、治疗、处方开具、医疗证明开具和心理治疗等服务。

以下是本中心希望您在接受心理咨询之前需要了解的信息：

一、心理咨询设置

（一）心理咨询预约。原则上需要有来访意愿的同学主动提前预约。

（二）心理咨询地点。所有咨询均在本中心指定地点进行。

（三）心理咨询时间。按本中心提供的咨询时间范围内预约，一般情况下每周一次、每次咨询时间为 50 分钟。原则上每位学生在一学期（选用）内最多可接受 8 次（选用）咨询。

二、咨询师的责任与义务

（一）保密 凡是能接触到来访者的个人资料和会谈信息的咨询师及中心工作人员，都必须严格遵守保密原则。为保障服务质量，在隐去可识别身份的信息后，来访者的案例信息可能被用于本中心研讨和督导，所有参与人会承诺保密；所有案例相关信息运用都需遵守专业伦理规范。

（二）保密例外 1. 来访者有伤害自身或伤害他人的严重危险时； 2. 不具备完全民事行为能力的未成年人等受到性侵犯或虐待时； 3. 法律规定需要披露时。 （三）录音录像

无特别约定，咨询师和来访者皆不得对心理咨询会谈进行录音录像。为保障服务质量，提升专业胜任力，咨询师如需录音录像需要和来访者另签知情同意协议。

（四）迟到与缺席 咨访双方均需遵守咨询设置。如咨询师未提前 24 小时（选用）告知中心的迟到或缺席， 应为来访者补足咨询时间。

三、来访者的权利与义务

心理咨询是来访者与咨询师共同工作的过程，其效果取决于双方配合等多种因素。来访者应向咨询师告知其心理问题的真实情况，包括其他专业机构对其心理问题的评估诊断；积极主动与咨询师一起探索解决问题的方法，完成双方商定的作业。来访者应自行承担未如实 告知个人真实情况所造成的不良后果。

（一）来访者有权提出更换咨询师，但在一学期（选用）内最多可申请更换一次。

（二）来访者有权终止咨询，但建议在咨询中与咨询师充分讨论后，由双方协商决定。

（三）改期或取消均需至少提前 24 小时（选用）告知中心办理。 来访者未有明确原因取消或迟到次数一学期（选用）累计达到 3 次（选用），须重 新预约等候。如来访者迟到，咨询时间不予补足或延长，如来访者迟到时间超过 30 分钟 （选用）或缺席，本次咨询取消。

四、转介

初始访谈评估后，中心根据来访者情况合理安排后续咨询（选用）。如来访者的情况不属于本中心工作范畴，咨询师根据本中心规定予以解释并告知合适的求助资源；如果来访者的咨询议题超出了咨询师的工作能力，咨询师应与来访者协商并妥善安排转介咨询

事宜。

五、费用

本中心对在校学生提供免费的心理咨询服务。

六、其他情况

来访者在两次咨询的间隔期，如遇到特殊情况，工作时间可以拨打办公电话_____；

非工作时间紧急情况可以拨打当地适用的心理援助热线，热线号码为：________。

本文件一式两份，心理中心和来访者各执一份。（选用）

希望您能在咨询前充分了解上述情况。 我已经阅读并充分理解了以上内容。我愿意在 **大学心理中心 接受心理咨询服务。

来访者签字： 日期： 咨询师签字： 日期：

Document 01 ChatGPT5 modified

心理咨询知情同意书

XX 学院大学生心理健康教育与咨询中心是学校为在校学生提供心理咨询、心理训练和心理健康教育的服务机构，旨在帮助来访者促进自我成长、完善人格、开发潜能，提升心理健康水平。

为保障心理咨询的顺利开展，明确来访者与心理咨询师的权利与义务，特作如下说明：

一、服务性质

1.1 本中心为非医疗性质的心理咨询服务机构，仅为本校在校学生提供心理咨询服务，不收取任何费用。

1.2 本中心不提供以下服务：精神医学鉴定、精神类药物处方、精神疾病临床诊断、资格证明开具、对严重心理障碍个案的长期心理治疗等。

二、保密原则

2.1 心理咨询师须严格遵守职业伦理与保密原则，未经来访者书面同意，不得向第三方泄露咨询内容。

2.2 以下特殊情况除外，心理咨询师有权并有义务在必要时披露相关信息：

(1) 来访者存在严重自伤或伤害他人的危险；

(2) 来访者患有可能危及他人的重大传染性疾病；

(3) 未成年人遭遇性侵犯或虐待；

(4) 法律法规规定必须披露的情形。

三、录音录像与资料使用

3.1 为提升咨询效果、接受专业督导或用于教学与科研目的，心理咨询师可能申请录音或录像。

3.2 在进行录音（像）前，须事先征得来访者的书面同意，来访者有权同意或拒绝。

3.3 相关资料仅用于专业目的，严格保密，不会对外公开。

四、咨询安排

4.1 每次咨询时间约 50 分钟，通常为每周 1 次。如遇特殊需要，经中心批准可增加咨询频次。

4.2 心理咨询是一个持续的过程，一次咨询不一定能解决所有问题，通常需要连续、定期的多次咨询，具体次数由来访者与心理咨询师协商决定。

4.3 若来访者接受连续咨询，应在每次结束后直接预约下次时间。

4.4 来访者应按时出席咨询，如因个人原因不能到场，请至少提前一个工作日告知中心以改期或取消。若无提前通知而迟到，咨询时间不予延长。

4.5 咨询师若因故需调整时间，应提前通知来访者；因不可抗力迟到时，不予追责。

五、咨询关系与转介

5.1 来访者有权寻求其他心理咨询师意见或变更咨询师，但需经中心同意。原则上一段时间内仅能与一位咨询师建立咨询关系。

5.2 如需转介至其他咨询师，应先正式结束与当前咨询师的咨询关系。

5.3 若咨询师因专业取向或业务学习等原因需安排两位及以上咨询师同时在场，须事先征得来访者同意，并报中心审核。

5.4 若来访者或咨询师认为咨询效果不佳，可协商终止或转介。

六、来访者责任与安全

6.1 在咨询过程中，若来访者拟作出重大决定（如自杀、自伤、伤人、出走、退学、休学、离婚等），应及时告知咨询师，共同讨论并签署《生命安全契约书》。若来访者隐瞒并自行作

出该类决定，视为咨询关系自然终止。
6.2 来访者有权随时接受或停止咨询，并可自由决定是否参加咨询相关活动。

七、紧急情况处理

7.1 在两次咨询间隔期间，如出现紧急情况，来访者可直接联系本班辅导员寻求协助预约或紧急支持。
7.2 如情况严重（如存在自伤或伤人风险），应立即联系家人、辅导员或拨打急救电话 120，并可联系中心值班人员。

八、来访者权利

8.1 来访者享有知情权、自主选择权和隐私权。
8.2 来访者有权对咨询过程提出问题或意见，并有权拒绝任何不适合自身的咨询方式。
8.3 来访者可随时撤回本知情同意。

九、确认条款

本人已认真阅读本知情同意书，充分理解其中内容及权利义务，并同意接受/提供心理中心安排的咨询服务。
签名：___________
日期：_____年___月___日

Document 02 ChatGPT5 modified

心理咨询知情同意书

目的

为明确来访者与咨询师各自的权利与责任，保障咨询顺利、安全与高效进行，请您在签署前仔细阅读本同意书。

一、术语说明

本中心：指提供心理咨询服务的机构/单位。

来访者：在本中心登记并接受心理咨询服务的个人（必要时含其监护人）。

咨询师：在本中心提供心理咨询服务的专业人员。

二、服务性质与目标

心理咨询是来访者与咨询师共同合作的专业助人过程，旨在促进自我了解、缓解困扰、提升适应与成长。

心理咨询不同于医学诊疗与药物治疗；咨询效果受多种因素影响，不保证完全解决所有问题或完全符合期待。

三、咨询师的权利与义务

（一）专业与伦理

专业服务：咨询师将以专业态度提供高质量服务，不得迟到、早退或无故缺诊。

职业伦理：严格遵守国家现行法律法规及中国心理学会《临床与咨询心理工作伦理守则》。

（二）保密与例外

保密原则：来访者的预约信息、咨询记录、测验结果等资料均属保密信息，由专人集中保管；咨询师不得将记录带离中心，不得在中心外（含私人/媒体等）谈论来访者信息。

督导与教学：如因专业需要进行督导，须事先征得来访者同意，并隐去姓名及可识别身份细节。

录音录像：咨询师不得在咨询时录音、录像、拍照；如因专业需要，须获得本中心同意并征得来访者书面同意与签名。

资料访问：咨询师不可接触其他咨询师的个案记录。

（三）打破保密的条件

当接待员或咨询师评估来访者存在或已出现危害本人或他人生命安全的风险时，为保护生命安全，有权打破保密并向中心主任、相关主管部门或公安机关报告。

当公安、法院等依法出示公函时，咨询师与本中心有权在法律要求范围内提供相关信息。

（四）咨询设置与迟到/缺诊

来访者迟到或缺诊且按中心规定仍需缴费时，咨询师应在该预约时段留在诊室内，基于既往记录进行思考与准备；不得为来访者任意延时，须遵守既定设置。

（五）适配性、转介与终止

服务边界：本中心不提供精神障碍的诊断与心理治疗，并严格按《中华人民共和国精神卫生法》执行，不接待精神障碍患者。如咨询师评估来访者可能存在精神障碍或需医疗诊治，将建议转至正规医疗机构。

转介：若咨询师评估自身不适合该来访者，将在征得来访者与中心同意后，负责及时转介至中心其他咨询师或其他正规机构。

终止：若来访者连续三次请假，本中心及咨询师有权自动终止咨询关系。

四、来访者的权利与义务

（一）来访者资格

来访者定义：完成登记、缴费、填写《来访者信息登记表》与《知情同意书》，并拟在本中心接受心理咨询服务的人员（含本人及必要时的监护人）。

（二）到诊与时间管理

按时就诊：请在预约时间内到诊；如迟到或早退，咨询师仅在原预约时段内提供服务。

记忆预约：来访者或家属须自行牢记预约时间；本中心无提前提醒业务，如不确定可主动联系中心确认。
（三）信息真实与决策提示
如实告知：来访者需提供真实个人信息；如为精神障碍患者或家属，预约时有义务告知真实症状与诊断。若隐瞒或提供不实信息造成损失、伤害或法律后果，相关责任由来访者自行承担。
重大决策提示：在咨询期间不建议做出自杀、自伤、伤害他人、离婚等重大决定；如确需做出，应提前告知咨询师，以便评估风险并提供支持。
（四）预约、缴费与变更
预约制度：每次咨询结束后缴费方可预约下一次时段。
临时取消/改期：
在前一个工作日的预约时段之前通知本中心：费用可顺延或退费；
晚于上述时间才通知：该次咨询费用需照付。
违约补偿（双向约束）：
如咨询师迟到，来访者将免费获得与迟到时长等同的咨询服务；
如咨询师临时取消/改期，须在前一个工作日预约时段前通知本中心与来访者；若未能按时通知，咨询师将为来访者免费提供一次咨询。
（五）终止与退费
退费规定：咨询关系结束后，如来访者已缴费且按制度取消但尚未接受服务，请来访者本人（或费用缴纳者）在缴费之日起 3 个月内前往医院办理退费。
五、个人信息与资料管理（补充说明）
保存与安全：纸质资料由专人在中心专柜保管；如涉及电子资料，将采取权限控制与加密等措施，仅限用于本中心服务与内部必要管理。
查阅与复制：在不涉及第三方隐私与专业安全的前提下，可按中心流程申请查阅个人资料。
教学/研究用途：如需将去标识化资料用于教学或研究，将另行征得来访者的书面知情同意。
最小化原则：资料的收集与使用遵循合法、正当、必要与最小够用原则。
六、风险提示与危机处置（补充说明）
心理咨询可能引发情绪波动或短期不适（如焦虑、悲伤加重等），一般属正常反应；如出现强烈痛苦或安全风险，请立即告知咨询师或本中心。
如您或他人生命安全受到威胁（如自杀/他杀意念或计划、严重自伤风险等），请立刻寻求紧急援助（拨打当地紧急电话或前往最近医院急诊），并尽快通知咨询师以协助后续处置。
七、未成年人与监护（补充说明）
未满法定年龄的来访者需由监护人知情并签署同意；在涉及安全或法律要求的情形下，咨询师可在最小必要范围内与监护人沟通相关信息。
八、沟通与投诉（补充说明）
如对咨询服务、流程或费用有疑问或建议，可通过本中心前台/电话/邮箱进行咨询或投诉；我们将及时受理并反馈。
九、同意声明
我已认真阅读并理解本同意书所列内容，包括咨询的性质与目标、双方权利与义务、保密原则及其例外、预约与费用规则、风险提示与危机处置、个人信息管理及未成年人监护等事项。
我知悉心理咨询不等同医疗诊疗，效果不作保证；我同意遵守本中心相关制度，并自愿接受心理咨询服务。
来访者签名：__________ 日期：____年__月__日
监护人签名（如适用）：__________ 日期：____年__月__日
咨询师签名：__________ 日期：____年__月__日
本中心联系人/电话：__________ 电子邮箱：__________
文档版本号：V____（以中心发布为准）

Document 03 ChatGPT5 modified

心理咨询知情同意书

为保证心理咨询工作的专业性与效果，更好地保护来访者权益，请您在签署前**仔细阅读并理解**以下条款。签署即表示您已知悉并同意本同意书的全部内容。

一、服务对象与基本信息

（一）**服务对象与费用**

1. XX 大学心理素质教育中心（以下简称“心理中心”）面向**在校学生**提供**免费的心理咨询服务**。
2. 原则上**每学期不多于 8 次咨询**（面对面与远程网络咨询均计入次数）；一般**每周 1 次，每次 50 分钟**。如有特殊情况，可与咨询师协商调整。

（二）**咨询地点与方式**

1. 咨询地点：**XX 大学心理素质教育中心（7 斋东侧一层心理素质教育中心）**。
2. 咨询方式：面谈或远程网络咨询（含语音/视频）。远程咨询可能存在网络不稳定、信息安全风险等技术局限，中心将采取合理的安全措施降低风险。

二、来访者的权利

（一）**自主选择与撤回**

1. 您可以**自主决定**是否开始或维持咨询关系，并可在任何时间**撤回同意、终止咨询或更换咨询师**。
2. 出于对您有益的考虑，建议先与当前咨询师沟通后再做决定，以便妥善**过渡与结案**。

（二）**知情权**

有权**充分了解**咨询的目标、流程、可能收益与局限、咨询师的专业资质与理论取向等信息。

（三）**隐私与保密权**

您的个人信息与个案资料将被依法、依规保护，详见第四条“保密与例外”。

三、来访者的义务

1. **如实告知**身心状况与重要事件，及时反馈咨询目标与感受，以利评估与干预。
2. **遵守预约与时间安排**：按时出席；若迟到，**咨询不顺延**。
3. **遵守咨询边界与空间规则**：尊重自他边界与场所秩序，维护专业关系与咨询安全。

四、保密与例外

（一）**保密范围**

心理咨询过程中收集的个人资料（含个案记录、测验资料、信件、录音、录像等）均属**专业信息**，由咨询师及经授权的中心工作人员在履职需要范围内接触并**严格保密**。资料按中心制度安全存放与管理，依法在规定期限内保存与处置。

（二）**保密例外（法律与伦理要求）**

在以下情形中，咨询师**可能依法或基于防止严重危害的伦理义务**披露必要信息：

1. 存在**自杀、自伤、他杀、伤人**等**现实或高度可疑风险**；
2. **不具备完全民事行为能力者**（如未成年人、严重精神障碍者）疑似遭受**虐待或忽视**；
3. **法律法规**要求披露的其他情形。

注：如需披露，咨询师将尽可能在可行范围内向您说明披露的理由、范围与对象，并以**最小必要**为原则保护您的隐私。

五、资料使用、督导与录音录像

（一）**督导/案例研讨/写作与科研**

为**保障服务质量与提升专业胜任力**，咨询师可能申请个案**督导、案例研讨、专业写作或科研**。此类使用须**事先与您讨论并获得您的同意**，且需**去标识化处理**，避免暴露您身份的任何信息。

（二）**录音录像管理**

1. **无特别约定**，双方**均不得**对咨询进行录音或录像。
2. 仅在获得您**书面同意**且为**专业目的**（如督导）时，咨询师方可录音/录像，并按规定严格保管与使用。
3. **未经书面许可**，来访者不得录音或录像；**违反者**咨询师有权**立即终止咨询**并保留依法申诉权。

六、预约、变更与缺席

（一）**预约与签到**

预约成功后，请**自行妥善记录**咨询时间，**不再另行通知**。请于预约时间前到达中心接待室，由接待员引导入室。

（二）**迟到与顺延**

若您未提前取消且**迟到**，**咨询时间不相应延长**。

（三）**变更与缺席规则**

1. 变更或取消预约须**提前不少于 24 小时**电话告知心理中心（**62335225**）。
2. **24 小时内**变更或**未到**记为**一次临时未到**；**连续两次**或**累计三次**临时未到，将**取消咨询资格**，并于**一个月后**方可重新进入预约队列。

（四）**咨询师变更或取消**

如咨询师需变更或取消预约，将**提前不少于 24 小时**通知心理中心，由中心**及时转告**您。

七、专业边界与关系规范

1. 咨询师与来访者建立**平等、尊重、合作**的**专业关系**，**不发展**咨询以外的**任何私人关系**或利益关系。
2. 为保护隐私与边界，**一般不使用个人社交媒体**处理咨询相关事务。必要沟通请通过中心渠道进行。

八、服务性质、适用范围与转介

1. 心理咨询旨在**提升心理健康与功能**，**不等同于**医疗诊断或医疗治疗。
2. 经评估，如问题**不属心理咨询范围**，咨询师有责任**终止咨询**并**建议或协助转介**至相关医院/机构，并告知您及必要时告知亲属或监护人。
3. 若您**放弃就医或转介**，请知悉并承担**咨询效果可能不达预期**的后果。因来访者**故意隐瞒或提供虚假信息**导致**延误就医**的，咨询师与心理中心**不承担相关责任**。
4. 如就医后医院建议**辅助支持性心理辅导**，且咨询师本人同意的，咨询师可提供相应支持。

九、危机应对与紧急联系方式

1. **工作时间**：可拨打心理中心办公室电话 **62335225**。
2. **非工作时间紧急情况**：可通过“**XX 心理**”微信公众号→“**专业服务**”→“**转介信息**”获取**心理援助热线**信息。
3. 如存在**生命安全紧急风险**或需要**急救/治安援助**，请**立即联系 110/120** 或就近前往**医院急诊**，并在安全后尽快告知咨询师/心理中心。

十、数据与记录管理

1. 个案资料与测评结果由中心**专人保管**，纸质与电子资料按制度**分类存储与权限管理**，并在**法定或机构规定期限**内保存与处置。
2. 您有权就个人信息保护事宜向心理中心**咨询**与**提出建议**。

十一、未成年人或需监护同意的情形

如来访者为**未成年人**或依法需监护人同意者，需由**监护人/法定代理人**共同阅读、知悉并签署本同意书相关条款。

同意与选择（请在相应方框内✓）

1. **我已阅读并理解本《心理咨询知情同意书》，并自愿同意接受心理咨询服务。** ☐同意
2. **案例督导/专业研讨（去标识化）**：☐同意 ☐不同意
3. **录音/录像（仅限专业目的，需去标识化、严格保管）**：☐同意 ☐不同意
4. **远程网络咨询及其技术局限与信息安全风险已知悉**：☐已知悉并同意

手写确认语（请抄写）：**“我已阅读并理解上述条款，自愿同意接受心理咨询服务。”**

来访者签名：_________ 日期：**年__月__日**

（如适用）监护人/法定代理人签名：______ 日期：___年__月__日

咨询师签名：_________ 日期：___年__月__日

本协议一式两份，来访者与心理中心各执一份，**自来访者签名之日起生效**。建议您妥善保管以备查阅。

便捷提示

- 预约/变更请致电：**62335225**
- 非工作时间紧急心理援助：请通过“**XX 心理**”微信公众号获取**转介信息与援助热线**；如遇突发危机，请直接联系 **110/120** 或医院急诊。

Document 04 ChatGPT5 modified

心理咨询知情同意书

亲爱的同学:

欢迎来到心理咨询室。你的到来体现了你对自我成长和心理健康的重视。研究表明，心理咨询有助于促进自我认识、自我发展与心理调适，其效果依赖于你与咨询师的共同努力。为了保障你的合法权益，并帮助你更好地了解咨询过程，请认真阅读以下内容:

一、来访者的权利

1. **免费服务**
    - 心理咨询由学校提供，费用由学校承担，你可享受免费的心理咨询服务。
    - 咨询内容包括：环境适应、学业压力、情绪调节、人际关系、恋爱与婚姻、性心理、自我发展、家庭关系、职业生涯规划、身心健康与压力管理等。
2. **隐私与保密**
    - 你的个人信息、经历和咨询内容将被严格保密。
    - 在以下特殊情况下，咨询师有义务依法报告相关信息:
        1. 你或他人存在严重人身安全风险;
        2. 涉及社会公共安全或违法行为。
    - 为了提升咨询质量，咨询过程中可能涉及录音或录像，仅用于督导、研讨或教学研究。咨询师须事先征得你的书面同意，所有资料均做匿名化处理，并严格限于专业人员使用。
3. **自主选择**
    - 你有权选择或更换咨询师。若感到咨询师的风格或方法不适合，你可以提出更换请求。
4. **收益与风险**
    - 心理咨询的潜在收益包括：缓解心理困扰、促进心理成长、提升自我认知和心理调适能力。
    - 咨询可能带来的风险包括：在回忆往事时体验不适或产生强烈情绪反应。积极、开放和诚实的参与有助于咨询效果。
5. **中止权利**
    - 在咨询过程中，如你感到不适、不愿继续，或认为咨询无法满足需求，你有权随时提出中止或更换咨询师。

二、来访者的责任

1. **预约与出席**
    - 咨询需提前预约，预约时间为工作日 10:00–13:00、16:00–19:00。
    - 请按时出席，并于约定时间前 10 分钟到达。若因特殊原因无法前来，请至少提前 24 小时告知咨询师。
    - 每次咨询时间为 50 分钟，通常为每周一次，或根据你与咨询师协商决定。
    - 在紧急状态下，学工部会为你及时安排合适的咨询师。
2. **信息登记**
    - 在首次咨询前，你需填写《来访者基本信息登记表》，以便咨询师快速了解你的情况并提供针对性帮助。
3. **反馈问卷**
    - 为了改进与提升服务质量，咨询结束后一周内，你将被邀请填写匿名的《咨询反馈问卷》。

    ○ 问卷结果将严格保密，仅用于改进心理咨询服务，请你如实填写真实感受。

三、确认与同意

我已认真阅读并理解以上内容，明确知晓在心理咨询过程中的权利与责任，并自愿接受心理咨询服务。

来访者签名：________

日期：___年__月__日

Document 05 ChatGPT5 modified

心理咨询知情同意书

欢迎您来到 XX 大学心理服务中心。在咨询开始前，请您认真阅读并理解以下内容，以明确您与咨询师的权利与责任。本同意书为电子文档，与纸质版本具有同等法律效力。

一、服务性质与资质

1. 本次心理咨询属于北京师范大学临床与咨询心理学硕士研究生毕业设计项目，咨询全程公益、免费。
2. 本中心的实习咨询师均为经过系统专业培养的应用心理学硕士研究生，服务过程由具备资质的督导师监督与保障。
3. 咨询双方应为完全民事行为能力人。未成年人需由监护人共同签署本同意书。

二、来访者责任

1. 来访者或监护人须在协议及相关表单中提供真实、完整的信息（如联系方式、出生年份、紧急联系人、精神科诊疗史、自伤/自杀经历等）。
2. 若存在故意隐瞒或虚构信息的情况，由此产生的后果由来访者自行承担。

三、服务范围与限制

1. 根据《中华人民共和国精神卫生法》等相关规定，本中心 **不提供精神障碍的诊断与治疗**。
2. 如咨询师在咨询中发现您可能存在精神障碍或需要医学干预，将建议您前往正规医疗机构就诊。
3. 心理咨询是来访者与咨询师的共同工作过程，其效果受多种因素影响。咨询师将提供科学、专业的方法，但无法保证完全解决所有问题。

四、咨询规则

1. 咨询频率与时长：原则上为每周一次，每次 50 分钟（家庭咨询可为 80 分钟），量表填写时间不计入其中。
2. 迟到与取消：
    - 来访者迟到，咨询仍于原定时间结束；
    - 咨询师迟到，将补足时间；
    - 如需改期，请至少提前 24 小时通知。若在 24 小时内取消，仅可更改至后天或之后的时间；
    - 连续两次临时取消或两次爽约者，三个月内不得再次预约。
3. 咨询终止：来访者和咨询师均有权提出终止咨询，建议进行一次结束性会谈。
4. 实名签署：来访者应以真实姓名签署本协议，并在同一时期仅与一位咨询师建立咨询关系。
5. 网络咨询：因网络、通讯或不可抗力导致的服务中断，中心不承担责任。

五、保密原则

1. 来访者的预约信息、咨询内容、记录及相关资料均予以严格保密。
2. 在学术与督导需要下，信息仅限咨询师、督导师、论文指导教师、论文答辩委员会成员知悉，且均已签署保密协议。
3. 硕士论文撰写过程中所涉及的咨询材料将严格去标识化，不包含任何能识别您个人身份的信息。
4. 未经来访者书面授权，咨询信息不会向第三方披露。

六、保密例外情况

在以下情形下，咨询师有权不经您同意披露相关信息：

1. 您有自伤、自杀或危害他人生命安全的紧急风险；
2. 未成年人或其他无民事行为能力人遭受或疑似遭受不法侵害；
3. 司法机关依法要求提供相关信息。

七、录音录像

1. 所有实习咨询师的咨询过程均需录音录像，以便督导与教学使用。相关资料仅限于督导范围，严格保密，不作为医疗记录保存。
2. 来访者未经书面许可，不得擅自录音或录像。

八、协议的变更与终止

1. 本中心有权根据需要修订本协议及相关规则，并通过公示方式告知。修订内容自公布之日起生效。
2. 如来访者不同意相关变更，可书面通知终止本协议。
3. 来访者在退出咨询后，本协议自动终止。

九、法律效力

1. 本协议的电子版与纸质版具有同等法律效力。来访者签署（或监护人签署）后即生效。
2. 未成年人须由监护人共同签署；成年来访者若为多人同时参与咨询，需共同签署。

签署人声明：我已仔细阅读并理解上述条款，知晓并同意遵守本协议的全部内容。

来访者签名：__________ 日期：**年__月__日**

（若为未成年人）监护人签名：______

Document 06 ChatGPT5 modified

心理咨询服务相关说明（知情同意书）

亲爱的同学：

欢迎你来到 XX 大学心理健康教育中心接受心理咨询服务。为保障你的知情权与合法权益，请在正式咨询前仔细阅读以下内容：

一、心理咨询的性质

1.1 心理咨询以“助人自助”为基本原则，主要面向希望缓解心理困扰、提升自我认知和促进个人成长的健康人群。

1.2 本中心咨询师均具备国家认可的职业资质，运用专业方法，以谈话为主的形式，帮助来访者更好地理解和应对生活中的困扰。

1.3 请理解，心理咨询不同于医学诊疗或精神科治疗，其目标是支持与陪伴，而非直接治愈。

二、来访者的权利与义务

2.1 **自主性**：你有权自由选择是否开始、维持或终止咨询关系。

2.2 **真实性**：你应尽量如实、完整地提供个人情况和困扰，这有助于咨询师为你提供更合适的帮助。若信息失真，可能影响咨询效果。

2.3 **合作性**：心理咨询的效果取决于你与咨询师的共同努力。积极参与和反馈能促进咨询效果。

2.4 **转介与就诊**：若咨询师评估认为你可能存在严重心理或精神疾病（如精神分裂症、重度抑郁或双相障碍），有义务建议你前往专业医疗机构诊治。你需如实告知既往治疗经历，以便做出适切评估。

三、保密原则及例外

3.1 你的个人信息和咨询内容将被严格保密，未经允许，咨询师不得向他人透露。

3.2 保密的例外情况包括：

- （1）你存在严重自伤或伤害他人的风险；
- （2）未成年人遭遇性侵犯或虐待；
- （3）法律法规要求必须披露时。

    3.3 在专业督导或培训需要时，部分咨询过程可能被匿名使用，但均会去除可识别信息。若涉及录音、录像，须事先征得你的书面同意。

四、咨询的时间与地点

4.1 每次心理咨询约 50 分钟，通常以每周一次为基本频率，具体次数和周期由你与咨询师协商决定。

4.2 请准时到达心理咨询室（心理健康教育中心指定地点）。如需更改或取消预约，请至少提前 24 小时电话告知。

4.3 若无提前说明而缺席，可能影响后续预约安排。

五、来访者退出与转介

5.1 你有权在任何时候中止咨询，并请及时告知咨询师。

5.2 如有需要，咨询师可协助推荐其他心理咨询师或相关支持资源。

六、风险与限制说明

6.1 心理咨询不能替代药物或医学治疗，亦不能保证所有困扰都能完全解决。

6.2 在咨询过程中，可能会触及敏感话题，引发暂时的不适，这是探索与成长的常见过程。

6.3 咨询师将依据专业伦理与评估结果，与你共同商讨最适合的帮助方式。

七、来访者确认

我已认真阅读并理解以上说明，知晓心理咨询的性质、流程、权利与义务，并同意接受亳州

职业技术学院心理健康教育中心提供的心理咨询服务。
来访者签名：________
日期：____年__月__日

Document 07 ChatGPT5 modified

学生心理咨询服务知情同意书

亲爱的同学：

欢迎你来到学生心理发展指导中心（以下简称“中心”）。在咨询开始之前，请你仔细阅读并理解以下内容。本说明旨在帮助你明确心理咨询的性质、咨询双方的权利与义务，以及在紧急情况下的相关安排。

一、服务性质与目标

1. 中心是为全体在校本科生提供心理健康教育与心理咨询服务的机构，旨在协助学生促进自我成长、完善人格、开发潜能、提升生活满意度。
2. 中心提供的心理咨询服务完全免费。

二、保密原则

1. 咨询内容将被严格保密，除非经过学生本人同意，不会向他人透露。
2. 以下情况属于保密例外，心理咨询师有义务依法披露：
   （1）学生存在严重的自伤或伤害他人风险；
   （2）法律法规要求必须通报的情形。

三、咨询过程与规范

1. 心理咨询原则上为每周一次，每次约 50 分钟。若有特殊需要，可经中心同意适当增加次数。
2. 若学生因故不能按时参加咨询，应至少提前 24 小时通知中心，协商改期或取消。
3. 一般情况下，学生在同一时段仅与一位心理咨询师保持咨询关系。如需更换咨询师，须经中心同意，并在结束当前咨询关系后再开始新的咨询。
4. 心理咨询通常需要连续进行一段时间，仅一次咨询难以解决所有问题。
5. 学生在咨询期间若需做出重大决定，建议提前告知咨询师，并听取专业意见以帮助更为理性地做出选择。
6. 学生有权选择接受或中止咨询，并决定是否参与咨询过程中安排的各类活动。

四、录音录像与专业使用

1. 为了更好地帮助学生解决问题，或用于专业督导、教学与科研目的，心理咨询师可能会提出进行录音或录像。
2. 此类操作必须事先征得学生同意，学生有权拒绝而不会影响其咨询服务。

五、服务范围与限制

1. 中心的服务范围包括学习生活困扰、情绪调适、人际关系、自我发展等方面的心理评估与咨询。
2. 中心不提供以下服务：精神疾病诊断、司法鉴定、精神类药物处方、资格证明出具，以及对严重心理障碍患者的长期心理治疗。
3. 若学生可能存在精神障碍或需要医学诊疗，应及时前往相关专科医院就诊。

六、紧急情况处理

1. 咨询间隙如遇紧急心理危机，可在工作时间（8:30–11:00、13:30–20:00）拨打中心电话：85099129。
2. 非工作时间可拨打：
   - 长春市心理援助热线：12320 / 0431-89685000 / 0431-89685333
   - 全国心理援助热线：800-810-1117（固话拨打）或 010-82951332（固话与手机均可）

请在确认已充分理解以上内容后，在本知情同意书上签名。你的签名表示你理解并同意遵守

相关条款。
来访者签名：_________
日期：___年__月__日

Document 08 ChatGPT5 modified

心理咨询来访者知情同意书

亲爱的来访同学：
你好！欢迎你来到心理健康教育与咨询中心。心理咨询是一种专业的助人关系，其目标是通过咨询师的专业服务，帮助来访者缓解困扰、促进成长和提升心理健康。
为明确双方的权利与义务，确保咨询顺利开展，请在咨询前认真阅读并理解以下内容。

一、服务范围

1. 本中心向本校正式注册的学生提供心理咨询服务。
2. 咨询内容主要涉及情绪调节、人际关系、学业适应、自我成长等方面。
3. 本中心不提供精神障碍的医学诊断、药物处方、医学证明或心理治疗等医疗性质服务。

二、心理咨询设置

1. 预约方式：来访者需主动提前预约咨询。
2. 咨询地点：所有心理咨询均在本中心指定地点进行。
3. 咨询时间：一般为每周一次，每次 50–60 分钟；如情况复杂，经双方同意可延长至 90 分钟。
4. 改期与取消：如需更改或取消预约，请至少提前 24 小时告知中心。
    - 未按时通知而缺席累计 3 次及以上者，将需重新排队预约。
    - 来访者迟到不补时；迟到超过 30 分钟视为缺席。

三、咨询师的责任与义务

1. 专业服务：咨询师须提供专业、诚恳的心理服务，但不承诺能完全解决来访者的所有问题。
2. 遵守职业伦理：咨询师须遵守国家相关法律法规及专业伦理守则。
3. 保密原则：来访者的个人资料和会谈内容将严格保密。为提升服务质量，部分去标识化案例可能用于督导和教学，所有参与者均须承诺保密。
4. 保密例外：在以下情况下，咨询师有权/有义务打破保密：
（1）来访者有伤害自己或他人的严重危险时；
（2）未成年人遭受性侵害或虐待时；
（3）法律或政策要求披露时。
5. 录音录像：未经双方另行书面同意，任何人不得对咨询会谈录音或录像。
6. 缺席处理：如咨询师因特殊情况需缺席，须提前至少 24 小时告知，并为来访者妥善补时。

四、来访者的权利与义务

1. 权利：
（1）有权了解咨询师的专业背景、咨询方式及流程；
（2）有权更换咨询师；

（3）有权在任何阶段终止咨询，但建议在与咨询师充分讨论后协商决定。

2. 义务：
   （1）如实向咨询师提供个人心理状况和相关信息；
   （2）积极参与咨询过程，完成双方约定的作业任务；
   （3）遵守预约与时间规定。

3. 风险提示：心理咨询是一个探索与成长的过程，可能会带来情绪波动或暂时的不适，这属于正常反应。如出现紧急危机情况（如严重自伤、自杀念头或冲动），请立即联系紧急联系人或拨打 110/120，或前往就近医院急诊。

五、转介

若经评估发现来访者情况超出本中心服务范围或咨询师专业能力，咨询师会向来访者说明，并协助提供合适的转介建议与资源。

六、费用

本中心为在校学生提供免费的心理咨询服务。

七、文件保存

本文件一式一份，由心理健康教育与咨询中心妥善保存。

我已仔细阅读并理解上述内容，并同意遵守本协议，愿意在广东工商职业技术大学心理健康教育与咨询中心接受心理咨询服务。

来访者签名：_________ 日期：年__月__日
咨询师签名：_____ 日期：____年__月__日

Document 09 ChatGPT5 modified

心理咨询知情同意书

亲爱的同学:

欢迎你来到 XX 大学大学生心理健康教育与咨询中心。为了保障心理咨询工作的专业性与有效性，维护你和心理咨询师双方的权益，请你在咨询开始前仔细阅读以下说明，并确认理解与同意。

一、服务性质与范围

1. 心理咨询中心为非医疗性质的服务机构，仅面向本校在校学生提供心理咨询服务，**不收取任何费用**。
2. 咨询内容主要涉及个人成长、学业与职业发展、情绪困扰、人际关系、家庭适应等方面。
3. 中心**不提供**以下服务：精神病学诊断与鉴定、药物处方、法律效力证明文件、严重精神障碍的长期治疗等。

二、心理咨询的原则

1. **助人自助**：心理咨询以帮助来访者自我理解和成长为目标，而非直接替代决策。
2. **过程性**：心理咨询通常需持续和定期进行。单次咨询（约 50 分钟，每周 1 次）无法解决所有问题，具体频次和次数由你与咨询师协商确定。
3. **双重关系回避**：咨询师不会与来访者建立不符合专业原则的关系，也不得收取任何费用或额外报酬。

三、保密原则

1. 咨询师对来访者的个人信息、咨询内容负有严格保密义务。
2. 以下情况为保密例外：
   1）你有严重的自伤或伤害他人风险；
   2）涉及虐待、性侵害等需依法报告的情况；
   3）涉及法律责任或法律规定必须披露的情况。
3. 咨询师在获得你的**书面同意**后，方可进行录音录像，用于督导、教学或科研，并确保隐去个人身份信息。

四、资料与个案处理

1. 咨询过程中形成的记录将以文本档案形式妥善保存，仅限心理中心内部用于专业目的。
2. 咨询师可能在专业督导或个案研讨中引用咨询情况，但会严格隐去可识别的个人信息。

五、来访者的权利与义务

1. 你有权：
   - 自愿选择开始、暂停或终止咨询；
   - 在必要时申请更换咨询师（需通过中心安排）；
   - 拒绝录音录像；
   - 对咨询服务提出意见或投诉。
2. 你有义务：
   - 提供真实、准确的个人信息及既往精神疾病病史；
   - 在需要时遵医嘱至相关医疗机构进行检查或鉴别诊断；
   - 在咨询过程中积极配合，若涉及重大决定（如自伤、自杀、退学、休学、出走等），须及时告知咨询师以便协商应对；

- 按约定的时间参与咨询，若需变更或取消，请提前告知。

六、危机干预与安全

如在咨询中发现你存在较大自伤或他伤风险，心理咨询师有义务采取必要措施，包括通知监护人、相关部门或转介至医疗机构，以保障你和他人的安全。

七、投诉与监督

如你对咨询服务存在疑问或不满，可直接向心理健康教育与咨询中心提出，或通过学校相关渠道反映。中心将严格依照专业规范进行处理。

来访者确认

我已认真阅读并理解本《心理咨询知情同意书》的内容，知晓心理咨询的性质、原则、保密范围及例外情况，并自愿接受 XX 大学　大学生心理健康教育与咨询中心提供的心理咨询服务。

来访者签名：_________ 日期：___年__月__日

（未成年人需由监护人签名确认）

Document 10 ChatGPT5 modified

心理咨询知情同意书

亲爱的同学:

欢迎你选择心理咨询服务。为了保障你的权益，明确双方的责任与义务，请仔细阅读以下内容，并在充分理解后签署同意书。

一、咨询服务基本情况

1. **服务对象**: 本中心为在校学生提供心理咨询服务。
2. **费用说明**: 学校心理咨询服务对本校学生完全免费。
3. **咨询时间与频率**:
   - 每次咨询时间约为 50 分钟;
   - 一般为每周一次，若有需要可由咨询师与来访者协商调整。

二、来访者的权利与义务

1. 来访者有权在任何时候中止咨询。
2. 来访者需在每次咨询结束后，与咨询师预约下次咨询时间，并按时到达。
3. 如因特殊情况需临时取消或更改咨询，请至少提前 1 天通知咨询师; 若未提前告知而缺席，将视为主动放弃该次咨询，原预约时间不再保留。若仍需咨询，请重新预约。
4. 来访者应如实提供与心理困扰相关的信息，以便咨询师给予更有效的帮助。

三、咨询师的权利与义务

1. 咨询师如因特殊情况需要取消或更改咨询时间, 也会至少提前 1 天告知并与来访者协商。
2. 咨询师将遵守职业伦理规范，提供专业服务，并尊重来访者的自主权和选择权。

四、保密原则

1. 来访者在咨询过程中提供的所有信息将被严格保密，未经来访者书面同意，咨询师不得泄露给第三方。
2. 但在以下特殊情况下，咨询师有义务依法或依伦理要求解除保密:
   - 来访者有明确的自伤或自杀意图;
   - 来访者有严重危害他人的倾向;
   - 法律法规要求披露的情形。

五、录音录像与督导

1. 为提升咨询质量与专业水平，部分咨询可能在来访者同意下进行录音、录像或由督导观察。
2. 所有录音录像资料仅限于督导与专业培训使用，未经来访者再次同意，绝不会向无关人员公开。

六、声明与确认

我已仔细阅读并理解上述说明, 知晓我有权就相关内容提出进一步询问。在充分理解的基础上，我自愿接受心理咨询服务，并愿意与咨询师共同努力，促进个人成长与心理健康。

来访者签名: ________　　　咨询师签名: ________

日期: ________

Document 11 ChatGPT5 modified

心理咨询知情同意书（优化版）

为保证心理咨询的专业性与安全性，明确来访者与咨询师的权利与义务，请仔细阅读本同意书。签署即表示你已知悉并愿意遵守相关约定，且对自身行为负责。

一、服务说明与适用对象

1. 本中心面向本校在册学生提供心理咨询服务，服务原则为助人自助。
2. 心理咨询不等同于精神科诊断与治疗，不开具医疗诊断、处方或医疗证明；如评估发现需要医疗或其他专门支持，咨询师将协助转介。

二、咨询设置（保留并细化你原文的第 1、3、5、6 条）

1. **时长与频率**：每次约 **50 分钟**，通常需持续、定期进行（一般每周一次）。
2. **预约与到访**：长期咨询的来访者在每次结束后预约下次时间；请**准时到达**。若未提前通知而迟到，**咨询时间不顺延**。
3. **取消与改期**：如需临时取消或更改预约，请**至少提前 1 天**通知（[填写中心联系方式]）。未提前通知且未到者，视为**放弃本次咨询**，原时段将开放给其他同学；如仍需咨询，请重新预约。
4. **咨询师变更时间**：如咨询师因故需调整时间，将提前告知并与您协商改期。
5. **中止与更改设置**：你有权在任何时候中止咨询；如需调整频率/形式/目标，请尽早与咨询师协商。

三、费用与资质（保留你原文第 3 条并补充）

1. **费用**：本中心对本校学生提供**免费**咨询服务。
2. **专业性**：咨询由具备相应资质的心理咨询师提供，并遵循国内外现行伦理与实践指南。

四、保密与例外（保留并完善你原文第 2 条）

1. **保密承诺**：除法定或伦理例外情形外，你在咨询中的个人信息与会谈内容将被严格保密，未经你的**书面同意**不向第三方披露。
2. **保密例外（典型情形）**：
    1. 出现**自伤/他伤**等重大安全风险；
    2. 涉及**未成年人受虐待/性侵**、老年人或残障人士受虐待等需依法报告的情形；
    3. 依法需配合**司法机关或校方**在特定程序下的调查；
    4. 为**专业督导、教学或质控**之目的在**去标识化**前提下的必要讨论（录音/录像须事先征得你的同意）；
    5. 其他法律法规或伦理规范明确规定的情形。
3. 如触及保密例外，咨询师将尽可能在确保安全的前提下与你沟通。

五、来访者权利

1. 知情权与选择权：你有权了解咨询目标、方式、潜在风险与获益，并自主决定是否开始、继续或终止咨询。
2. 隐私与尊重：你有权在被尊重、无歧视的环境中接受服务。
3. 获取与转介：当问题超出本中心服务范围时，你有权获得**转介建议**与相关资源信息。
4. 咨询记录知悉：在不侵犯他人权益和不违反法律的前提下，你可按程序申请查阅与本人相关的咨询记录要点。

六、来访者义务

1. 真实、完整地提供与来访问题相关的信息；
2. 遵守预约与时间安排，尊重咨询边界（如非紧急情况不通过私人渠道联系等）；

3. 按约定完成必要的量表或作业，以提升咨询效果。

七、可能的获益与风险（新增必备条款）

1. **潜在获益**：增进自我理解、情绪调节与问题解决能力，改善人际与学习适应。
2. **可能风险**：在探讨敏感议题时，短期内可能出现情绪波动或不适；如出现明显加重，请及时告知咨询师，以便共同调整方案或转介。

八、特殊情形与在线咨询（如适用，新增）

1. **线上咨询**：需确保私密安静的环境与稳定网络；线上沟通不适合作为危机干预渠道。
2. **危机与紧急情况**：当你或他人处于**急性风险**时，请**立即**联系校内外紧急资源（如：校内心理危机热线：[**]；校园保卫处：[**]；当地紧急电话：[110/120]），并在可能时通知咨询师后续跟进。

九、信息记录与使用（补充细化）

1. 本中心将依法依规保存与管理你的预约信息、个案记录及测验资料，仅限为提供服务、督导与质量改进之必要目的使用。
2. 涉及录音/录像、科研或教学用途时，将**另行征得你的书面同意**；你有权**拒绝**，且不影响你获得咨询服务。

十、投诉与反馈（新增）

如对咨询服务有建议或投诉，可通过以下渠道反馈：[中心邮箱/电话/办公地点]。我们将按流程受理与回复。

知情同意与签署

我已仔细阅读并理解本《心理咨询知情同意书》的内容，知晓保密原则及其例外、咨询设置、可能风险与应对安排，并已就疑问得到充分解答。我**自愿**参加心理咨询，并承诺遵守相关约定。

来访者姓名：__________ 性别：___ 年龄：___ 班级：______________

联系电话：______________

紧急联系人及电话（建议填写）：____________________________________

咨询师：______________ 日期：_____年___月___日

Document 12 ChatGPT5 modified

心理咨询来访者知情同意书
亲爱的来访同学:
您好！欢迎来到华北理工大学大学生心理健康教育与咨询中心（以下简称“中心”）。
心理咨询是心理咨询师与来访者之间建立的一种专业助人关系，旨在帮助您更好地理解自我、应对困扰并促进个人成长。本知情同意书旨在明确双方的权利、责任与义务，确保咨询过程顺利、安全和有效地开展。
本中心向本校学生提供免费的心理咨询服务。根据《中华人民共和国精神卫生法》第二十三条的规定，本中心不具备精神障碍的诊断、治疗、处方开具、医疗证明开具及心理治疗资质。如在咨询中发现存在相关需要，中心将建议您前往具备资质的医疗机构，并在必要时告知学院及辅导员老师，以便学校及时提供支持与帮助。
一、心理咨询设置
预约与到访：心理咨询需提前预约，并在约定时间准时到中心。
咨询时间：一般为每周一次，每次 50 分钟。如有特殊需要，可在与咨询师协商后适当调整。
二、咨询师的责任与义务
（一）保密原则
咨询师及中心工作人员须对您的个人信息及咨询内容严格保密。
为提升服务质量，去除身份信息后，您的案例资料可能用于教学、督导或学术讨论，所有参与人员均须严格保密。
（二）保密例外
在以下特殊情况下，咨询师有权突破保密原则:
经评估，发现您存在严重的自伤、他伤或危害社会安全的风险；
经评估，发现您有明确的自杀可能；
您患有可能危及他人的严重传染性疾病；
涉及性侵犯、虐待或严重违法行为；
法律法规明确要求披露的情况。
（三）录音录像
原则上，未经双方同意，任何人不得对咨询会谈进行录音或录像。
若因督导或培训需要，咨询师需提前说明，并与您签署录音录像知情同意书。
三、来访者的权利与义务
如实告知与积极参与：请如实告知相关情况，包括既往在医疗机构的诊疗信息，并积极配合咨询工作。若因隐瞒信息造成不良后果，需自行承担相应责任。
自主权：您有权随时终止咨询，但建议与咨询师充分讨论，并在双方协商后决定。
预约与守时:
如需更改或取消预约，请至少提前 24 小时电话通知中心。
若无正当理由迟到或取消，累计达 3 次，本学期将需重新排队预约。
迟到时间不予补足；如迟到超过 30 分钟，本次咨询视为取消。
四、紧急情况处理
在非咨询时间，若您遇到紧急心理危机，可联系以下 24 小时心理援助热线:
河北省心理援助热线：0312-96312，0311-68052995
北京市心理援助热线：010-82951332，010-58303286，010-58340263
如情况紧急，请立即拨打 110 或前往就近医院急诊。
确认与签署
我已认真阅读并充分理解以上内容，自愿在华北理工大学大学生心理健康教育与咨询中心接受心理咨询服

务。

来访者签名：_________ 日期：年__月__日

咨询师签名：_____ 日期：___年__月__日

Document 13 ChatGPT5 modified

心理咨询知情同意书

亲爱的同学:

您好! 欢迎您来到心理咨询中心。感谢您对我们工作的信任, 愿意将内心的声音与我们分享。心理咨询是一段由您与咨询师共同参与的探索与成长之旅, 我们将竭诚为您提供专业、真诚与安全的心理支持。

为保障您的权益, 确保咨询的顺利开展, 请您仔细阅读以下内容:

一、咨询服务说明

(一) 服务对象

本中心为本校在读学生提供免费的心理咨询服务。心理咨询不同于医学诊断和治疗, 不提供药物处方、医疗证明或临床治疗。

(二) 咨询方式与时间

1. 一般情况下, 心理咨询为**每周一次**, 每次约 **50–60 分钟**, 如有需要可由来访者与咨询师协商调整。
2. 请您按时赴约。如需取消或变更预约, 请**至少提前 5 小时**通知咨询师。若未及时通知且无故缺席, 视为主动放弃本次咨询。
3. 咨询师如需调整时间, 将提前与您沟通。

(三) 保密原则

心理咨询遵循严格的保密制度, 您的个人信息、咨询内容及相关资料 (包括记录、测验、音视频等) 将受到保护。未经您的书面同意, 不会向第三方泄露。但以下情况除外:

1. 您已授权披露的情形;
2. 存在严重的自我伤害或伤害他人风险时, 为了保障安全, 咨询师可能需要联系相关部门或您的家人;
3. 涉及法律责任或司法调查时 (如危害公共安全、暴力犯罪等), 咨询师需依法披露;
4. 咨询师因督导或学术讨论需要使用相关资料时, 将严格去除可识别个人的信息, 并事先征得您的同意;
5. 来访者为未成年人时, 其监护人依法享有必要的信息知情权。

二、来访者的权利与义务

(一) 权利

1. 您有权自主选择是否接受咨询, 以及选择或更换咨询师;
2. 您有权决定咨询的进展或中止;
3. 您有权了解咨询师的资质、咨询方法和可能的效果;
4. 您的隐私将受到尊重和保护。

(二) 义务

1. 请您如实提供与问题相关的个人资料, 并积极参与咨询过程;
2. 尊重咨询师, 遵守预约时间和中心相关规定;
3. 积极与咨询师合作, 完成双方商定的作业或任务, 共同探索问题的解决方式。

三、咨询师的责任与义务

(一) 遵守专业与伦理

咨询师须遵守国家法律法规及专业伦理守则, 确保咨询过程安全、合规与专业。

(二) 尊重与接纳

咨询师将真诚、耐心、尊重地对待每一位来访者, 接纳您的情绪与感受, 建立互相信任的咨询关系。

（三）保密与例外
咨询师严格遵守保密制度，并在必要时向您说明例外情况（详见“一（三）”）。
（四）专业胜任
当咨询师因专业限制无法继续有效帮助时，将坦诚告知，并协助您转介至其他合适的专业机构或人员。
四、紧急情况处理
若在咨询过程中或非咨询时间内，您出现严重的自伤或伤害他人风险，请立即联系：
- 学校心理健康教育中心：_______
- 校医院急诊电话：_______
- 校外紧急救援电话：120/110

咨询师将在其专业职责范围内，尽力协助您获得及时与合适的帮助。
五、签署确认
我已认真阅读并理解本知情同意书的全部内容，知晓并认可心理咨询的相关原则与规定，同意在自愿的前提下接受心理咨询服务，并履行相应的权利与义务。
来访者签名：_________ 联系电话：_________
咨询师签名：_________ 联系电话：_________
日期：___年__月__日

Document 14 ChatGPT5 modified

心理咨询知情同意书

亲爱的同学:

您好！欢迎来到学生心理健康教育与咨询中心。为了保障心理咨询顺利进行、明确双方权利与义务，请您在接受咨询前仔细阅读以下内容，并在同意后签署本知情同意书。

一、心理咨询的基本信息

1. **服务对象**：本中心服务对象为全日制在读学生（不含休学学生）。
2. **咨询地点**：学生心理健康教育与咨询中心指定的心理咨询室。
3. **咨询时间**：每次咨询约 90 分钟，一般为每周 1 次；每学期最多 6 次。若需继续咨询，须经评估后决定是否延长。
4. **预约与守时**：来访者需按预约时间准时到达咨询室，若迟到，咨询时间不予顺延。

二、预约与变更规定

1. **来访者取消或更改预约**：需至少提前 1 个工作日通知中心。若未提前通知或缺席，仍计入 6 次咨询总数。
2. **缺席处理**：若来访者缺席超过 2 次（包括无故缺席、临时请假或提前请假），将暂停当前阶段咨询资格，需重新预约排队。若无故缺席并失联超过 48 小时，中心有权联系院系，保障您的学业与人身安全。
3. **更换咨询师**：来访者仅可在第一次咨询结束后提出更换咨询师的请求。
4. **咨询师取消或变更预约**：咨询师如因特殊原因需更改预约时间，也须提前 1 个工作日通知中心。

三、咨询师与来访者的权利与义务

1. **来访者的权利**：
    - 有权知情并自主决定是否开始、维持或终止咨询。
    - 有权在知情同意范围内参与咨询过程，包括了解咨询师资质和咨询方式。
    - 有权在第一次咨询后申请更换咨询师。
2. **咨询师的义务**：
    - 遵守职业伦理，尊重并支持来访者。
    - 提供专业的心理支持与帮助，但不承诺一定达到来访者预期效果。
    - 如因督导、教学或科研需要需录音/录像，必须事先取得来访者同意，且会妥善保护资料安全。

四、保密原则与例外情况

1. **严格保密**：来访者在咨询中提供的个人信息、经历和体验将严格保密。未经本人书面同意，不会向第三方透露。
2. **保密例外**（在以下情形下，咨询师有权或有义务打破保密）：
    - 来访者有自伤或伤害他人的严重危险；
    - 涉及法律法规明确规定必须报告的情况；
    - 司法部门依法要求提供相关记录时。
3. **资料保存**：咨询记录仅用于中心内部工作，妥善保管，不向任何机构或个人（包括来访者本人）提供。

五、服务范围与限制

1. 本中心提供与学习生活、个人成长和自我发展相关的心理评估与咨询服务。
2. 不提供以下服务：精神障碍的诊断、司法鉴定、开具药物处方、出具资格证明或医学证明、对严重心理障碍患者的长期心理治疗等。

3. 如来访者被怀疑存在精神障碍，将建议前往有资质的专科医疗机构就诊。

六、教学与科研说明

本中心承担临床与咨询心理专业研究生的培养任务，部分咨询过程中可能涉及督导、跟诊或研究调查。所有相关活动均会遵守伦理规范，必要时将事先征求您的同意。

七、确认与签署

请确认：

- 我已完整阅读以上内容；
- 我理解并同意遵守相关规定；
- 我自愿参加心理咨询，并为自己的选择负责。

姓名：________

日期：___年__月__日

Document 15 ChatGPT5 modified

心理咨询知情同意书

亲爱的来访同学:

您好！欢迎您来到大学生心理健康中心。为了保障您的权益，明确您与咨询师在心理咨询过程中的权利与义务，请在正式咨询前仔细阅读并理解以下内容:

一、心理咨询服务说明

1. **服务性质与范围**
   - 本中心为非医疗性质的心理咨询服务机构，仅面向本校在校学生，所有服务均为公益性质，不收取任何费用。
   - 本中心不提供精神障碍的诊断、治疗、处方开具、医疗证明开具和心理治疗等医疗性服务。若存在相关需求，我们将建议并协助您转介至专业医疗机构。
2. **预约与地点**
   - 咨询需由有意愿的学生提前预约。
   - 所有心理咨询均在本中心指定场所（教学楼 104 室）进行。
3. **咨询时间与频率**
   - 每次咨询时间一般为 50 分钟，通常以每周一次为原则。
   - 心理咨询是一个循序渐进的过程，一次咨询可能无法完全解决问题，通常需要持续而定期的连续咨询。
   - 原则上，每位学生每学期最多可接受 8 次咨询，具体次数由来访者与咨询师共同商定。

二、咨询师的责任与义务

1. **专业服务**
   - 咨询师将基于专业训练和伦理规范，竭诚为来访者提供心理咨询服务。
   - 咨询师会根据您的实际情况提供适合的咨询方法和支持，但心理咨询的效果需要双方共同努力，不能保证完全满足所有预期。
2. **保密原则**
   - 咨询师及中心工作人员必须严格遵守保密制度，确保来访者的个人信息与咨询内容不被泄露。
   - 所有案例的使用和资料存档均遵循国家法律法规及心理学伦理规范。
3. **保密例外**
   在以下情形下，咨询师可能依法或基于保护生命安全的考虑，不得不披露相关信息:
   - 来访者存在严重的自伤或伤害他人的危险;
   - 涉及法律要求必须披露的情况;
   - 来访者为未成年人，且出现被侵害、虐待等情况。
4. **时间约定**
   - 咨询师应遵守咨询时间。如因特殊情况需调整，咨询师需至少提前 24 小时告知中心并与来访者重新预约。

三、来访者的权利与义务

1. **如实告知**
   - 来访者应尽可能如实地向咨询师告知自身情况，包括既往接受过的心理评估或诊断。
   - 若未如实告知而导致不良后果，责任由来访者自行承担。
2. **咨询自主权**

    - 来访者有权选择或变更咨询师，但需经中心协调。原则上同一阶段仅与一位咨询师建立咨询关系。
    - 来访者有权随时终止咨询，但建议与咨询师讨论后协商决定。
3. **出勤与时间管理**
    - 来访者若需改期或取消，应至少提前 24 小时告知中心。
    - 若迟到，咨询时间不予延长；迟到超过 15 分钟或未通知缺席，本次咨询视为取消。
4. **重大决定的告知义务**
    - 在咨询期间，如来访者计划做出重大决定（如自伤、自杀、伤人、出走、退学、休学等），请务必告知咨询师并协商处理。若未告知，则视为咨询关系的自然终止。

四、转介说明

- 初次访谈后，中心将根据评估结果为来访者安排合适的后续咨询。
- 若来访问题超出本中心职责或咨询师能力范围，咨询师会及时说明并协助转介到合适的专业机构或资源。

确认与签署

请您在充分理解上述内容的基础上，自愿决定是否接受心理咨询服务。

我已阅读并理解以上内容，愿意在吉林体育学院心理健康教育中心接受心理咨询服务。

来访者签字：________ 日期：**年__月__日**

咨询师签字：_____ 日期：___年__月__日

Document 16 ChatGPT5 modified

心理咨询知情同意书

尊敬的来访同学:

您好！感谢您选择 XX 大学心理健康教育中心的心理咨询服务。心理咨询是一种专业助人关系，旨在帮助心理健康状态正常的在校学生应对生活适应、学业科研、人际交往、恋爱情感、家庭关系、人生价值、发展规划、压力情绪等方面的困惑与困扰。

本着“以人为本、助人自助”的原则，我们希望通过本《知情同意书》明确来访者与咨询师的权利与义务，共同保障咨询的顺利开展。请您仔细阅读以下内容:

一、心理咨询的设置

1. **保密原则**
    - 咨询师必须严格保守来访者的个人信息和咨询内容。
    - 以下情形属保密例外:
      （1）来访者出现自伤或伤害他人的严重危险;
      （2）咨询内容涉及法律责任;
      （3）来访者因严重心理失常需转介至医疗机构。
2. **咨询服务**
    - 学校心理中心对在校学生提供免费心理咨询。
    - 每次咨询时长约 50 分钟，一般每周 1 次，次数由来访者与咨询师协商确定。
    - 如因特殊原因无法按时参加，来访者或咨询师须至少提前半天通知预约人员，由专人告知对方。
3. **预约原则**
    - 一般情况下，来访者一周仅可预约 1 次咨询。
    - 原则上同一时期仅可预约 1 位咨询师；在咨询关系结束后，可预约其他咨询师。
4. **督导与录音录像**
    - 如咨询师需将咨询用于专业督导或教学科研，须事先征得来访者同意，并隐去所有可识别信息。
    - 若需录音或录像，须另行签署《录音/录像知情同意书》。
5. **专业边界**
    - 为避免双重关系影响，咨询师不得与来访者建立咨询关系以外的其他关系，不得接受来访者礼物。
    - 如咨询师因个人或专业限制无法继续提供服务，应坦诚说明并协助来访者进行转介。

二、来访者的权利与义务

1. **权利**
    - 来访者自愿参与心理咨询，有权主动预约、选择或更换咨询师。
    - 来访者有权了解咨询流程、咨询师资质及理论取向，并决定是否继续咨询。
2. **义务**
    - 以积极、坦诚的态度参与咨询，如实表达自己的体验与想法;
    - 尊重咨询师，遵守约定的咨询时间与安排;
    - 理解心理咨询是一个过程，问题的解决与成长需要持续的努力。

三、咨询师的权利与义务

1. **真诚与尊重**
    - 咨询师将以真诚、耐心、尊重的态度对待来访者，建立互信的关系。
2. **保密**
    - 咨询师严格遵守保密原则，仅在保密例外情形下依法或依规定披露信息。
3. **专业责任**
    - 咨询师应遵守国家法律法规及职业伦理守则，提供科学、专业的服务。

四、其他约定事项

1. 来访者如产生自伤或伤害他人的想法或行为，应及时告知咨询师，以便获得必要支持和转介。
2. 在咨询过程中，咨询师与来访者将共同讨论目标与重点，必要时可签署书面协议。
3. 本《知情同意书》由咨询师妥善保存并严格保密。
4. 特殊人群说明：若来访者为未成年人或在认知/行为上存在特殊情况，则需监护人同意并共同参与相关约定。

咨询师签名： **来访者签名：**

年 月 日 年 月 日

Document 17 ChatGPT5 modified

心理咨询知情同意书

亲爱的来访同学:

您好! 欢迎您来到本中心接受心理咨询服务。心理咨询是来访者与心理咨询师之间建立的一种专业助人关系，旨在帮助您更好地认识自我、缓解困扰、促进成长。为保障双方的权益，请您在咨询前认真阅读并理解以下内容。

一、服务说明

1. 本中心仅向本校正式注册学生提供 **免费的心理咨询服务**。
2. 本中心 **不提供** 精神障碍的诊断、药物处方、医疗证明开具及心理治疗等医疗性质的服务。
3. 咨询频率与形式:
    - 一般为每周一次，每次约 50 分钟;
    - 咨询需由来访者主动预约，并在中心指定地点进行。

二、心理咨询师的责任与义务

1. **遵守保密原则**: 所有涉及来访者的个人资料和咨询内容均严格保密。
2. **保密例外**: 在以下情形下，咨询师有义务依法或依伦理规范披露必要信息:
   (1) 来访者有严重的自伤或伤害他人风险;
   (2) 未成年人受到性侵犯或虐待;
   (3) 法律法规规定必须披露的情况。
3. **录音录像**: 未经双方事先书面同意，不得进行录音录像。若因督导、教学或科研需要，咨询师将另行征得来访者同意，并保证隐去可识别信息。
4. **时间遵守**: 咨询师应准时到场。如无故迟到或缺席，应补足来访者的咨询时间。

三、来访者的权利与义务

1. 来访者有权:
   (1) 了解咨询师的专业资质和咨询方式;
   (2) 在一学期内最多一次申请更换咨询师;
   (3) 终止咨询，但建议与咨询师讨论后，经双方协商决定。
2. 来访者有义务:
   (1) 如实告知个人情况及既往诊疗经历;
   (2) 积极配合咨询过程，完成双方商定的任务;
   (3) 遵守咨询时间与规定:
   - 改期或取消需至少提前半天通知;
   - 无故迟到或缺席累计 3 次需重新预约;
   - 迟到不补时，超过 30 分钟视为缺席。

四、转介说明

1. 初始访谈后，中心将根据情况安排是否继续咨询。
2. 若来访者的问题超出本中心服务范围，或超出咨询师的专业能力，咨询师将协商并推荐合适的外部资源。

五、紧急情况

1. 在非咨询时段，如遇到紧急心理危机，可拨打:
    - 中心电话: 0816-6905269 (游仙); 0816-3963291 (安州)
      (服务时间: 周一至周五 9:00–12:00, 14:30–17:30, 19:00–21:00)
    - 绵阳市 24 小时公益心理援助热线: 0816-2424666、0816-2268885。

2. 如出现严重安全风险，请立即寻求校内外应急资源，必要时拨打 **110** 或 **120**。

六、特别说明

1. 心理咨询的效果依赖于双方的共同努力，咨询师不保证一定能解决所有问题或完全符合来访者期待。
2. 本同意书体现了来访者的 **自愿参与**。您有权在知情的前提下做出是否接受服务的决定。

确认声明

我已阅读并充分理解以上内容，愿意在 XX 学院大学生心理健康教育与发展服务中心接受心理咨询服务。

来访者签字：________ 日期：**年__月__日**

咨询师签字：_____ 日期：___年__月__日

Document 18 ChatGPT5 modified

个体心理咨询知情同意书（优化版）

亲爱的来访同学：

您好！欢迎来到 XX 大学大学生心理辅导与服务中心。

心理咨询是来访者与心理咨询师之间建立的一种专业助人关系，旨在帮助您更好地理解自我、应对困扰与促进成长。为了明确双方的权利与义务，保障心理咨询的顺利开展，请您在咨询前仔细阅读并理解以下内容：

一、心理咨询服务说明

1. 服务对象：本中心面向本校正式注册的学生，提供专业心理咨询服务。
2. 服务性质：心理咨询不属于医疗行为，中心不提供精神障碍的诊断、药物处方、医疗证明开具和心理治疗等服务。
3. 服务方式：主要以面对面谈话为主，必要时可辅以心理测评或其他专业方法。

二、咨询安排

1. **预约**：来访者应主动提前预约。
2. **地点**：咨询在本中心指定的咨询室进行。
3. **时间**：一般每周一次，每次 50 分钟。特殊情况可由咨询师与来访者协商调整。
4. **迟到与缺席**：
    - 来访者迟到，咨询时间不予补足或延长；
    - 来访者迟到超过 10 分钟或缺席，当次咨询自动取消；
    - 咨询师若无故迟到或缺席，中心将为来访者补足时间或妥善安排。

三、咨询师的责任与义务

1. **专业性**：咨询师承诺提供高质量的专业心理服务，但不保证所有问题完全解决。
2. **保密**：来访者的个人信息与会谈内容严格保密。案例可能在隐去身份信息的前提下用于督导、教学或科研，所有参与人员均须遵守保密规定。
3. **保密例外**：以下情况咨询师有义务依法或依规披露信息：
    （1）来访者存在严重的自伤或伤害他人的危险；
    （2）不具备完全民事行为能力的未成年人遭遇虐待或性侵犯；
    （3）法律法规要求披露时。
4. **录音录像**：如因督导或专业发展需要进行录音录像，须事先征得来访者书面同意。

四、来访者的权利与义务

1. **权利**
    （1）有权了解咨询流程、咨询师资质及理论取向；
    （2）有权提出更换咨询师；
    （3）有权在与咨询师讨论后选择终止咨询。
2. **义务**
    （1）如实告知个人心理状况及相关就诊信息；
    （2）积极参与咨询，配合完成双方商定的任务；
    （3）如需改期或取消咨询，应提前告知中心。
    注：因隐瞒信息导致的不良后果由来访者自行承担。

五、转介

如来访者的情况超出本中心服务范围或超出咨询师的专业胜任力，咨询师将说明原因并协助来访者转介至更合适的资源。

六、费用

本中心为本校学生免费提供心理咨询服务。

七、紧急情况

1. 工作时间内可拨打中心办公电话（0471-4305710）；
2. 非工作时间如遇心理危机，可拨打中心 24 小时心理援助热线（0471-4306364）；
3. 若出现严重自伤或伤人危险，请第一时间拨打 110 或 120 寻求紧急帮助。

八、知情确认

我已认真阅读并充分理解上述内容，愿意遵守相关规定，并在 XX 大学大学生心理辅导与服务中心接受心理咨询服务。

来访者签名：________ 日期：___年__月__日

（如为未成年人，应由监护人共同签署）

Document 19 ChatGPT5 modified

优化后的版本

编号：_______

心理咨询知情同意书

亲爱的来访同学:

您好! 感谢您信任并选择山东农业工程学院大学生心理健康教育中心（以下简称“本中心”）。心理咨询是一种专业的助人关系，旨在通过咨询师的专业服务，帮助来访者更好地认识自我、缓解困扰、促进成长。

为了保障您的知情权与合法权益，请您在咨询开始前仔细阅读以下内容。若有不理解的地方，您可随时向咨询师提出并获得解释。

一、服务说明

1. **服务对象**：本中心仅向本校正式注册的在校学生提供专业心理咨询服务。
2. **服务范围**：本中心不提供精神障碍的诊断、治疗、药物处方、医疗证明开具或心理治疗等医疗性质服务。
3. **咨询设置**：
   （1）预约：来访者需提前主动预约。
   （2）地点：咨询在本中心指定地点进行。
   （3）时间：原则上每周一次，每次约 50 分钟。

二、咨询师的责任与义务

1. **保密原则**：
   （1）咨询师及相关工作人员须严格保密来访者的个人资料和会谈信息。
   （2）为保障服务质量，来访案例可能在隐去身份信息后，用于督导与专业研讨，所有参与者均承担保密义务。
2. **保密例外**（以下情形不适用保密）：
   （1）来访者有自伤或伤害他人的严重危险；
   （2）未成年人遭遇性侵犯或虐待；
   （3）法律法规规定必须披露时。
3. **录音录像**：
   咨询会谈不得擅自录音录像。如因督导或教学需要，咨询师须事先征得来访者书面同意并签署额外协议。
4. **出勤规定**：
   咨询师若未提前 24 小时通知中心而缺席，应为来访者补足咨询时间。

三、来访者的权利与义务

1. **权利**：
   （1）有权知情并自主决定是否接受或继续咨询；
   （2）有权申请更换咨询师，每学期最多 1 次；
   （3）有权中止咨询，但建议在与咨询师充分讨论后协商决定。
2. **义务**：
   （1）如实告知自身情况（包括既往诊断和治疗），积极配合咨询；
   （2）遵守预约时间，若需改期或取消，应至少提前 24 小时通知中心；
   （3）若累计 3 次无正当理由迟到或缺席，本阶段咨询将终止，需重新预约。
3. **迟到规定**：
   迟到时间不予补足；迟到超过 30 分钟视为缺席。

四、转介

如评估发现来访者问题不属于本中心服务范围，或超出咨询师专业能力，咨询师会及时说明并提供合适的求助资源，协助进行转介。

五、费用

本中心为本校学生提供心理咨询服务，**全程免费**。

六、风险与突发情况

1. 心理咨询是一个过程，效果因人而异，需来访者与咨询师共同努力。
2. 在咨询过程中，若因来访者心理状况复杂或严重，可能出现以下情况：

   （1）精神病性症状发作；

   （2）自伤、自杀或伤害他人等行为。

   出现上述情况时，咨询师将依法依规采取必要措施，并可能建议转介至医疗机构。本中心不对因个体心理状况导致的突发事件承担法律责任。

七、危机干预与紧急联系方式

1. 工作时间可拨打本中心电话：0531-88117870。
2. 非工作时间，如遇紧急情况，可拨打心理援助热线：
   - 全国生命危机干预 24 小时热线：400-161-9995（1 学生专线，2 抑郁专线，3 生命专线）；
   - 济南市医学心理咨询中心热线：0531-85713287 / 85713289。

八、来访者确认

我已认真阅读并理解上述内容，并在必要时获得了充分解释。我自愿接受本中心的心理咨询服务，并承诺遵守相关约定。

来访者签字：_______ 日期：**年__月__日**

咨询师签字：___ 日期：___年__月__日

Document 20 ChatGPT5 modified

心理咨询知情同意书

亲爱的来访同学：

您好！感谢您信任并选择心理咨询服务。为了保障您与心理咨询师的权益，促进咨询工作的顺利开展，请您仔细阅读以下内容，并在知情理解的前提下自愿签署本同意书。

一、心理咨询的性质

1. 心理咨询是来访者与经过专业训练的心理咨询师之间建立的专业助人关系，旨在帮助来访者缓解心理困扰、增进心理健康与自我成长。
2. 心理咨询不同于精神科的诊断与治疗，不能替代药物治疗或其他医学手段。如有相关医疗需要，请遵循医生的建议。

二、保密原则

1. 咨询师将严格遵守心理咨询保密原则，保护来访者的个人隐私。来访者的资料与会谈内容不会向无关人员泄露。
2. 在以下特殊情况下，咨询师有权在必要范围内披露相关信息：
   （1）来访者存在自伤或伤害他人的现实危险；
   （2）来访者的问题涉及法律责任；
   （3）为提升专业水平需要进行督导或个案研讨，但会去除可识别的个人信息，仅限于专业场合。

三、个人信息与资料管理

1. 为保证咨询的持续性与安全性，来访者需如实提供姓名、联系方式及紧急联系人等信息，仅用于心理咨询管理与必要的安全保障，不会泄露给无关单位或个人。
2. 如需进行录音或录像，咨询师将提前告知，并征得来访者的明确同意。

四、咨询设置

1. 每次咨询时间一般为 50 分钟。咨询次数由来访者与咨询师根据需要协商决定。
2. 双方应遵守约定时间，若需变更或取消，请提前告知。

五、责任说明

1. 心理咨询师将竭力提供专业支持，但不能保证所有问题在短期内解决。
2. 对于严重心理障碍（如精神分裂症、严重抑郁症、自杀风险等），咨询师会建议来访者及时就医，并在必要时将情况告知监护人或相关责任人，以确保安全。
3. 因来访者及其家人隐瞒关键信息而导致的后果，由来访者自行承担。

六、来访者的权利

1. 来访者有权在任何时候提出问题，并获得相关解释。
2. 来访者有权选择、拒绝或中止咨询服务。
3. 来访者有权要求更换咨询师。

本人已认真阅读并理解以上条款，知晓心理咨询的性质、权利与义务，并同意自愿参与心理咨询。

来访者签名：________

咨询师签名：________

日期：___年__月__日

Document 21 ChatGPT5 modified

心理辅导与咨询知情同意书

亲爱的来访同学：

您好！感谢您对本中心的信任。为了保障心理咨询工作的顺利开展，明确来访者与咨询师的权利与义务，请您仔细阅读以下说明，并在签字确认后表示您已理解并愿意遵守。

一、咨询的目的

心理辅导与咨询旨在帮助来访者：

1. 提升心理健康水平，促进自我理解与成长；
2. 尽早发现心理不适，并进行科学的预防与干预。

二、自愿原则与信息提供

1. 来访者进入本中心接受服务完全出于自愿。
2. 来访者应如实提供相关信息。若因隐瞒或失真而影响咨询效果，由此产生的后果由来访者本人承担。

三、咨询关系与权利

1. 咨询在互信、理解的基础上进行，咨询方案由咨询师与来访者协商确定。
2. 来访者有权：
   （1）选择或更换咨询师；
   （2）与咨询师讨论任何相关问题；
   （3）在必要时主动中止咨询。
3. 咨询师亦有权：
   （1）基于来访者的福祉提出转介或中止咨询；
   （2）在专业能力或服务范围之外，建议来访者寻求其他合适的帮助。

四、咨询设置

1. 咨询需提前预约。
2. 每次咨询时长一般为 50 分钟，通常以每周一次为原则，次数由来访者与咨询师协商决定。
3. 本中心不提供精神障碍的诊断、药物处方、医疗证明开具及心理治疗等医疗性服务。

五、保密原则

1. 中心及咨询师将对来访者的个人信息和会谈内容严格保密。
2. 若因教学、督导或科研需要使用相关资料，须事先征得来访者同意，并对信息进行匿名化处理。
3. 以下情形属于保密例外情况：
   （1）来访者有自伤或伤害他人的严重危险；
   （2）来访者患有可能危及他人的严重传染性疾病；
   （3）未成年人受到性侵犯或虐待；
   （4）法律或政策规定必须披露时；
   （5）来访者的问题超出心理咨询服务范围，需要联系相关人员以转介帮助。
4. 在必要时，中心会以专业方式联系来访者的亲属、辅导员或其他相关支持系统，以最大程度保障来访者的安全与权益。

六、其他说明

1. 若咨询需要录音、录像或使用相关资料，须事先征得来访者书面同意。
2. 咨询效果取决于来访者与咨询师的共同努力，咨询并不能保证完全解决所有问题。
3. 来访者在咨询过程中享有平等、尊重与安全的权利。

来访者签名: __________ 日期: **年__月__日**

咨询师签名: ______ 日期: ___年__月__日

Document 22 ChatGPT5 modified

心理咨询知情同意书

亲爱的来访同学：

您好！欢迎来到 XX 大学大学生心理健康教育中心（以下简称“心理中心”）。

心理咨询是一种专业助人关系，旨在帮助来访者缓解困扰、促进自我理解与成长。为保障咨询顺利开展，明确双方的权利与义务，请您在咨询前仔细阅读并确认以下内容：

一、保密原则

1. 心理中心及咨询师须对来访者的个人资料和咨询内容严格保密，未经本人书面同意不得向第三方透露。
2. 以下情形属于保密例外：
   （1）来访者存在自伤或伤害他人的严重危险；
   （2）来访者患有可能危及他人的重大传染性疾病；
   （3）未成年人遭受性侵犯或虐待；
   （4）法律法规要求披露；
   （5）咨询师判断来访者可能出现行为失控，危及校园安全或教学秩序时。此类情况，咨询师有权通知来访者辅导员或紧急联系人。
3. 为提高咨询质量或用于教学、督导与科研，咨询师可能申请录音/录像。须在咨询前取得来访者的**书面同意**，来访者有权同意或拒绝。

二、咨询时间与次数

1. 每次咨询时间约为 **45 分钟**。请来访者准时到场，迟到将不顺延咨询时间。
2. 如需取消或更改时间，请至少提前 **1 个工作日**告知。每学期请假次数原则上不超过 2 次。若无故缺席 1 次，心理中心将取消本学期后续咨询安排。
3. 咨询通常需要持续过程，具体次数由来访者与咨询师协商决定。
4. 因资源有限，每位学生每学期的常规咨询次数（含请假）原则上不超过 **8 次**。

三、咨询的终止与转介

1. 达成咨询目标后，咨询自然终止。
2. 若来访者或咨询师认为咨询效果不佳，可经协商决定终止或进行转介。
3. 来访者有权随时选择接受、暂停或终止咨询，并决定是否参与相关活动。

四、咨询过程中的责任与义务

1. 来访者需提供真实的个人信息及相关情况，以帮助咨询师准确理解与评估问题。
2. 来访者在咨询期间有义务避免任何自伤或危害他人的行为。若出现严重心理或行为问题，咨询师可建议转介至医疗机构进一步评估和治疗。
3. 对于正在接受精神科药物治疗的个体，如其本人及家属希望接受心理咨询，且来访者具备参与咨询的能力，心理中心可提供支持性咨询。
4. 咨询师与来访者应保持专业关系。原则上不建立咨询之外的私人联系（如互加微信、QQ、交换手机号等）。如需确认或调整咨询时间，请通过心理中心电话 **62216906** 联系。

五、服务说明

1. 心理中心面向本校全体学生提供**免费**心理咨询服务，主要涵盖个人成长、自我发展及适应性问题。
2. 心理中心 **不提供** 以下服务：精神病学诊断与鉴定、药物处方、医疗证明或资格证明开具等。
3. 如遇超出心理中心服务范围的情况，咨询师将建议来访者转介至相关专业医疗机构。

六、其他说明
未尽事宜，由双方协商补充。
签署确认：
心理咨询师：___________
来访学生：___________ 电话：___________
紧急联系人：___________ 电话：___________
日期：_____年___月___日

Document 23 ChatGPT5 modified

心理咨询知情同意书

亲爱的来访同学：

您好！感谢您对本中心的信任。心理咨询是心理咨询师与来访者之间建立的一种专业助人关系，旨在通过专业支持帮助来访者提升心理健康水平。为保障双方权益并确保咨询工作的顺利进行，请您在正式咨询前认真阅读以下说明：

一、服务对象与性质

1. 本中心为本校全日制在读学生（不含休学学生）提供免费的心理咨询服务。
2. 本中心不提供以下服务：精神障碍的诊断与治疗、药物处方、医疗证明出具以及心理治疗等医疗性质服务。

二、咨询设置

1. 咨询地点：三个校区的心理咨询室。
2. 咨询时间：一般为每周 1 次，每次 50 分钟。
3. 咨询次数：每学期最多可接受 8 次咨询，原则上不得在同一天预约多个时段。特殊情况可由咨询师评估后酌情处理。

三、来访者的权利与义务

1. 来访者应在预约时间前 10 分钟到达咨询室。
2. 若不能按时来访，应提前在 APP 上取消预约。
3. 迟到说明：
   - 咨询时间不因迟到而延长。
   - 迟到超过 30 分钟或缺席，则本次咨询取消并计入 8 次额度。
   - 一学期内无正当理由取消或迟到累计 3 次，将暂停当学期预约资格。
4. 来访者有权申请更换咨询师，每学期最多一次。若因结案原因无法预约新的咨询师，请拨打 **85463928** 协助处理。
5. 来访者有权在任何阶段中止咨询。

四、咨询师的责任与义务

1. 咨询师因个人原因需取消或更改预约时，应提前告知并重新安排。
2. 咨询师有责任尊重与保护来访者的隐私和个人信息。所有记录将严格保密，仅用于专业目的。
3. 咨询师可能在去除身份信息后，将案例用于中心内部督导或研讨，所有相关人员均须遵守保密原则。
4. 咨询师会在专业能力范围内提供帮助。如遇超出本中心工作范畴的问题，将向来访者说明并推荐合适资源。

五、保密与例外

1. 一般情况下，咨询内容及个人信息均严格保密。
2. 以下情况属于保密例外，咨询师有权利并可能需要突破保密原则：
   - 来访者存在自伤或伤害他人的严重危险；
   - 涉及未成年人遭受虐待或性侵害；
   - 法律法规要求披露（如司法诉讼需要）；
   - 经评估，来访者可能对校园安全或公共秩序造成严重威胁。
3. 在保密例外情况下，咨询师可能联系相关部门或紧急联系人。

六、其他说明

1. 来访者未经允许，不得在咨询过程中进行录音或录像。
2. 咨询记录将妥善保存于中心内部，不会提供给任何机构或个人（包括来访者本人）。
3. 若无可预约时段，可拨打 **85401212** 申请电话咨询。

本人已认真阅读上述内容，知悉并愿意遵守。

来访者姓名：_________

日期：___年__月__日

Document 24 ChatGPT5 modified

学生心理健康指导中心

心理咨询知情同意书

亲爱的同学:

您好! 欢迎来到学校心理中心。心理咨询是心理咨询师与来访者之间建立的一种专业助人关系, 旨在帮助来访者增进自我理解、提升心理健康和应对能力。本中心仅面向本校在籍学生提供心理咨询服务, 不提供精神障碍的诊断、治疗、处方开具、医疗证明及心理治疗等医疗服务。

为保障您的权益, 确保心理咨询顺利开展, 请您仔细阅读以下内容, 并在理解与认可后签字确认。

一、心理咨询设置

1. **预约制度**

    （1）来访同学需本人主动提前预约。若他人代为预约, 除非来访者明确否认, 则视为本人同意。

    （2）咨询结束后, 下一次咨询的预约时间由咨询师与来访者协商确定。

2. **咨询地点**

    所有咨询均在本中心指定的咨询室进行。

3. **咨询时间**

    （1）一般每周一次, 每次 50 分钟。

    （2）每位学生一学期内最多可接受 10 次咨询, 特殊情况需与咨询师协商。

4. **迟到与缺席**

    （1）改期或取消需至少提前 24 小时通知（紧急情况除外）。

    （2）来访者迟到, 咨询时间不予延长; 迟到超过 30 分钟或缺席, 则视为本次咨询取消。

    （3）来访者在未结案前累计无故缺席或迟到达 3 次, 需重新预约等候。

    （4）如咨询师未提前说明而迟到或缺席, 应为来访者补足相应时长或次数。

二、心理中心及咨询师的责任与义务

1. **保密原则**

    （1）咨询师及中心工作人员须严格保密来访者的个人资料和会谈内容。

    （2）为提升服务质量, 去除可识别信息后, 案例资料可用于中心的督导或研讨, 所有参与人员均承担保密义务。

2. **保密例外**

    以下情况, 咨询师可依法或依专业伦理突破保密:

    1）来访者存在伤害自身或他人的严重危险;

    2）未成年人或不具备完全民事行为能力者遭受性侵犯或虐待;

    3）法律法规要求披露的情况。

3. **录音录像**

    原则上, 咨询过程不得录音或录像。若确需用于督导或研究, 须另行征得来访者的书面同意。

三、来访者的权利与义务

1. **权利**

    （1）有权平等对待并受到尊重, 不因性别、民族、信仰等受到歧视。

    （2）有权了解咨询师的资质和咨询方式。

（3）有权选择或在一学期内更换一次咨询师。
（4）有权随时提出中止咨询，但建议在与咨询师充分讨论后协商决定。
（5）有权随时撤回对知情同意书的签署，但已完成的咨询不受影响。

2. **义务**

（1）如实告知个人心理问题及相关经历，包括是否接受过其他机构的评估或治疗。
（2）按时出席咨询，遵守咨询规则，积极配合完成双方商定的任务。

四、转介

若经评估发现来访者的情况超出本中心服务范围或咨询师专业能力，咨询师将解释原因，并协助推荐合适的外部资源或转介渠道。

五、费用

本中心为在校学生提供的心理咨询服务均为免费。

六、紧急与特殊情况

1. 在两次咨询间隔期，如遇紧急情况：

（1）工作时间可拨打办公电话：

- 明向校区：0351-3176212
- 迎西校区：0351-6010260

（2）非工作时间可拨打以下心理援助热线：

- 山西医科大学第一医院 24 小时心理热线：0351-4639459
- 清华大学“珍惜生命”大学生心理热线（16:30—22:30）：4006-525-521

2. 如遇危及自身或他人生命安全的紧急情况，请立即联系 110 或就近医疗机构急诊科。

七、签署确认

我已认真阅读并充分理解以上内容，确认自愿在 XX 大学心理中心接受心理咨询服务。

来访者签字：________ 日期：**年__月__日**

咨询师签字：_____ 日期：___年__月__日

注：此文件一式两份，一份交来访者本人，一份由心理中心留存。

Document 25 ChatGPT5 modified

XX 大学心理学部心理咨询研究与培训中心

心理咨询知情同意书

亲爱的来访同学:
欢迎您来到 XX 大学心理学部心理咨询研究与培训中心（以下简称“本中心”）接受心理咨询服务。本中心的心理咨询由接受过系统专业训练的应用心理临床与咨询方向硕士实习咨询师提供，均在督导教师的指导与监督下开展，服务全程免费。

为确保心理咨询过程科学、规范和有效，现将相关信息说明如下，请您认真阅读并确认:

一、服务说明

1. 服务性质：心理咨询旨在帮助来访者缓解心理困扰、促进自我认识与成长。咨询师不会进行医学诊断、开具药物或医疗证明。

2. 服务对象：本中心主要面向在校大学生提供心理咨询服务。

3. 合作关系：心理咨询是一种专业助人关系，其效果取决于咨询师与来访者的共同努力与配合。

二、保密原则

1. 基本承诺：咨询师将严格遵守保密原则，未经来访者同意，不会向任何第三方泄露来访者的个人信息或咨询内容。咨询记录不会进入学籍档案或职员档案，不影响学业成绩和人际关系。

2. 例外情况（在以下情形中，咨询师有权突破保密原则）:
（1）来访者有自伤、自杀、严重自残或危及他人生命安全的可能;
（2）涉及法律法规要求必须披露的情况;
（3）来访者为未成年人、患有严重精神障碍或丧失民事行为能力时，咨询师需与监护人或相关责任人沟通;
（4）出现危机状况，需通知校方相关部门（如大学生心理健康教育与服务中心），以保障来访者安全与权益。

3. 督导与教学：如需录音、录像或个案讨论，仅限于咨询督导、培训和学术研究使用，且均在严格保密的前提下进行，个人信息将被隐去，所有相关人员均签署保密协议。

三、咨询安排

1. 时间：一般每周一次，每次约 50 分钟；部分特殊情况（如家庭咨询）可延长至不超过 90 分钟。来访者请至少提前 10 分钟到达咨询室或登录线上平台。如迟到，咨询时间不予顺延。

2. 取消或更改：如因故无法按时参加，请至少提前 24 小时通知本中心。若咨询师因工作原因无法按时咨询，会提前告知并重新安排。

3. 心理测评：必要时，咨询师可能建议进行心理测验，以帮助更全面了解来访者情况。
4. 录音录像：在征得来访者知情同意后，咨询可能会被录音或录像，仅用于督导与教学，不作其他用途。
5. 校内学生管理：若来访者为 XX 大学学生，需通过学校心理健康教育与服务中心进行预约备案。若出现危机情况（如严重精神障碍或生命安全风险），相关情况将反馈至学校心理健康教育与服务中心，以便获得进一步支持。

四、转介原则

1. 若来访者问题涉及严重心理障碍或需要医学干预，本中心将建议转介至更合适的专业机构或临床医生。
2. 若咨询师因专业限制无法继续提供合适帮助，经双方协商后，可转介至其他合适的咨询师。

五、来访者的权利与义务

1. 权利：
   （1）有权知情并自主决定是否接受或中止咨询；
   （2）有权在咨询中提出问题，或在必要时申请更换咨询师；
   （3）有权拒绝录音、录像或参加心理测评。
2. 义务：
   （1）如实提供个人情况及相关信息；
   （2）遵守预约规定，准时出席咨询；
   （3）尊重咨询师的专业工作与咨询流程。

六、知情确认

请您确认已仔细阅读并理解以上内容。签字即表明：

- 您自愿接受心理咨询服务；
- 您知晓并同意遵守上述约定；
- 在咨询过程中如有任何疑问，您可随时向咨询师提出并获得解释。

来访者签名：___________
日期：_____年___月___日

XX 大学心理学部心理咨询研究与培训中心

Document 26 ChatGPT5 modified

心理咨询知情同意书

亲爱的来访同学：

您好！感谢您选择本中心的心理咨询服务。为确保咨询顺利进行，维护您和咨询师的权益，现将相关事项告知如下，请您认真阅读并确认理解。

一、心理咨询基本说明

1. **服务性质**
    - 心理咨询旨在帮助来访者缓解困扰、促进自我认识与成长。
    - 咨询不能替代医学诊断与药物治疗。如有精神障碍或其他医学需要，请遵循医嘱并及时就医。
2. **咨询时间与次数**
    - 每次咨询时间约为 50 分钟。
    - 咨询次数由您与咨询师协商确定，双方应共同遵守约定。
3. **资料记录**
    - 为方便咨询服务与后续联系，中心将登记您的真实姓名、联系方式及紧急联系人等信息，仅用于心理咨询管理，不会泄露给无关人员或机构。
4. **录音录像**
    - 若因督导或教学研究需要进行录音、录像，须事先征得您的知情同意。

二、保密原则

1. **一般保密**
    - 咨询师须严格保护您的隐私与咨询内容。您的个人资料及咨询记录不会被随意谈论或外泄。
2. **保密例外**
    - 以下情形下，咨询师有权/有义务在必要范围内披露相关信息：
        1. 您存在自伤或伤害他人的严重危险；
        2. 涉及法律责任或司法调查；
        3. 您为未成年人或缺乏完全民事行为能力，需要告知监护人；
        4. 为督导或专业研讨需要，且所有个人身份信息均已隐去。

三、风险告知与责任说明

1. **自伤/自杀风险**
    - 如您存在自杀想法或行为，请务必提前告知咨询师，以便及时提供支持与必要干预。
    - 咨询师将尽力帮助，但不能保证完全消除自杀冲动及其风险。若您最终选择自伤/自杀行为，相关责任由您个人承担。
2. **严重心理症状**
    - 对于自杀观念、精神分裂、重度抑郁等高风险状况，中心与咨询师有义务进行风险提示和转介建议，但不对由此产生的后果承担法律责任。

四、来访者的权利

1. **自愿原则**
    - 您有权决定是否开始、继续或中止咨询。
    - 您可以选择或更换咨询师。
2. **知情权**
    - 您有权了解咨询师的专业资质、工作方法及咨询安排。

    - 您可要求对不理解的术语或过程进行解释。
3. **平等与尊重**
    - 您将在一个尊重、接纳、无歧视的环境中接受服务。
4. **投诉与反馈**
    - 如您对咨询服务有意见或建议，可通过中心设立的反馈渠道（如主管教师、中心负责人或意见箱）提出。

五、来访者的义务

1. 按约定时间准时到访。如需取消或变更，请提前通知。
2. 以真实、坦诚的态度与咨询师沟通，避免隐瞒重要信息。
3. 对咨询过程积极投入，主动为自身成长和问题解决负责。

六、双方约定

- 本知情同意书一经双方确认，即视为您已充分理解并同意上述内容。
- 如有任何疑问，您可随时向咨询师或中心工作人员提出。

来访者签名：__________　日期：___年__月__日

咨询师签名：__________　日期：___年__月__日

Document 27 ChatGPT5 modified

心理咨询知情同意书

为保障来访学生（以下简称“来访者”）与心理咨询教师（以下简称“咨询师”）双方的权益，明确权利与义务，确保心理咨询的专业性和有效性，请您在咨询前仔细阅读以下内容，并在确认理解与同意后签字。

一、来访者的权利与义务

1. **咨询规范**
   - 来访者应遵守预约制度，准时参加咨询。如需取消或变更时间，请提前告知心理中心。
   - 每次咨询时长约为 50 分钟，通常以每周 1 次为宜。
   - 来访者需凭有效证件登记，以确保咨询工作的安全与规范。
2. **真实沟通**
   - 来访者应如实填写个人信息，真实表达想法与感受。
   - 隐瞒或误导性信息可能影响咨询效果，由此带来的不良后果由来访者本人承担。
3. **对咨询的理解**
   - 心理咨询是一个逐步推进的过程，无法通过一次咨询解决所有问题。
   - 咨询师在过程中扮演倾听、支持和引导的角色，而非替代来访者做决定。
   - 心理测验仅为了解情况的辅助工具，不具有临床诊断效力。
4. **来访者权利**
   - 有权知情咨询流程及咨询师资质。
   - 有权选择或更换咨询师，并可随时提出终止咨询。
   - 在终止或更换过程中，来访者应理解可能对咨询进程产生一定影响。

二、咨询师的权利与义务

1. **严格保密**
   - 咨询师将对来访者的个人信息和咨询内容严格保密。
   - 如需在科研、督导或教学中使用案例，必须隐去一切可识别个人身份的信息，并征得来访者同意。
2. **保密例外**

   在以下情形下，咨询师可不经同意披露相关信息：

   1）来访者存在自伤或伤害他人的现实危险；

   2）来访者疑似严重心理障碍，可能危及自身或他人；

   3）来访者患有可能危及他人生命健康的重大传染性疾病；

   4）来访者遭受严重侵害或处于紧急危险中；

   5）法律法规要求必须披露的情况。
3. **专业职责**
   - 咨询师应遵循心理咨询伦理守则，提供耐心、真诚和专业的帮助。
   - 咨询师仅提供心理咨询服务，不从事精神障碍诊断、药物处方或医疗证明开具等医疗行为。
4. **咨询师权利**
   - 在充分评估来访者情况后，如咨询超出自身专业范围或不适宜继续进行，咨询师有权提出转介或终止咨询。

三、双方共同遵守的约定

1. **咨询目标**
    - 来访者与咨询师应通过讨论，共同设定具体、积极且可行的心理咨询目标。
2. **避免多重关系**
    - 为保障咨询的专业性与边界，双方应避免建立额外的关系（如经济、商业或其他可能影响咨询中立性的关系）。
3. **联系方式与紧急情况**
    - 咨询事务原则上通过中心办公电话或官方渠道联系。私人联系方式（如手机、微信等）不能保证即时回复。
    - 若来访者出现紧急危险（如强烈自伤/自杀意念），请立即拨打 **110/120** 或前往医院急诊，并同时联系心理中心。

本人已阅读并理解上述内容，同意遵守相关规定。

来访者签名：________

日期：___年__月__日

XX 大学学生工作处心理健康教育服务中心

Document 28 ChatGPT5 modified

个体心理咨询知情同意书

亲爱的来访者：

欢迎您来到心理健康教育中心接受心理咨询服务。为了充分尊重和保护您与咨询师的权益，规范咨询过程并确保咨询效果，我们特拟定本《个体心理咨询知情同意书》。请仔细阅读以下内容，并在最后签字确认，表示您已理解并同意相关条款。

一、心理咨询的基本信息

1. 本中心为全校学生免费提供心理咨询服务。
2. 咨询安排：
    - 每次咨询时长约 50 分钟；
    - 一般每周 1 次；
    - 原则上每学期累计不超过 8 次。
3. 咨询地点和时间将由中心与您协商确定：
    - 咨询时间：________；
    - 咨询地点：________。

二、来访者的权利与义务

1. **准时出席**：请您按预约时间准时到达咨询室。如需变更或取消预约，请至少提前一个工作日通知中心。
    - 若连续两次无故缺席，中心将取消后续咨询安排。
    - 若迟到，咨询时间不作顺延，仍按原定时间结束。
2. **自主选择**：您有权在任何时候中止咨询，但建议提前与咨询师沟通，以帮助顺利结束咨询过程。
3. **如实告知**：若您曾在专业医疗机构获得过精神疾病诊断，请如实告知并提供相关材料。若隐瞒病史或症状而导致不良后果，本中心及咨询师不承担责任。
4. **紧急情况**：在两次咨询的间隔期，如遇特殊情况，您可：
    - 工作时间拨打中心电话：85250525；
    - 非工作时间拨打长春市心理援助热线：12320 / 0431-89685000 / 0431-89685333；
    - 全国心理援助热线：800-810-1117（固定电话）或 010-82951332（固定电话及移动电话均可）。

三、咨询师的责任与义务

1. **专业服务**：咨询师将基于专业知识和技能为您提供涉及学习、生活、个人成长及自我发展的心理测评与咨询。
    - 本中心不提供精神鉴定、精神障碍诊断、药物处方、资格证明出具或严重精神障碍的长期心理治疗。若评估您可能存在精神障碍，咨询师会建议您前往专业医疗机构就诊。
2. **保密原则**：
    - 您在咨询中提供的个人信息和资料将被严格保密。
    - 咨询师可能会在学术研究或专业督导中使用部分案例，但会隐去所有可识别个人身份的信息。
    - 以下情况属于保密例外，咨询师可能需要突破保密原则：
        1. 您有明确的自伤或伤害他人风险；
        2. 涉及法律法规要求披露的情形；

3. 发现您存在严重精神障碍，且可能危及自身或他人安全时。

3. **录音录像**：若需要录音或录像（用于督导、教学或研究），须事先征得您书面同意。
4. **职业界限**：咨询师不得接受您的礼物，也不会在咨询室之外与您进行具有咨询性质的交流，以避免双重关系影响。

四、特别提示

1. 心理咨询不能替代药物治疗，如您正在接受精神科药物治疗，请严格遵循医嘱。
2. 咨询的效果取决于双方的共同努力，需要积极配合与持续投入。
3. 如对咨询师的服务或中心安排有疑问或意见，您可向心理健康教育中心提出。

本人已认真阅读并充分理解上述条款，愿意遵守并配合相关安排。

来访者签名：________ 日期：**年__月__日**

咨询师签名：_____ 日期：___年__月__日

Document 29 ChatGPT5 modified

心理咨询知情同意书

亲爱的来访同学:

您好！感谢您信任并选择来到心理健康教育中心。心理咨询是一种专业助人关系，旨在通过咨询师的专业支持，帮助您更好地认识自我、应对困扰、促进成长。为了保障双方的权利与义务，并保证心理咨询顺利开展，请您在咨询开始前仔细阅读以下内容。

一、心理咨询设置

1. **预约与次数**：心理咨询需由来访者本人主动预约。一般情况下每周一次，每次 50 分钟。原则上每位学生每学期最多可接受 8 次咨询。
2. **地点**：所有咨询均在本中心指定的咨询室进行。
3. **适用范围**：本中心仅为本校注册学生提供心理咨询服务，不提供精神障碍的诊断、治疗、处方开具、医疗证明或心理治疗等医疗性质的服务。
4. **咨询过程**：心理咨询是一个渐进的过程，效果取决于来访者与咨询师的共同努力。一次咨询无法解决所有问题，通常需要持续的配合。

二、咨询师的责任与义务

1. **保密原则**
    - 咨询师及中心工作人员须对来访者的个人信息、咨询内容严格保密。
    - 在隐去可识别信息的前提下，案例材料可能用于本中心的督导、研讨或教学，所有参与人员均遵守保密规范。
2. **保密例外**
    在以下情况下，咨询师有义务依法或依伦理突破保密：

1. 来访者存在严重自伤或伤害他人的危险；
2. 未成年人或不具备完全民事行为能力者遭受性侵害或虐待；
3. 法律法规要求披露的情况。

3. **录音录像**
    未经双方明确书面同意，任何一方不得对咨询进行录音录像。如因督导或教学需要使用音视频资料，须另行签署知情同意。
4. **守时原则**
    - 咨询师应遵守约定时间，若需变更将提前 24 小时通知。
    - 如无提前告知，咨询师迟到或缺席时应为来访者补足咨询时间。

三、来访者的权利与义务

1. **如实告知**：来访者应如实提供个人情况，包括既往就诊或评估信息，以便咨询师做出合理判断。若隐瞒信息，相关后果由来访者自行承担。
2. **积极参与**：心理咨询需要来访者的积极配合，包括参与讨论、完成双方约定的任务。
3. **咨询师选择权**：来访者有权提出更换咨询师（每学期最多一次）。
4. **终止权**：来访者可在任何阶段终止咨询，建议在与咨询师充分讨论后共同决定。
5. **改期与取消**：如需改期或取消，请至少提前 24 小时告知中心。若累计 2 次无故缺席或迟到超过 20 分钟，则需重新预约排队。迟到时间不予延长。

四、转介

若经评估发现来访者的情况超出本中心的工作范围或咨询师的专业能力，咨询师会及时说明，并协助推荐合适的资源或机构。

五、费用

本中心为在校学生提供的心理咨询服务均为公益性、免费服务。

六、紧急情况处理

在两次咨询间隔期，如遇心理危机：

- 工作时间：可拨打中心电话 86608122 / 86601122；
- 非工作时间：可拨打心理援助热线
  - 白班：0371-86169595
  - 晚班：0371-22993442

    并请及时联系辅导员或相关支持人员。

七、说明与存档

1. 本协议一式两份，由来访者与本中心各执一份。
2. 来访者有权向咨询师或中心工作人员提出疑问，并获得合理解释。
3. 本协议由 XX 大学心理健康教育中心代为保存。

来访者确认

我已认真阅读并理解以上内容，知晓心理咨询的原则、权利与义务，并自愿在本中心接受心理咨询服务。

来访者签名：________ 日期：___年__月__日

咨询师签名：________ 日期：___年__月__日

Document 30 ChatGPT5 modified

心理咨询知情同意书

亲爱的同学:

您好！欢迎来到重庆第二师范学院心理健康教育与咨询中心。感谢您的信任并选择心理咨询服务。在正式开始咨询前，请您仔细阅读以下内容，以保障您的权益并确保咨询的顺利开展。

一、服务对象

1. 本中心心理咨询服务的对象为本校在读学生。
2. 咨询服务旨在帮助来访学生识别、疏导与调整在学习、生活和人际关系等方面产生的心理困扰。

二、咨询原则

1. **尊重与平等**
    - 中心秉持“尊重、平等、真诚、保密”的服务理念。
    - 您有权了解咨询师的资质、服务内容、咨询时间和方式，并可根据自身情况进行选择。
2. **循序渐进**
    - 心理咨询是一个过程，通常需要一定时间才能见效。一次咨询难以彻底解决所有问题，请您保持耐心与信心。
3. **保密原则**
    - 咨询师将严格保密您的个人信息与咨询内容。
    - 以下情况属于保密例外:
      （1）您存在自伤或伤害他人的危险；
      （2）法律法规要求披露；
      （3）需要转介至其他机构或专业人员以保障您的安全与健康。
4. **自愿与自主**
    - 是否开始或终止咨询完全由您决定。咨询师会提供专业建议，但不会替您做决定。
    - 若您决定中止咨询，由此产生的不良影响需自行承担。
5. **坦诚沟通**
    - 心理咨询是“助人自助”的过程。真诚表达您的真实感受和困惑，将有助于提升咨询效果。
    - 如对咨询方向或方法有疑虑，可随时与咨询师讨论并调整。

三、咨询安排

1. 咨询采取预约制，每次咨询时长为 **40–60 分钟**，一般每周一次。
2. 咨询次数根据个人需求及中心安排确定。
3. 如需取消或变更预约，请至少提前 **24 小时**通知中心，以便合理安排。若未按时到访且未提前告知，可能影响您继续预约咨询。

四、来访者权利与义务

1. 您享有知情权、选择权、更换咨询师的权利。
2. 您有权在咨询中获得尊重与支持，并在需要时提出意见或建议。
3. 您应准时到访，尊重咨询流程，并积极配合咨询工作。

我已认真阅读并理解以上内容，并同意遵守相关约定。

来访者签名：________

日期：___年__月__日

Document 31 ChatGPT5 modified

心理咨询知情同意书

亲爱的来访同学：

您好！感谢您对心理咨询工作的信任。心理咨询是一种专业的助人关系，其目的是帮助您在面对生活中的困扰时获得支持与成长。本中心希望通过知情同意，使您和咨询师在咨询开始前充分了解彼此的权利与义务，以保障心理咨询的顺利与有效开展。

一、心理咨询服务说明

1. **服务对象**：本中心仅向本校正式注册的在读学生提供心理咨询服务，不收取任何费用。
2. **服务性质**：心理咨询不同于医疗服务，不提供精神障碍的诊断、治疗、处方开具、医疗证明或心理治疗等。必要时，咨询师会建议您寻求专业医疗机构的帮助。
3. **咨询设置**：
    - **预约**：原则上由有咨询意愿的同学主动提前预约。
    - **地点**：
        - 花溪校区：学生活动中心 3 楼大学生心理健康与成才服务中心；
        - 两江校区：学生公寓 2 栋 112-114 室。
    - **时间**：每次约 50 分钟，一般为每周一次。原则上每位学生在一学年内最多可接受 8 次咨询。

二、咨询师的责任与义务

1. **保密原则**：
    - 咨询师与中心工作人员必须严格保密，不会将来访者的个人信息及会谈内容透露给无关人员。
    - 为保障服务质量，可能在隐去可识别信息的前提下，用于督导或专业研讨，所有相关人员均承担严格的保密义务。
2. **保密例外**（根据法律法规及伦理守则）：
    - 来访者有自伤或伤害他人的严重危险；
    - 不具备完全民事行为能力的未成年人遭遇性侵犯或虐待；
    - 法律法规明确要求披露的情况（如卫生、司法或公安机关调查）。
      在此类情况下，咨询师有义务采取必要措施（如通知家属或相关部门），并按《重庆理工大学心理危机干预流程》处理。
3. **录音录像**：
    - 除非另行征得来访者的书面同意，否则双方均不得擅自录音录像。
    - 若出于督导或培训目的，需另行签署专项知情同意书。
4. **时间规范**：
    - 咨询师应遵守时间安排，如因个人原因未提前 24 小时告知而缺席，应为来访者补足咨询时间。

三、来访者的权利与义务

1. 来访者应如实告知个人情况（含既往评估或诊断），并积极参与咨询过程，承担因隐瞒信息造成的不良后果。
2. 来访者有权：
    - 提出更换咨询师（每学期最多一次）；
    - 在任何阶段终止咨询，但建议与咨询师讨论后再作决定。
3. 来访者义务：

    - 改期或取消咨询需提前至少 24 小时告知。
    - 若一学期内累计无故迟到或缺席 3 次，需重新预约排队。
    - 迟到不予补足咨询时间；迟到超过 30 分钟视为缺席。

四、转介

- 初始访谈后，若问题超出本中心工作范畴，咨询师将说明情况并推荐合适资源。
- 若议题超出咨询师专业能力，将与来访者协商妥善转介。
- 在与其他机构的接触过程中产生的纠纷由来访者自行承担。

五、费用

- 本中心对在校学生提供免费心理咨询服务。

六、紧急联络与支持

- 工作时间（上午 10:00-12:00，下午 14:00-17:00，晚上 19:00-22:00），可拨打中心电话：62563208。
- 非工作时间遇紧急情况，可拨打重庆市心理援助热线：023-12320-1。

七、紧急联系人

- 来访者需提供一位紧急联系人，并保证信息真实有效。
- 在危及生命安全等紧急情况下，心理中心可能联系该紧急联系人，但不会泄露来访者的具体隐私内容。
- 若提供的联系人信息无效，由此产生的后果由来访者本人承担。

我已阅读并理解以上内容，同意遵守相关约定，自愿参加心理咨询，并对个人行为负责。

来访者签名：__________ 日期：___年__月__日

Document 32 ChatGPT5 modified

学生心理健康教育与咨询中心

心理咨询知情同意书

亲爱的来访同学:

欢迎您来到心理咨询中心。为保障您的合法权益，明确您与咨询师双方的责任与义务，提升咨询质量，请您在咨询开始前仔细阅读并确认以下内容。

一、心理咨询的基本说明

1. 心理咨询是指在良好的咨询关系基础上，由经过专业训练并具备相关资质的心理咨询师，运用心理学的理论与技术，帮助来访者缓解或消除心理困扰，促进心理健康与自我发展的过程。
2. 心理咨询属于非医疗性服务，不提供精神障碍的诊断、药物处方或医疗证明。若发现需要医学干预的情况，咨询师会建议您到相关医疗机构就诊。

二、咨询的设置与流程

1. **预约与准时**：首次来访需携带本人学生证，并按照预约时间准时到达。如因故不能如期到达，请务必提前电话通知中心。
2. **咨询频率与地点**：咨询通常每次约 50 分钟，在中心指定的咨询室内进行。咨询不得在咨询室外进行。
3. **续约与更换**：若需续约，请提前与中心确认。您有权在咨询过程中提出更换咨询师或中止咨询，中心将协助安排新的咨询师或推荐合适的转介服务。
4. **环境要求**：在等待和咨询过程中，请保持安静，并爱护中心的公共设施。
5. **费用说明**：本中心为本校学生提供的心理咨询服务免费。

三、保密与例外情况

1. 咨询师将严格遵守《中国心理学会临床与咨询心理学工作伦理守则（第二版）》中的“保密原则”，您的个人信息和咨询内容不会向无关人员泄露。
2. 为提升咨询质量，咨询过程中可能需要录音或录像，仅用于督导、个案研讨或教学研究。在此之前，中心会征得您的书面同意。
3. 以下情况为保密的例外，咨询师有权利并有义务向相关方披露必要信息：
   （1）您存在自伤或伤害他人的严重危险；
   （2）未成年人或其他无完全民事行为能力者受到性侵犯或虐待；
   （3）法律规定必须披露的其他情况。

四、来访者的权利与义务

1. 您有权在知情的基础上自由选择是否接受或继续咨询，并充分了解咨询的目标、方式和可能的效果。
2. 您应保持积极、开放和诚实的态度，与咨询师共同努力达成咨询目标，并按中心要求填写相关资料。
3. 您有权提出咨询中的意见或疑虑，并可随时申请中止或变更咨询安排。

五、咨询师的责任与承诺

1. 咨询师将遵守职业伦理，竭诚为您提供专业的心理咨询服务。
2. 咨询师将尊重您的个体差异与自主权，不以任何形式歧视、评判或伤害来访者。
3. 咨询师将依据您的情况，必要时建议您寻求医学或其他相关支持。

确认与签署

我已认真阅读并充分理解以上内容，知晓在心理咨询中我所享有的权利及应履行的义务，并自愿参加心理咨询。

来访者签名：_________

日期：____年__月__日

Document 33 ChatGPT5 modified

高校心理咨询知情同意书

亲爱的来访同学：您好！

心理咨询是心理咨询师与来访者之间建立的一种专业助人关系，其目的是帮助来访者更好地认识自我、缓解困扰、促进个人成长。本中心通过知情同意程序，确保来访者与咨询师对咨询过程的权利、义务和相关事项有清晰了解，从而共同维护咨询的顺利开展。

本中心仅向本校正式注册的学生提供心理咨询服务，不提供精神障碍的诊断、治疗、处方开具、医疗证明开具及心理治疗等医疗性质的服务。

请您在咨询前仔细阅读以下内容：

一、心理咨询设置

1. **预约方式**：心理咨询原则上需由来访者本人主动提前预约。
2. **咨询地点**：所有咨询均在本中心指定场所进行。
3. **咨询时间**：一般情况下为每周一次，每次约 50 分钟。原则上每位学生每学期最多可接受 8 次（如中心有特殊规定，则依具体说明）咨询。

二、咨询师的责任与义务

1. **保密原则**
    - 咨询师及中心工作人员须对来访者的个人资料与会谈内容严格保密。
    - 在去除可识别信息后，案例材料可能会用于教学、督导或研究，但所有使用均需遵守专业伦理，确保不泄露来访者身份。
2. **保密例外情况**
   在以下情形中，咨询师有责任依法或依伦理规定披露相关信息：
   （1）来访者有自伤或伤害他人的严重危险；
   （2）不具备完全民事行为能力的未成年人受到性侵犯或虐待；
   （3）法律法规要求披露的其他情况。
3. **录音录像**
    - 咨询过程原则上不进行录音录像。
    - 如因督导或专业发展需要进行录音录像，咨询师需事先取得来访者的书面同意。
4. **守时义务**
    - 咨询师如需取消或变更咨询，应至少提前 24 小时告知来访者，并妥善安排补偿或调整。

三、来访者的权利与义务

1. **如实告知**
   来访者应如实向咨询师提供相关的心理情况，包括在其他机构的评估或诊断结果，以便咨询师全面了解情况并提供适切帮助。
2. **参与义务**
   来访者需积极配合咨询，完成双方商定的作业与探索。若因隐瞒真实情况导致不良后果，由来访者自行承担相应责任。
3. **自主选择**
    - 来访者有权申请更换咨询师（原则上每学期最多一次）。
    - 来访者有权在任何阶段终止咨询，但建议在与咨询师讨论后，经双方协商作出决定。
4. **预约管理**

- 若需取消或改期，来访者应至少提前 24 小时通知中心。
- 如来访者无故迟到或缺席累计 3 次，本阶段咨询将暂停，需重新预约。
- 迟到不延长咨询时间；迟到超过 30 分钟视为缺席。

四、转介说明

1. 初始访谈评估后，中心将根据来访者的情况合理安排咨询。
2. 如来访者的困难超出本中心服务范围，咨询师将向其说明并提供合适的转介资源。
3. 若来访议题超出咨询师的专业能力，咨询师将与来访者协商并妥善安排转介。

五、费用

本中心为在校学生提供心理咨询服务，全程免费。

六、紧急情况处理

1. 咨询间隔期如遇特殊情况，工作时间可拨打中心办公电话：_____。
2. 非工作时间的紧急情况，请拨打当地心理援助热线（号码：_____）。
3. 若涉及人身安全的严重紧急情况，请立即拨打 110 或前往最近的医院急诊科。

七、协议生效与存档

本文件一式两份，中心与来访者各执一份，具有同等效力。

请您在签字前确认已阅读并理解以上内容。如有疑问，可随时向咨询师或中心工作人员提出。

我已充分理解上述内容，并自愿在 _____ 大学心理中心接受心理咨询服务。

来访者签字：________ 日期：**年__月__日**

咨询师签字：____ 日期：___年__月__日

Document 01 Grok4 modified

心理咨询知情同意书

XX 学院大学生心理健康教育与咨询中心是为全校学生提供心理咨询、心理训练和心理健康教育的专业服务机构。我们致力于帮助您实现自我成长、人格完善、潜能开发，并过上健康的生活。为确保心理咨询顺利进行，并维护您的权利与义务以及咨询师的权利与义务，我们对以下事项进行说明。请仔细阅读，并在充分理解后签署。本同意书基于国际（如 APA 伦理准则）和国内（如中国心理卫生协会伦理指南）最新心理咨询伦理与实践标准制定。

1. 服务性质与范围

1.1 本中心是非医疗性质的咨询服务单位，仅对本校在校学生提供免费心理咨询服务。

1.2 我们仅提供涉及个人成长和自我发展类问题的心理咨询，不包括精神鉴定、心理诊断、开具精神类药物、出具资格证明、对严重心理障碍患者的长程心理治疗等医疗相关服务。若您需要此类服务，我们可协助转介至专业医疗机构。

1.3 咨询益处：心理咨询可帮助您更好地理解自己、缓解压力、提升应对能力，但效果因人而异，可能需要时间，且无法保证完全解决问题。

1.4 潜在风险：咨询过程可能引发短暂的情绪不适，如回忆过去创伤。请随时告知咨询师您的感受。

2. 保密原则

2.1 咨询师须对您的咨询内容严格保密，未经您的书面同意，不得泄露给任何第三方。

2.2 例外情况（基于法律和伦理要求，我们必须披露）：

- 2.2.1 您有伤害自身或他人的严重危险时；
- 2.2.2 您有致命传染性疾病且可能危及他人时；
- 2.2.3 未成年人在受到性侵犯或虐待时；
- 2.2.4 法律规定需要披露时。

2.3 若涉及录音/录像或其他记录（如为督导、教学或科研需要），咨询师必须事先征求您的意见，并在获得您的书面同意后方可进行。您有权同意或拒绝，且可随时撤回同意。所有记录将安全存储，并仅用于指定目的。

3. 咨询安排与规则

3.1 每次咨询会谈约 50 分钟，通常每周一次。若有特殊情况，经中心同意，可增加频次。

3.2 心理咨询是一个过程，一次咨询不一定能解决所有问题，通常需要持续而定期的连续咨询。咨询次数由您与咨询师协商决定。

3.3 接受连续咨询的来访者需在每次咨询结束后，直接与咨询师预约下次时间。您需准时到达；若未提前通知而迟到，咨询师将不延长咨询时间。

3.4 若您因故无法按时咨询，请提前一个工作日告知中心改期或取消。咨询师若需改变时间，将提前通知您（不可抗力因素导致迟到除外）。

3.5 在两次咨询间隔期，若遇紧急情况，您无须预约，可直接拨打本班辅导员电话，由辅导员协助处理或预约。

3.6 咨询师资质：所有咨询师均持有相关专业资格，并接受定期督导，以确保服务质量。

4. 您的权利与义务

4.1 您有权接受或停止咨询，也有权决定是否参与咨询过程中的有关活动。停止咨询不会影响您的其他权益。

4.2 您有权寻求其他咨询师的意见或变更咨询师，但需经中心同意。原则上，一段时间内您只能与一位咨询师咨询；若需转介，应先结束当前咨询关系。

4.3 若咨询师基于理论取向或业务学习需要两位及以上咨询师在场，必须事先征得您的同意，

并经中心审核决定。
4.4 在咨询期间，若您欲做出重大决定（如自杀、自伤、伤人、出走、离婚、退学、休学等），请务必告知咨询师协商处理，并签署个人生命安全契约书。否则，将视为咨询关系的自然终止。
4.5 若您或咨询师感觉咨询效果不佳，可协商转介事宜。
4.6 您有权了解咨询过程、提出疑问，并获得解释。若对服务有不满，可向中心主任投诉，我们将公正处理。
4.7 本中心承诺无歧视服务，尊重您的文化背景、性别、年龄等多样性。
5. 同意声明
我已认真阅读并充分理解以上内容，自愿同意接受本中心安排的心理咨询服务。我明白同意是可撤回的，且不会因撤回而受罚。签名：____________________ 日期：___年___月___日

Document 02 Grok4 modified

心理咨询知情同意书
XX 学院大学生心理健康教育与咨询中心致力于为全校学生提供专业心理咨询、心理训练和心理健康教育服务，帮助您实现自我成长、人格完善和潜能开发。以下内容明确了心理咨询过程中咨询师与来访者的权利与义务，以确保咨询顺利进行。请仔细阅读，并在充分理解后签署。本中心为本校正式注册学生提供免费心理咨询服务，非医疗性质，不提供精神障碍诊断、治疗、药物处方、医疗证明或长程心理治疗。如有此类需求，我们将协助转介至专业医疗机构。
1. 服务性质与范围
1.1 服务内容：本中心提供涉及个人成长、情绪调节和自我发展的心理咨询服务，旨在帮助您更好地应对学业、生活和人际关系中的挑战。
1.2 服务限制：根据《中华人民共和国精神卫生法》，本中心不接待精神障碍患者，不提供精神鉴定、心理诊断、药物处方或医疗证明。若评估发现您可能需要此类服务，我们将建议并协助转介至正规医疗机构。
1.3 咨询益处与风险：
益处：心理咨询可能帮助您提升自我认知、缓解压力、改善情绪和应对能力。
风险：咨询过程可能引发短暂情绪不适（如回忆创伤）。请随时告知咨询师您的感受，我们将提供支持。
1.4 服务对象：本中心服务对象为完成登记、签署知情同意书的在校学生及其监护人（如适用）。
2. 咨询安排与规则
2.1 咨询时长与频次：每次咨询约 50 分钟，通常每周 1 次。一学期最多提供 8 次咨询，具体次数由您与咨询师协商确定。
2.2 预约与准时：
您需提前通过中心官网、电话或 APP 预约，并在预约时间准时到达。
若迟到，咨询时间不予延长；若早退，咨询师仅在预约时段内提供服务。
若需取消或更改预约，请至少提前 1 个工作日通知中心，否则可能视为缺诊。
若连续 3 次缺诊（未提前通知），本中心有权终止咨询关系。 2.3 紧急情况：在两次咨询间隔期，若遇紧急情况，可直接联系本班辅导员，由其协助安排咨询或提供支持。 2.4 咨询环境：咨询在中心指定室内进行，确保隐私和安全。未经双方同意，禁止录音、录像或拍照。 2.5 咨询记录：若因教学、科研或督导需要录音/录像，须事先获得您的书面同意。您有权同意或拒绝，且可随时撤回同意。所有记录将安全存储，仅限指定用途。
3. 保密原则
3.1 保密承诺：咨询师及中心工作人员对您的预约信息、咨询内容、测验结果等严格保密，所有资料由专人保管于中心专用档案柜，咨询师不得将记录带出中心或在非专业场合讨论。
3.2 保密例外（基于法律和伦理要求）：
您存在或可能存在危害自身或他人生命安全的风险；
您患有致命传染性疾病且可能危及他人；
未成年人遭受性侵犯或虐待；
法律要求披露（如法院、公安机关出具公函）。
3.3 在例外情况下，咨询师将尽量事先告知您，并征得同意（紧急情况除外）。若需为督导或教学使用案例，将隐去您的身份信息。
3.4 咨询师不得接触其他咨询师的个案记录，确保信息隔离。
4. 咨询师的权利与义务
4.1 提供专业服务：咨询师将竭诚提供高质量服务，态度端正，遵守预约时间，不迟到、早退或无故缺诊。心理咨询效果取决于双方配合，咨询师不保证能完全解决所有问题或达到您的全部预期。
4.2 遵守伦理规范：咨询师严格遵循国家法律法规及《中国心理学会临床与咨询心理学工作伦理守则》，尊

重您的文化背景、性别、年龄等多样性。
4.3 转介责任：若咨询师评估自身不适合为您提供咨询，将在征得您和中心同意后，及时转介至其他咨询师或正规机构。
4.4 应对缺诊：若您迟到或缺诊，咨询师将在预约时段内待在咨询室，研究您的咨询记录，不从事其他活动。
4.5 违约补偿：若咨询师因故需取消或更改预约，须提前 1 个工作日通知您和中心；若未能在规定时间通知，咨询师将为您提供一次免费咨询。
5. 来访者的权利与义务
5.1 您的权利：
有权接受或终止咨询，终止不会影响您的其他权益。
有权了解咨询过程、提出疑问并获得解释。
有权申请更换咨询师或寻求其他专业意见（需经中心同意）。
有权对服务质量提出反馈或向中心主任投诉，我们将公正处理。 5.2 您的义务：
提供真实信息，包括精神健康史（如有）。若因隐瞒或提供虚假信息导致任何后果，您需自行承担责任。
遵守预约制度，准时参加咨询或按规定取消/更改预约。
在咨询期间，若考虑重大决定（如自杀、自伤、伤人、退学、休学等），须提前告知咨询师协商处理，并签署个人生命安全契约书，否则视为咨询关系终止。 5.3 费用与退费（如适用）：
若本中心为收费服务，每次咨询后需缴费方可预约下次时段。
若因规定取消咨询且尚未接受服务，可在缴费后 3 个月内申请退费，需由缴费人本人办理。
若您迟到或未按时取消预约，费用照付（若适用）。
若咨询师迟到，您将获得等同迟到时间的免费咨询服务。
6. 终止咨询关系
6.1 您可随时自愿终止咨询关系，无需理由。
6.2 若您连续 3 次缺诊（未提前通知），或因重大决定未提前告知咨询师，中心有权终止咨询关系。
6.3 终止后，您的咨询记录将按规定保密保存，且不会影响您再次寻求本中心服务。
7. 其他事项
7.1 本中心无提前提醒预约时间的义务，请您自行记录或联系中心确认。
7.2 若咨询师因理论取向或业务学习需多位咨询师在场，须事先征得您的书面同意并经中心批准。
7.3 本中心承诺提供无歧视服务，尊重您的多样性背景。
8. 同意声明
我已认真阅读并充分理解以上内容，自愿接受本中心安排的心理咨询服务。我知晓同意可随时撤回，且不会因撤回而受罚。来访者签名：____________________
日期：___年___月___日咨询师签名（如适用）：____________________
日期：___年___月___日

Document 03 Grok4 modified

心理咨询知情同意书

尊敬的来访者：

为确保心理咨询工作的专业性、有效性及保护您的权益，请您仔细阅读并理解以下内容。本协议旨在明确心理咨询的服务内容、双方责任及相关规定。如有疑问，请随时与咨询师或 XX 大学心理素质教育中心（以下简称“心理中心”）工作人员沟通。阅读并同意后，请在协议末尾签名。

1. 咨询服务概述

1.1 自由选择权：您有权自由决定是否开始、继续或终止心理咨询关系，并可随时了解咨询师的专业资质、理论取向及咨询过程。

1.2 服务对象：本服务面向 XX 大学在校学生，免费提供。

1.3 服务设置：

- 每学期最多提供 8 次咨询（包括面对面及远程网络咨询）。
- 常规频率为每周 1 次，每次 50 分钟。
- 如需调整咨询设置（如频率或时长），可与咨询师协商。

1.4 服务地点：XX 大学心理素质教育中心（7 斋东侧一层）。

2. 保密原则

2.1 保密承诺：心理中心及咨询师对您的个人信息（包括个案记录、测验资料、信件、录音、录像等）严格保密，仅用于专业目的。

2.2 保密例外：以下情况可能需要披露信息：

- 2.2.1 您存在自杀、自伤、伤害他人或其他严重安全风险；
- 2.2.2 未成年人、精神障碍者等弱势群体遭受虐待或忽视；
- 2.2.3 法律法规要求披露的情况。

2.3 信息使用：

- 为提升服务质量，咨询师可能在督导、案例研讨、写作或科研中使用您的案例信息，但须：
  - 事先获得您的书面同意；
  - 隐去可识别您身份的信息。
- 未经双方书面同意，咨询师和您均不得对咨询过程录音或录像。
- 如您未经许可擅自录音录像，咨询师有权立即终止咨询，并保留依法申诉的权利。

3. 预约与取消

3.1 预约流程：

- 预约成功后，请牢记咨询时间并准时到达心理中心接待室，由工作人员引导进入咨询室。
- 迟到不延长咨询时间，未取消预约的缺席计为一次咨询。

3.2 取消或变更：

- 您需至少提前 24 小时通过心理中心电话（62335225）取消或变更预约。
- 24 小时内取消或无故缺席记为“临时未到”。连续 2 次或累计 3 次临时未到，将暂停咨询资格 1 个月，之后可重新预约。
- 咨询师需变更预约时，将提前 24 小时通知心理中心，由心理中心告知您。

4. 咨询关系

4.1 专业关系：咨询师与您共同建立平等、尊重、合作的咨询关系，不发展任何私人关系。

4.2 终止或更换：

- 您可随时终止咨询或更换咨询师，建议事先与当前咨询师沟通，以确保咨询效果。
- 咨询师若评估您的需求超出心理咨询范围（如需医疗干预），将终止咨询并建议转介至相关医院或机构。

4.3 转介与责任：

- 若您拒绝转介或因隐瞒信息导致延误就医，咨询效果可能受限，心理中心及咨询师不承担相关责任。
- 若您经医院评估后需辅助性心理辅导，咨询师可在双方同意下提供支持性辅导。

5. 紧急情况支持

5.1 咨询间隔期支持：

- 工作时间：可拨打心理中心电话（62335225）寻求帮助。
- 非工作时间：可通过“北科心理”公众号（“专业服务”-“转介信息”）获取心理援助热线。

5.2 危机干预：若您出现严重心理危机（如自杀倾向），咨询师将根据保密例外条款采取必要措施以保障您的安全。

6. 权利与义务

6.1 您的权利：

- 了解咨询过程、咨询师资质及服务内容；
- 随时提出疑问或反馈；
- 决定是否同意案例用于督导或研究（见附件授权表）。

6.2 您的义务：

- 提供真实、准确的信息；
- 遵守预约和咨询设置；
- 尊重咨询师及心理中心的专业规范。

7. 协议生效与保管

7.1 本协议一式两份，您和心理中心各持一份，签字后生效。

7.2 请妥善保管本协议，如有遗失可联系心理中心补办。

声明与签名

我已阅读并理解上述心理咨询知情同意书内容，同意遵守相关规定，并自愿接受心理咨询服务。 来访者签名：________________________ 日期：______________

咨询师签名：________________________ 日期：______________ 附件：案例使用授权（请勾选）

☐ 我同意咨询师在符合伦理规范前提下，将我的案例用于督导、研讨或研究（隐去可识别信息）。

☐ 我不同意将我的案例用于上述用途。

Document 04 Grok4 modified

心理咨询知情同意书

亲爱的同学：

欢迎来到学校心理咨询中心！选择心理咨询体现了你对自己心理健康的重视与负责态度。心理咨询是一个合作过程，旨在帮助你更好地认识自我、应对挑战并促进个人成长。为确保你充分了解咨询的过程、权利与责任，请仔细阅读以下内容，并在咨询开始前签署确认。如有疑问，可随时与咨询师沟通。

1. 心理咨询的目标与性质

1.1 咨询目标：心理咨询旨在通过专业方法支持你探索和解决心理困扰，提升心理健康与生活质量。咨询效果依赖于你与咨询师的共同努力。

1.2 咨询范围：咨询内容包括但不限于环境适应、学业压力、情绪管理、人际关系、恋爱与婚姻、性心理、自我发展、家庭关系、职业规划、身心健康及压力调节等。

1.3 咨询性质：心理咨询通过谈话与专业干预促进自我理解和成长，非医疗治疗或药物干预。咨询成果因人而异，无法保证特定效果。

2. 你的权利

2.1 免费咨询：心理咨询服务由学校免费提供，咨询师报酬由学校承担。

2.2 隐私保密：

- 你的个人信息、咨询内容及相关记录将严格保密，仅限专业人员在必要范围内使用。
- 例外情况：根据法律与伦理要求，若咨询内容涉及你或他人的生命安全、严重违法行为或社会安全问题，咨询师有义务向相关部门报告。
- 为提升咨询质量，可能需要对咨询过程进行录音或录像，用于专业督导、教学或研究。录音/录像前，咨询师会征得你的明确同意，隐去可识别信息，并签署保密协议。

2.3 选择与更换咨询师：你有权根据个人需求选择适合的咨询师。若感到当前咨询师不适合，可随时提出更换，中心将协助安排。

2.4 知情同意与风险：

- 咨询收益：通过咨询，你可能缓解心理困扰、提升自我认知并促进个人成长。
- 潜在风险：咨询可能涉及讨论不愉快的经历，引发强烈情绪或暂时不适。保持开放、诚实的态度有助于咨询效果。

2.5 中止咨询：你有权随时提出中止咨询，或因感到不适、受侵犯等原因要求更换咨询师，无需说明理由。

3. 你的责任

3.1 预约与守时：

- 咨询需提前预约，预约时间为工作日 10:00-13:00 或 16:00-19:00。
- 请提前 10 分钟到达咨询室。若无法按时前来，请至少提前 24 小时通知咨询师取消预约。
- 咨询通常每周一次，每次 50 分钟，具体频率可与咨询师协商确定。

3.2 信息登记：首次咨询前，你需填写《信息登记表》，提供基本信息以帮助咨询师了解你的情况。

3.3 反馈参与：咨询结束后一周内，请填写匿名的《咨询反馈问卷》。你的真实反馈将帮助提升咨询质量，反馈内容严格保密，仅用于服务改进。

4. 紧急情况处理

4.1 若你处于心理危机（如严重情绪困扰、自伤倾向等），请立即告知咨询师或联系学工部，中心将优先安排专业支持。

4.2 紧急联系方式：【此处建议补充具体紧急联系电话或方式，如学工部电话或 24 小时热线】。
5. 咨询流程说明
5.1 咨询频率与时长：心理咨询是一个持续过程，通常每周一次，每次 50 分钟，具体安排可与咨询师协商。
5.2 咨询终止：当你与咨询师共同认为目标达成，或你选择结束咨询时，咨询可自然终止。终止前，建议与咨询师进行总结性讨论。
6. 签署确认我已仔细阅读并理解上述内容，对心理咨询的过程、我的权利与责任有充分了解。
我同意按照本知情同意书的内容参与心理咨询。来访者姓名：___________________
签名：___________________
日期：___________________ 咨询师姓名：___________________
签名：___________________
日期

Document 05 Grok4 modified

XX 大学心理服务中心

心理咨询知情同意书

欢迎您选择 XX 大学心理服务中心接受心理咨询服务！为明确双方的权利与义务，保障咨询过程的顺利进行，请您仔细阅读以下内容并签署确认。本协议以电子或纸质形式签署，均具有同等法律效力。

一、协议目的与适用范围

1.1 目的：本协议旨在明确您（来访者）与心理服务中心（以下简称“中心”）在心理咨询过程中的权利、义务及相关规定，确保咨询过程符合伦理与法律要求。

1.2 适用对象：本协议适用于具有完全民事行为能力的成年人。未成年人需由法定监护人代为签署或共同签署。无民事行为能力人不在服务范围。

1.3 服务性质：本咨询服务为北京师范大学临床与咨询心理方向研究生毕业设计项目，公益免费。

二、咨询服务说明

2.1 服务范围：

- 本中心提供心理咨询服务，旨在协助您处理心理困扰、促进个人成长。
- 本中心不提供精神障碍诊断、心理治疗或医疗服务。如咨询师评估发现您可能需要相关服务，将建议您转诊至法定医疗机构。

2.2 咨询效果：

- 心理咨询是来访者与咨询师共同努力的过程，效果取决于双方的配合、问题性质等因素。
- 咨询师将采用基于临床研究、得到专业认可的技术方法，但无法保证完全解决问题。

2.3 咨询师资质：

- 咨询师为应用心理学专业硕士在读研究生，接受过系统化专业培训，并通过考核，在注册心理督导师指导下提供服务。

三、来访者责任

3.1 信息真实性：

- 您需提供真实、准确的个人信息，包括但不限于联系方式、出生年份、紧急联系人、精神健康史、自伤/自杀倾向等。
- 若故意隐瞒或提供虚假信息，您需自行承担由此导致的后果。

3.2 遵守规则：

- 您需遵守中心的咨询设置及相关规定（如预约、取消政策等）。
- 在咨询期间，您应仅与一位咨询师进行咨询工作，若发现同时预约多位咨询师，中心有权暂停或终止服务。

四、保密性与例外

4.1 保密原则：

- 您的个人信息及咨询内容受严格保护，仅限以下人员在必要范围内接触：
  - 您的咨询师；
  - 个案督导师；
  - 论文指导老师及论文答辩委员会成员（仅用于学术和督导目的）。
- 所有接触信息的人员均签署保密协议，严禁对外泄露。
- 咨询相关内容可能用于学术研究（如硕士论文），但将匿名处理，不包含可识别身份的信息。

4.2 保密例外：

- 若咨询师评估认为您存在精神疾病倾向，可能危害自身或他人安全，中心有权联系相关人员或机构（如紧急联系人、医疗机构）以确保安全。
- 若司法机构依法要求配合，中心需提供相关信息。

4.3 知情权：

- 您有权了解咨询相关研究结果，可随时联系咨询师获取信息。

五、录音录像

5.1 目的：为提升咨询质量及督导效果，中心提倡对咨询过程进行录音/录像，仅用于咨询师反思、督导及学术研究。

5.2 管理：

- 录音/录像由咨询师严格保管，遵循保密原则，不作为咨询记录或医疗记录。
- 未经中心书面同意，您不得自行对咨询过程录音或录像。

六、咨询设置

6.1 时间安排：

- 咨询通常每周一次，每次 50 分钟（家庭咨询可为 80 分钟）。填写量表时间不计入咨询时间。
- 请按约定时间准时参加咨询（线上登录或线下到达）。若您迟到，咨询仍按原定时间结束；若咨询师迟到，将补足相应时间。

6.2 预约调整：

- 如需更改预约，请至少提前 24 小时通知咨询师。若在 24 小时内更改，仅可将咨询调整至后天或更晚时间。
- 若连续两次在 24 小时内取消预约或爽约两次，中心将在 3 个月内暂停向您提供咨询服务。

6.3 咨询终止：

- 您或咨询师均有权在任何时间提出终止咨询，但建议与咨询师进行至少一次结束性会谈以妥善处理。

七、网络咨询特别说明

7.1 技术限制：若选择网络咨询，中心无法完全保证网络服务的稳定性、安全性或准确性。

7.2 免责声明：因网络连接、设备故障、不可抗力（如自然灾害、战争等）导致的服务中断或延迟，中心不承担责任。

八、协议变更与终止

8.1 协议变更：

- 中心有权根据需要修改本协议或相关规则，变更内容将通过公示方式公布，公布后即生效。
- 若您不同意变更，可书面通知中心终止协议。

8.2 协议终止：

- 协议在您退出咨询后自动终止。
- 若未成年人未获监护人签署同意，咨询师有权拒绝或终止咨询。

九、签署与生效

9.1 签署要求：

- 您需以真实姓名签署本协议。
- 未成年人需由法定监护人签署或共同签署。
- 多人共同咨询时，所有参与者需签署协议。

9.2 协议保存：

- 您可通过预览模式打印（PC 端）或截图保存（移动端）本协议，请妥善保管。

9.3 生效：本协议自您或监护人签署并提交后生效，纸质与电子形式具有同等法律效力。

十、其他

10.1 联系方式：如有疑问，请联系中心（联系方式：XXX）。

10.2 学术用途补充：

- 咨询过程可能用于教学或研究，相关内容将严格匿名处理，确保您的隐私不受侵犯。

10.3 伦理依据：本协议遵循《中华人民共和国精神卫生法》《中国心理学会临床与咨询心理学工作伦理守则》等法律法规及伦理规范。

签署声明

本人已仔细阅读并理解上述内容，同意遵守本协议的条款，并承诺提供真实信息，配合咨询工作。 来访者签名：________________ 日期：_____________

监护人签名（如适用）：________________ 日期：_____________

Document 06 Grok4 modified

心理咨询知情同意书

尊敬的同学：

欢迎您选择 XX 大学心理健康教育中心提供的心理咨询服务。本同意书旨在帮助您了解心理咨询的服务内容、流程、权利与义务。请仔细阅读，并在充分理解后签署。如有任何疑问，请随时向咨询师提出，我们将耐心解答。

一、心理咨询服务概述

1. 服务目标：心理咨询以“助人自助”为原则，旨在帮助您缓解心理困扰、促进自我成长与发展，适合心理健康的个体或有轻度心理困扰的来访者。
2. 服务方式：由持有专业资质的心理咨询师通过谈话、心理技术等方法，协助您认识并应对生活中的情绪、行为或人际困扰。
3. 服务局限：心理咨询无法替代医学治疗或药物干预。对于严重精神疾病（如精神分裂症、双相情感障碍等），咨询师将建议转介至专业医疗机构。

二、咨询安排

1. 咨询时长与频率：
    - 每次咨询时长约为 50 分钟。
    - 咨询通常每周进行一次，具体频率由您与咨询师协商确定。
2. 预约与取消：
    - 请在每次咨询结束后与咨询师预约下次咨询时间，并准时到达心理咨询室。
    - 如需更改或取消预约，请至少提前 24 小时通过电话（[咨询中心电话]）或[其他联系方式]通知咨询师。
3. 咨询费用：本中心为在校学生提供免费心理咨询服务。如涉及转介或额外服务，可能产生费用，咨询师将提前告知。

三、隐私与保密

1. 保密原则：您的个人信息及咨询内容将严格保密，未经您书面同意，咨询师不得向任何第三方披露。
2. 保密例外：以下情况可能需要部分披露信息，咨询师会在必要时提前与您沟通：
    - 存在伤害自身或他人的严重风险；
    - 未成年人遭受性侵犯、虐待或其他严重侵害；
    - 法律、法规要求披露信息。
3. 记录管理：咨询记录将妥善保存，仅限咨询师及必要时授权的专业人员查阅，用于支持咨询过程或监督评估。

四、来访者权利与义务

1. 您的权利：
    - 随时提出疑问或讨论咨询相关事宜；
    - 在任何阶段选择终止咨询，并可要求咨询师推荐其他专业资源；
    - 了解咨询师的资质及咨询过程的相关信息。
2. 您的义务：
    - 提供真实、完整的个人信息及心理困扰相关信息，以确保咨询效果；
    - 告知咨询师既往心理咨询或精神科治疗经历，以便进行准确评估；
    - 遵守预约时间及咨询安排。

五、咨询的风险与限制

1. 可能的风险：咨询可能涉及讨论敏感话题，可能引发暂时的情绪不适（如焦虑、悲

伤等）。咨询师将尽力提供支持，帮助您应对。

2. 效果限制：心理咨询的效果因个体差异而异，无法保证特定结果。成功依赖于您的积极参与及与咨询师的合作。
3. 转介建议：若您的需求超出本中心服务范围（如需要精神科治疗或长期心理治疗），咨询师将提供转介建议并协助联系相关资源。

六、终止咨询

1. 您有权随时终止咨询，请及时告知咨询师以便进行总结或转介安排。
2. 若咨询师认为您的需求不适合继续在本中心接受服务，将与您充分沟通并提供其他支持方案。

七、疑问与反馈

1. 如对本同意书或咨询过程有任何疑问，请随时向咨询师提出，咨询师将与您充分讨论。
2. 如需进一步帮助，可联系心理健康教育中心，联系方式：[咨询中心电话/邮箱/地址]。
3. 若对服务有意见或投诉，可联系中心负责人：[负责人联系方式]。

八、知情同意声明本人已阅读并理解上述心理咨询服务相关说明，同意接受 XX 大学心理健康教育中心的心理咨询服务，并承诺遵守相关规定。来访者签名：______________________
日期：_______年_______月_______日 咨询师签名（可选，视中心要求）：______________________

日期：_______年_______月_______日

Document 07 Grok4 modified

学生心理发展指导中心
心理咨询知情同意书
引言
欢迎您选择学生心理发展指导中心（以下简称“中心”）的心理咨询服务。本中心致力于为全体在校本科生提供心理健康教育与咨询，帮助您实现自我成长、人格完善、潜能开发和快乐生活。本文件旨在清晰说明心理咨询的过程、您的权利与责任，以及我们的服务承诺。请仔细阅读并在咨询开始前签署确认。如有疑问，欢迎随时与咨询师沟通。
1. 心理咨询服务概述
1.1 服务目标：通过专业支持，帮助您探索个人问题、缓解心理困扰、促进自我成长，适用于学习生活、个人成长和自我发展相关问题。
1.2 服务形式：包括面对面咨询、线上咨询（视频/电话）等，具体形式由您与咨询师协商确定。
1.3 时长与频率：每次咨询约 50 分钟，通常每周 1 次，具体频率根据您的需求和咨询师建议确定。
1.4 服务范围：中心提供心理评估与咨询服务，不提供精神疾病诊断、精神类药物处方、精神鉴定、资格证明或严重心理障碍的长程心理治疗。如需上述服务，建议前往专业医疗机构就诊。
2. 费用与可及性
2.1 免费服务：中心为所有在校本科生提供免费心理咨询服务，不收取任何费用。
2.2 紧急支持：如在咨询间隔期遇到紧急情况，可在工作时间（每日 8:30-11:00、13:30-20:00）拨打中心热线：85099129。非工作时间可拨打以下热线：

- 长春市心理援助热线：12320，0431-89685000，0431-89685333
- 全国心理援助热线：800-810-1117（仅限固定电话），010-82951332（固定电话及移动电话均可）

3. 保密原则与例外
3.1 保密承诺：咨询师对您在咨询中提供的所有信息严格保密，未经您明确同意，不会向任何第三方披露。
3.2 保密例外：在以下情况下，咨询师可能依法或依伦理要求披露信息：

- 3.2.1 您存在伤害自身或他人的严重危险；
- 3.2.2 法律、法规或司法程序要求咨询师通报或披露信息。

3.3 知情披露：如需向相关人员（如家长、校方）披露信息，咨询师将事先征得您的书面同意，并明确披露的内容和范围。
4. 录音与记录
4.1 用途：为提升咨询效果、接受专业督导或用于教学科研，咨询师可能需要对咨询过程进行录音或录像。
4.2 您的权利：录音或录像须事先征得您的明确同意。您有权同意或拒绝，且拒绝不会影响您接受咨询服务。
4.3 管理与销毁：录音或录像仅限专业用途，严格保密，使用后将按规定销毁。
5. 咨询安排与变更
5.1 预约与取消：

- 5.1.1 咨询通常每周 1 次，每次约 50 分钟。如需调整频率，需经中心同意。
- 5.1.2 如需改期或取消预约，请至少提前 24 小时通知中心，否则可能影响后续预约

安排。

5.2 咨询师变更：

- 5.2.1 您有权申请更换咨询师或寻求其他咨询师的意见，需提前向中心提出申请并经批准。
- 5.2.2 原则上，您在同一时期内仅与一位咨询师建立咨询关系。如需更换咨询师，需先终止与当前咨询师的咨询关系。

6. 您的权利与责任

6.1 权利：

- 6.1.1 您有权随时接受或终止咨询服务，无需说明理由。
- 6.1.2 您有权决定是否参与咨询过程中建议的活动（如心理测评、练习等）。
- 6.1.3 您有权了解咨询师的资质、咨询过程及可能的风险。

6.2 责任：

- 6.2.1 为确保咨询效果，请尽量按时参加咨询并积极配合咨询师的建议。
- 6.2.2 如在咨询期间计划做出重大决定（如学业、职业或人际关系相关），建议事先与咨询师讨论，以获得专业支持。

7. 咨询效果与预期

7.1 持续性：心理咨询通常需要多次会谈才能逐步解决问题，单次咨询可能难以达到预期效果。

7.2 个体差异：咨询效果因人而异，取决于您的参与度、问题性质及咨询目标。

7.3 风险提示：咨询可能涉及探讨情绪或敏感话题，可能引发暂时的不适感。咨询师将尽力提供安全支持，帮助您应对。

8. 咨询师资质与伦理

8.1 资质：中心所有咨询师均具备国家认可的心理咨询资质（如国家二级心理咨询师或注册心理师），并定期接受专业督导和培训。

8.2 伦理承诺：咨询师严格遵循中国心理学会及国际心理咨询伦理准则，尊重您的隐私、尊严和自主权。

9. 终止咨询

9.1 终止条件：您可随时提出终止咨询，咨询师也可能在专业判断下建议终止（如目标达成或需转介至其他专业服务）。

9.2 转介服务：如您的需求超出中心服务范围（如严重心理障碍治疗），咨询师将协助您转介至专业医疗机构。

10. 联系与反馈

10.1 联系方式：如有疑问或需进一步支持，请联系中心：

- 电话：85099129（工作时间：8:30-11:00，13:30-20:00）
- 邮箱：[补充中心邮箱，如适用]

10.2 反馈机制：您可随时向中心反馈咨询体验或提出建议，我们将认真对待并持续改进服务。

签署声明

我已仔细阅读并理解本知情同意书的内容，同意接受中心提供的心理咨询服务，并承诺履行相关责任。如有疑问，我已向咨询师或中心咨询并得到解答。学生签名：________________

日期：____________________ 咨询师签名：_______________

日期：____________________

Document 08 Grok4 modified

XX 大学心理健康教育与咨询中心

心理咨询来访者知情同意书

亲爱的同学:

您好!

心理咨询是心理咨询师与来访者之间建立的专业助人关系，旨在通过专业服务帮助您更好地应对心理困扰。本知情同意书旨在清晰说明心理咨询的相关信息、双方的权利与义务，以确保咨询过程顺利开展。请您仔细阅读并确认理解以下内容。

一、心理咨询服务概述

1.1 服务对象：本中心为广东工商职业技术大学正式注册的学生提供免费心理咨询服务。

1.2 服务范围：提供心理咨询与支持服务，不包括精神障碍诊断、药物治疗、医疗证明开具或心理治疗等医疗服务。

1.3 服务目标：通过专业支持，帮助来访者探索心理困扰，提升心理健康水平，促进个人成长。

二、心理咨询设置

2.1 预约方式

需由有咨询意愿的同学主动提前预约。

预约方式：通过本中心指定渠道（如电话、线上系统）办理。

2.2 咨询地点

所有咨询在本中心指定咨询室进行，确保私密性和安全性。

2.3 咨询时间

常规咨询：每周 1 次，每次 50-60 分钟。

特殊情况：如咨询议题复杂，经双方协商可延长至 90 分钟。

预约时间：根据本中心提供的咨询时间表安排。

2.4 咨询形式

主要为面对面咨询，必要时可协商其他形式（如线上咨询，需另签协议）。

三、咨询师的责任与义务

3.1 保密原则

咨询师及中心工作人员对来访者的个人信息和咨询内容严格保密。

为提升服务质量，咨询师可能在隐去可识别身份信息的情况下，将案例用于专业研讨或督导，所有参与人员均遵守保密义务。

3.2 保密例外

在以下情况下，咨询师依法或依伦理要求披露相关信息：

来访者存在严重伤害自身或他人的风险；

未成年人或无完全民事行为能力者遭受性侵犯、虐待等情况；

法律法规要求披露信息。

3.3 录音录像

未经双方同意，咨询师和来访者均不得对咨询过程进行录音或录像。

如需录音录像用于专业培训或督导，咨询师须提前与来访者协商并签署单独的知情同意协议。

3.4 缺席与补时

咨询师如因故需取消或改期，须提前 24 小时通知来访者，并安排补足咨询时间。

来访者因故缺席或改期的规定见 4.3 条款。

四、来访者的权利与义务

4.1 权利

知情权：有权了解咨询过程、目标、方法及可能的局限性。
选择权：有权提出更换咨询师，中心将根据情况协调安排。
终止权：有权随时提出终止咨询，建议事先与咨询师充分讨论以协商终止事宜。
4.2 义务
提供真实信息：向咨询师如实告知心理问题相关情况，包括其他专业机构的评估或诊断结果。
积极参与：与咨询师共同探索解决问题的方法，完成双方商定的咨询任务或作业。
后果自负：因未如实提供信息或未积极配合导致的不良后果，由来访者自行承担。
4.3 预约管理
改期或取消：需至少提前 24 小时通过本中心指定渠道办理。
迟到：迟到不补足咨询时间；迟到超过 30 分钟或缺席，当次咨询取消。
限制：一学期内无明确原因取消预约累计达 3 次，需重新排队等候下一次咨询机会。
五、转介安排
5.1 若经初始评估，来访者的问题超出本中心服务范围（如需精神科治疗），咨询师将向来访者解释并提供合适的求助资源建议。
5.2 若咨询议题超出咨询师的专业能力范围，咨询师将与来访者协商，妥善安排转介至其他专业人士或机构。
六、费用
6.1 本中心对在校学生提供免费心理咨询服务，无需支付任何费用。
七、其他事项
7.1 文件保存：本知情同意书一式一份，由心理健康教育与咨询中心存档。
7.2 咨询效果：心理咨询的效果取决于来访者与咨询师的共同努力及多种因素，中心不对具体咨询效果做出保证。
7.3 疑问解答：如对本协议或咨询过程有任何疑问，可随时向咨询师或中心工作人员咨询。
八、签署声明
我已仔细阅读并充分理解以上内容，同意在 XX 大学心理健康教育与咨询中心接受心理咨询服务，并遵守相关规定。来访者签字：____________________
日期：__________________________ 咨询师签字：____________________
日期：__________________________

Document 09 Grok4 modified

XX 大学心理健康教育与咨询中心

心理咨询知情同意书为确保心理咨询服务的专业性、有效性和安全性，维护来访者与心理咨询师的合法权益，促进咨询过程顺利进行，特制定本知情同意书。请您仔细阅读以下内容，并在充分理解后签署。

一、心理咨询服务概述

1.1 服务性质：XX 大学心理健康教育与咨询中心（以下简称“中心”）为本校在校学生提供非医疗性质的心理咨询服务，旨在支持个人成长与心理健康发展，不收取任何费用。

1.2 服务范围：中心提供与个人成长、情绪调节、人际关系、学业压力等相关的短期心理咨询服务，不提供精神疾病诊断、开具精神类药物、心理鉴定、资格证明或严重心理障碍的长程心理治疗。

1.3 咨询形式与时长：

- 每次咨询会谈约 50 分钟，通常每周一次。
- 根据需要，经中心同意，可调整咨询频次或形式（如线上或线下）。
- 心理咨询是一个持续过程，解决问题的效果需多次会谈，具体次数由来访者与咨询师协商确定。

二、双方权利与义务

2.1 来访者的权利

2.1.1 有权了解心理咨询的过程、目标、方法及潜在风险。

2.1.2 有权选择是否接受咨询、同意或拒绝录音/录像，以及更换咨询师（需经中心协调）。

2.1.3 有权随时提出疑问或终止咨询关系，终止需提前告知咨询师以妥善处理。

2.1.4 有权要求咨询师保护个人隐私，并了解保密原则及例外情况。

2.2 来访者的义务

2.2.1 提供真实、准确的个人信息（包括姓名、联系方式及相关病史），以便中心进行档案管理和后续跟踪服务。

2.2.2 积极配合咨询师的建议，主动参与咨询过程，如需专业精神疾病诊断，需按咨询师要求前往专业医疗机构评估。

2.2.3 若涉及重大决定（如休学、退学、自伤、伤害他人等），须及时告知咨询师协商处理，否则视为咨询关系自然终止。

2.2.4 如有精神疾病相关诊断或病史，须如实告知，不得隐瞒。

2.3 心理咨询师的权利与义务

2.3.1 咨询师有义务为来访者提供专业、尊重和支持的咨询服务，遵循伦理规范，保护来访者隐私。

2.3.2 咨询师有权在专业范围内提出建议或转介建议，若来访者提供虚假信息，咨询师不对因此产生的咨询效果负责。

2.3.3 咨询师有权在专业场合（如督导或个案讨论）隐去来访者身份信息后进行案例讨论，以提升咨询质量。

三、保密原则与例外

3.1 保密原则：

- 心理咨询师对来访者的个人信息、咨询内容及是否接受咨询严格保密，不向中心以外的任何非专业人员或机构透露。
- 所有咨询记录（包括文字、录音/录像）仅用于专业目的，存档于中心并严格管理。

3.2 保密例外：在以下情况下，咨询师可能依法依规披露相关信息：

- 来访者存在自伤或伤害他人的倾向或行为；
- 涉及法律责任或司法程序要求；
- 为保护来访者或公众安全，需向相关机构或人员报告。

3.3 录音/录像：

- 为教学、科研或督导需要，咨询师可能提出录音/录像请求，但须事先取得来访者的书面同意。
- 来访者有权同意或拒绝录音/录像，且拒绝不影响咨询服务质量。

四、咨询关系管理

4.1 避免双重关系：咨询师不得与来访者建立除咨询关系外的其他关系（如私人交往、商业关系等），不得向来访者收取任何费用。

4.2 咨询师更换与转介：

- 来访者有权申请更换咨询师，需经中心协调并结束与当前咨询师的咨询关系。
- 若咨询师认为自身专业能力无法满足来访者需求，可建议转介至其他咨询师或专业机构。

4.3 咨询终止：

- 来访者可随时提出终止咨询，建议提前与咨询师沟通以完成必要总结。
- 若来访者未按约定参与咨询或作出重大决定未告知，视为咨询关系自然终止。

五、其他事项

5.1 信息管理：来访者提供的个人信息仅用于咨询档案管理，严格保密，不向任何无关单位或个人披露。

5.2 风险提示：心理咨询旨在促进心理健康，但不保证解决所有问题，可能因个体差异或配合程度影响效果。

5.3 联系方式：如有疑问或需要进一步信息，可联系中心（联系方式：XXX）。

六、签署声明

本人已认真阅读并充分理解以上内容，同意接受 XX 大学心理健康教育与咨询中心提供的心理咨询服务，并遵守相关规定。来访者签名：________________

日期：______________________

心理咨询师签名：______________

日期：______________________

Document 10 Grok4 modified

心理咨询知情同意书

尊敬的来访者，欢迎您选择心理咨询服务。本协议旨在帮助您了解心理咨询的相关信息、权利与义务。请仔细阅读以下内容，并在充分理解后签署以确认您自愿参与咨询并能够为自己的行为负责。本协议遵循《国际心理咨询伦理准则》及《中国心理学会临床与咨询心理学工作伦理守则（第三版）》等最新伦理与实践指南，确保咨询过程的专业性与规范性。

一、心理咨询基本信息

1. 咨询时长与频率

   1.1 每次咨询时长约为 50 分钟。

   1.2 咨询通常每周进行一次，具体频率可根据来访者需求与咨询师协商确定。

   1.3 为确保咨询效果，建议保持持续且定期的咨询安排。

2. 咨询费用

   2.1 本校心理咨询中心对在校学生提供免费咨询服务。

   2.2 如涉及特殊服务（如心理测评等），将提前告知相关费用并征得同意。

二、咨询中的权利与义务

1. 来访者的权利

   1.1 有权随时提出疑问并获得咨询师的清晰解答。

   1.2 有权在任何时候选择中止咨询，无需说明理由。

   1.3 有权了解咨询过程、方法及咨询师的资质信息。

2. 来访者的义务

   2.1 按时参加预约的咨询，若迟到，咨询时长不予延长。

   2.2 如需取消或更改预约时间，请至少提前 24 小时通知咨询师。

   2.3 未提前通知且未按时参加咨询，视为主动放弃当次咨询，中心将不保留原预约时间，且需重新预约。

3. 咨询师的义务

   3.1 咨询师将以专业态度提供服务，尊重来访者的隐私与自主权。

   3.2 如需取消或更改预约时间，咨询师将至少提前 24 小时通知来访者并协商调整。

   3.3 咨询师将根据需要向来访者提供有关咨询进程的反馈与建议。

三、保密原则

1. 保密承诺

   1.1 来访者在咨询中提供的所有信息将严格保密，未经来访者书面同意，不得向任何第三方披露。

   1.2 咨询记录、录音或录像（如适用）仅用于专业督导或培训，且需事先征得来访者同意。

2. 保密例外情况

   2.1 若存在涉及来访者或他人生命安全的风险（如自杀倾向、伤害他人倾向等），咨询师有义务向相关部门或人员报告，以确保安全。

   2.2 若法律或司法程序要求披露信息，咨询师需依法配合，但将尽可能保护来访者的隐私。

四、咨询记录与督导

1. 为提升咨询质量，咨询师可能在征得来访者书面同意后，对咨询过程进行录音、录像或邀请专业督导观察。
2. 所有记录仅用于专业督导或培训目的，未经来访者同意，不得向第三方披露，且记

录将在咨询结束后按规定销毁或妥善保存。

五、预约与取消政策

1. 预约安排

1.1 长期咨询的来访者需在每次咨询结束后预约下次咨询时间。

1.2 请按约定时间准时到达，迟到将占用原定咨询时长。

2. 取消与更改

2.1 来访者因特殊情况需取消或更改预约，请至少提前 24 小时通知咨询师，协商新的咨询时间。

2.2 咨询师因故需调整时间，将提前 24 小时通知来访者并协商安排。

3. 未按时参加的处理

3.1 未提前通知且未按时参加咨询，视为主动放弃当次咨询，中心将安排其他来访者的咨询。

3.2 若仍需继续咨询，需重新预约。

六、咨询终止

1. 来访者有权随时终止咨询，终止时可与咨询师沟通以确保适当的结束流程。
2. 若咨询师认为继续咨询不再适合（如超出专业能力范围），将提前告知并协助转介至其他合适资源。

七、其他事项

1. 咨询师将根据来访者的需求，采用适合的咨询方法（如认知行为疗法、叙事疗法等），并在咨询开始前向来访者说明。
2. 如有任何疑问或需要进一步信息，可随时向咨询师提出，咨询师将提供清晰解答。

声明与签字

我已仔细阅读并理解上述内容，知晓自己的权利与义务，并自愿参与心理咨询。我明白可随时向咨询师提出疑问以获得进一步解释。来访者姓名：_________

签名：_________

日期：_________ 咨询师姓名：_________

签名：_________

日期：_________

Document 11 Grok4 modified

心理咨询知情同意书

欢迎你选择本校心理咨询服务！为确保你了解心理咨询的过程、权利与责任，请仔细阅读以下内容，并在充分理解后签署，表明你自愿参加咨询并能为自己的行为负责。如有任何疑问，可随时向咨询师提问。

一、心理咨询服务概述

1.1 咨询时长与频率

- 每次心理咨询约 50 分钟，通常每周或每两周进行一次，具体频率由你与咨询师协商确定。
- 为获得最佳效果，建议保持持续、定期的咨询。

1.2 服务对象

- 本咨询服务主要面向本校在读学生，免费提供。

二、保密原则

2.1 信息保密

- 你在咨询中分享的所有信息将严格保密，未经你的书面同意，咨询师不会向任何第三方透露。

2.2 保密例外情况

为保障你及他人的安全，以下情况可能需要打破保密原则：

- 2.2.1 存在严重自杀倾向或自伤风险；
- 2.2.2 存在伤害他人或公共安全的倾向；
- 2.2.3 法律或法院要求披露相关信息；
- 2.2.4 涉及未成年人或无行为能力人的虐待或忽视情况。

  在上述情况下，咨询师将依法采取必要行动，并尽可能提前告知你。

三、预约与取消

3.1 预约规则

- 每次咨询结束后，你需与咨询师预约下次咨询时间。
- 请按时到达预约的咨询地点。未提前通知的迟到将不延长咨询时间。

3.2 取消或变更预约

- 如需取消或变更预约，请至少提前 24 小时通知咨询师。
- 未提前通知且未到场，将视为放弃本次咨询，咨询时间将优先安排给其他来访者。你可重新预约后续咨询。

3.3 咨询师变更通知

- 若咨询师因故需调整咨询时间，将提前通知你并协商新的安排。

四、你的权利与责任

4.1 权利

- 你有权随时提出问题或澄清咨询相关事项。
- 你可随时选择暂停或终止咨询，无需说明理由。
- 你有权了解咨询师的资质、咨询方法及咨询过程。

4.2 责任

- 请积极参与咨询，坦诚分享你的感受与想法，以促进咨询效果。
- 如需调整咨询安排，请尽早与咨询师沟通。

五、咨询风险与局限

5.1 可能的挑战

- 心理咨询可能引发暂时的情绪波动或不适，这是探索内心问题的正常部分。
- 咨询效果因人而异，可能需要一定时间，无法保证立即解决所有问题。

5.2 服务范围

- 本咨询服务旨在提供心理支持，不提供药物治疗或其他医疗服务。如需此类服务，咨询师可协助转介。

六、紧急情况处理

- 若出现心理危机（如强烈自杀念头或紧急情绪问题），请立即联系咨询师或校内心理危机热线（[插入具体联系方式]）。
- 非工作时间，可拨打当地心理危机干预热线（如[插入本地热线号码]）。

七、签署同意我已仔细阅读并理解上述内容，知晓自己的权利与责任，同意自愿参加心理咨询，并可在咨询过程中随时提出疑问。来访者信息

姓名：____________________

性别：____________________

年龄：____________________

班级：____________________

联系电话：_________________ 签名

来访者签名：_______________

日期：____________________ 咨询师信息

姓名：____________________

资质：____________________

日期：____________________

Document 12 Grok4 modified

XX 大学大学生心理健康教育与咨询中心
心理咨询知情同意书
亲爱的同学：
您好！
心理咨询是心理咨询师与来访者之间建立的专业助人关系，旨在通过专业服务支持您更好地应对心理困扰或提升心理健康。本知情同意书旨在明确双方的权利、责任与义务，确保心理咨询过程顺利、透明且符合伦理规范。感谢您选择 XX 大学大学生心理健康教育与咨询中心的服务！
1. 服务概述
1.1 服务性质：本中心为 XX 大学在校学生提供免费心理咨询服务，旨在帮助学生处理情绪困扰、提升心理健康及促进个人成长。
1.2 服务范围与限制：根据《中华人民共和国精神卫生法》（第二十三条），本中心不具备心理或精神疾病的诊断、治疗、处方开具、医疗证明开具或心理治疗资质。如涉及上述情况，本中心将建议您前往符合规定的医疗机构就诊，并可能在必要时（详见 2.2 保密例外）将相关信息告知您所在学院及辅导员，以提供进一步支持。
1.3 知情同意：签署本同意书表示您已了解心理咨询的基本信息、流程及双方责任，并自愿接受本中心提供的心理咨询服务。
2. 心理咨询设置
2.1 预约与时间：
需提前通过电话或本中心指定方式预约咨询。
咨询频率通常为每周一次，每次 50 分钟，具体时间由双方协商确定。
请准时到达，迟到将占用咨询时间，迟到超过 30 分钟视为取消本次咨询，且不予补时。
2.2 取消或更改预约：
如需取消或更改预约，请至少提前 24 小时电话通知本中心。
一学期内无明确原因取消或迟到累计达 3 次，需重新预约并等待排期。
2.3 咨询形式：咨询通常以面对面形式进行，特殊情况下可协商线上咨询，需遵守相同伦理规范。
3. 咨询师的责任与义务
3.1 保密原则：
本中心所有工作人员（包括咨询师、行政人员等）对您的个人信息及咨询内容严格保密。
为提升服务质量，您的案例可能在隐去可识别信息后用于专业督导、教学或案例研讨，所有参与者均遵守保密协议。
3.2 保密例外：在以下情况下，咨询师可能需依法或依伦理要求披露信息：
3.2.1 存在伤害自身、他人或社会安全的风险（如自杀、自伤或暴力倾向）。
3.2.2 发现来访者可能患有严重心理或精神疾病，需转介医疗机构。
3.2.3 来访者涉及致命传染病且可能危及他人安全。
3.2.4 来访者遭受或实施性侵犯、虐待等违法行为。
3.2.5 法律、法规或司法程序要求披露信息。
若涉及上述情况，咨询师将在必要范围内与您沟通，并以保护您及他人安全为首要原则。
3.3 录音录像：
未经双方同意，咨询过程不得录音或录像。
如需录音录像（例如用于专业督导或培训），咨询师将提前与您协商并签署单独的《录音录像知情同意书》。
3.4 专业服务：
咨询师将秉持专业态度，运用心理学知识与技术，提供适合您的支持。

咨询师将根据您的需求，协助制定咨询目标并定期评估进展。
4. 来访者的权利与义务
4.1 权利：
4.1.1 您有权了解咨询过程、目标、方法及可能的风险。
4.1.2 您有权随时提出疑问或表达对咨询的意见，咨询师将认真回应。
4.1.3 您有权在充分讨论后选择终止咨询，并可与咨询师协商终止方式。
4.1.4 您有权了解本中心的服务范围、转介流程及保密政策。
4.2 义务：
4.2.1 心理咨询效果依赖于双方的共同努力。请尽量如实提供您的心理状况、相关经历及医疗信息（如其他机构评估或诊断结果）。
4.2.2 请积极参与咨询过程，配合完成双方商定的目标或任务（如心理作业）。
4.2.3 未如实提供信息或未积极配合可能影响咨询效果，相关后果由您自行承担。
4.2.4 请遵守预约时间及取消/更改预约的规定。
5. 紧急情况处理
5.1 若在咨询间隔期遇到紧急心理危机（如强烈的情绪困扰或自杀意念），请及时寻求帮助：
河北省 24 小时心理援助热线：0312-96312，0311-68052995
北京市 24 小时心理援助热线：010-82951332，010-58303286，010-58340263
5.2 本中心可协助您联系紧急支持资源或转介至专业医疗机构。
5.3 若咨询师评估您存在严重安全风险，可能通知您的紧急联系人或学院辅导员以确保您的安全。
6. 其他事项
6.1 咨询效果：心理咨询的效果因个体差异、问题性质及配合程度而异。本中心不保证特定结果，但将尽力提供专业支持。
6.2 转介服务：若您的需求超出本中心服务范围，咨询师将协助您转介至合适的专业机构，并提供必要的支持信息。
6.3 投诉与反馈：如对咨询服务有任何疑问或不满意，可向本中心负责人反馈，联系方式为[插入中心联系方式]。我们将认真处理您的意见，确保服务质量。
6.4 协议终止：本协议在您完成咨询或正式终止咨询关系后自动失效。终止后，您的个人信息将按相关法规妥善保存或销毁。
7. 声明与签署我已仔细阅读并充分理解本知情同意书的内容，同意遵守相关规定，并自愿接受 XX 大学大学生心理健康教育与咨询中心的心理咨询服务。来访者签名：____________________
日期：___年___月___日 咨询师签名：____________________
日期：___年___月___日

Document 13 Grok4 modified

心理咨询知情同意书亲爱的同学:

您好！欢迎来到本心理咨询中心！感谢您信任我们，与我们分享您的内心世界。我们期待与您共同开启一段探索与成长的旅程。心理咨询的效果取决于您与咨询师的共同努力以及外部环境的影响。为确保咨询顺利进行并保障您的权益，以下是关于心理咨询的重要信息，请仔细阅读并确认。

一、咨询服务说明

1. 服务对象

   本中心为本校学生提供专业心理咨询与治疗服务。心理咨询师将运用专业技术和方法，为您提供个性化的支持与帮助。

2. 收费标准

   本中心为本校学生提供免费心理咨询服务。

3. 咨询时间与安排

   - 每次咨询时长为 50-60 分钟，通常每周 1-2 次。具体频率和时间由您与咨询师协商确定。
   - 请准时参加咨询。如需更改或取消预约，请至少提前 5 小时通知咨询师；咨询师如需调整时间，也会提前通知您。
   - 未按约定取消预约可能影响后续安排，请理解并配合。

4. 保密原则

   保密是心理咨询的核心伦理要求，咨询师将严格保护您的个人信息、咨询记录、测试资料等。未经您明确授权，咨询内容及相关资料（如录音、录像）不会公开或传播。以下情况为保密例外:

   - 4.1 授权披露：经您书面同意，咨询师可按约定范围使用相关信息。
   - 4.2 安全风险：若您有严重自我伤害或伤害他人的倾向，咨询师有义务通知相关部门或您的紧急联系人，但会将信息披露控制在最小范围，并尽可能事先与您沟通。
   - 4.3 法律要求：若涉及法律责任（如犯罪事实、危害公共安全等），咨询师须依法披露相关信息。
   - 4.4 专业督导：为提升咨询质量，咨询师可能在专业督导或案例讨论中提及您的咨询情况，但会隐去可识别身份的信息，并事先征得您同意。
   - 4.5 未成年人：若您未满 18 岁，您的监护人有权申请查阅咨询记录。

5. 记录与资料管理

   - 咨询过程中产生的记录、测试资料等将妥善保存，仅用于咨询目的。
   - 未经您同意，咨询师不会对咨询过程进行录音或录像。如需录音/录像，需提前取得您的书面授权。

二、来访者的权利与责任

1. 权利

   - 自主选择：您有权选择咨询师，或在协商一致后更换咨询师。
   - 自愿参与：您可随时决定暂停或终止咨询，并与咨询师协商调整咨询目标或方法。
   - 知情同意：您有权了解咨询过程、方法及可能的风险，并在充分知情的基础上做出决定。

2. 责任

- 真诚合作：请坦诚提供与心理问题相关的真实信息，积极参与咨询，完成与咨询师协商的作业或练习。
- 主动沟通：积极与咨询师探讨问题，表达需求，共同制定适合您的咨询方案。
- 遵守约定：请准时赴约，遵守预约时间及相关安排。

三、咨询师的权利与责任

1. 权利
    - 咨询师有权了解与您的心理问题相关的必要信息，以提供有效帮助。
    - 若因专业限制或伦理要求（如双重关系、能力范围等），咨询师可提出转介或终止咨询，并向您说明原因并协助转介。
2. 责任
    - 专业性：遵守国家法律法规及《心理咨询职业伦理守则》，以专业态度提供服务。
    - 真诚与尊重：热情接待每位来访者，耐心倾听，尊重您的思想、情感及意愿，建立信任的咨询关系。
    - 保密性：严格遵守保密原则，妥善保管咨询资料，不在咨询室外随意讨论您的信息。
    - 守时性：遵守预约时间，如有特殊情况需调整，提前通知您。
    - 持续成长：定期接受专业督导和培训，以确保咨询质量。

四、其他重要事项

1. 咨询风险与局限性
    - 心理咨询旨在促进自我理解与成长，但效果因个体差异、问题复杂性及外界因素而异，可能无法完全解决所有问题。
    - 咨询过程中可能出现情绪波动或不适，咨询师会尽力协助您应对。
2. 转介与终止
    - 若您的需求超出咨询师的专业范围或本中心服务能力，咨询师会建议转介至更适合的机构或专业人士，并协助完成转介。
    - 您或咨询师均可在适当情况下提出终止咨询，双方应坦诚沟通并妥善处理。
3. 紧急情况
    - 若您在咨询期间出现紧急心理危机（如严重抑郁、自伤倾向等），请立即联系咨询师或拨打校内/校外紧急求助热线（如校医院、当地心理危机干预热线）。
    - 本中心紧急联系方式：【请填写具体联系方式】。

五、知情同意声明我已仔细阅读并理解上述内容，同意与【咨询师姓名】建立专业心理咨询关系。我承诺履行来访者的责任与义务，并知晓自己的权利。我理解咨询的保密原则及例外情况，并同意在咨询过程中配合咨询师共同努力。来访者签名：________________

日期：____________________ 咨询师签名：________________

日期：____________________ 联系方式：

- 来访者：____________________
- 咨询中心：__________________

谢谢您的信任与配合!

如有疑问，请随时与咨询师或本中心联系。祝您在咨询旅程中收获成长与支持!

Document 14 Grok4 modified

心理咨询知情同意书
尊敬的_____同学，您好：
为确保心理咨询过程规范、有效，保障您的权益，以下为心理咨询知情同意书。请仔细阅读并签署。本协议严格遵循《国际心理咨询伦理准则》（如美国心理学会 APA 伦理准则）及《国内心理咨询伦理与实践指南》（如中国心理学会心理咨询伦理规范，2023 修订版），以保护您的隐私与咨询体验。

一、协议目的与双方责任

1.1 本协议旨在规范心理咨询过程，确保咨询服务专业、有效进行。

1.2 协议双方为：

- 来访者：签署本协议的全日制在读高校学生（不含休学学生）。
- 咨询机构：学生心理健康教育与咨询中心（以下简称“中心”）。

1.3 双方承诺共同遵守本协议，维护咨询关系的专业性与信任。

二、咨询服务安排

2.1 咨询地点：学生心理健康教育与咨询中心。

2.2 咨询时长与频次：

- 每次咨询时长为 90 分钟。
- 每周咨询 1 次，每学期默认最多 6 次。

2.3 额外咨询：如需超过 6 次咨询，须经中心专业评估后批准。

2.4 咨询内容：

- 提供学习生活、个人成长、自我发展相关的心理评估与咨询服务。
- 不提供精神疾病诊断、精神类药物处方、司法鉴定、资格证明或严重心理障碍的长程治疗。如需此类服务，建议前往专业精神卫生机构就诊。

三、预约与取消规则

3.1 准时到场：请按预约时间准时到达咨询室，迟到不延长咨询时间。

3.2 取消或变更预约：

- 来访者需提前至少 1 个工作日通知中心，否则计入 6 次咨询限额。
- 咨询师需提前至少 1 个工作日通知中心以变更或取消预约。

3.3 缺席与资格管理：

- 缺席（包括无故缺席、临时请假、提前请假）超过 2 次，将暂停本学期咨询资格，需重新预约排队。
- 无故缺席且失联 48 小时，中心将联系所在院系确保您的安全及学业状态。

3.4 跟诊规则：

- 中心默认安排同一咨询师跟诊，以确保咨询连续性。
- 如不同意跟诊，可提出申请，中心将酌情调整安排，可能延后咨询时间。

3.5 更换咨询师：

- 来访者可在第一次咨询后提出更换咨询师的请求，仅限一次。

四、隐私与保密原则

4.1 保密义务：

- 咨询师对您在咨询中分享的个人信息、经历及感受严格保密，未经您明确同意，不得向第三方披露。
- 咨询记录由中心妥善保存，不提供给任何机构或个人（包括来访者本人）。

4.2 保密例外：

- 若您的行为可能对自己或他人造成严重伤害（如自伤、伤人风险），咨询师有义务向相关部门报告。
- 若涉及司法程序要求提供证供，咨询师可能依法披露相关信息。
- 为学术研究或教学需要，咨询师可能匿名化处理案例信息，确保无法识别您的身份。

4.3 数据管理：

- 咨询记录严格按照相关法规（如《个人信息保护法》）存储与管理，仅用于专业目的。

五、中心职责与限制

5.1 服务范围：

- 中心专注于心理健康教育与短期心理咨询，旨在支持学生应对学业、情绪及人际关系等常见问题。
- 不提供精神疾病诊断、药物治疗或长程心理治疗。

5.2 教学与研究：

- 中心承担临床专业研究生培养任务，可能涉及不定期研究调查或实习生跟诊（匿名化处理）。
- 您的配合将有助于提升服务质量，但您有权拒绝参与研究或跟诊，拒绝不会影响您的咨询权益。

六、来访者权利与义务

6.1 权利：

- 获得专业、尊重和保密的咨询服务。
- 在第一次咨询后提出更换咨询师。
- 随时提出疑问或反馈，中心将及时回应。

6.2 义务：

- 遵守预约时间与咨询规则。
- 提前沟通变更或取消需求。
- 配合咨询师完成必要的评估或反馈。

七、其他事项

7.1 本协议自签署之日起生效，适用于您在本学期的所有咨询活动。

7.2 如有疑问或需进一步解释，请联系中心工作人员（联系方式：中心办公室或官网）。

7.3 本协议内容可能根据法律法规或中心政策更新，中心将提前通知。

声明

本人已仔细阅读并理解上述内容，同意遵守相关规定，并自愿接受心理咨询服务。来访者签名：_______________

日期：____________________

Document 15 Grok4 modified

心理咨询知情同意书

亲爱的同学：

您好！

XX 大学心理健康教育中心（以下简称“本中心”）致力于为在校学生提供免费、非医疗性质的心理咨询服务。本中心不提供精神障碍诊断、治疗、处方开具、医疗证明或心理治疗等服务。为确保您充分了解心理咨询的过程与相关规定，请仔细阅读以下内容，并在充分理解后签署同意书。

一、心理咨询服务概况

1.1 服务性质

本中心提供免费心理咨询服务，旨在帮助学生应对学业、生活、情感等方面的心理困扰，促进个人成长与心理健康。服务不涉及医疗诊断或治疗。

1.2 预约方式

- 心理咨询需由学生主动预约，原则上通过本中心指定方式（如电话、线上系统）提前预约。
- 首次咨询前，中心将安排初始访谈以评估需求并安排后续咨询。

1.3 咨询地点

- 所有咨询在本中心指定地点（教学楼 104 室）进行，确保安全与隐私。
- 非指定地点（如线上咨询）需经中心批准并遵守相关规定。

1.4 咨询时间

- 每次咨询时长为 50 分钟，原则上每周一次。
- 咨询次数由来访者与咨询师协商确定，原则上每位学生每学年最多接受 8 次咨询。
- 心理咨询是一个持续过程，单次咨询可能无法完全解决问题，建议定期参与。

二、咨询师的责任与义务

2.1 保密原则

- 本中心所有工作人员（包括咨询师、行政人员）严格遵守保密原则，确保来访者的个人信息及咨询内容不被泄露。
- 咨询记录仅用于专业目的（如案例分析、督导），并以匿名方式处理，遵守《中华人民共和国个人信息保护法》及相关伦理规范。

2.2 保密例外

在以下情况下，咨询师可能依法或依伦理要求披露部分信息：

- 来访者存在明显自伤、伤人或危害公共安全的风险；
- 法律、法规或司法机关要求披露；
- 涉及未成年人或弱势群体的虐待或忽视情况。

  如需披露，咨询师将尽可能事先告知来访者并限制披露范围。

2.3 专业行为

- 咨询师遵守《中国心理学会临床与咨询心理学工作伦理守则》，以专业态度提供服务。
- 咨询师尊重来访者的文化背景、价值观和个人选择，避免任何形式的歧视或偏见。

2.4 时间管理

- 咨询师需准时开始和结束咨询。如因特殊情况需改期或取消，咨询师将提前 24 小时通知来访者并重新安排时间。
- 若咨询师无法继续提供服务（如离职），本中心将妥善安排转介或过渡。

三、来访者的权利与义务

3.1 权利

- 知情权：来访者有权了解咨询过程、目标、方法及可能的风险。
- 选择权：来访者可申请更换咨询师或寻求其他专业意见，需提前告知本中心并经同意。
- 终止权：来访者可随时终止咨询，建议在咨询中与咨询师充分讨论后再决定。
- 隐私权：来访者的个人信息和咨询内容受法律和伦理保护。

3.2 义务

- 如实告知：来访者需向咨询师提供真实、完整的心理状况信息，包括其他专业机构的评估或诊断结果。
- 积极配合：来访者应积极参与咨询过程，完成与咨询师协商的作业或目标。
- 时间管理：
  - 改期或取消咨询需提前 24 小时通知本中心。
  - 若来访者迟到，咨询时间不予延长；迟到超过 15 分钟或缺席，当次咨询自动取消。
- 重大决定告知：若来访者考虑重大决定（如自杀、自伤、伤人、退学、休学等），需及时告知咨询师共同协商，否则可能导致咨询关系终止。

3.3 后果承担

未如实提供信息或未遵守约定可能影响咨询效果，来访者需自行承担因此产生的不良后果。

四、转介与终止

4.1 转介

- 初始访谈后，若来访者的问题超出本中心服务范围（如需精神科治疗），咨询师将提供解释并推荐合适的专业资源（如医疗机构）。
- 若咨询师认为自身专业能力不足以处理来访者的问题，将与来访者协商后安排转介至其他咨询师或机构。

4.2 咨询终止

- 咨询关系可在双方协商后终止，或因来访者单方决定终止。
- 若来访者连续两次无故缺席或未提前通知取消咨询，视为自动终止咨询关系。
- 本中心保留在特殊情况下（如来访者违反伦理规范）终止咨询的权利，并将提前告知。

五、其他事项

5.1 咨询风险

- 心理咨询可能引发暂时的情绪波动或不适，这是正常现象。咨询师将协助来访者应对。
- 咨询效果因个体差异、配合程度等因素而异，无法保证特定结果。

5.2 录音与记录

- 未经来访者书面同意，咨询过程不得录音或录像。
- 咨询记录仅用于专业用途，存储于安全系统，符合数据保护要求。

5.3 投诉与反馈

- 若对服务有疑问或不满，可向本中心提出反馈（联系方式：XXX）。
- 本中心将依法依规处理，保护来访者权益。

六、签署声明

我已仔细阅读并充分理解上述内容，同意在 XX 大学心理健康教育中心接受心理咨询服务。我了解咨询的性质、权利、义务及可能的风险，并愿意配合咨询师开展工作。

来访者签名：________________ 日期：_______

咨询师签名：________________ 日期：_______

Document 16 Grok4 modified

XX 大学心理健康教育中心
心理咨询知情同意书尊敬的来访者:
您好!
XX 大学心理健康教育中心致力于为心理健康的在校大学生提供专业支持，帮助您应对生活适应、学业科研、人际交往、恋爱情感、家庭关系、人生价值、发展规划、压力情绪等方面的困扰。我们秉持“以人为本，助人自助”的原则，为确保咨询过程透明、规范，维护您与咨询师的权益，特制定本知情同意书。请您仔细阅读并签署。

一、心理咨询服务概述

1.1 服务对象：本中心心理咨询服务面向心理健康的 XX 大学在校大学生。

1.2 服务目标：通过专业咨询，帮助您探索并解决成长中的困扰，促进自我认知与心理健康。

1.3 服务形式：咨询通常为面对面或线上形式（视情况而定），每次咨询时长 50 分钟，频率一般为每周 1 次，具体次数由您与咨询师协商确定。

二、心理咨询的设置

2.1 保密原则

- 咨询师对您的个人信息及咨询内容严格保密，符合中国心理学会及国际心理咨询伦理规范。
- 例外情况（需打破保密原则）:
  2.1.1 您有明显自我伤害或伤害他人的倾向或行为;
  2.1.2 涉及法律责任或司法程序要求披露;
  2.1.3 您因严重心理问题需转介医疗或专业机构。
- 在上述情况下，咨询师将依法依规采取必要措施，并尽可能事先告知您。

2.2 咨询安排

- 本中心为在校学生提供免费心理咨询服务。
- 每次咨询时长为 50 分钟，频率一般为每周 1 次。如需调整频率，需与咨询师协商。
- 如需取消或变更预约，双方应至少提前 12 小时通知心理中心预约人员，由专人告知对方。
- 原则上，您在同一时期只与一名咨询师建立咨询关系。咨询关系结束后，可选择其他咨询师。

2.3 专业督导与记录

- 为提升咨询质量，咨询师可能需接受专业督导。督导过程中，您的个人信息将被匿名化处理，且需事先征得您的同意。
- 如需对咨询过程录音或录像，咨询师将与您另行签订《录音/录像知情同意书》，并确保记录安全存储与保密。

2.4 职业边界

- 为避免双重关系，咨询师不得接受您的礼物或与您建立咨询关系以外的其他关系（如私人社交关系）。
- 若咨询师因个人限制无法继续提供服务，将坦诚告知并协助您转介至其他合适资源。

三、来访者的权利与义务

3.1 权利

- 自愿性：您有权自主选择是否接受咨询、选择咨询师，或随时终止咨询。
- 知情权：您有权了解咨询过程、目标、方法及可能的局限性。
- 更换权：如对咨询师或咨询效果不满意，可申请更换咨询师。

3.2 义务

- 积极参与：以开放、坦诚的态度参与咨询，分享真实感受，配合咨询师共同制定目标。
- 遵守约定：尊重咨询安排，按时参加或提前告知变更。
- 持续努力：心理咨询是一个过程，需投入时间与精力，逐步实现自我成长。
- 及时沟通：如有伤害自己或他人的想法或行为，需及时告知咨询师，以便获得必要支持。

四、咨询师的权利与义务

4.1 权利

- 咨询师有权在专业范围内设定咨询目标与方法，并与您协商一致。
- 若您违反咨询约定（如反复迟到或取消预约），咨询师有权与您讨论是否继续咨询关系。

4.2 义务

- 真诚与专业：以真诚、尊重的态度倾听您的需求，提供专业支持。
- 保密责任：严格遵守保密原则，保护您的隐私。
- 尊重与包容：尊重您的思想、情感与选择，不予评判。
- 持续学习：通过专业培训与督导提升咨询能力，确保服务质量。

五、咨询过程的约定

5.1 目标协商：咨询师将与您共同讨论咨询目标与重点，必要时形成书面协议。

5.2 风险告知：心理咨询可能引发暂时的情绪波动，您有权随时与咨询师讨论相关感受。

5.3 终止咨询：

- 您可随时提出终止咨询，咨询师将与您讨论终止原因并提供必要建议。
- 若咨询师认为您的问题超出其专业范围，将协助转介至其他专业资源。

六、其他事项

6.1 文件保存：本知情同意书由咨询师保存，严格保密，仅用于咨询相关工作。

6.2 疑问解答：如对本协议或咨询过程有任何疑问，请随时向咨询师或心理中心提出。

6.3 联系方式：心理健康教育中心联系方式：（电话/邮箱/预约方式，建议补充具体信息）。

七、签署声明我已仔细阅读并理解本知情同意书的内容，同意遵守相关约定，并自愿参与心理咨询。

来访者签名：____________________

日期：______年______月______日

咨询师签名：____________________

日期：______年______月______日

Document 17 Grok4 modified

心理咨询知情同意书

亲爱的同学，您好！心理咨询是心理咨询师与来访者建立的专业助人关系，旨在通过专业服务帮助您更好地应对心理困扰或提升心理健康。本中心通过本知情同意书向您说明心理咨询的相关信息、双方权利与义务，以确保咨询过程顺利进行。如有疑问，请随时与咨询师或中心工作人员沟通。本中心为 XX 学院正式注册的学生提供免费心理咨询服务，服务内容不包括精神障碍诊断、治疗、处方开具、医疗证明开具或心理治疗等医疗行为。请您仔细阅读以下内容，并在充分理解后签署确认。

一、心理咨询设置

1. 预约方式
    - 心理咨询需由您主动提前预约，预约方式包括但不限于中心官网、电话或现场登记。
    - 首次咨询通常为初始评估，之后根据情况安排后续咨询。
2. 咨询地点
    - 所有咨询在本中心指定地点进行，确保环境安全、隐私。
3. 咨询时间
    - 每次咨询时长为 50 分钟，通常每周一次，具体时间根据中心安排和您的预约确定。
    - 咨询时间以双方约定的时间为准，建议您准时到达。

二、咨询师的责任与义务

1. 保密原则
    - 咨询师及中心工作人员对您的个人信息和咨询内容严格保密，遵守相关法律法规及专业伦理规范。
    - 为提升服务质量，您的案例信息可能在隐去可识别身份信息后，用于专业督导、教学或研究，所有参与人员均承诺保密。
2. 保密例外情况

    以下情况下，咨询师可能依法或依伦理要求披露相关信息：
    - (1) 您存在严重伤害自身或他人的风险；
    - (2) 未成年人或无完全民事行为能力者遭受性侵犯、虐待等情况；
    - (3) 法律、法规或司法程序要求披露；
    - (4) 您书面同意向特定第三方披露。
3. 录音录像
    - 未经双方书面同意，咨询师和来访者均不得对咨询过程进行录音或录像。
    - 如需录音录像（例如用于专业督导或培训），咨询师需提前向您说明并签署单独的知情同意书。
4. 迟到与缺席
    - 咨询师如因故迟到或缺席，将与您协商补足咨询时间或重新安排。
    - 若因不可抗力（如突发公共事件）导致咨询无法进行，中心将及时通知并协调处理。

三、来访者的权利与义务

1. 权利
    - 知情权：您有权了解咨询过程、方法、咨询师资质及可能的咨询效果与风险。
    - 选择权：您可在一学期内申请更换咨询师一次，中心将尽量满足您的需求。

    - 终止权：您有权随时终止咨询，建议在咨询中与咨询师充分讨论后再决定。
    - 隐私权：您的个人信息和咨询内容受保密原则保护（除保密例外情况）。
2. 义务
    - 提供真实、准确的个人信息及心理问题相关情况（如既往诊断、治疗经历等），以便咨询师制定合适的咨询计划。
    - 积极参与咨询过程，与咨询师共同探讨问题解决方案，并按约定完成咨询任务或作业。
    - 未如实提供信息可能影响咨询效果，相关后果由您自行承担。
3. 预约管理
    - 改期或取消：需至少提前 12 小时通过电话或中心指定方式告知。
    - 迟到与缺席：
        - 若您迟到，咨询时间不予延长，剩余时间继续进行。
        - 若迟到超过 30 分钟或缺席，本次咨询取消。
        - 累计迟到或无故取消达 3 次，需重新排队预约。

四、转介与终止

1. 初始评估与转介
    - 首次咨询后，咨询师将根据您的需求和情况安排后续咨询或建议转介。
    - 若您的需求超出本中心服务范围（如需精神科治疗或医疗干预），咨询师将向您解释并提供适合的求助资源或转介建议。
2. 咨询终止
    - 咨询目标达成或双方协商一致时，咨询可终止。
    - 若咨询师认为继续咨询不适合（如超出专业能力范围），将与您协商并妥善安排转介。

五、紧急情况处理

1. 若您在咨询间隔期遇到心理危机或紧急情况，请及时联系本中心：
    - 游仙校区：0816-6905269
    - 安州校区：0816-3963291
    - 服务时间：周一至周五 9:00-12:00，14:30-17:30，19:00-21:00
2. 您也可拨打绵阳市 24 小时公益心理援助热线：
    - 0816-2424666
    - 0816-2268885

六、其他事项

1. 本中心咨询服务遵循《美国心理学会伦理准则》《中国心理学会临床与咨询心理学工作伦理守则》等专业规范，确保服务质量和伦理合规。
2. 若您对咨询过程或服务有疑问、建议或投诉，可联系中心工作人员或通过中心官网反馈。
3. 本同意书内容如有调整，中心将提前告知并征求您的同意。

声明与签署我已仔细阅读并充分理解以上内容，同意在 XX 学院大学生心理健康教育与发展服务中心接受心理咨询服务，并遵守相关约定。

来访者签名：________________ 日期：________________

咨询师签名：________________ 日期：________________

Document 18 Grok4 modified

个体心理咨询知情同意书

尊敬的来访同学:

您好!

心理咨询是一种基于信任的专业助人关系,旨在通过心理咨询师的专业服务支持您探索和解决心理困扰。本中心希望通过本知情同意书，帮助您了解心理咨询的相关信息，明确双方的权利与义务，共同促进咨询过程顺利开展。 本中心为 XX 大学正式注册的学生提供免费心理咨询服务，但不提供精神障碍诊断、治疗、药物处方、医疗证明或心理治疗等服务。如有疑问，请随时与我们沟通。

一、心理咨询设置

1.1 预约方式

- 心理咨询需由您主动提前预约，可通过本中心官网、电话或现场方式完成。
- 预约确认后，请按时参加咨询。

1.2 咨询地点

- 所有咨询在本中心指定地点进行，确保安全与隐私。

1.3 咨询时间

- 通常每周一次，每次咨询时长为 50 分钟，具体时间根据本中心安排确定。
- 如需调整时间，需提前与中心联系。

1.4 咨询形式

- 咨询主要以面对面形式进行，特殊情况下可协商其他形式（如线上咨询，需另签协议）。

二、咨询师的责任与义务

2.1 保密原则

- 咨询师及中心工作人员对您的个人信息和咨询内容严格保密。
- 为提升服务质量，您的案例信息在隐去可识别身份信息后，可能用于专业研讨、督导或培训，所有参与人员均遵守保密义务。

2.2 保密例外

为保障您及他人的安全，以下情况可能需要披露信息:

- 您存在严重伤害自身或他人的风险;
- 未成年人或其他无完全民事行为能力者遭受性侵犯、虐待或其他非法侵害;
- 法律、法规要求披露信息;
- 您明确授权披露特定信息。

2.3 录音录像

- 未经双方同意，咨询师和来访者均不得对咨询过程进行录音或录像。
- 如因专业需要（如教学、督导或质量评估）需录音录像，咨询师将提前与您协商并签署单独的知情同意书。

2.4 迟到与缺席

- 咨询师如因故迟到或缺席，将补足咨询时间或重新安排。
- 如您迟到，咨询时间不予延长;迟到超过 10 分钟或缺席，当次咨询将取消。

三、来访者的权利与义务

3.1 权利

- 您有权了解咨询师的资质、咨询过程及相关安排。
- 您有权提出更换咨询师，中心将尽量满足您的需求。

- 您有权随时终止咨询，建议在咨询中与咨询师充分讨论后协商决定。
- 您有权对咨询过程提出疑问或反馈，中心将及时回应。

3.2 义务

- 请如实告知与心理问题相关的真实情况，包括其他专业机构的评估或诊断结果。
- 请积极参与咨询，配合咨询师共同探索解决问题的方法，完成双方商定的咨询任务。
- 未如实提供信息可能影响咨询效果，相关后果由您自行承担。
- 请按时参加咨询，如需改期或取消，请至少提前 24 小时通知中心。

四、转介与终止

4.1 转介

- 若您的需求超出本中心服务范围（如需精神科治疗或其他专业服务），咨询师将向您解释并提供适合的求助资源或转介安排。
- 若您的咨询议题超出咨询师的专业能力，咨询师将与您协商并妥善安排转介。

4.2 终止

- 咨询师可能因专业判断（如咨询目标达成或不适合继续）建议终止咨询，并与您充分沟通。
- 若您选择终止咨询，请提前告知咨询师或中心。

五、费用

- 本中心为在校学生提供免费心理咨询服务，无需支付任何费用。

六、紧急情况支持

- 咨询间隔期如遇心理紧急情况，请在工作时间拨打中心办公电话：(0471) 4305710。
- 非工作时间可拨打 24 小时心理援助热线：（0471）4306364。
- 若涉及生命安全，请立即拨打 120 或 110 求助。

七、其他事项

- 本中心保留根据实际情况调整服务安排的权利，调整将提前告知您。
- 如您对本协议或其他事宜有疑问，欢迎随时与咨询师或中心工作人员联系。

声明

我已仔细阅读并理解上述内容，自愿在 XX 大学大学生心理辅导与服务中心接受心理咨询服务，并同意遵守本协议约定的内容。 来访者签名： ________________

日期： ________________ 心理咨询中心联系方式：

地址：XX 大学大学生心理辅导与服务中心

电话：（0471）4305710

热线：（0471）4306364（24 小时）

Document 19 Grok4 modified

心理咨询知情同意书

编号：_______亲爱的同学：

您好！

心理咨询是心理咨询师与来访者之间建立的专业助人关系，旨在帮助您通过专业服务获得心理支持。XX 大学大学生心理健康教育中心（以下简称“本中心”）通过本知情同意书，明确双方的权利与义务，确保心理咨询过程顺利进行。本同意书内容遵循《国际心理咨询伦理准则》及《中国心理学会临床与咨询心理学工作伦理守则（第三版）》，以清晰、简洁的语言帮助您了解咨询相关信息。如有疑问，请随时向咨询师提问，我们将提供详尽解释。

一、心理咨询服务概述

1.1 服务对象：本中心为 XX 大学正式注册的在校学生提供免费心理咨询服务。

1.2 服务范围：提供心理咨询与支持服务，不包括精神障碍诊断、治疗、处方开具、医疗证明开具或心理治疗等医疗行为。

1.3 咨询目标：通过专业支持，帮助您探索心理困扰，缓解情绪压力，提升心理健康水平。

二、心理咨询设置

2.1 预约方式

- 需由您主动提前预约，预约方式可通过本中心公布的联系方式（如电话或线上系统）完成。
- 未预约者无法安排咨询。

2.2 咨询地点

- 所有咨询在本中心指定场所进行，确保私密性和安全性。

2.3 咨询时间

- 每周一次，每次 50 分钟，具体时间在中心开放的咨询时段内安排。
- 特殊情况需调整时间，双方应提前协商。

2.4 守时要求

- 来访者：迟到不延长咨询时间，迟到超过 30 分钟或缺席视为取消本次咨询。一学期内无故取消或迟到累计 3 次，需重新预约并排队等候。
- 咨询师：如咨询师因故迟到或缺席，未提前 24 小时通知，将为来访者补足咨询时间。

三、咨询师的责任与义务

3.1 保密原则

- 咨询师及本中心工作人员严格遵守保密义务，确保您的个人信息及咨询内容安全。
- 为提升服务质量，咨询师可能在隐去可识别身份信息后，将案例用于专业研讨或督导，所有参与人员均遵守保密协议。

3.2 保密例外情况

- 您存在伤害自身或他人的严重风险；
- 未成年人或其他无完全民事行为能力者遭受性侵犯或虐待；
- 法律、法规要求披露信息。
- 上述情况下，咨询师将依法采取必要措施，并尽可能提前告知您。

3.3 录音录像

- 未经双方同意，咨询师和来访者均不得对咨询过程进行录音或录像。
- 如需录音录像用于专业培训或督导，咨询师须提前与您协商并签署单独的知情同意书。

四、来访者的权利与义务

4.1 来访者权利

- 知情权：有权了解咨询过程、方法及可能的风险，并可随时向咨询师提问。
- 选择权：有权申请更换咨询师（每学期限 1 次），需提前与中心沟通。
- 终止权：有权随时终止咨询，建议与咨询师充分讨论后协商决定。

4.2 来访者义务

- 真实提供与心理问题相关的个人信息，包括其他专业机构的评估或诊断结果。
- 积极配合咨询师，共同探讨问题解决方案，并按约定完成咨询任务或作业。
- 未如实提供信息可能导致的不良后果，由您自行承担。
- 改期或取消咨询需提前 24 小时通知本中心。

五、转介机制

5.1 若初始评估发现您的需求超出本中心服务范围（如需精神科治疗），咨询师将向您解释并提供适合的求助资源（如精神卫生机构或专业医疗机构）。

5.2 若咨询议题超出咨询师的专业能力，咨询师将与您协商，妥善安排转介至其他适合的咨询师或机构。

六、费用说明

6.1 本中心为山东农业工程学院在校学生提供免费心理咨询服务，无任何额外费用。

七、可能的风险与应对

7.1 心理咨询中的潜在风险

- 心理咨询可能因个体心理状况的复杂性，引发情绪波动或暂时不适。
- 极少数情况下，可能出现的情况
    - 精神病性症状发作；
    - 自杀、自伤或伤害他人等行为。
- 上述情况发生时，咨询师将根据专业伦理采取必要措施（如转介或报告），但不承担因此产生的后果责任。

7.2 紧急情况联系方式

- 工作时间：拨打本中心办公电话 0531-88117870。
- 非工作时间：
    - 全国生命危机干预热线：4001619995（1 学生专线，2 抑郁专线，3 生命专线）；
    - 济南市医学心理咨询中心热线：0531-85713287/85713289。

八、其他说明

8.1 本中心保留根据实际情况调整咨询安排的权利，并提前通知您。

8.2 本同意书内容如有更新，将以书面形式通知您并重新签署。

声明

我已阅读并理解以上内容，对不理解的部分已向咨询师咨询并获得满意解答。我自愿接受 XX 大学大学生心理健康教育中心的心理咨询服务，并同意遵守相关规定。来访者签字：________________ 日期：年___月___日

咨询师签字：_____________ 日期：___年___月___日

Document 20 Grok4 modified

心理咨询知情同意书

欢迎您选择本心理咨询服务！为保障您的权益并明确双方责任，请仔细阅读以下内容，并在充分理解后签署本同意书，表示您知晓并同意相关规定，并能为自身行为负责。

1. 咨询服务概述

1.1 目的：心理咨询旨在通过专业方法协助您应对心理困扰、提升心理健康，但不保证特定结果。咨询效果取决于双方的共同努力。

1.2 形式：咨询通常以面对面、电话或在线形式进行，具体方式由您与咨询师协商确定。

1.3 时长与频率：单次咨询通常为 50 分钟，咨询频率和总次数由您与咨询师根据需求共同商定，双方需严格遵守约定时间。

1.4 非医疗性质：心理咨询不等同于医疗或药物治疗。如您正在接受药物治疗，请严格遵循医生嘱咐，并告知咨询师相关情况。

2. 保密原则

2.1 保密承诺：保密是心理咨询的核心伦理要求。您的个人信息、咨询内容及相关记录将受到严格保护，不会向无关第三方透露。

2.2 保密例外：以下情况可能需要部分披露信息，但仅限于必要范围，并尽可能保护您的隐私：

- 2.2.1 您存在明显自我伤害或伤害他人的风险；
- 2.2.2 涉及法律责任（如法院要求披露信息）；
- 2.2.3 为提升咨询质量，咨询师需在专业场合（如督导或个案讨论）分享匿名化信息，隐去可识别您身份的细节。

2.3 记录管理：您的个人信息（如姓名、联系方式、紧急联系人等）仅用于咨询服务管理，存储于安全系统，不会被泄露或用于其他目的。

3. 录音与录像

3.1 若咨询需录音或录像（如用于培训、督导或质量控制），咨询师将事先征得您的明确书面同意。

3.2 您有权拒绝录音或录像，且拒绝不会影响咨询服务的提供。

4. 双方权利与责任

4.1 来访者权利：

- 4.1.1 随时提出问题或澄清咨询相关事宜；
- 4.1.2 在任何时候选择中止咨询，无需说明理由；
- 4.1.3 获取有关咨询师资质、方法及咨询过程的必要信息。

4.2 来访者责任：

- 4.2.1 提供真实、准确的个人信息及相关背景资料；
- 4.2.2 按时参加咨询，提前告知取消或调整预约；
- 4.2.3 主动告知咨询师有关心理或生理健康的重要信息（如诊断、药物使用等）。

4.3 咨询师责任：

- 4.3.1 提供专业、符合伦理规范的咨询服务；
- 4.3.2 对存在高风险情况（如自杀倾向、严重精神疾病）履行告知义务，可能需联系您的紧急联系人或相关机构；
- 4.3.3 若因您或家人隐瞒关键信息（如诊断或症状）导致后果，咨询师及机构不承担责任。

4.4 机构责任限制：本机构及咨询师仅提供心理咨询服务，不承担超出咨询范围的责任（如

医疗诊断、法律后果等）。
5. 咨询终止
5.1 您可随时终止咨询，建议与咨询师讨论以确保适当的结束流程。
5.2 咨询师可能在以下情况建议终止咨询：

- 5.2.1 您的需求超出咨询师专业能力范围；
- 5.2.2 咨询目标已达成或咨询不再适合；
- 5.2.3 您持续未能履行咨询约定（如多次缺席）。

6. 其他事项
6.1 费用与支付（如适用）：咨询费用（如有）及支付方式将在首次咨询前明确告知，具体标准请咨询工作人员。
6.2 投诉与反馈：如对咨询服务有疑问或不满，可向本机构提出反馈，联系方式为[插入机构联系方式]。
6.3 紧急情况：如遇心理危机（如强烈自杀意念），请立即联系咨询师、紧急联系人或拨打本地心理危机热线[插入热线号码]。
7. 同意声明
我已仔细阅读并理解上述内容，知晓心理咨询的目的、过程、权利与责任。我同意参与心理咨询，并可在咨询过程中随时提出疑问。
来访者签名：________________
日期：___年___月___日
咨询师签名：________________
日期：___年___月___日

Document 21 Grok4 modified

心理咨询知情同意书

亲爱的来访者：感谢您选择本心理咨询中心的服务！我们致力于为您提供专业的心理支持，帮助您提升心理健康水平。为了确保您充分了解心理咨询的过程、权利与责任，请仔细阅读以下内容，并在确认理解后签署本同意书。本同意书旨在保障您的权益，并促进咨询过程的顺利进行。

一、心理咨询的目的与流程

1.1 目的：心理咨询旨在促进心理健康，协助您识别、预防和干预心理困扰，提升生活质量与心理适应能力。

1.2 流程：

- 在咨询助理的指导下完成预约登记、心理测评（如适用）等环节。
- 咨询师将与您共同制定个性化的咨询方案，确保咨询过程符合您的需求。
- 咨询形式包括但不限于个别咨询、团体咨询或线上咨询，具体形式由您与咨询师协商确定。

二、来访者的权利

2.1 自愿参与：心理咨询为自愿行为，您有权随时选择参与或中止咨询。

2.2 选择咨询师：您有权选择适合的咨询师，或在咨询过程中申请更换咨询师。

2.3 自由表达：您有权在咨询中自由讨论任何问题，咨询师将以尊重和接纳的态度倾听。

2.4 知情同意：咨询师将在咨询开始前向您说明咨询目标、方法、时长、可能的风险与效果，并确保您理解。

2.5 中止咨询：您有权在任何时候主动提出中止咨询，并可与咨询师讨论中止的原因与后续安排。

三、来访者的责任

3.1 提供真实信息：为确保咨询效果，您需提供真实、准确的个人信息和相关情况。隐瞒或提供虚假信息可能影响咨询效果，由此产生的后果由您自行承担。

3.2 遵守约定：请按时参加预约的咨询，按约定完成咨询师建议的相关任务或活动。

3.3 主动沟通：如对咨询过程有任何疑问或不适，请及时与咨询师沟通，以便调整咨询方案。

四、咨询师的责任

4.1 专业服务：咨询师将遵循专业伦理准则，以科学、尊重和关怀的态度为您提供服务。

4.2 转介或中止：若咨询师认为您的需求超出其专业能力范围或本中心服务范围，将向您说明并协助转介至更合适的资源或专业人士。

4.3 反馈与沟通：咨询师将定期与您讨论咨询进展，确保咨询目标与您的期望一致。

五、隐私与保密原则

5.1 保密承诺：本中心对您在咨询中提供的所有信息严格保密，仅限咨询师及必要工作人员在专业需要范围内接触相关档案，且均须遵守保密原则。

5.2 保密例外情形：在以下情况下，咨询师可能依法或依伦理要求披露相关信息：

- 5.2.1 您存在严重伤害自身或他人的风险；
- 5.2.2 您患有危及他人的致命传染性疾病；
- 5.2.3 涉及未成年人遭受性侵害、虐待或其他法律规定的必须报告情况；
- 5.2.4 法律、法规或司法程序要求披露信息；
- 5.2.5 您的需求超出心理咨询服务范围，需转介至其他专业机构。

5.3 联系他人：在必要时（如为您的福祉或安全考虑），咨询师可能在征得您同意后（或在紧急情况下依法无需同意）以专业方式联系您的亲属、辅导员或其他相关人员，以为您寻求更

多支持资源。
六、其他事项
6.1 咨询风险：心理咨询可能涉及探讨敏感话题，可能引发暂时的情绪不适。咨询师将尽力协助您应对这些感受，并确保咨询过程安全。
6.2 记录与档案：咨询过程可能涉及记录（如咨询笔记、测评结果），所有记录将妥善保存并严格保密，仅用于专业目的。
6.3 咨询费用（如适用）：如本中心提供收费服务，费用标准将在咨询前明确告知，您有权了解费用详情并选择是否继续。
6.4 投诉与反馈：如对咨询服务有任何意见或不满，您可向本中心反馈，我们将认真处理并保护您的权益。
七、签署声明
我已仔细阅读并理解上述内容，同意接受本中心的心理咨询服务，并承诺履行来访者的责任。我了解咨询的目的、流程、权利、责任及保密原则，并同意在咨询中与咨询师共同合作。
来访者签名：______________________
日期：______________________
咨询中心代表签名：______________________
日期：______________________

Document 22 Grok4 modified

大学生心理健康教育中心

心理咨询知情同意书根据《中华人民共和国心理咨询师职业标准》及相关法规，XX 大学大学生心理健康教育中心心理咨询中心（以下简称“心理中心”）与来访学生本着平等、自愿、协商一致的原则，就心理咨询服务相关事宜达成以下协议。本协议旨在明确双方的权利与义务，确保心理咨询过程的规范性与有效性。

一、咨询服务概述

1. 服务对象：本协议适用于沈阳医学院全体在校学生，心理中心为学生提供免费心理咨询服务，旨在促进个人成长与心理健康。
2. 服务范围：
    - 提供个人成长、情绪管理、人际关系、学习压力等心理议题的咨询服务。
    - 不提供精神疾病诊断、精神鉴定、开具精神类药物或出具资格证明等服务。
3. 咨询形式：主要为面对面个体咨询，必要时可根据实际情况提供线上咨询（需双方协商一致）。
4. 咨询目标：通过专业支持，帮助来访学生提升心理健康水平，解决特定心理困扰或实现个人成长目标。

二、保密原则

1. 保密承诺：
    - 心理中心及咨询师对来访学生的个人信息及咨询内容严格保密，未经书面同意，不向任何第三方披露。
    - 咨询记录仅用于专业目的（如咨询过程记录、督导或科研），并以匿名形式处理。
2. 保密例外：
    - 来访学生存在严重自伤或伤害他人风险；
    - 来访学生涉及致命传染性疾病且可能危及他人；
    - 未成年人遭受性侵犯或虐待；
    - 法律、法规或司法程序要求披露；
    - 咨询师评估认为来访学生可能出现行为失控，影响自身安全、校园安全或教学秩序时，可通知辅导员或紧急联系人。
3. 录音/录像：
    - 为专业督导、教学或科研需要，咨询师可能提出录音/录像请求，须事先取得来访学生书面同意。
    - 来访学生有权同意或拒绝录音/录像，且拒绝不影响咨询服务。

三、咨询安排

1. 咨询时长：
    - 每次咨询时长为 45 分钟，视情况可延长至 50 分钟。
    - 建议每周 1 次，具体频率由来访学生与咨询师协商确定。
2. 预约与取消：
    - 请勿迟到或无故缺席。如需请假或取消咨询，需至少提前 24 小时通过电话（62216906）告知心理中心。
    - 每学期请假次数不超过 2 次；无故缺席 1 次，心理中心有权取消后续咨询安排。
3. 咨询次数：

- 因资源有限，每人每学期常规咨询次数（含请假）不超过 8 次。
- 咨询次数由来访学生与咨询师根据实际需求协商确定。

4. 时间确认：
   - 每次咨询结束前，来访学生需与咨询师确认下次咨询时间后离开。

四、咨询过程

1. 来访学生义务：
   - 提供真实、准确的个人信息及相关资料，以确保咨询效果。
   - 承诺在咨询期间不故意伤害自己或他人，不从事危害人身安全的行为。
2. 咨询师职责：
   - 提供专业、尊重和非评判性的咨询服务。
   - 与来访学生保持职业边界，不建立咨询外的私人联系（如互加微信、QQ、手机号等）。
   - 如需调整咨询时间或讨论额外议题，双方通过心理中心官方电话（62216906）沟通。
3. 特殊情况：
   - 对于正在接受精神科药物治疗的来访学生，若本人及家属希望进行心理咨询，且咨询师评估认为适合，可提供咨询服务。

五、咨询终止与转介

1. 终止条件：
   - 达成咨询目标，双方协商一致终止咨询。
   - 来访学生或咨询师认为咨询效果不佳，可协商终止或转介至其他专业机构。
2. 来访学生权利：
   - 有权随时决定接受或终止咨询。
   - 有权选择是否参与咨询中的特定活动（如心理测评、练习等）。
3. 转介安排：
   - 若心理中心无法满足来访学生需求（如需精神科治疗），咨询师将协助推荐至校外专业机构。

六、其他事项

1. 联系方式：
   - 心理中心联系电话：62216906。
   - 来访学生需提供紧急联系人信息，以便在紧急情况下联系。
2. 服务费用：
   - 心理中心为沈阳医学院学生提供免费心理咨询服务。
3. 未尽事宜：
   - 双方可协商补充协议条款，补充条款与本协议具有同等效力。

七、协议签署

本协议经双方签署后生效。来访学生签署本协议，即表示已充分理解并同意协议内容。

心理咨询师：________________

来访学生：__________________

联系电话：__________________

紧急联系人：________________

紧急联系人电话：_____________

签署日期：___年___月___日

Document 23 Grok4 modified

心理咨询知情同意书

亲爱的同学，您好：感谢您选择本中心心理咨询服务！心理咨询是咨询师与来访者之间建立的一种专业助人关系，旨在通过专业服务支持您更好地面对心理困扰或个人成长需求。为保障您的权益并确保咨询过程顺利进行，请您仔细阅读以下内容，并在充分理解后确认同意。本协议基于国际与国内最新心理咨询伦理与实践指南（如《美国心理学会伦理准则》《中国心理学会临床与咨询心理学工作伦理守则》）制定，语言简洁、结构清晰，适合高校学生群体理解。

一、咨询服务概述

1.1 服务对象：本中心向本校全日制在读学生（不含休学学生）提供免费心理咨询服务。

1.2 服务范围：提供心理支持与辅导，不包括精神障碍诊断、治疗、药物处方、医疗证明开具或心理治疗等医疗服务。

1.3 服务形式：

- 咨询地点：学校三个校区心理咨询室。
- 咨询频率：原则上每周 1 次，每次 50 分钟。
- 每学期最多提供 8 次咨询，同一时段不可重复预约。特殊情况需经专业评估后方可延长咨询次数。

二、预约与取消规则

2.1 准时到场：请提前 10 分钟到达咨询室。

2.2 迟到与缺席：

- 迟到不延长咨询时间；迟到超过 30 分钟或缺席，视为取消本次咨询，计入 8 次咨询限额。
- 一学期内无明确理由取消或迟到达 3 次，将暂停本学期预约资格。

2.3 取消预约：如无法按时到场，请提前通过 APP 取消预约。

2.4 咨询师取消：如咨询师因故需取消或更改预约时间，将提前通知您并协商新的咨询时间。

三、咨询师与来访者权利与义务

3.1 来访者权利：

- 有权申请更换咨询师，每学期限 1 次。
- 有权了解咨询过程、目标及可能的局限性。
- 有权随时终止咨询并获取转介资源（如适用）。

3.2 咨询师义务：

- 尊重您的隐私，遵守专业伦理规范，确保咨询过程安全与专业。
- 若您的情况超出本中心服务范围，咨询师将解释原因并提供合适的求助资源（如校外专业机构）。

3.3 更换咨询师流程：若因系统显示咨询师未结案导致无法预约新咨询师，请拨打 85463928 联系本中心处理。

3.4 紧急求助：若无可用预约时段，可拨打 85401212 进行电话咨询。

四、隐私与保密原则

4.1 保密承诺：

- 您的个人信息及咨询内容严格保密，仅在中心内部以加密形式保存。
- 未经您同意，咨询记录不会提供给任何个人或机构（包括您本人）。

4.2 保密例外：以下情况可能突破保密原则：

- 您的行为可能对自己或他人造成严重伤害（如自伤、伤害他人）。

- 司法机关依法要求提供咨询记录作为呈堂证供。
- 在上述情况下，咨询师将向相关单位报告必要信息，并尽可能保护您的隐私。

4.3 案例使用：为提升服务质量，您的案例在隐去可识别身份信息后，可能用于中心内部研讨或专业督导。所有参与人员均遵守严格保密协议。

4.4 录音录像限制：未经咨询师同意，咨询过程中禁止录音、录像或以其他方式记录。

五、其他重要事项

5.1 服务局限性：本中心提供心理支持而非医疗服务。若您的情况需专业医疗干预，咨询师将协助转介至校外医疗机构。

5.2 反馈与投诉：如对咨询服务有任何疑问或建议，请拨打 85463928 联系本中心。

5.3 知情同意：签署本协议表示您已充分理解并同意遵守上述条款。咨询师也承诺遵守专业伦理规范，为您提供安全、专业的服务。

六、签署确认

我已仔细阅读并理解本知情同意书的内容，同意遵守相关规定，并自愿接受本中心的心理咨询服务。

姓名：____________________

日期：____________________

签名：____________________

Document 26 Grok4 modified

XX 大学学生心理健康指导中心

心理咨询知情同意书

亲爱的同学：欢迎您选择 XX 大学学生心理健康指导中心（以下简称“本中心”）的心理咨询服务。本中心致力于为在校学生提供专业的心理支持，帮助您更好地应对学业、生活及个人成长中的挑战。心理咨询是咨询师与来访者共同努力的过程，旨在促进您的心理健康与个人发展。为保障您的权益并确保咨询过程顺利有效，请您仔细阅读以下内容，充分了解心理咨询的相关信息。如无异议，请在文末签字确认。本同意书一式两份，您与本中心各保留一份。

一、心理咨询服务概述

1. 服务性质

   心理咨询是心理咨询师与来访者之间建立的专业助人关系，旨在通过专业服务促进来访者的心理健康与问题解决。本中心为具有本校学籍的学生提供心理咨询服务，但不提供精神障碍诊断、治疗、药物处方、医疗证明或心理治疗等服务。

2. 服务目标

   通过与咨询师的合作，帮助您探索和解决心理困扰，提升心理韧性与自我成长能力。

二、心理咨询设置

1. 预约方式
   - 心理咨询需由您本人主动提前预约，预约方式包括但不限于线上系统、电话或现场登记。
   - 若由他人代为预约，未经您明确拒绝，视为您同意预约。
   - 本中心鼓励您尽早预约以确保咨询安排顺利。
2. 咨询地点
   - 所有咨询均在本中心指定场所进行，具体地点将在预约时告知。
3. 咨询时间
   - 首次咨询时间由本中心安排，后续咨询时间由您与咨询师协商确定。
   - 咨询频率一般为每周 1 次，每次 50 分钟。
   - 每位学生每学期最多可接受 10 次咨询，特殊情况需与咨询师协商并经本中心批准。
4. 迟到与缺席
   - 双方均应准时参加咨询。如需改期或取消，需至少提前 24 小时通知本中心（紧急情况除外）。
   - 若咨询师未提前告知迟到或缺席，将为您补足咨询次数或时长。
   - 若您迟到，咨询时长不予延长；迟到超过 30 分钟或缺席，本次咨询取消。
   - 未结案期间，若您无明确理由取消或迟到累计达 3 次，需重新预约并等待安排。

三、本中心的责任与义务

1. 保密原则
   - 本中心所有工作人员（包括咨询师、行政人员等）对您的个人信息及咨询内容严格保密。
   - 为提升服务质量，您的案例信息可能在隐去可识别身份信息后用于本中心的专业研讨或督导，所有参与者均承诺遵守保密义务。
   - 所有案例使用均严格遵循心理咨询伦理规范。
2. 保密例外

以下情况本中心可能依法披露相关信息：

- 您存在伤害自身或他人的严重风险；
- 未成年人或不具备完全民事行为能力者遭受性侵犯、虐待或其他非法侵害；
- 法律法规或司法机关要求披露。

若发生上述情况，本中心将在必要范围内披露信息，并尽可能提前告知您。

3. 录音录像
   - 未经双方同意，咨询过程中禁止录音或录像。
   - 为专业培训或质量监督需要，咨询师可能申请录音或录像，但需与您另行签订书面知情同意书，并明确用途及保密措施。

四、来访者的权利与义务

1. 权利
   - 知情权：您有权了解咨询过程、方法、咨询师资质及可能的咨询效果。
   - 选择权：您可申请更换咨询师，每学期最多 1 次，需向本中心提出书面申请。
   - 终止权：您可随时终止咨询，建议事先与咨询师充分讨论以达成共识。
   - 反馈权：您有权对咨询服务提出意见或投诉，本中心将认真处理。
2. 义务
   - 向咨询师如实提供与心理问题相关的真实信息，包括其他专业机构的评估或诊断结果。未如实告知可能影响咨询效果，您需自行承担相关后果。
   - 积极参与咨询过程，与咨询师共同制定目标并完成约定的任务（如心理作业）。
   - 遵守咨询设置，准时出席，尊重咨询师及本中心工作人员。

五、转介与危机干预

1. 转介
   - 若您的咨询需求超出本中心服务范围（如需精神科治疗或其他专业服务），咨询师将向您解释并推荐合适的求助资源。
   - 若您的议题超出咨询师的专业能力，咨询师将与您协商并妥善安排转介事宜。
2. 危机干预
   - 若您在咨询期间或间隔期遇到紧急心理危机，可通过以下方式求助：
     - 工作时间：拨打本中心办公电话：
       - 明向校区：0351-3176212
       - 迎西校区：0351-6010260
     - 非工作时间：拨打心理援助热线：
       - 山西医科大学第一医院 24 小时心理热线：0351-4639459
       - 清华大学珍惜生命大学生心理热线：4006-525-521（16:30–22:30）
   - 本中心将协助您获得及时支持。

六、费用

- 本中心为在校学生提供免费心理咨询服务，无需支付任何费用。

七、其他事项

1. 咨询效果
   - 心理咨询的效果取决于您与咨询师的配合、问题的复杂性及外部支持等多种因素。
   - 本中心致力于提供专业支持，但无法保证特定结果。
2. 咨询师资质
   - 本中心所有咨询师均具备心理学相关专业背景，并接受过系统的心理咨询培

训，符合国家相关资质要求。

- 您有权了解咨询师的资质信息，可向本中心查询。

3. 投诉与反馈
    - 若您对咨询服务有任何疑问或不满，可联系本中心行政办公室（联系方式同上）。我们将认真倾听并妥善处理。

八、知情同意声明

我已仔细阅读并充分理解上述内容，知晓心理咨询的过程、权利、义务及相关设置。我自愿接受 XX 大学学生心理健康指导中心的心理咨询服务，并同意遵守本同意书的相关规定。

来访者签名：________________

咨询师签名：________________

日期：_____________________

Document 25 Grok4 modified

XX 大学心理学部心理咨询研究与培训中心
心理咨询知情同意书
欢迎您选择 XX 大学心理学部心理咨询研究与培训中心（以下简称“本中心”）的心理咨询服务。本中心由接受过系统专业培训的应用心理学临床与咨询方向专业硕士担任实习咨询师，在资深督导指导下为您提供免费、专业的心理咨询服务。为确保咨询过程科学、规范、有效，请您仔细阅读以下内容，并确认理解和同意。
1. 服务概述
1.1 服务性质：本中心提供心理咨询服务，旨在帮助您处理情绪、心理或人际关系等方面的问题，促进心理健康与个人成长。
1.2 服务提供者：咨询由接受系统培训的实习咨询师提供，所有咨询过程均在专业督导指导下进行。
1.3 服务对象：本服务主要面向 XX 大学在校学生及其他符合条件的来访者。
2. 保密原则
2.1 保密承诺：本中心严格遵守保密原则，未经您明确书面同意，您的个人信息及咨询内容不会向任何第三方披露，也不会记录于学籍档案、职员档案或影响您的学业与社交关系。
2.2 保密例外：以下情况下，本中心可能需要突破保密原则，以确保您或他人的安全及符合法律法规：
2.2.1 您存在自伤、自残、自杀等威胁自身生命安全的行为，或可能对他人生命安全构成威胁；
2.2.2 涉及法律法规要求（如法院传唤、涉嫌违法行为等）；
2.2.3 您为未成年人、患有严重精神障碍或丧失民事行为能力时，本中心可能需与您的监护人或家属沟通。
2.3 保密措施：在咨询师与督导讨论案例或进行团体督导时，您的身份信息将被隐去，仅分享必要信息。所有接触您信息的咨询师、督导师及相关人员均签署保密协议并受其约束。
3. 咨询安排
3.1 咨询时间：
3.1.1 常规咨询每周 1 次，每次约 50 分钟。特殊情况（如婚姻家庭咨询或特定咨询方法需要）可延长至不超过 90 分钟。
3.1.2 请提前 10 分钟到达咨询室或登录线上咨询平台。未取消预约的迟到不予延长咨询时间。
3.2 预约与取消：
3.2.1 请至少提前 24 小时通知取消预约，以便为其他来访者安排时间。
3.2.2 如咨询师因出差、会议等原因无法按时提供咨询，将提前通知您并协商调整安排。
3.3 心理测试：根据您的需求，咨询师可能建议进行心理测试，以更全面了解您的心理状况。测试结果将严格保密，仅用于咨询目的。
3.4 录音录像：为提升咨询质量及接受专业督导，咨询过程可能进行录音或录像。所有记录严格遵守保密原则，仅限咨询师及督导使用，并在咨询结束后按规定销毁或妥善保存。
3.5 本校学生特别说明：若您为 XX 大学学生，您的预约信息将通过 XX 大学大学生心理健康教育与服务中心备案。若出现危机情况（如生命安全威胁、严重精神障碍等），本中心将依法依规通知该服务中心。
4. 转介原则
4.1 若您的心理问题超出本中心服务范围（如需长期治疗或专业医疗干预），咨询师将与您协商并推荐更适合的治疗师或机构。
4.2 若咨询师认为自身专业能力无法充分满足您的需求，将与您协商后转介至其他合适咨询师。
5. 您的权利与责任
5.1 权利：
5.1.1 您有权了解咨询过程、方法及咨询师的资质。
5.1.2 您有权随时提出疑问、表达需求或终止咨询。
5.1.3 您有权要求了解与您相关的保密信息处理方式。

5.2 责任:
5.2.1 请如实提供与咨询相关的个人信息和情况，以确保咨询效果。
5.2.2 请按时参加预约的咨询或提前通知取消。
5.2.3 请尊重咨询师及咨询过程，共同维护良好的咨询关系。
6. 风险与局限性
6.1 心理咨询旨在促进心理健康，但无法保证特定结果，效果因人而异。
6.2 咨询可能涉及探讨敏感话题，可能引发短暂情绪波动，咨询师将尽力提供支持。
6.3 若您对咨询过程或效果有任何疑问，欢迎随时与咨询师沟通。
7. 联系方式如有疑问或需要进一步信息，请联系本中心:
电话：XXX-XXXX-XXXX
邮箱：XXX@xxx.edu.cn
地址：XX 大学心理学部心理咨询研究与培训中心
来访者确认
本人已仔细阅读并充分理解上述内容，自愿接受 XX 大学心理学部心理咨询研究与培训中心的心理咨询服务，并同意遵守相关规定。
来访者签名：____________________
日期：___年___月___日

Document 26 Grok4 modified

心理咨询知情同意书

为秉持“以人为本，助人自助”的原则，保障来访者与心理咨询师的合法权益，确保心理咨询顺利进行，特制定本知情同意书，说明心理咨询的基本原则、双方权利与义务等事项。请您仔细阅读并确认理解后签署。

一、心理咨询基本原则

1. 咨询目标与性质
    - 心理咨询旨在通过专业方法帮助您探索内心困扰，促进自我成长与问题解决。
    - 心理咨询不能替代医学治疗或药物干预，需遵循医嘱配合治疗。
2. 保密原则
    - 保密是心理咨询的核心伦理要求。您的个人信息、咨询内容及记录将严格保密，不会泄露给任何非相关人员或机构。
    - 例外情况（在以下情形下，咨询师可能依法或为保护您及他人安全披露信息）：
        - 1.2.1 您有明显自我伤害或伤害他人的倾向；
        - 1.2.2 您的行为涉及法律责任；
        - 1.2.3 为提升咨询效果，咨询师需在专业场合（如督导或个案讨论）分享匿名化信息，仅限专业人员知晓。
    - 披露前，咨询师将尽量与您沟通并征得同意（紧急情况除外）。
3. 信息管理
    - 为提供持续咨询服务，需登记您的真实姓名、联系方式及紧急联系人信息。这些信息仅用于咨询管理，严格保密，不会泄露给任何第三方。
4. 录音或录像
    - 如需对咨询过程录音或录像，咨询师将提前告知并征得您的书面同意。您有权拒绝或随时撤回同意。
5. 咨询安排
    - 每次咨询时长一般为 50 分钟，具体次数由您与咨询师协商确定，双方应严格遵守约定时间。
    - 如需调整或取消预约，请提前 24 小时告知咨询师。
6. 风险告知
    - 心理咨询旨在帮助您应对情绪、行为或心理困扰，但无法完全消除特定症状（如自杀观念、抑郁情绪等）或保证未来不再出现类似问题。
    - 若您有自杀意念或行为史，请及时向咨询师坦诚告知，以便共同制定支持方案。咨询师将尽全力提供帮助，但您对自身行为及后果承担最终责任。
    - 对于因隐瞒诊断或症状导致的后果，咨询机构及咨询师不承担法律责任。

二、来访者的权利与义务

1. 权利
    - 2.1.1 自愿参与：您有权自主选择是否开始、继续或终止咨询，并可与咨询师协商选择适合的咨询师或调整咨询目标与方法。
    - 2.1.2 知情同意：您有权了解咨询过程、方法、潜在风险及咨询师的资质。
    - 2.1.3 隐私保护：您有权要求保护个人信息，并了解保密例外情况。
    - 2.1.4 反馈与申诉：您有权对咨询服务提出意见或向咨询机构反馈问题。
2. 义务
    - 2.2.1 坦诚沟通：以开放、诚实的态度参与咨询，真实表达感受与困扰。

- 2.2.2 积极配合：积极参与咨询过程，努力实现自我成长，不依赖咨询师为您做决定。
- 2.2.3 遵守约定：按时参加咨询，如需调整或取消，请提前告知。

三、心理咨询师的权利与义务

1. 权利
    - 3.1.1 咨询师有权根据专业判断调整咨询方案，并在必要时建议转介至其他专业服务（如精神科医生或其他机构）。
    - 3.1.2 咨询师有权在确保安全与合法的前提下，拒绝或终止不适合继续的咨询关系。
2. 义务
    - 3.2.1 提供专业、符合伦理的咨询服务，尊重您的隐私与自主性。
    - 3.2.2 向您说明咨询过程、方法、风险及可能的局限性。
    - 3.2.3 在发现您有严重风险（如自伤或伤人倾向）时，及时采取必要措施并告知您。

四、其他事项

1. 咨询效果说明
    - 心理咨询的效果因人而异，受多种因素影响（如您的参与度、问题复杂性等）。咨询师将尽力提供支持，但无法保证特定结果。
2. 紧急情况处理
    - 若您在咨询期间或咨询外出现紧急情况（如强烈自杀冲动），请立即联系咨询师、紧急联系人或拨打当地心理援助热线（如中国大陆：12320 或当地心理危机干预热线）。
3. 服务终止
    - 您可随时选择终止咨询，建议与咨询师进行一次总结性会谈以规划后续支持。
    - 若因特殊情况（如咨询师判断您需转介至更适合的服务）需终止咨询，咨询师将提前告知并协助转介。

五、签署确认

我已仔细阅读并理解本知情同意书的内容，同意遵守上述条款，自愿参与心理咨询。我了解咨询的性质、风险及双方责任，并知晓可随时向咨询师或机构咨询疑问。

来访者姓名：________________

签名：________________

日期：________________

咨询师姓名：________________

签名：________________

日期：________________

Document 27 Grok4 modified

心理咨询知情同意书

为保障心理咨询的有效性与专业性，保护来访学生（以下简称“来访者”）与咨询教师（以下简称“咨询师”）的合法权益，明确双方权利与义务，请来访者仔细阅读并理解以下内容。本协议适用于 XX 大学学生工作处心理健康教育服务中心（以下简称“心理中心”）提供的心理咨询服务。

一、来访者的权利与义务

1. 咨询规范

   1.1 来访者应准时赴约，每次咨询时长约为 50 分钟，两次咨询间隔通常不少于 1 周。

   1.2 如需取消或更改预约，请至少提前 24 小时通知心理中心。

   1.3 首次咨询时，请携带有效学生证件以核验身份。

2. 坦诚沟通

   2.1 来访者需如实提供个人信息及心理状况，坦诚表达真实感受。

   2.2 若因隐瞒或掩饰信息导致咨询效果受限，来访者需自行承担相关后果。

3. 咨询认知

   3.1 心理咨询是一个协作、渐进的过程，旨在促进自我成长与问题解决。咨询师作为倾听者、陪伴者与助人者，非决策者或问题解决者。

   3.2 单次咨询无法解决所有问题，来访者的积极参与和持续努力是咨询成功的关键。

   3.3 心理测评（如量表）为辅助工具，用于了解来访者状况，不具有临床诊断效力。

4. 来访者权利

   4.1 来访者有权了解咨询师的资质、咨询方式及流程。

   4.2 来访者可随时提出更换咨询师或终止咨询，但需知晓可能对咨询进程的影响。

   4.3 来访者有权对咨询服务提出反馈或投诉，并可联系心理中心获取支持。

二、咨询师的权利与义务

1. 保密原则

   1.1 咨询师严格保护来访者的个人信息及咨询内容隐私，未经同意不得向第三方披露。

   1.2 如需在教学、科研或专业督导中使用案例，咨询师将匿名化处理，确保无法识别来访者身份。

2. 保密例外

   2.1 在以下情况下，咨询师可能依法或依伦理要求披露信息：

   - 来访者存在明显伤害自身或他人的风险；
   - 来访者疑似患有严重心理或精神障碍，需转介专业机构；
   - 来访者涉及危及生命健康的传染性疾病（如 A 类、B 类传染病）；
   - 来访者近期遭受严重侵害（如虐待、暴力等）；
   - 法律法规要求披露的其他情况。

     2.2 披露前，咨询师将尽可能与来访者沟通，并尽量限制披露范围。

3. 专业规范

   3.1 咨询师秉持专业态度，耐心倾听，真诚协助来访者探索与解决问题。

   3.2 咨询师仅提供心理咨询服务，不提供精神疾病诊断、心理治疗或药物治疗等医疗服务。

4. 咨询师权利

   4.1 咨询师可根据专业判断，建议转介至更适合的机构或专业人员（如精神科医生）。

4.2 如咨询关系无法有效推进，咨询师可在充分沟通后终止咨询，并说明原因。

三、咨访双方共同约定

1. 咨询目标

1.1 咨访双方通过协商，共同制定具体、可行、积极且符合心理咨询性质的目标。

1.2 咨询目标可根据咨询进展适时调整。

2. 避免多重关系

2.1 为维护咨询关系的专业性，咨访双方应避免建立除咨询关系外的其他关系（如经济、商业或私人关系）。

3. 联系方式

3.1 咨询相关沟通优先通过心理中心官方电话或邮箱进行。

3.2 非紧急情况下，手机、微信等个人联系方式可能无法保证及时回复。

4. 服务范围与限制

4.1 本服务面向 XX 大学在校学生，主要针对学业、情绪、人际关系等常见心理困扰。

4.2 对于超出心理咨询范围的问题（如严重精神障碍），心理中心将协助转介至专业机构。

5. 知情同意与终止

5.1 来访者签署本协议即表示已充分理解并同意遵守上述条款。

5.2 任何一方终止咨询时，应及时告知对方并进行必要沟通。

四、其他事项

1. 咨询费用

1.1 XX 大学心理中心为在校学生提供免费心理咨询服务。

1.2 如涉及转介服务，可能由外部机构按其标准收费，心理中心将提前告知。

2. 反馈与投诉

2.1 来访者可通过心理中心官方渠道提出服务反馈或投诉，心理中心将及时处理。

3. 协议修订

3.1 本协议可能根据法律法规或心理中心政策更新，最新版本以心理中心公布为准。

声明

本人已仔细阅读并理解上述内容，同意遵守相关约定，并自愿接受 XX 大学学生工作处心理健康教育服务中心的心理咨询服务。

来访者签名：____________________

日期：______年______月______日 心理中心（盖章）：

XX 大学学生工作处心理健康教育服务中心

日期：______年______月______日

Document 28 Grok4 modified

个体心理咨询知情同意书

亲爱的来访者：欢迎您选择心理健康教育中心的心理咨询服务！为保障您与咨询师的权益，规范咨询过程，确保咨询顺利进行，我们特制定本《个体心理咨询知情同意书》。请您仔细阅读以下内容，并在充分理解后签名确认，表示您已知晓并同意遵守相关条款。

一、心理咨询服务概述

1.1 服务对象与内容

本中心为全校学生免费提供心理咨询服务，旨在帮助您应对学习、生活、个人成长及自我发展相关问题。服务内容包括心理评估与咨询，但不包括精神疾病诊断、开具精神类药物处方、提供资格证明或针对严重精神障碍的长程心理治疗。

1.2 咨询安排

- 每次咨询时长：50 分钟。
- 咨询频率：每周 1 次，每学期最多 8 次。
- 咨询时间：_______（由中心与您协商确定）。
- 咨询地点：_______（由中心指定）。

1.3 服务限制

若您被专业医疗机构诊断为精神疾病或需药物治疗，建议前往专科医院就诊。心理咨询不能替代药物治疗，请严格遵循医嘱用药。

二、来访者权利与责任

2.1 权利

- 您有权随时终止咨询，但建议在终止前与咨询师充分沟通您的决定。
- 您有权了解咨询过程、目标及可能的局限性，咨询师将以通俗易懂的方式向您解释。
- 您有权对咨询服务提出反馈或疑问，中心将认真回应。

2.2 责任

- 准时性：请按预约时间准时到达咨询室。若需更改或取消预约，请至少提前 1 个工作日通知中心。连续两次无故缺席将导致剩余咨询资格取消。
- 迟到处理：若您迟到，咨询时长将不予延长，仍按原定时间结束。
- 信息披露：若您曾被专业医疗机构诊断为精神疾病，请主动提供诊断证明并如实告知相关病史。如因隐瞒病史导致的后果，中心及咨询师不承担责任。

三、咨询师责任与行为规范

3.1 专业职责

- 咨询师将秉持专业态度，为您提供安全、支持性的咨询环境。
- 咨询师不得接受您的礼物，或在咨询室外与您进行与咨询相关的交流，以维护专业边界。

3.2 录音/录像管理

- 若需对咨询过程录音或录像，须事先征得双方同意，并明确用途及保存方式。未经同意，任何一方不得擅自录音或录像。

四、保密原则与例外

4.1 保密承诺

- 咨询师对您在咨询中提供的所有信息严格保密，包括但不限于个人信息、咨询内容及记录。
- 若需因学术研究、教学或专业督导使用咨询信息，将严格隐去可识别您身份的任何信息。

4.2 保密例外

在以下情况下，咨询师可能依法或依伦理要求披露相关信息：

- 您有明确证据表明可能对自己或他人造成严重伤害（如自伤、他伤或自杀风险）。
- 司法机关依法要求提供相关信息。
- 其他法律或伦理规定的特殊情况。

  在上述情况下，咨询师将尽量提前与您沟通，并在必要范围内披露信息。

五、紧急情况支持

5.1 咨询间隔期支持

- 若在两次咨询间隙遇到心理困扰，可在工作时间拨打中心办公电话：85250525。
- 非工作时间如遇紧急情况，可拨打以下热线：
  - 长春市心理援助热线：12320，0431-89685000，0431-89685333。
  - 全国心理援助热线：800-810-1117（仅限固定电话），010-82951332（固定/移动电话均可）。

5.2 紧急转介

若咨询师评估您的情况超出本中心服务范围，将协助您转介至专业医疗机构。

六、知情同意与签名

6.1 声明

本人已仔细阅读并理解上述条款，同意遵守相关规定，并自愿参与心理咨询服务。

6.2 签名

来访者姓名：_______ 签名：_______ 日期：_______

咨询师姓名：_______ 签名：_______ 日期：_______

Document 29 Grok4 modified

XX 大学心理健康教育中心

心理咨询知情同意书

亲爱的同学：您好！

心理咨询是心理咨询师与来访者基于信任建立的专业助人关系，旨在帮助您更好地应对心理困扰，提升心理健康。本知情同意书旨在帮助您了解心理咨询的相关信息，明确双方的权利与义务，以确保咨询过程顺利开展。

本中心为 XX 大学正式注册的学生提供免费心理咨询服务，但不提供精神障碍诊断、治疗、药物处方、医疗证明或心理治疗等医疗服务。

请您仔细阅读以下内容，并在充分理解后签署本协议。

一、心理咨询设置

1.1 预约方式

- 心理咨询需由您主动提前预约，预约方式可通过本中心官网、电话或现场登记完成。
- 首次咨询前，中心将安排初始评估访谈，以了解您的需求并安排后续服务。

1.2 咨询地点

- 所有咨询在本中心指定地点进行，具体地点将在预约时告知。

1.3 咨询时间

- 每次咨询时长为 50 分钟，原则上每周一次。
- 每位学生每学期最多可接受 8 次咨询，特殊情况需经中心评估后调整。

1.4 迟到与缺席

- 如您迟到，咨询时间将不予补足或延长；迟到超过 20 分钟或缺席，本次咨询自动取消。
- 若您因故需改期或取消预约，请至少提前 24 小时通过中心电话或官网告知。
- 每学期无故取消或迟到累计达 2 次，需重新排队等候预约。
- 如咨询师因故迟到或缺席，将提前 24 小时通知，并为您补足咨询时间或重新安排。

二、咨询师的责任与义务

2.1 保密原则

- 本中心所有工作人员（包括咨询师、行政人员等）对您的个人信息及咨询内容严格保密。
- 为提升服务质量，您的案例可能在隐去可识别身份信息后，用于本中心内部专业研讨、督导或培训，所有参与者均遵守保密义务。

2.2 保密例外情况

根据法律法规及伦理规范，以下情况可能需披露您的信息：

- 您存在伤害自身或他人的严重风险；
- 涉及未成年人或其他无完全民事行为能力者遭受性侵犯、虐待等情况；
- 法律、法规或司法机构要求披露相关信息。

  如发生上述情况，中心将在必要范围内披露信息，并尽可能事先告知您。

2.3 录音录像

- 未经双方同意，咨询师与来访者均不得对咨询过程进行录音或录像。
- 如需录音录像用于专业培训或督导，咨询师将提前与您协商并签署单独的知情同意书。

三、来访者的权利与义务

3.1 来访者的权利

- 您有权了解咨询师的资质、咨询过程及相关安排。
- 您有权申请更换咨询师，每学期最多可更换一次，中心将根据实际情况安排。
- 您有权随时提出终止咨询，建议在咨询中与咨询师充分讨论后再协商决定。

3.2 来访者的义务

- 为确保咨询效果，您需尽可能真实、完整地向咨询师提供相关信息，包括心理问题现状、既往评估或诊断等。
- 积极配合咨询师，参与咨询过程，完成双方商定的咨询目标或任务。
- 若因未如实提供信息导致不良后果，由您自行承担相应责任。

四、转介与终止

4.1 转介

- 初始访谈后，若您的需求超出本中心服务范围（如需精神科医疗服务），咨询师将向您解释并提供适合的求助资源或转介建议。
- 若咨询议题超出咨询师专业能力，咨询师将与您协商并妥善安排转介事宜。

4.2 终止

- 咨询目标达成或您提出终止时，咨询可结束，建议与咨询师充分讨论后再决定。
- 若您连续缺席或不配合咨询，中心保留暂停或终止服务的权利，并提前通知您。

五、费用

- 本中心为在校学生提供免费心理咨询服务，无任何附加费用。

六、紧急情况处理

- 在两次咨询间隔期，如遇紧急情况，您可于工作时间（周一至周五，9:00-17:00）拨打本中心办公电话：86608122 或 86601122。
- 非工作时间，请拨打当地心理援助热线：
    - 白班（8:00-20:00）：0371-86169595
    - 晚班（20:00-8:00）：0371-22993442
- 紧急情况下，请及时联系您的辅导员或拨打 120/110 寻求帮助。

七、协议保存与生效

- 本协议由 XX 大学心理健康教育中心代为保存，保存期限遵循相关法律法规及伦理规范。
- 本协议自您签署之日起生效，适用于您在本中心接受的所有心理咨询服务。

签署声明

我已仔细阅读并充分理解上述内容，同意在 XX 大学心理健康教育中心接受心理咨询服务，并遵守相关规定。

来访者签名：__________________

日期：__________________

心理咨询中心代表签名：__________________

日期：__________________

Document 30 Grok4 modified

XX 大学心理健康教育与咨询中心

心理咨询知情同意书亲爱的同学：

您好！欢迎选择 XX 大学心理健康教育与咨询中心的心理咨询服务，感谢您的信任！为确保您充分了解心理咨询的过程与相关事项，请仔细阅读以下内容，并在理解后签署本同意书。

一、服务对象

1.1 本中心的心理咨询服务面向全体在校学生，旨在为有心理困扰或希望提升心理健康的同学提供支持。

二、服务内容

2.1 提供心理困扰的鉴别、疏导与调整服务，帮助来访者更好地应对学业、生活、情感等方面的问题。

2.2 根据需要，提供心理健康教育、危机干预或转介服务。

三、心理咨询原则

3.1 尊重与平等

3.1.1 本中心秉持尊重、平等、真诚的服务理念，致力于为每位来访者提供安全、包容的咨询环境。

3.1.2 您有权了解咨询师的专业资质、服务内容、咨询时间及方式等信息，并根据自身需求选择适合的咨询师、咨询方式及时间。

3.1.3 若对咨询过程或进展不满意，您可随时与咨询师沟通调整，或选择终止咨询。

3.2 保密原则

3.2.1 本中心严格遵守保密原则，咨询中的所有谈话内容及相关资料将妥善保管，不向第三方披露。

3.2.2 例外情况：

- 若您存在伤害自己、他人或公共安全的风险，咨询师可能需依法向相关人员或机构披露必要信息，以确保安全。
- 若需转介至其他专业机构（如精神科医生），咨询师可能在征得您同意后与相关专业人员共享必要信息。
- 若涉及法律要求（如法院传票），咨询师需依法提供相关资料。

3.2.3 以上例外情况发生前，咨询师将尽可能与您沟通，并说明必要性。

3.3 自主性

3.3.1 心理咨询遵循“助人自助”理念，咨询师将陪伴并支持您，但最终的决定和改变由您负责。

3.3.2 您有权自主决定是否开始、继续或终止咨询，咨询师仅提供建议，不予强制。

3.4 坦诚与合作

3.4.1 为取得最佳咨询效果，请尽可能坦诚表达您的感受、困惑或咨询过程中的疑问，与咨询师充分沟通。

3.4.2 若对咨询方向、方法或进程有异议，可与咨询师讨论并协商调整。

3.5 自愿与责任

3.5.1 接受心理咨询完全基于自愿，您可随时决定终止咨询。

3.5.2 若随意终止咨询可能导致的不良后果（如问题未缓解），由您自行承担。

3.6 持续性与耐心

3.6.1 心理问题的解决通常需要一定时间，咨询过程循序渐进，难以一次解决问题，请保持耐心与信心，积极配合咨询安排。

四、咨询设置

4.1 预约与时长

4.1.1 心理咨询采用预约制，每次咨询时长为 40-60 分钟。

4.1.2 若一次咨询未能完成，可在咨询结束后与咨询师约定下次咨询时间。

4.2 取消或更改预约

4.2.1 如需取消或更改预约，请至少提前 24 小时联系中心说明情况，并确定新的咨询时间。

4.2.2 未提前通知的缺席可能影响后续预约安排。

4.3 咨询方式

4.3.1 咨询方式包括但不限于面对面咨询、电话咨询或线上咨询，具体方式由您与咨询师协商确定。

4.4 费用

4.4.1 本中心为在校学生提供免费心理咨询服务。

五、来访者权利与义务

5.1 权利

5.1.1 您有权了解咨询师的资质、经验及咨询安排。

5.1.2 您有权在咨询过程中提出疑问、建议或反馈，并获得咨询师的回应。

5.1.3 您有权随时终止咨询，并可要求转介至其他咨询师或机构。

5.2 义务

5.2.1 请按时参加预约的咨询，配合咨询师完成咨询过程。

5.2.2 请尊重咨询师的工作，保持礼貌与合作的沟通态度。

六、风险与局限性

6.1 心理咨询可能涉及探讨敏感或痛苦的情绪，您可能在过程中感到暂时的不适，这是正常现象，咨询师将尽力支持您。

6.2 心理咨询的效果因人而异，取决于您的参与度、问题性质及外部环境等因素，无法保证特定结果。

6.3 若咨询师评估您的需求超出本中心服务范围，将建议转介至更适合的专业机构（如精神科或医疗机构）。

七、联系方式

7.1 如有疑问或需进一步了解，可通过以下方式联系本中心：

- 电话：【请填写中心联系电话】
- 邮箱：【请填写中心邮箱】
- 办公地址：【请填写中心地址】
- 工作时间：【请填写中心工作时间】

八、知情同意声明

我已仔细阅读并理解以上《心理咨询知情同意书》的内容，清楚心理咨询的过程、原则、权利与义务，以及可能的风险与局限性。我同意接受 XX 大学心理健康教育与咨询中心的心理咨询服务，并遵守相关约定。

来访者签名：____________________

日期：_______年_______月_______日

咨询师签名（如适用）：____________________

日期：_______年_______月_______日

Document 31 Grok4 modified

心理咨询知情同意书

亲爱的同学：您好！欢迎选择本中心提供的心理咨询服务。心理咨询是心理咨询师与来访者之间建立的专业助人关系，旨在通过专业支持帮助您更好地应对心理困扰。本知情同意书旨在明确双方的权利与义务，确保咨询过程顺利开展。请您仔细阅读以下内容，并在充分理解后签署。

一、心理咨询服务概述

1. 服务对象：本中心为重庆理工大学正式注册的学生提供免费心理咨询服务。
2. 服务范围：提供心理咨询与支持服务，不包括精神障碍诊断、治疗、处方开具、医疗证明开具或心理治疗等医疗服务。
3. 服务目标：通过专业咨询，促进来访者的心理健康与个人成长。

二、心理咨询设置

1. 预约方式：
    - 需由有咨询意愿的同学主动提前预约。
    - 预约方式：通过本中心官方渠道（如电话 62563208 或线上系统）进行。
2. 咨询地点：
    - 花溪校区：学生活动中心 3 楼大学生心理健康与成才服务中心。
    - 两江校区：学生公寓 2 栋 112-114 室。
3. 咨询时间：
    - 每周一次，每次 50 分钟，具体时间根据中心提供的预约时段安排。
    - 每位学生每学年最多可接受 8 次咨询，特殊情况需经中心评估。
4. 迟到与缺席：
    - 双方需遵守约定时间。如来访者迟到，咨询时间不予补足；迟到超过 30 分钟或缺席，本次咨询取消。
    - 一学期内无明确原因取消或迟到累计达 3 次，需重新预约等候。
    - 如咨询师未提前 24 小时告知迟到或缺席，将为来访者补足咨询时间。

三、咨询师的责任与义务

1. 保密原则：
    - 咨询师及中心工作人员对来访者的个人信息及会谈内容严格保密。
    - 为提升服务质量，隐去可识别身份信息后，案例可能用于内部研讨或督导，所有参与人员均承诺遵守保密义务。
    - 所有案例相关信息的使用均符合《美国心理学会（APA）伦理准则》及《中国心理学会临床与咨询心理学工作伦理守则》等专业规范。
2. 保密例外：
    - 当来访者存在伤害自身或他人的风险时，咨询师将根据《重庆理工大学心理危机干预流程》采取必要措施（如通知相关部门或家属）。
    - 未成年人或不具备完全民事行为能力者遭受性侵犯或虐待时，需依法报告。
    - 法律、司法、公安或卫生部门依法要求披露信息时。
3. 录音录像：
    - 未经双方同意，咨询师与来访者均不得对咨询过程录音或录像。
    - 如需录音录像以提升专业能力，咨询师将与来访者另行签署知情同意协议。

四、来访者的权利与义务

1. 来访者的权利：

- 有权了解咨询过程、方法及咨询师资质。
- 有权申请更换咨询师，每学期最多一次。
- 有权随时终止咨询，建议在咨询中与咨询师充分讨论后协商决定。
- 有权对咨询服务提出反馈或投诉，联系方式见“六、联系方式”。

2. 来访者的义务：
    - 提供真实的心理问题相关信息，包括其他专业机构的评估或诊断结果。
    - 积极配合咨询师，参与讨论并完成双方商定的咨询任务。
    - 改期或取消预约需提前 24 小时通知中心。
    - 对未如实提供信息或未遵守咨询设置导致的后果自行负责。

五、转介与咨询终止

1. 转介：
    - 初始评估后，若来访者问题超出本中心服务范围或咨询师专业能力，咨询师将向来访者说明并提供其他求助资源（如专业医疗机构）。
    - 转介过程中产生的任何纠纷由来访者自行负责，与本中心无关。
2. 咨询终止：
    - 来访者可随时提出终止咨询，建议与咨询师协商。
    - 若因来访者多次违反咨询设置或咨询效果不佳，中心可终止服务并说明原因。

六、费用

- 本中心为在校学生提供免费心理咨询服务。

七、联系方式

1. 常规联系：
    - 工作时间（周一至周五，10:00-12:00，14:00-17:00，19:00-22:00）：
        - 电话：023-62563208
        - 地址：花溪校区学生活动中心 3 楼或两江校区学生公寓 2 栋 112-114 室
2. 紧急情况：
    - 非工作时间可拨打重庆市心理援助热线：023-12320-1
    - 紧急情况（如涉及生命安全）可直接拨打 110 或 120。

八、紧急联系人

1. 来访者需提供一位紧急联系人的真实、准确、有效信息（姓名、联系方式、关系等）。
2. 在紧急情况下（如存在危及自身或他人生命安全的情况），中心可能联系该紧急联系人，通报必要状况，但不会泄露隐私内容。
3. 若提供的联系人信息无效，由此导致的后果由来访者自行承担。

九、签署声明

本人已仔细阅读并充分理解上述内容，自愿接受本中心的心理咨询服务，同意遵守相关规定，并对自己的行为负责。

来访者签名：________________

日期：_____________________

紧急联系人信息：

姓名：________________

关系：________________

联系电话：________________ 心理咨询中心（盖章）：

日期：_____________________

Document 32 Grok4 modified

XX 大学学生心理健康教育与咨询中心

心理咨询知情同意书为保障来访者与咨询师的权益，提升心理咨询服务的专业水平，根据《中华人民共和国精神卫生法》及《中国心理学会临床与咨询心理学工作伦理守则(第二版)》的“善行、责任、诚信、公正、尊重”原则，特制定本知情同意书。请您仔细阅读并确认理解以下内容：

一、心理咨询的定义与目的

1.1 心理咨询是指在专业咨询关系基础上，由具备资质的心理咨询师运用心理学理论与技术，帮助来访者缓解心理困扰，促进心理健康与个人成长的过程。

1.2 本中心致力于为来访者提供安全、专业的心理支持，帮助您实现共同制定的咨询目标。

二、咨询服务流程

2.1 首次咨询要求

- 请携带有效学生证，并按预约时间准时到达。
- 如无法按时到访，请至少提前 24 小时通过电话或中心指定方式告知。

2.2 咨询安排

- 咨询将在中心指定咨询室内进行，不得要求在咨询室外开展。
- 本中心提供免费心理咨询服务，您无需支付任何费用。

2.3 咨询目标与配合

- 请以积极、开放、诚实的态度参与咨询，配合咨询师共同制定并努力实现咨询目标。
- 需按要求填写相关文件（如评估表、反馈表等）。

2.4 中断或转介

- 您有权随时提出中断咨询或更换咨询师，本中心将及时安排新的咨询师或协助转介至其他合适服务机构。

2.5 续约咨询

- 如需继续咨询，请提前与本中心预约确认。

三、隐私与保密原则

3.1 保密承诺

- 本中心严格遵守《中国心理学会临床与咨询心理学工作伦理守则（第二版）》的保密原则，保护您的个人隐私及咨询内容。
- 未经您明确同意，咨询信息不会向第三方披露。

3.2 保密例外情况

根据法律法规及伦理守则，以下情况可能需要披露信息，中心将依法与相关方面联系：

- (1) 发现您有严重伤害自身或他人的危险；
- (2) 未成年人或无完全民事行为能力者遭受性侵犯、虐待或其他严重伤害；
- (3) 法律法规要求披露的其他情况。

3.3 录音或录像

- 为提升咨询质量，用于个案督导、研讨或教学研究，中心可能需对咨询过程进行录音或录像。
- 录音或录像前，中心将征得您的书面同意，并严格保护相关资料的保密性。

3.4 数据管理

- 所有与咨询相关的信息（包括录音、录像及书面记录）将妥善保存，仅限授权专业人员在必要时使用，并遵守保密规定。

四、来访者权利与义务

4.1 权利

- 您有权了解咨询师的资质、咨询过程及相关安排。
- 您可随时提出问题、建议，或对咨询服务提出反馈。
- 您有权拒绝录音、录像或任何不适的咨询安排。

4.2 义务

- 请保持积极配合的态度，诚实表达感受与需求。
- 在咨询中心内保持安静，爱护公共设施，尊重其他来访者及工作人员。
- 遵守中心预约及相关规定。

五、其他事项

5.1 本中心保留根据实际情况调整咨询安排的权利，并将及时告知您。

5.2 如有疑问或需进一步了解，可随时联系本中心工作人员。

六、声明与签署

我已仔细阅读并理解上述内容，同意接受重庆师范大学学生心理健康教育与咨询中心的心理咨询服务，并遵守相关规定。

来访者签名： ____________________

日期： ______年______月______日

咨询中心联系方式：

电话：_________

邮箱：_________

地址：XX 大学学生心理健康教育与咨询中心

Document 33 Grok4 modified

高校心理咨询知情同意书亲爱的同学，您好！

心理咨询是一种专业的助人关系，旨在通过心理咨询师与来访者的合作，帮助您更好地应对心理困扰或提升心理健康。本知情同意书旨在清晰说明双方的权利与义务，确保心理咨询过程顺利开展。

本中心为本校正式注册学生提供免费心理咨询服务，不提供精神障碍诊断、治疗、处方开具、医疗证明或心理治疗等服务。

请您仔细阅读以下内容，并在充分理解后签署本协议。

一、心理咨询设置

1. 预约方式
    - 心理咨询需由您主动提前预约，可通过本中心指定方式（电话、线上系统等）进行。
    - 未预约的情况下，中心无法保证即时提供咨询服务。
2. 咨询地点
    - 所有咨询在本中心指定的专业咨询室进行，确保安全与私密性。
3. 咨询时间
    - 每次咨询时长为 50 分钟，通常每周一次，具体时间根据中心安排预约。
    - 每位学生每学期最多可接受 8 次咨询，具体次数以中心安排为准。
4. 咨询形式
    - 咨询以面对面形式为主，特殊情况下（如疫情）可协商提供线上咨询，需另行签署协议。

二、咨询师的责任与义务

1. 保密原则
    - 咨询师及中心工作人员对您的个人信息和咨询内容严格保密。
    - 为提升服务质量，您的案例可能在隐去可识别身份信息后，用于中心内部专业研讨或督导，所有参与人员均遵守保密义务。
    - 所有信息使用均符合中国心理学会伦理规范及相关法律法规。
2. 保密例外

    在以下情况下，咨询师可能依法披露部分信息：
    - 您存在严重伤害自身或他人的风险；
    - 未成年人或其他无完全民事行为能力者遭受性侵犯、虐待等情况；
    - 法律、法规或司法程序要求披露。
3. 录音录像
    - 未经双方同意，咨询师和来访者均不得对咨询过程进行录音或录像。
    - 如需录音录像（用于培训或督导），咨询师将提前告知并与您另行签署书面同意书。
4. 迟到与缺席
    - 咨询师如因故迟到或缺席，未提前 24 小时通知中心，将为您补足咨询时间或重新安排。
    - 咨询师将尽力准时并确保咨询环境的稳定性。

三、来访者的权利与义务

1. 来访者权利
    - 您有权了解咨询师的专业资质、咨询过程及相关安排。

- 您有权申请更换咨询师，每学期最多可更换一次，需向中心说明原因。
- 您有权随时提出终止咨询，建议在咨询中与咨询师充分讨论后协商决定。

2. 来访者义务
   - 为确保咨询效果，您需尽量如实提供心理问题的相关信息，包括其他专业机构的评估或诊断结果。
   - 您应积极参与咨询过程，配合完成双方商定的咨询目标或作业。
   - 未如实提供信息可能导致的不良后果由您自行承担。
3. 预约调整与取消
   - 改期或取消预约需至少提前 24 小时通过中心指定方式办理。
   - 无明确原因迟到或取消累计达 3 次（每学期），需重新预约并可能进入等候名单。
   - 若您迟到，咨询时间不予延长；迟到超过 30 分钟或缺席，当次咨询自动取消。

四、转介与评估

1. 初始评估
   - 首次咨询将进行初步评估，以确定您的需求是否适合本中心服务范围。
   - 若您的需求超出本中心服务范围（如需精神科治疗），咨询师将向您解释并提供适当的求助资源或转介建议。
2. 转介安排
   - 若咨询议题超出咨询师的专业能力，咨询师将与您协商并妥善安排转介，确保您获得适合的支持。

五、费用

- 本中心为在校学生提供免费心理咨询服务，无需支付任何费用。

六、紧急情况与求助渠道

1. 咨询间隔期支持
   - 在两次咨询间隔期间，如遇紧急情况，可在工作时间拨打本中心办公电话：_______。
   - 非工作时间，请拨打当地心理援助热线：_______。
2. 危机干预
   - 若您出现严重心理危机（如自杀意念或行为），请立即拨打当地急救电话（如 120）或心理援助热线，并告知本中心。

七、协议签署与效力

1. 本协议一式两份，心理咨询中心与来访者各执一份，具有同等效力。
2. 本协议自双方签字之日起生效，适用于您在本中心的全部咨询过程。

声明

我已仔细阅读并充分理解上述内容，同意在 大学心理咨询中心 接受心理咨询服务，并遵守相关约定。

来访者签字：_________________ 日期：_________________

咨询师签字：_________________ 日期：_________________
