# Supplementary material for: Optimization of University Counseling Consent Forms With Large Language Models: Multidimensional Comparative Evaluation
Source: J Med Internet Res. 2026 Apr 1;28:e86502. doi: 10.2196/86502 (PMC13043017; doi:10.2196/86502)
Supplement: Multimedia Appendix 4 [file jmir-v28-e86502-s004.pdf]

Section 1. Consistency and intraclass correlation coefficient (ICC) calculation

```
# ==========================================================
# Supplementary Material: Python Code for Inter-Rater Reliability Analysis
# This script calculates Intraclass Correlation Coefficients (ICC) for inter-rater reliability
# of content quality ratings (5 raters, total score A) and reading comprehension ratings
# (10 raters, composite score) across 99 texts (Original, ChatGPT, Grok versions, 33 texts each).
# ICC(2,1) (single rater consistency) and ICC(2,k) (average rater consistency, k=5 or 10)
# are computed for each version and overall dataset.

import pandas as pd
import pingouin as pg

# --- Content Quality Ratings ---
# Data: 99 texts (33 Original, 33 ChatGPT, 33 Grok), 5 raters (R1-R5), total score A (0-100)
content_data = pd.DataFrame({
	'version': ['Original']*33 + ['ChatGPT']*33 + ['Grok']*33,
	'text': [str(i).zfill(2) for i in range(1, 34)]*3,
	'R1': [ ],
	'R2': [ ],
	'R3': [ ],
	'R4': [ ],
	'R5': [ ]
})

# --- Reading Comprehension Ratings ---
# Data: 99 texts (33 Original, 33 ChatGPT, 33 Grok), 10 raters (R1-R10), composite score (1-
25)
reading_data = pd.DataFrame({
	'version': ['Original']*33 + ['ChatGPT']*33 + ['Grok']*33,
	'text': [str(i).zfill(2) for i in range(1, 34)]*3,
	'R1': [ ],
	'R2': [ ],
	'R3': [ ],
	'R4': [ ],
	'R5': [ ],
	'R6': [ ],
	'R7': [ ],
	'R8': [ ],
	'R9': [ ],
	'R10': [ ]
})

# Function to calculate ICC for a given dataset
def calculate_icc(data, rating_columns, name):
```

```
    # Generate unique Text_ID
    data['Text_ID'] = data['version'] + '_' + data['text']

    # Convert to long format
    data_long = pd.melt(
        data,
        id_vars=['Text_ID', 'version'],
        value_vars=rating_columns,
        var_name='Rater',
        value_name='Score'
    )

    # Split by version and overall
    data_original = data_long[data_long['version'] == 'Original']
    data_chatgpt = data_long[data_long['version'] == 'ChatGPT']
    data_grok = data_long[data_long['version'] == 'Grok']
    data_all = data_long

    # Calculate ICC for each subset
    icc_results = {}
    for subset_name, df in [('Original', data_original), ('ChatGPT', data_chatgpt), ('Grok',
data_grok), ('All', data_all)]:
        icc = pg.intraclass_corr(data=df, targets='Text_ID', raters='Rater', ratings='Score')
        icc_results[subset_name] = icc.set_index('Type')[['ICC', 'F', 'df1', 'df2', 'pval',
'CI95%']].round(3)

    # Print results
    print(f"\n{name} ICC Results:")
    for subset_name, icc in icc_results.items():
        print(f"{subset_name}:\n{icc}\n")

    return icc_results

# Run ICC calculations
content_icc = calculate_icc(content_data, ['R1', 'R2', 'R3', 'R4', 'R5'], "Content Quality")
reading_icc = calculate_icc(reading_data, ['R1', 'R2', 'R3', 'R4', 'R5', 'R6', 'R7', 'R8', 'R9', 'R10'],
"Reading Comprehension")
```

Section 2. Text structure and readability calculation

```
# ============================================================

# -*- coding: utf-8 -*-
"""
Batch-compute expression-quality metrics (including the Lee–Yang readability index) and
export to Excel:

1) character_count (characters, whitespace removed)
2) word_count (hybrid count: each Chinese character = 1; English/digits counted by \\w+
tokens)
3) sentence_count (split by 。！？； and newlines)
4) words_per_sentence (average sentence length by "words")
5) characters_per_word (average word length: characters per word)
6) nested_sentence_ratio (share of nested sentences: ≥ 2 separators or ≥ 2 subordinate
conjunctions)
7) LY_readability_index (Lee–Yang: LY = 0.8*ASL + 3.129*AWL;
   ASL = characters/sentence; AWL = characters/word)
8) tone_friendliness_0to5 (tone friendliness: 0–5)

Input:
- Word documents directory: ---
Output:
- Excel: ---
"""

import os
import re
from typing import List
from docx import Document
from openpyxl import Workbook

# ======================== Fixed path configuration
========================
DOC_DIR     = r"---"
OUT_XLSX    = r"---"
FILE_RANGE = range(1, 34)   # 1..33

# ===================== Basic regex / utilities =====================
SENT_SPLIT_RE = re.compile(r'[。！？!?；;]\s*|\n+')
CJK_RE            = re.compile(r'[\u4e00-\u9fff]')
WORD_EN_RE       = re.compile(r'[A-Za-z0-9]+')

def split_sentences(text: str) -> List[str]:
```

```
    sents = [s.strip() for s in SENT_SPLIT_RE.split(text) if s and s.strip()]
    return sents if sents else ([text.strip()] if text.strip() else [])

def character_count(text: str) -> int:
    return len(re.sub(r'\s+', '', text))

def mixed_word_count(text: str) -> int:
    chinese_chars = CJK_RE.findall(text)
    en_tokens     = WORD_EN_RE.findall(text)
    return len(chinese_chars) + len(en_tokens)

def sentence_count(text: str) -> int:
    return len(split_sentences(text))

def words_per_sentence(text: str) -> float:
    sc = sentence_count(text)
    wc = mixed_word_count(text)
    return wc / sc if sc > 0 else 0.0

def characters_per_word(text: str) -> float:
    wc = mixed_word_count(text)
    cc = character_count(text)
    return cc / wc if wc > 0 else 0.0

# ====================== Nested sentences / Lee–Yang
======================
SUBORD_CONJ = ["如果", "由于", "因此", "以便", "从而", "并且", "或者", "而且", "同时", "然而
", "但是", "尽管", "除非"]
SEP_MARKS    = [",", "，", "；", ";", "、"]

def nested_sentence_ratio(text: str) -> float:
    sents = split_sentences(text)
    if not sents:
        return 0.0
    complex_cnt = 0
    for s in sents:
        sep_hits  = sum(s.count(m) for m in SEP_MARKS)
        conj_hits = sum(1 for c in SUBORD_CONJ if c in s)
        if sep_hits >= 2 or conj_hits >= 2:
            complex_cnt += 1
    return complex_cnt / len(sents)

def ly_readability_index(text: str) -> float:
    """
```

```
    Lee–Yang index (official formula):
      LY = 0.8 * ASL + 3.129 * AWL
    """
    cc = character_count(text)
    sc = sentence_count(text)
    wc = mixed_word_count(text)
    asl = (cc / sc) if sc > 0 else 0.0
    awl = (cc / wc) if wc > 0 else 0.0
    return 0.8 * asl + 3.129 * awl

# ====================== Tone friendliness ======================
POLITE_CUES = ["请", "感谢", "谢谢", "欢迎", "如有疑问请", "如需帮助", "联系我们", "敬请", "歉意", "抱歉", "理解与支持"]
HARSH_CUES  = ["必须", "不得", "严禁", "否则", "追究", "终止服务", "责令", "强制", "保留权利", "责任自负", "违者", "处罚"]

def tone_friendliness_0to5(text: str) -> float:
    sents = split_sentences(text)
    n = max(len(sents), 1)
    polite_hits = sum(len(re.findall(re.escape(k), text)) for k in POLITE_CUES)
    harsh_hits  = sum(len(re.findall(re.escape(k), text)) for k in HARSH_CUES)
    balance = (polite_hits - harsh_hits) / n
    balance = max(-1.0, min(1.0, balance))   # clip to [-1, 1]
    return round((balance + 1.0) * 2.5, 3)   # [-1, 1] -> [0, 5]

# ====================== Compute and export
======================

def compute_all_metrics(text: str) -> dict:
    return {
        "character_count": character_count(text),
        "word_count": mixed_word_count(text),
        "sentence_count": sentence_count(text),
        "words_per_sentence": round(words_per_sentence(text), 6),
        "characters_per_word": round(characters_per_word(text), 6),
        "nested_sentence_ratio": round(nested_sentence_ratio(text), 6),
        "LY_readability_index": round(ly_readability_index(text), 6),
        "tone_friendliness_0to5": tone_friendliness_0to5(text)
    }

def read_docx_text(path: str) -> str:
    doc = Document(path)
    parts = [p.text for p in doc.paragraphs]
    return "\n".join(parts).strip()
```

```
def main():
    rows = []
    missing = []
    for i in FILE_RANGE:
        fname = f"{i}.docx"
        fpath = os.path.join(DOC_DIR, fname)
        if not os.path.exists(fpath):
            missing.append(fname)
            continue
        text = read_docx_text(fpath)
        metrics = compute_all_metrics(text)
        rows.append({"filename": fname, **metrics})

    if not rows:
        print("[INFO] No results generated. Please check paths and filenames.")
        if missing:
            print("[WARN] Missing files: " + ", ".join(missing))
        return

    wb = Workbook()
    ws = wb.active
    ws.title = "expr_metrics"

    headers = ["filename"] + [k for k in rows[0].keys() if k != "filename"]
    ws.append(headers)
    for r in rows:
        ws.append([r.get(h, "") for h in headers])

    for col_idx, h in enumerate(headers, start=1):
        ws.column_dimensions[ws.cell(row=1, column=col_idx).column_letter].width = max(len(h) + 2, 14)

    os.makedirs(os.path.dirname(OUT_XLSX), exist_ok=True)
    wb.save(OUT_XLSX)
    print(f"[OK] Metrics exported to Excel: {OUT_XLSX}")
    if missing:
        print("[WARN] The following files were not found and were skipped: " + ", ".join(missing))

if __name__ == "__main__":
    main()
```

Section 3. Statistical analysis

```
# ============================================================

# -*- coding: utf-8 -*-
"""
stat_analysis_wilcoxon_fdr.py

Wilcoxon pairwise comparison with FDR adjustment (BH), descriptive stats, and effect size r.
- Input: One Excel workbook with 3 sheets (three versions). Each sheet contains the same factor columns.
- Output: One Excel workbook with two sheets: "descriptives" and "wilcoxon".
- Tests are two-sided. Zero differences are KEPT (Pratt method), i.e., 0 is treated as a valid value.
- p values are also shown in formatted style: <0.001 displayed as "＜0.001".
- FDR (BH) applied across ALL pairwise tests within a given input workbook.
- Effect size r is computed as |Z| / sqrt(n), where n is the paired count (including zero differences).

Author: ChatGPT
"""

import pandas as pd
import numpy as np
from itertools import combinations
from scipy.stats import wilcoxon
from statsmodels.stats.multitest import multipletests
from typing import List, Optional

def _fmt_p(p: float) -> str:
    """Format p value with Chinese full-width less-than if < 0.001."""
    if pd.isna(p):
        return ""
    return "＜0.001" if p < 0.001 else f"{p:.3f}"

def analyze_file(
    in_path: str,
    out_path: str,
    alpha: float = 0.05,
    comparison_order: Optional[List[str]] = None,
) -> str:
    """
    Analyze one Excel workbook with 3 sheets (3 versions).
    Each sheet must contain the same factor columns (any number).
```

```
    Steps:
      1) Keep only common columns across the three sheets (preserving the first sheet's column order).
      2) Compute descriptives per Version × Factor:
           Mean, SD, Mean±SD, Mean[Mean-SD – Mean+SD]
      3) For each Factor, perform pairwise Wilcoxon (two-sided) across versions:
           Pratt method (keeps zero differences), compute W, raw p, Z, r=|Z|/√n
      4) Control FDR (BH) across all pairwise tests within this workbook.
      5) Add formatted p and FDR-p columns, and a significance flag for q<alpha.
      6) Export to Excel with sheets: "descriptives", "wilcoxon".
    """
    # Load all sheets
    xls = pd.ExcelFile(in_path)
    sheet_names = xls.sheet_names
    if comparison_order:
        missing = [s for s in comparison_order if s not in sheet_names]
        if missing:
            raise ValueError(f"comparison_order contains sheets not in workbook: {missing}")
        sheet_names = comparison_order

    data = {name: pd.read_excel(in_path, sheet_name=name) for name in sheet_names}

    # Common factor columns, keep the first sheet's column order
    common_cols = set.intersection(*[set(df.columns) for df in data.values()])
    first_cols = list(data[sheet_names[0]].columns)
    common_cols = [c for c in first_cols if c in common_cols]

    # Coerce to numeric (non-numeric → NaN)
    for k in data:
        df = data[k][common_cols].apply(pd.to_numeric, errors="coerce")
        data[k] = df

    # ===== Descriptives =====
    desc_rows = []
    for ver, df in data.items():
        for col in common_cols:
            s = df[col].dropna()
            n = int(s.shape[0])
            mean = float(s.mean()) if n > 0 else np.nan
            sd = float(s.std(ddof=1)) if n > 1 else np.nan
            lower = mean - sd if (n > 1 and pd.notna(mean) and pd.notna(sd)) else np.nan
            upper = mean + sd if (n > 1 and pd.notna(mean) and pd.notna(sd)) else np.nan
            desc_rows.append({
```

```
                    "Version": ver,
                    "Factor": col,
                    "n": n,
                    "Mean": mean,
                    "SD": sd,
                    "Mean±SD": (f"{mean:.2f}±{sd:.2f}" if pd.notna(mean) and pd.notna(sd)
else ""),
                    "Mean[Mean-SD – Mean+SD]": (f"{mean:.2f}[{lower:.2f}–{upper:.2f}]" if
pd.notna(mean) and pd.notna(sd) else "")
                })
    desc_df = pd.DataFrame(desc_rows)

    # ===== Wilcoxon pairwise (two-sided, Pratt) =====
    wilcoxon_rows = []
    all_pvals = []
    pairs = list(combinations(sheet_names, 2))

    for a, b in pairs:
        dfa = data[a]
        dfb = data[b]
        for col in common_cols:
            x = dfa[col]
            y = dfb[col]
            valid = ~(x.isna() | y.isna())
            xv = x[valid].to_numpy()
            yv = y[valid].to_numpy()
            n_pairs = int(len(xv))   # paired count including zero differences

            W_stat = np.nan
            p_raw = np.nan
            Z_val = np.nan
            r_eff = np.nan

            if n_pairs == 0:
                pass   # remain NaN
            else:
                diff = xv - yv
                if np.all(diff == 0):
                    W_stat, p_raw, Z_val, r_eff = 0.0, 1.0, 0.0, 0.0
                else:
                    try:
                        res = wilcoxon(xv, yv, alternative="two-sided",
zero_method="pratt")
                        W_stat = float(res.statistic)
```

```
                p_raw = float(res.pvalue)
                mean_T = n_pairs * (n_pairs + 1) / 4.0
                sd_T = np.sqrt(n_pairs * (n_pairs + 1) * (2 * n_pairs + 1) / 24.0)
                Z_val = (W_stat - mean_T) / sd_T if sd_T > 0 else 0.0
                r_eff = abs(Z_val) / np.sqrt(n_pairs) if n_pairs > 0 else np.nan
            except Exception:
                pass

        wilcoxon_rows.append({
            "Factor": col,
            "Comparison": f"{a} vs {b}",
            "n": n_pairs,
            "W statistic": W_stat,
            "Z value": Z_val,
            "r": r_eff,
            "p value": p_raw
        })
        all_pvals.append(p_raw)

    wilcoxon_df = pd.DataFrame(wilcoxon_rows)

    # ===== FDR (BH) across all tests in this workbook =====
    p_array = np.array(all_pvals, dtype=float)
    adj_p = np.full_like(p_array, np.nan, dtype=float)
    mask = ~np.isnan(p_array)
    if mask.sum() > 0:
        _, p_adj, _, _ = multipletests(p_array[mask], alpha=alpha, method="fdr_bh")
        adj_p[mask] = p_adj
    wilcoxon_df["FDR-p"] = adj_p

    # Significance flag after FDR
    wilcoxon_df["FDR-significant (q<0.05)"] = (wilcoxon_df["FDR-p"] < alpha).map({True:
"Yes", False: "No"})

    # Pretty p-values
    wilcoxon_df["p value (fmt)"] = wilcoxon_df["p value"].apply(_fmt_p)
    wilcoxon_df["FDR-p (fmt)"] = wilcoxon_df["FDR-p"].apply(_fmt_p)

    # Reorder columns
    wilcoxon_df = wilcoxon_df[[
        "Factor", "Comparison", "n", "W statistic", "Z value", "r",
        "p value", "p value (fmt)", "FDR-p", "FDR-p (fmt)", "FDR-significant (q<0.05)"
    ]]
```

```python
    # ===== Export =====
    with pd.ExcelWriter(out_path, engine="xlsxwriter") as writer:
        desc_df.to_excel(writer, sheet_name="descriptives", index=False)
        wilcoxon_df.to_excel(writer, sheet_name="wilcoxon", index=False)

    return out_path

if __name__ == "__main__":
    # Example batch usage:
    # Put S.xlsx, E.xlsx, O.xlsx in the same folder and run:
    #     python stat_analysis_wilcoxon_fdr.py
    inputs = ["S.xlsx", "E.xlsx", "O.xlsx"]
    outputs = ["S_Wilcoxon_FDR.xlsx", "E_Wilcoxon_FDR.xlsx", "O_Wilcoxon_FDR.xlsx"]
    for inp, outp in zip(inputs, outputs):
        try:
            print(analyze_file(inp, outp))
        except Exception as e:
            print(f"Error processing {inp}: {e}")
```

Section 4. Linear mixed-effects model of content quality

```
# ============================================================

# -*- coding: utf-8 -*-
import warnings
import numpy as np
import pandas as pd
import statsmodels.formula.api as smf
from statsmodels.tools.sm_exceptions import ConvergenceWarning

# Suppress convergence warnings from mixed linear models
warnings.simplefilter('ignore', ConvergenceWarning)

# === Load data ===
# Ensure the Excel file contains columns: version, rater, text, E, S, O, A
df = pd.read_excel("---")

# Four scoring dimensions
dimensions = ['E', 'S', 'O', 'A']

# Three pairwise comparisons (IMPORTANT: spellings must match the data, e.g., ChatGPT/ChaGPT)
comparisons = [
    ("Original", "ChatGPT"),
    ("ChatGPT", "Grok"),
    ("Original", "Grok")
]

# Helper: safely get the parameter name (compatible with C(version) or version naming)
def _get_param_name(fit, level):
    cand = [f"C(version)[T.{level}]", f"version[T.{level}]"]
    for name in cand:
        if name in fit.params.index:
            return name
    return None

results = []

for dim in dimensions:
    for v1, v2 in comparisons:
        sub = df[df['version'].isin([v1, v2])].copy()

        # Skip if the subset contains fewer than two versions
        if sub['version'].nunique() < 2:
```

```
        results.append({
            "Dimension": dim, "Comparison": f"{v2} vs {v1}",
            "Beta": np.nan, "SE": np.nan, "95% CI": "[]", "p-value": np.nan,
            "Var(rater)": np.nan, "Var(text)": np.nan, "Var(resid)": np.nan,
            "ICC": np.nan, "R²_marginal": np.nan, "R²_conditional": np.nan,
            "Note": "Skipped: only one version present in subset"
        })
        continue

    # —— Key step: restrict the categorical to these two levels in order ——
    # Option A: remove unused categories and reorder
    sub['version'] = sub['version'].astype('category')
    sub['version'] = sub['version'].cat.remove_unused_categories()
    sub['version'] = sub['version'].cat.reorder_categories([v1, v2], ordered=True)
    # Option B (equivalent): directly set categories
    # sub['version'] = pd.Categorical(sub['version'], categories=[v1, v2], ordered=True)

    # 0/1 coding (v1=0, v2=1), used for a simple R²  approximation
    sub['version_code'] = sub['version'].cat.codes

    try:
        # Mixed-effects model: rater as group; text as variance component
        model = smf.mixedlm(f"{dim} ~ version", sub, groups=sub["rater"],
                            re_formula="~1", vc_formula={"text": "0 + C(text)"})
        fit = model.fit(method='lbfgs', reml=False)
    except Exception as e:
        results.append({
            "Dimension": dim, "Comparison": f"{v2} vs {v1}",
            "Beta": np.nan, "SE": np.nan, "95% CI": "[]", "p-value": np.nan,
            "Var(rater)": np.nan, "Var(text)": np.nan, "Var(resid)": np.nan,
            "ICC": np.nan, "R²_marginal": np.nan, "R²_conditional": np.nan,
            "Note": f"Model fitting failed: {e}"
        })
        continue

    # Fixed effects
    pname = _get_param_name(fit, v2)
    if pname is None:
        coef = se = pval = np.nan
    else:
        coef = float(fit.params.get(pname, np.nan))
        se   = float(fit.bse.get(pname, np.nan))
        pval = float(fit.pvalues.get(pname, np.nan))
```

```
        ci_low    = coef - 1.96 * se if np.isfinite(coef) and np.isfinite(se) else np.nan
        ci_high = coef + 1.96 * se if np.isfinite(coef) and np.isfinite(se) else np.nan

        # Random-effect variances
        try:
            var_rater = float(fit.cov_re.iloc[0, 0])
        except Exception:
            var_rater = np.nan

        try:
            # vcomp may have one entry per text level; take the mean for a concise
summary
            var_text = float(np.nanmean(fit.vcomp)) if hasattr(fit, "vcomp") and
len(fit.vcomp) > 0 else np.nan
        except Exception:
            var_text = np.nan

        try:
            var_resid = float(fit.scale)
        except Exception:
            var_resid = np.nan

        # Total variance
        parts = [v for v in [var_rater, var_text, var_resid] if np.isfinite(v)]
        total_var = float(np.nansum(parts)) if parts else np.nan

        # ICC (based on rater)
        icc = (var_rater / total_var) if (np.isfinite(var_rater) and total_var and total_var > 0)
else np.nan

        # R² approximation (fixed part variance via version_code * coef)
        coef_safe = coef if np.isfinite(coef) else 0.0
        var_fixed = float(np.var(sub['version_code'] * coef_safe, ddof=0))    # ddof=0:
constant -> 0, no NaN
        if np.isfinite(total_var) and total_var > 0:
            r2_marginal = var_fixed / total_var
            r2_conditional = (var_fixed +
                              (var_rater if np.isfinite(var_rater) else 0.0) +
                              (var_text if np.isfinite(var_text) else 0.0)) / total_var
        else:
            r2_marginal = np.nan
            r2_conditional = np.nan

        results.append({
```

```
            "Dimension": dim,
            "Comparison": f"{v2} vs {v1}",
            "Beta": None if not np.isfinite(coef) else round(coef, 3),
            "SE": None if not np.isfinite(se) else round(se, 3),
            "95% CI": f"[{'' if not np.isfinite(ci_low) else round(ci_low, 3)}, "
                      f"{'' if not np.isfinite(ci_high) else round(ci_high, 3)}]",
            "p-value": (None if not np.isfinite(pval) else ("<.001" if pval < 0.001 else round(pval, 4))),
            "Var(rater)": None if not np.isfinite(var_rater) else round(var_rater, 3),
            "Var(text)": None if not np.isfinite(var_text) else round(var_text, 3),
            "Var(resid)": None if not np.isfinite(var_resid) else round(var_resid, 3),
            "ICC": None if not np.isfinite(icc) else round(icc, 3),
            "R²_marginal": None if not np.isfinite(r2_marginal) else round(r2_marginal, 3),
            "R²_conditional": None if not np.isfinite(r2_conditional) else round(r2_conditional, 3),
        })

# Export results to Excel
result_df = pd.DataFrame(results)
result_df.to_excel("---", index=False)
print("Analysis complete. Results saved to '---'")
```

Section 5. Linear mixed-effects model of reading comprehension

```
# ============================================================
# -*- coding: utf-8 -*-
import warnings
import numpy as np
import pandas as pd
import statsmodels.formula.api as smf
from statsmodels.tools.sm_exceptions import ConvergenceWarning

# Suppress convergence warnings from mixed linear models
warnings.simplefilter('ignore', ConvergenceWarning)

# === Load data ===
# Ensure the Excel file contains the following columns:
# version, rater, text, Comprehensibility, Clarity, Trustworthiness,
# Friendliness, Professionalism, Acceptability, Aggregate
df = pd.read_excel("---")

# Seven evaluation dimensions (including overall aggregate score)
dimensions = [
    'Comprehensibility',    # comprehension / reading comprehension
    'Clarity',
    'Trustworthiness',
    'Friendliness',
    'Professionalism',
    'Acceptability',
    'Aggregate'
]

# Three pairwise comparisons (IMPORTANT: version names must match the dataset, e.g., ChatGPT vs ChaGPT)
comparisons = [
    ("Original", "ChatGPT"),
    ("ChatGPT", "Grok"),
    ("Original", "Grok")
]

# Helper function: safely get the parameter name (compatible with C(version) or version naming)
def _get_param_name(fit, level):
    cand = [f"C(version)[T.{level}]", f"version[T.{level}]"]
    for name in cand:
        if name in fit.params.index:
            return name
```

```
        return None

results = []

for dim in dimensions:
    for v1, v2 in comparisons:
        sub = df[df['version'].isin([v1, v2])].copy()

        # Skip if the subset contains only one version
        if sub['version'].nunique() < 2:
            results.append({
                "Dimension": dim, "Comparison": f"{v2} vs {v1}",
                "Beta": np.nan, "SE": np.nan, "95% CI": "[]", "p-value": np.nan,
                "Var(rater)": np.nan, "Var(text)": np.nan, "Var(resid)": np.nan,
                "ICC": np.nan, "R²_marginal": np.nan, "R²_conditional": np.nan,
                "Note": "Skipped: only one version present in subset"
            })
            continue

        # —— Key fix: restrict the categorical variable to exactly these two levels in order
——
        # Option A: remove unused categories then reorder
        sub['version'] = sub['version'].astype('category')
        sub['version'] = sub['version'].cat.remove_unused_categories()
        sub['version'] = sub['version'].cat.reorder_categories([v1, v2], ordered=True)
        # Option B (equivalent): directly specify categories
        # sub['version'] = pd.Categorical(sub['version'], categories=[v1, v2], ordered=True)

        # Encode as 0/1 (v1=0, v2=1), used for simple R² calculation
        sub['version_code'] = sub['version'].cat.codes

        try:
            # Mixed-effects model: rater as grouping factor, text as variance component
            model = smf.mixedlm(f"{dim} ~ version", sub, groups=sub["rater"],
                                re_formula="~1", vc_formula={"text": "0 + C(text)"})
            fit = model.fit(method='lbfgs', reml=False)
        except Exception as e:
            results.append({
                "Dimension": dim, "Comparison": f"{v2} vs {v1}",
                "Beta": np.nan, "SE": np.nan, "95% CI": "[]", "p-value": np.nan,
                "Var(rater)": np.nan, "Var(text)": np.nan, "Var(resid)": np.nan,
                "ICC": np.nan, "R²_marginal": np.nan, "R²_conditional": np.nan,
                "Note": f"MODEL_FAIL: {e}"
            })
```

```
            continue

        # Fixed effects
        pname = _get_param_name(fit, v2)
        if pname is None:
            coef = se = pval = np.nan
        else:
            coef = float(fit.params.get(pname, np.nan))
            se   = float(fit.bse.get(pname, np.nan))
            pval = float(fit.pvalues.get(pname, np.nan))

        ci_low  = coef - 1.96 * se if np.isfinite(coef) and np.isfinite(se) else np.nan
        ci_high = coef + 1.96 * se if np.isfinite(coef) and np.isfinite(se) else np.nan

        # Random-effect variances
        try:
            var_rater = float(fit.cov_re.iloc[0, 0])
        except Exception:
            var_rater = np.nan

        try:
            # vcomp may generate one column per text level; take the mean as a summary
            var_text = float(np.nanmean(fit.vcomp)) if hasattr(fit, "vcomp") and
len(fit.vcomp) > 0 else np.nan
        except Exception:
            var_text = np.nan

        try:
            var_resid = float(fit.scale)
        except Exception:
            var_resid = np.nan

        # Total variance
        parts = [v for v in [var_rater, var_text, var_resid] if np.isfinite(v)]
        total_var = float(np.nansum(parts)) if parts else np.nan

        # ICC (based on rater variance)
        icc = (var_rater / total_var) if (np.isfinite(var_rater) and total_var and total_var > 0)
else np.nan

        # R²  (approximation: variance of version_code * coef as fixed part)
        coef_safe = coef if np.isfinite(coef) else 0.0
        var_fixed = float(np.var(sub['version_code'] * coef_safe, ddof=0))   # ddof=0
ensures constant -> 0
```

```
        if np.isfinite(total_var) and total_var > 0:
            r2_marginal = var_fixed / total_var
            r2_conditional = (var_fixed +
                              (var_rater if np.isfinite(var_rater) else 0.0) +
                              (var_text if np.isfinite(var_text) else 0.0)) / total_var
        else:
            r2_marginal = np.nan
            r2_conditional = np.nan

        results.append({
            "Dimension": dim,
            "Comparison": f"{v2} vs {v1}",
            "Beta": None if not np.isfinite(coef) else round(coef, 3),
            "SE": None if not np.isfinite(se) else round(se, 3),
            "95% CI": f"[{'' if not np.isfinite(ci_low) else round(ci_low, 3)}, "
                      f"{'' if not np.isfinite(ci_high) else round(ci_high, 3)}]",
            "p-value": (None if not np.isfinite(pval) else ("<.001" if pval < 0.001 else round(pval, 4))),
            "Var(rater)": None if not np.isfinite(var_rater) else round(var_rater, 3),
            "Var(text)": None if not np.isfinite(var_text) else round(var_text, 3),
            "Var(resid)": None if not np.isfinite(var_resid) else round(var_resid, 3),
            "ICC": None if not np.isfinite(icc) else round(icc, 3),
            "R²_marginal": None if not np.isfinite(r2_marginal) else round(r2_marginal, 3),
            "R²_conditional": None if not np.isfinite(r2_conditional) else round(r2_conditional, 3),
        })

# Export results
result_df = pd.DataFrame(results)
result_df.to_excel("---", index=False)
print("Analysis complete. Results saved to '---'")
```
