# Supplementary material for: Optimization of University Counseling Consent Forms With Large Language Models: Multidimensional Comparative Evaluation
Source: J Med Internet Res. 2026 Apr 1;28:e86502. doi: 10.2196/86502 (PMC13043017; doi:10.2196/86502)
Supplement: Multimedia Appendix 5 [file jmir-v28-e86502-s005.pdf]

Supplementary Data S1. Table of detailed ratings by expert 01 for all documents

Version	Indicator	Dimension	Document ID																																
			01	02	03	04	05	06	07	08	09	10	11	12	13	14	15	16	17	18	19	20	21	22	23	24	25	26	27	28	29	30	31	32	33
Original	Confidentiality	Existence	1	1	1	0	1	1	1	1	1	1	1	1	1	1	1	0	1	1	1	1	1	1	1	1	1	1	1	1	1	1	1	1	1
		Specificity	1	0	2	1	1	1	1	0	2	0	1	0	0	1	0	1	0	1	1	1	0	2	1	0	1	0	1	1	0	0	0	1	1
		Operability	0	1	2	1	1	1	2	1	1	1	2	2	0	1	2	1	2	1	2	1	1	2	2	2	0	1	1	2	2	1	1	0	1
		Total	2	2	5	2	3	3	4	2	4	2	4	3	1	3	3	2	3	3	4	3	2	5	4	3	2	2	3	4	3	2	2	2	3
	Exceptions to confidentiality	Existence	1	0	1	1	1	0	1	1	1	0	0	1	1	1	1	0	1	1	1	0	0	1	1	1	0	0	1	1	1	0	1	1	1
		Specificity	2	1	2	1	2	1	0	1	1	0	0	2	0	1	1	0	1	1	1	0	1	1	1	1	0	0	0	1	1	0	2	1	1
		Operability	1	1	2	2	1	1	2	1	1	1	1	2	1	1	1	1	2	1	2	1	1	2	2	2	1	1	1	2	2	1	2	2	1
		Total	4	2	5	4	4	2	3	3	3	1	1	5	2	3	3	1	4	3	4	1	2	4	4	4	1	1	2	4	4	1	5	4	3
	Client rights	Existence	1	1	1	1	1	1	1	1	1	1	1	1	1	1	1	1	1	1	1	1	1	1	0	1	1	0	1	1	1	1	1	1	0
		Specificity	1	0	1	1	0	2	1	0	2	0	0	0	1	2	1	0	1	1	1	0	0	1	0	1	1	0	1	0	2	2	1	2	1
		Operability	2	1	2	1	2	1	2	1	1	1	1	2	1	0	1	1	2	1	2	1	1	2	1	2	0	1	0	2	2	1	2	1	1
		Total	4	2	4	3	3	4	4	2	4	2	2	3	3	3	3	2	4	3	4	2	2	4	1	4	2	1	2	3	5	4	4	4	2
	Guardian consent	Existence	1	1	1	0	1	1	0	1	0	0	1	0	1	0	0	0	1	1	1	0	1	1	1	0	1	0	0	0	0	0	1	1	1
		Specificity	2	1	2	1	2	1	1	1	1	1	2	1	1	1	1	1	1	2	1	1	2	2	1	0	1	1	1	1	1	0	1	1	1
		Operability	1	1	2	0	1	1	1	1	1	1	1	1	0	0	1	1	2	0	2	1	1	2	2	1	1	1	1	1	1	1	2	1	1
		Total	4	3	5	1	4	3	2	3	2	2	4	2	2	1	2	2	4	3	4	2	4	5	4	1	3	2	2	2	2	1	4	3	3
	Goals and scope	Existence	0	0	1	0	1	0	0	1	0	0	0	1	1	0	1	0	1	1	1	0	1	1	1	1	0	0	1	0	1	0	1	1	1
		Specificity	1	1	1	0	2	1	0	2	1	0	0	1	0	1	1	0	1	1	1	0	0	0	2	1	0	0	0	1	2	0	2	0	1
		Operability	1	1	2	1	1	1	1	2	1	1	1	2	1	0	1	1	1	1	2	0	1	2	2	2	1	1	0	1	2	1	2	2	1
		Total	2	2	4	1	4	2	1	5	2	1	1	4	2	1	3	1	3	3	4	0	2	3	5	4	1	1	1	2	5	1	5	3	3
	Format and frequency	Existence	1	0	1	1	1	1	1	1	1	1	1	1	1	0	1	0	1	0	1	1	0	1	1	1	0	1	1	0	1	1	1	0	1
		Specificity	2	0	2	2	1	2	1	2	1	2	0	2	2	1	2	1	2	1	2	1	0	0	1	2	1	1	1	1	2	1	2	0	2
		Operability	0	1	1	1	0	1	2	2	1	1	1	2	1	1	1	1	2	1	2	1	0	1	2	2	1	2	1	1	2	1	2	1	1
		Total	3	1	4	4	2	4	4	5	3	4	2	5	4	2	4	2	5	2	5	3	0	2	4	5	2	4	3	2	5	3	5	1	4
	Fees and cancellation policy	Existence	1	0	1	0	1	1	1	1	1	1	1	1	1	1	0	1	1	1	1	0	0	1	1	1	1	0	1	1	1	0	1	1	1
		Specificity	2	1	1	1	0	1	0	0	0	1	2	1	2	1	1	1	1	1	2	0	0	0	1	1	2	0	0	1	1	1	0	1	1
		Operability	1	1	2	1	1	1	2	1	1	1	1	1	1	1	1	1	1	2	2	1	1	2	2	2	2	1	2	2	2	1	2	1	1
		Total	4	2	4	2	2	3	3	2	2	3	4	3	4	3	2	3	3	4	5	1	1	3	4	4	5	1	3	4	4	2	3	3	3
	Recording methods	Existence	1	1	1	1	1	0	1	1	1	1	0	1	0	1	0	1	1	1	0	1	1	1	1	1	1	0	0	1	1	0	1	1	1
		Specificity	1	1	1	1	1	0	1	2	1	1	1	1	1	2	1	2	2	1	0	1	1	1	2	1	1	1	0	1	2	0	1	1	1

	Operability	2	1	2	0	1	1	2	2	1	1	1	2	0	1	0	1	2	1	1	1	1	2	2	2	1	1	1	2	2	1	2	1	1
	Total	4	3	4	2	3	1	4	5	3	3	2	4	1	4	1	4	5	3	1	3	3	4	5	4	3	2	1	4	5	1	4	3	3
Authorization	Existence	1	1	1	1	0	0	1	1	1	0	0	1	1	0	0	1	1	1	1	1	0	1	1	1	1	1	1	0	1	1	1	0	1
and revocation	Specificity	1	0	1	0	1	0	0	0	1	0	1	0	0	0	1	1	0	0	0	1	1	1	0	0	0	0	0	0	0	0	0	1	0
	Operability	1	1	2	0	1	1	1	1	1	1	1	2	1	0	0	0	2	1	2	1	1	2	2	2	1	1	1	1	2	1	2	1	1
	Total	3	2	4	1	2	1	2	2	3	1	2	3	2	0	1	2	3	2	3	3	2	4	3	3	2	2	2	1	3	2	3	2	2
Crisis	Existence	1	0	1	1	1	1	1	1	1	1	1	0	1	1	1	1	1	1	1	1	1	1	1	1	1	1	0	1	1	1	1	1	1
procedures	Specificity	1	0	1	1	1	1	0	2	0	0	0	0	0	0	1	0	1	2	1	0	1	0	0	2	1	0	0	0	0	1	0	0	0
	Operability	1	0	2	1	0	1	2	1	1	0	1	1	1	1	1	1	2	0	1	1	0	2	2	2	1	1	1	2	2	1	2	1	0
	Total	3	0	4	3	2	3	3	4	2	1	2	1	2	2	3	2	4	3	3	2	2	3	3	5	3	2	1	3	3	3	3	2	1
Complaints	Existence	0	0	1	0	0	0	0	0	0	0	0	0	1	0	1	0	0	1	0	0	0	0	0	1	1	0	0	0	0	0	0	1	0
and appeals	Specificity	1	1	1	0	1	1	0	0	1	1	1	0	0	0	1	1	0	0	1	0	0	1	0	0	0	0	0	0	0	0	0	1	0
	Operability	0	1	2	1	1	1	1	1	1	1	1	1	1	0	1	1	1	1	1	1	1	1	1	2	1	1	1	1	1	1	1	1	1
	Total	1	2	4	1	2	2	1	1	2	2	2	1	2	0	3	2	1	2	2	1	1	2	1	3	2	1	1	1	1	1	1	3	1
Data	Existence	0	0	0	1	0	0	0	0	0	0	0	1	0	1	0	0	0	0	0	0	0	0	1	0	0	0	1	0	0	0	1	1	0
protection	Specificity	1	0	0	0	1	0	0	1	0	0	0	0	1	1	0	1	0	0	0	0	0	0	1	0	0	0	0	0	1	0	1	0	0
	Operability	1	1	1	1	1	1	1	1	1	0	1	2	1	1	1	1	1	1	1	1	1	1	1	1	1	0	2	1	1	1	2	2	1
	Total	2	1	1	2	2	1	1	2	1	0	1	3	2	3	1	2	1	1	1	1	1	1	3	1	1	0	3	1	2	1	4	3	1
Disclaimer of	Existence	0	0	0	0	0	0	0	0	0	0	0	0	0	0	0	0	0	0	0	0	0	0	0	1	0	0	0	0	0	0	1	0	1
boundaries	Specificity	1	1	1	0	1	1	1	1	1	0	1	1	1	1	1	0	1	1	1	1	1	1	1	2	1	1	1	1	1	1	1	1	2
	Operability	1	1	1	1	1	1	1	1	1	1	1	1	1	1	1	1	1	1	1	1	1	1	1	2	1	0	1	1	1	1	1	1	1
	Total	2	2	2	1	2	2	2	2	2	1	2	2	2	2	2	1	2	2	2	2	2	2	2	5	2	1	2	2	2	2	3	2	4
Language	Existence	0	0	0	1	1	0	0	0	0	0	0	0	0	0	0	1	0	0	0	0	0	1	0	0	0	0	0	0	0	0	0	0	0
clarity	Specificity	1	0	0	0	0	0	0	0	0	0	0	0	0	0	0	0	0	1	1	0	0	0	0	1	0	0	0	0	0	0	0	0	0
	Operability	1	1	1	1	1	1	0	1	1	1	1	1	1	1	1	1	1	1	1	1	1	1	1	1	1	1	1	1	1	1	1	1	1
	Total	2	1	1	2	2	1	0	1	1	1	1	1	1	1	1	2	1	2	2	1	1	2	1	2	1	1	1	1	1	1	1	1	1
Voluntariness	Existence	0	1	0	0	0	1	0	0	1	1	1	0	1	0	0	1	0	0	1	0	1	1	0	1	1	1	0	0	0	1	1	0	0
	Specificity	0	0	0	0	0	0	0	0	0	0	1	0	0	0	0	0	0	0	0	0	0	1	0	1	0	0	1	0	0	0	0	0	0
	Operability	1	1	1	1	1	1	1	0	1	1	1	1	1	0	1	1	1	1	1	1	0	1	1	1	1	1	1	1	1	1	2	1	1
	Total	1	2	1	1	1	2	1	0	2	2	3	1	2	0	1	2	1	1	2	1	1	3	1	3	2	2	2	1	1	2	3	1	1
Client	Existence	0	1	0	0	1	0	0	1	1	0	0	1	1	1	1	1	1	0	1	0	0	1	0	1	0	0	0	0	1	0	1	1	1
obligations	Specificity	1	1	0	0	0	0	0	0	0	0	0	0	1	0	0	0	0	0	0	0	0	0	0	1	1	0	0	0	0	0	0	0	0
	Operability	1	1	1	1	1	1	1	1	1	1	1	2	1	1	1	1	2	1	1	1	0	2	1	2	1	1	1	1	2	1	2	1	1

		Total	2	3	1	1	2	1	1	2	2	1	1	3	3	2	2	2	3	1	2	1	0	3	1	4	2	1	1	1	3	1	3	2	2
	Counseling limitations	Existence	0	0	0	1	0	0	0	0	0	0	0	1	0	0	0	0	0	0	0	0	0	1	0	0	0	0	0	0	0	0	0	0	0
		Specificity	0	0	0	0	1	0	0	1	0	0	1	0	0	0	1	0	0	0	0	0	0	1	0	0	1	0	0	0	0	0	0	0	0
		Operability	1	1	1	1	1	1	1	1	1	1	1	1	1	1	0	1	1	1	1	0	1	2	1	1	1	1	1	1	1	1	1	1	1
		Total	1	1	1	2	2	1	1	2	1	1	2	2	1	1	1	1	1	1	1	0	1	4	1	1	2	1	1	1	1	1	1	1	1
	Counselor qualifications	Existence	1	0	1	1	1	0	1	1	1	1	0	1	1	1	0	1	1	1	1	1	0	1	0	0	1	1	1	1	1	0	1	1	1
		Specificity	0	0	1	0	0	0	0	0	0	0	0	0	0	0	0	0	0	0	0	0	0	0	0	1	0	0	0	1	0	0	0	0	0
		Operability	1	1	2	1	1	1	2	1	2	1	1	2	1	2	1	2	2	2	2	1	0	2	1	1	1	1	1	2	2	1	2	1	1
		Total	2	1	4	2	2	1	3	2	3	2	1	3	2	3	1	3	3	3	3	2	0	3	1	2	2	2	2	4	3	1	3	2	2
	Counseling modalities	Existence	0	1	1	0	1	0	0	0	0	0	0	0	1	0	0	0	0	0	0	0	0	0	0	0	1	0	1	0	0	0	0	0	0
		Specificity	0	1	1	1	0	1	0	0	0	0	0	1	1	0	0	1	0	1	0	0	1	0	0	0	0	0	0	1	0	0	0	0	0
		Operability	1	1	2	1	0	1	1	1	0	1	1	1	1	1	1	1	1	1	1	1	1	1	1	1	1	1	1	1	1	1	1	1	1
		Total	1	3	4	2	1	2	1	1	0	1	1	2	3	1	1	2	1	2	1	1	2	1	1	1	2	1	2	2	1	1	1	1	1
	Target population	Existence	1	0	1	0	0	0	1	1	1	1	1	1	1	1	1	1	1	1	1	0	0	1	0	0	1	0	0	1	1	1	1	1	1
		Specificity	2	0	0	0	0	0	0	2	2	1	0	1	1	0	2	1	1	1	1	0	0	0	1	0	1	1	0	1	1	0	0	1	1
		Operability	1	1	2	1	1	1	2	1	0	0	2	2	1	0	1	1	2	1	2	1	1	2	1	1	1	1	1	2	2	1	2	1	1
		Total	4	1	3	1	1	1	3	4	3	2	3	4	3	1	4	3	4	3	4	1	1	3	2	1	3	2	1	4	4	2	3	3	3
	Total score	Existence	11	8	14	10	13	7	10	13	12	9	8	13	15	10	9	10	13	12	13	7	7	16	10	13	12	6	10	8	12	7	16	13	13
		Specificity	21	9	18	10	15	13	6	15	14	7	11	11	12	12	15	11	12	15	14	6	8	12	12	15	12	5	6	11	14	6	11	11	12
		Operability	19	19	33	18	18	20	28	22	19	17	22	31	17	14	18	20	31	20	30	18	15	33	29	32	19	19	20	28	32	20	34	22	19
		Total	51	36	65	38	46	40	44	50	45	33	41	55	44	36	42	41	56	47	57	31	30	61	51	60	43	30	36	47	58	33	61	46	44
ChatGPT-5	Confidentiality	Existence	1	1	1	1	0	1	1	1	1	1	1	1	0	1	1	1	0	1	1	0	1	1	1	1	1	1	1	1	1	1	1	1	0
		Specificity	2	2	2	1	1	2	1	2	2	2	2	1	1	2	0	1	1	2	1	1	2	2	2	1	2	2	0	2	1	1	1	2	1
		Operability	0	1	2	1	1	1	2	1	1	1	1	2	1	1	1	1	1	2	2	0	1	2	2	2	1	1	1	2	2	1	2	1	1
		Total	3	4	5	3	2	4	4	4	4	4	4	4	2	4	2	3	2	5	4	1	4	5	5	4	4	4	2	5	4	3	4	4	2
	Exceptions to confidentiality	Existence	1	1	1	1	1	1	1	1	0	0	1	1	0	1	1	1	1	1	1	0	1	1	1	1	1	1	1	1	1	1	1	1	1
		Specificity	1	2	2	0	2	2	2	2	1	1	2	2	1	2	2	1	1	2	2	1	1	2	1	1	1	1	0	1	2	1	2	2	2
		Operability	2	1	2	1	1	1	2	1	1	1	1	2	1	1	1	1	2	2	2	1	1	2	2	2	1	2	1	2	2	1	2	1	2
		Total	4	4	5	2	4	4	5	4	2	2	4	5	2	4	4	3	4	5	5	2	3	5	4	4	3	4	2	4	5	3	5	4	5
	Client rights	Existence	1	1	1	1	1	1	0	0	0	1	1	1	1	1	1	1	1	0	0	1	1	1	1	1	1	1	1	1	1	1	1	1	0
		Specificity	2	1	2	2	0	0	1	1	1	0	1	1	0	1	2	1	1	1	1	1	1	2	1	2	2	2	1	0	1	2	1	2	1
		Operability	2	1	2	1	1	1	1	1	1	1	1	2	1	1	1	1	2	1	1	2	2	2	2	2	1	1	1	2	2	1	2	1	1
		Total	5	3	5	4	2	2	2	2	2	2	3	4	2	3	4	3	4	2	2	4	4	5	4	5	4	4	3	3	4	4	4	4	2

Guardian	Existence	0	1	1	0	1	1	0	1	1	0	1	0	1	0	1	1	1	1	1	1	1	1	1	1	1	0	0	0	1	0	1	1	1
consent	Specificity	1	2	2	1	0	2	1	2	2	1	2	0	2	1	1	1	2	2	2	0	2	2	2	2	1	1	0	1	2	1	2	2	2
	Operability	0	1	2	1	0	1	1	1	1	0	1	1	0	1	1	1	2	2	2	1	1	2	2	2	1	1	0	1	2	1	2	1	1
	Total	1	4	5	2	1	4	2	4	4	1	4	1	3	2	3	3	5	5	5	2	4	5	5	5	3	2	0	2	5	2	5	4	4
Goals and	Existence	1	0	1	0	1	1	1	1	1	0	1	1	1	1	1	1	1	1	1	1	1	1	1	1	0	1	1	0	1	0	0	1	1
scope	Specificity	1	1	2	0	2	2	1	0	1	0	2	1	2	2	2	1	1	0	1	1	0	0	2	1	1	0	0	1	0	0	1	1	1
	Operability	0	1	2	1	1	2	1	1	2	1	1	2	1	2	1	1	2	2	2	1	1	2	2	2	0	1	1	1	2	1	1	1	1
	Total	2	2	5	1	4	5	3	2	4	1	4	4	4	5	4	3	4	3	4	3	2	3	5	4	1	2	2	2	3	1	2	3	3
Format and	Existence	1	1	1	1	1	1	1	1	1	1	0	1	0	1	1	1	1	1	1	1	1	1	1	1	1	1	1	1	1	1	1	1	0
frequency	Specificity	1	0	2	1	1	2	1	0	0	1	1	2	1	1	2	1	1	2	1	1	1	0	2	1	2	1	2	1	2	0	2	0	1
	Operability	1	2	2	1	2	1	2	0	2	1	1	2	1	2	1	1	2	2	2	1	1	2	2	2	1	2	1	2	2	1	2	1	1
	Total	3	3	5	3	4	4	4	1	3	3	2	5	2	4	4	3	4	5	4	3	3	3	5	4	4	4	4	4	5	2	5	2	2
Fees and	Existence	1	1	0	1	1	1	1	0	1	0	1	1	1	1	1	1	1	1	1	1	0	1	1	1	1	1	1	1	1	1	1	1	1
cancellation	Specificity	1	1	1	0	2	1	1	1	1	0	1	1	2	0	1	0	1	2	1	1	1	1	1	1	1	1	1	2	1	1	1	2	1
policy	Operability	2	1	1	1	1	1	2	1	0	1	1	2	0	0	1	2	2	1	2	2	1	2	2	2	1	1	1	2	2	0	2	1	1
	Total	4	3	2	2	4	3	4	2	2	1	3	4	3	1	3	3	4	4	4	4	2	4	4	4	3	3	3	5	4	2	4	4	3
Recording	Existence	1	1	1	1	1	1	1	1	0	1	1	1	1	1	1	1	1	1	1	1	1	1	1	1	1	1	0	1	1	0	1	1	1
methods	Specificity	1	2	2	1	2	1	0	1	1	2	2	2	1	2	1	2	0	0	1	1	1	1	2	1	1	1	1	1	2	0	1	1	1
	Operability	2	1	1	1	0	1	2	2	1	1	1	2	0	1	2	2	2	2	2	1	1	2	2	2	1	1	1	1	2	1	2	1	1
	Total	4	4	4	3	3	3	3	4	2	4	4	5	2	4	4	5	3	3	4	3	3	4	5	4	3	3	2	3	5	1	4	3	3
Authorization	Existence	1	1	1	1	1	1	1	1	0	1	1	1	1	1	0	1	1	1	1	1	1	1	1	1	1	1	1	1	1	0	1	1	1
and revocation	Specificity	0	1	1	1	1	1	0	1	1	2	2	0	1	0	0	1	1	1	1	0	1	1	0	1	0	0	0	1	1	0	1	1	1
	Operability	1	1	1	1	0	1	2	1	1	1	1	2	1	1	1	1	2	1	2	1	1	2	2	2	1	1	0	2	2	1	2	1	1
	Total	2	3	3	3	2	3	3	3	2	4	4	3	3	2	1	3	4	3	4	2	3	4	3	4	2	2	1	4	4	1	4	3	3
Crisis	Existence	1	1	1	1	0	1	1	1	1	1	1	1	1	1	1	1	1	1	1	1	1	1	1	0	1	1	1	0	1	1	1	1	1
procedures	Specificity	2	0	1	1	1	1	0	1	0	0	2	2	1	0	1	0	1	2	0	0	0	0	1	1	2	0	1	0	1	0	1	0	1
	Operability	1	1	2	1	0	1	2	1	1	1	1	2	1	1	1	0	1	1	2	0	1	2	2	1	1	1	0	1	2	1	2	1	1
	Total	4	2	4	3	1	3	3	3	2	2	4	5	3	2	3	1	3	4	3	1	2	3	4	2	4	2	2	1	4	2	4	2	3
Complaints	Existence	0	1	1	0	0	0	0	1	1	0	0	0	0	0	0	0	0	1	0	0	1	0	1	0	0	1	0	0	0	0	1	0	0
and appeals	Specificity	1	2	1	1	1	1	0	1	1	1	1	0	1	1	0	1	1	1	0	1	1	1	0	1	0	1	0	1	1	0	1	1	1
	Operability	1	0	0	1	1	0	1	1	0	1	1	1	1	1	0	1	1	2	1	1	1	0	2	1	1	1	1	1	1	1	2	1	1
	Total	2	3	2	2	2	1	1	3	2	2	2	1	2	2	0	2	2	4	1	2	3	1	3	2	1	3	1	2	2	1	4	2	2
Data	Existence	1	1	1	0	0	0	0	0	0	0	0	0	0	0	0	0	0	0	1	0	0	0	1	0	0	1	0	0	0	0	1	0	0

protection	Specificity	1	2	2	1	1	0	0	1	1	0	1	1	0	1	1	1	0	0	1	0	1	0	0	0	1	0	1	0	1	1	0	0	0
	Operability	1	0	2	1	1	1	1	1	1	1	1	1	0	1	1	1	1	1	2	1	1	1	2	1	1	1	1	1	1	1	2	1	1
	Total	3	3	5	2	2	1	1	2	2	1	2	2	0	2	2	2	1	1	4	1	2	1	3	1	2	2	2	1	2	2	3	1	1
Disclaimer of boundaries	Existence	0	0	0	0	0	0	0	0	0	0	0	0	0	0	0	0	0	0	0	0	1	0	0	0	1	0	0	0	0	0	0	0	0
	Specificity	1	1	1	0	1	1	1	1	1	1	1	1	1	1	1	1	1	1	1	1	2	1	1	1	2	1	1	1	1	1	1	1	1
	Operability	1	1	1	1	1	1	1	0	1	1	1	1	1	1	1	1	1	1	1	1	1	1	1	1	1	1	1	1	1	1	1	1	1
	Total	2	2	2	1	2	2	2	1	2	2	2	2	2	2	2	2	2	2	2	2	4	2	2	2	4	2	2	2	2	2	2	2	2
Language clarity	Existence	1	0	0	1	0	0	0	0	0	0	0	0	0	0	0	0	0	0	0	1	0	0	0	0	0	0	1	0	0	0	0	0	1
	Specificity	1	0	0	0	0	0	1	0	0	1	0	0	0	0	1	0	0	0	0	1	0	0	0	0	0	0	0	0	0	0	0	0	0
	Operability	2	1	1	1	0	1	1	1	1	1	0	1	1	1	0	1	1	1	1	1	1	1	1	1	0	1	0	1	1	1	1	1	1
	Total	4	1	1	2	0	1	2	1	1	2	0	1	1	1	1	1	1	1	1	3	1	1	1	1	0	1	1	1	1	1	1	1	2
Voluntariness	Existence	1	1	1	0	0	1	1	0	1	1	1	1	0	1	1	1	1	1	1	1	1	0	0	1	1	1	0	1	1	1	1	1	1
	Specificity	1	0	0	0	0	1	0	0	1	0	0	0	0	0	1	0	0	1	0	0	0	0	0	0	0	0	0	0	0	0	0	0	0
	Operability	2	1	2	0	1	1	2	1	1	1	0	1	1	1	0	1	2	1	2	1	1	1	1	1	1	1	0	2	2	1	2	1	1
	Total	4	2	3	0	1	3	3	1	3	2	1	2	1	2	2	2	3	3	3	2	2	1	1	2	2	2	0	3	3	2	3	2	2
Client obligations	Existence	0	0	1	0	0	0	0	1	1	1	1	1	1	1	0	0	1	1	1	1	1	0	0	1	1	0	1	1	1	1	1	0	1
	Specificity	0	0	1	0	1	0	0	1	1	0	1	1	1	2	0	1	0	0	1	0	0	0	0	0	1	0	1	0	1	0	0	0	1
	Operability	1	1	2	1	1	1	1	1	1	1	1	2	1	1	1	1	2	2	2	0	1	1	1	2	1	1	1	2	1	1	2	1	1
	Total	1	1	4	1	2	1	1	3	3	2	3	4	3	4	1	2	3	3	4	1	2	1	1	3	3	1	3	3	3	2	3	1	3
Counseling limitations	Existence	1	1	0	0	0	0	0	0	0	0	0	0	0	0	0	0	1	1	0	0	0	0	0	0	0	0	0	0	0	0	0	1	0
	Specificity	0	1	0	0	0	1	0	0	0	1	0	0	0	0	0	0	0	0	0	0	0	0	0	1	0	0	0	0	0	0	0	0	0
	Operability	1	1	1	1	1	1	1	1	1	1	1	1	1	1	1	1	2	2	1	1	1	1	1	1	1	1	1	1	1	1	1	1	1
	Total	2	3	1	1	1	2	1	1	1	2	1	1	1	1	1	1	3	3	1	1	1	1	1	2	1	1	1	1	1	1	1	2	1
Counselor qualifications	Existence	1	1	1	1	1	1	1	1	1	1	1	0	1	1	0	1	1	1	1	1	1	1	1	1	0	1	1	1	1	0	1	1	1
	Specificity	1	1	0	0	0	0	1	0	0	0	0	0	0	0	0	0	0	0	0	0	0	0	0	0	0	0	0	1	0	0	0	0	0
	Operability	2	1	2	1	1	1	2	1	1	2	2	1	1	2	1	0	2	2	2	1	1	2	2	2	1	1	1	2	2	1	2	1	1
	Total	4	3	3	2	2	2	4	2	2	3	3	1	2	3	1	1	3	3	3	2	2	3	3	3	1	2	2	3	4	1	3	2	2
Counseling modalities	Existence	0	0	1	0	1	0	0	0	0	0	1	0	1	0	1	0	0	0	0	0	0	0	0	0	1	0	1	0	0	0	0	0	0
	Specificity	1	1	1	1	2	1	1	1	1	1	2	1	2	1	0	1	0	0	1	1	1	0	1	0	2	1	0	1	0	0	0	0	1
	Operability	1	1	2	1	2	1	1	1	1	1	2	1	1	1	1	1	1	1	1	1	1	1	1	1	1	1	2	1	1	1	1	1	1
	Total	2	2	4	2	5	2	2	2	2	2	5	2	4	2	2	2	1	1	2	2	2	1	2	1	4	2	3	2	1	1	1	1	2
Target population	Existence	1	0	0	0	0	0	1	0	1	1	1	1	1	1	1	1	1	1	1	0	0	1	1	1	1	0	1	1	1	0	1	1	1
	Specificity	2	0	0	0	0	0	0	1	2	1	1	1	1	0	2	0	1	1	0	0	0	1	1	0	0	0	0	0	1	1	1	1	1

		Operability	2	1	1	1	1	0	2	1	1	1	0	1	1	2	1	2	2	2	2	1	1	1	2	2	0	1	1	2	1	1	2	2	2
		Total	5	1	1	1	1	0	3	2	4	3	2	3	3	3	4	3	4	4	3	1	1	3	4	3	1	1	2	3	3	2	4	4	4
	Total score	Existence	15	14	15	10	10	12	11	11	11	10	14	12	11	13	12	13	14	15	14	12	14	12	14	13	14	13	13	11	14	8	15	14	12
		Specificity	21	20	23	11	18	19	12	17	18	15	24	17	18	17	18	14	13	18	15	11	15	14	17	15	19	12	9	13	19	9	16	16	17
		Operability	25	19	31	19	17	19	30	19	20	20	19	30	16	23	18	21	33	31	34	19	21	30	34	32	17	22	16	30	32	19	35	21	22
		Total	61	53	69	40	45	50	53	47	49	45	57	59	45	53	48	48	60	64	63	42	50	56	65	60	50	47	38	54	65	36	66	51	51
Grok-4	Confidentiality	Existence	1	0	1	1	1	1	1	1	1	0	1	1	1	1	1	1	1	1	1	1	1	1	1	1	1	0	1	1	1	1	1	1	1
		Specificity	2	1	2	2	2	2	1	1	2	1	1	1	2	2	2	2	2	1	2	2	2	2	1	1	2	1	1	1	1	0	1	2	2
		Operability	0	1	1	1	1	1	2	1	1	1	1	2	0	1	1	1	2	1	2	0	1	1	2	2	2	1	1	2	2	1	2	1	1
		Total	3	2	4	4	4	4	4	3	4	2	3	4	3	4	4	4	5	3	5	3	4	4	4	4	5	2	3	4	4	2	4	4	4
	Exceptions to confidentiality	Existence	1	0	1	0	1	1	1	1	1	1	1	1	0	1	1	1	1	0	1	1	1	1	1	1	1	1	1	1	1	1	1	1	1
		Specificity	1	1	2	1	1	1	1	1	2	2	2	2	1	2	2	1	2	1	2	1	2	2	2	2	2	2	1	2	2	1	2	2	2
		Operability	2	0	2	1	1	1	2	1	1	1	1	2	1	1	1	1	1	1	2	2	1	2	2	2	2	1	1	2	2	1	2	1	1
		Total	4	1	5	2	3	3	4	3	4	4	4	5	2	4	4	3	4	2	5	4	4	5	5	5	5	4	3	5	5	3	5	4	4
	Client rights	Existence	1	0	1	1	1	1	1	1	1	1	1	1	1	1	1	1	0	1	1	1	1	1	1	1	1	1	1	1	1	0	1	0	1
		Specificity	2	1	2	1	0	2	2	2	2	2	2	2	2	2	2	2	1	2	2	1	2	2	2	2	2	1	2	2	2	1	2	1	2
		Operability	2	1	2	1	1	1	2	1	1	1	1	2	0	1	0	1	1	2	2	1	2	2	1	2	2	1	1	2	2	1	2	1	1
		Total	5	2	5	3	2	4	5	4	4	4	4	5	3	4	3	4	2	5	5	3	5	5	4	5	5	3	4	5	5	2	5	2	4
	Guardian consent	Existence	0	1	1	0	1	1	0	0	0	1	1	0	1	0	1	0	1	1	1	0	1	1	0	0	1	0	0	0	1	0	1	1	1
		Specificity	1	2	1	1	1	2	1	0	1	2	2	1	2	1	2	0	1	1	0	1	1	2	1	1	2	1	1	1	0	1	1	2	2
		Operability	0	0	1	1	1	1	1	1	1	2	1	1	1	1	1	1	2	1	1	1	1	2	1	1	2	1	1	1	1	1	2	1	1
		Total	1	3	3	2	3	4	2	1	2	5	4	2	4	2	4	1	4	3	2	2	3	5	2	2	5	2	2	2	2	2	4	4	4
	Goals and scope	Existence	1	1	1	1	1	0	1	0	1	1	1	1	1	1	1	1	1	1	1	1	1	1	1	1	1	1	1	1	1	0	1	1	1
		Specificity	1	1	2	2	2	1	1	1	2	2	1	2	1	2	2	2	2	1	1	2	2	1	2	2	1	1	2	2	2	1	1	0	2
		Operability	0	1	2	1	1	1	2	1	1	1	1	2	0	1	1	2	2	1	2	0	1	2	0	2	2	1	0	2	2	1	2	1	1
		Total	2	3	5	4	4	2	4	2	4	4	3	5	2	4	4	5	5	3	4	3	4	4	3	5	4	3	3	5	5	2	4	2	4
	Format and frequency	Existence	1	1	1	0	1	1	1	1	1	1	0	1	1	1	1	1	1	1	1	1	1	1	1	1	1	0	1	1	0	1	1	0	1
		Specificity	2	2	2	1	2	2	2	2	2	2	1	1	2	2	2	2	2	2	2	1	0	2	2	2	2	1	1	2	1	1	2	0	2
		Operability	2	1	2	0	1	0	1	0	1	1	0	2	1	1	1	1	2	1	2	1	0	1	2	2	2	1	2	2	1	1	2	1	0
		Total	5	4	5	1	4	3	4	3	4	4	1	4	4	4	4	4	5	4	5	3	1	4	5	5	5	2	4	5	2	3	5	1	3
	Fees and cancellation	Existence	1	1	1	1	0	0	1	1	1	1	0	1	1	1	1	0	1	1	1	1	1	1	1	1	1	1	1	1	1	1	1	0	1
		Specificity	2	0	1	1	1	1	2	1	1	1	1	1	1	1	1	1	1	0	1	0	1	1	1	1	1	2	2	0	1	1	1	0	2

policy	Operability	1	0	2	1	1	1	2	1	1	1	0	2	1	1	1	1	1	2	2	1	1	2	2	1	2	1	1	2	1	1	2	1	1
	Total	4	1	4	3	2	2	5	3	3	3	1	4	3	3	3	2	3	3	4	2	3	4	4	3	4	4	4	3	3	3	4	1	4
Recording	Existence	1	1	1	1	0	1	1	1	1	1	0	1	1	1	1	1	1	1	1	1	1	1	0	1	1	1	0	1	1	0	1	1	1
methods	Specificity	2	2	1	1	1	1	2	1	1	2	0	2	2	2	1	2	1	1	1	1	1	1	1	2	1	2	1	2	2	0	1	2	0
	Operability	1	1	2	1	0	1	2	1	0	1	1	1	0	2	0	1	2	1	2	1	1	1	1	2	2	1	1	2	2	1	2	1	1
	Total	4	4	4	3	1	3	5	3	2	4	1	4	3	5	2	4	4	3	4	3	3	3	2	5	4	4	2	5	5	1	4	4	2
Authorization	Existence	1	1	1	1	1	0	1	1	1	0	1	0	1	1	1	1	1	0	1	1	1	1	1	1	1	1	1	1	1	1	1	1	1
and revocation	Specificity	1	1	1	0	1	1	1	0	1	1	1	1	1	0	1	0	1	1	0	1	0	1	0	2	1	1	0	1	0	0	0	1	1
	Operability	1	1	1	1	1	1	2	1	1	1	1	1	0	0	1	0	1	1	2	1	0	2	1	2	2	1	1	2	2	1	1	1	1
	Total	3	3	3	2	3	2	4	2	3	2	3	2	2	1	3	1	3	2	3	3	1	4	2	5	4	3	2	4	3	2	2	3	3
Crisis	Existence	0	0	1	0	1	1	1	1	1	1	1	1	1	1	1	1	1	1	1	1	0	1	0	1	1	0	1	1	0	1	1	1	1
procedures	Specificity	0	0	1	1	1	1	2	1	0	1	2	1	1	0	1	0	0	2	0	0	0	1	0	2	1	0	2	0	1	1	2	1	1
	Operability	1	1	2	1	1	1	1	1	0	0	1	2	0	1	1	1	2	0	2	1	1	2	1	2	2	1	1	2	1	1	1	1	0
	Total	1	1	4	2	3	3	4	3	1	2	4	4	2	2	3	2	3	3	3	2	1	4	1	5	4	1	4	3	2	3	4	3	2
Complaints	Existence	1	0	1	0	0	1	0	0	0	0	0	1	0	0	1	0	1	0	0	1	0	0	1	1	0	1	1	0	0	0	1	0	0
and appeals	Specificity	1	1	1	0	1	2	1	0	1	1	1	0	0	0	1	1	0	0	1	1	0	1	0	1	1	1	1	1	0	0	0	1	0
	Operability	1	1	2	1	1	1	1	0	1	0	1	2	1	0	1	1	2	1	1	1	0	1	2	2	1	1	1	1	1	1	2	1	1
	Total	3	2	4	1	2	4	2	0	2	1	2	3	1	0	3	2	3	1	2	3	0	2	3	4	2	3	3	2	1	1	3	2	1
Data	Existence	0	1	0	1	1	1	1	0	1	1	0	1	0	0	1	1	1	1	0	1	1	0	1	0	1	1	1	0	1	0	1	1	0
protection	Specificity	0	1	0	0	1	1	1	1	0	2	0	2	1	1	0	2	0	0	0	2	1	1	1	0	2	1	2	1	1	0	0	1	0
	Operability	1	0	1	1	0	1	2	1	1	1	1	2	1	1	1	0	2	1	1	0	1	1	2	1	2	2	2	1	2	1	2	2	1
	Total	1	2	1	2	2	3	4	2	2	4	1	5	2	2	2	3	3	2	1	3	3	2	4	1	5	4	5	2	4	1	3	4	1
Disclaimer of	Existence	1	0	0	0	0	0	0	0	0	0	0	0	0	0	0	0	0	0	0	0	0	0	0	0	0	0	0	0	0	0	0	0	0
boundaries	Specificity	1	1	1	1	1	1	1	1	1	1	1	1	1	1	1	1	1	1	1	1	1	1	1	1	1	1	1	1	1	1	1	1	1
	Operability	1	0	1	1	1	1	1	1	1	1	1	1	1	1	1	1	1	1	1	1	0	1	1	1	1	1	1	1	1	0	1	1	1
	Total	3	1	2	2	2	2	2	2	2	2	2	2	2	2	2	2	2	2	2	2	1	2	2	2	2	2	2	2	2	1	2	2	2
Language	Existence	1	0	0	1	0	0	1	1	0	1	0	0	0	0	1	0	1	0	1	0	0	0	1	0	0	0	0	1	0	0	0	0	1
clarity	Specificity	0	0	0	0	0	0	0	0	0	0	0	0	0	0	0	0	0	0	0	1	0	0	0	0	0	0	0	0	1	0	0	0	0
	Operability	1	1	1	1	1	1	2	1	1	1	1	1	0	1	1	1	2	1	2	1	1	1	1	1	1	1	1	2	1	1	1	1	1
	Total	2	1	1	2	1	1	3	2	1	2	1	1	0	1	2	1	3	1	3	2	1	1	2	1	1	1	1	3	2	1	1	1	2
Voluntariness	Existence	0	0	1	0	0	0	1	0	0	1	1	1	1	1	0	1	0	1	0	0	1	1	1	1	1	1	1	1	0	1	1	0	0
	Specificity	0	0	1	0	0	0	0	0	0	1	0	0	0	0	0	0	0	0	0	0	1	0	0	0	0	0	0	0	1	0	0	0	0

	Operability	1	1	2	1	1	1	2	0	1	1	2	2	1	0	1	1	1	1	1	1	0	2	1	2	2	1	1	2	1	1	2	1	1
	Total	1	1	4	1	1	1	3	0	1	3	3	3	2	1	1	2	1	2	1	1	2	3	2	3	3	2	2	3	2	2	3	1	1
Client obligations	Existence	0	1	1	1	0	1	1	1	1	1	0	1	1	1	1	1	1	1	1	0	1	0	0	0	0	1	1	0	1	1	1	1	1
	Specificity	1	0	1	1	0	0	0	1	0	0	0	2	0	1	1	0	1	1	2	0	0	0	0	0	0	0	1	0	1	0	1	0	1
	Operability	1	1	2	1	1	1	2	1	1	2	1	2	1	1	1	1	2	0	2	1	0	1	1	1	1	1	1	1	2	1	2	2	1
	Total	2	2	4	3	1	2	3	3	2	3	1	5	2	3	3	2	4	2	5	1	1	1	1	1	1	2	3	1	4	2	4	3	3
Counseling limitations	Existence	0	1	0	0	0	1	1	0	1	0	0	1	1	1	1	1	0	1	0	1	0	0	1	1	0	0	0	0	0	0	0	0	0
	Specificity	0	0	0	0	0	0	2	0	1	1	1	0	0	0	0	0	0	0	0	0	0	0	0	1	1	0	0	0	0	0	0	0	0
	Operability	1	1	1	1	1	1	2	1	1	1	0	2	0	1	1	1	1	0	1	1	0	1	2	2	1	1	1	1	1	1	1	1	1
	Total	1	2	1	1	1	2	5	1	3	2	1	3	1	2	2	2	1	1	1	2	0	1	3	4	2	1	1	1	1	1	1	1	1
Counselor qualifications	Existence	1	1	1	1	1	1	1	0	1	1	1	1	1	1	1	1	1	1	1	1	0	1	1	1	1	1	0	1	1	1	1	1	1
	Specificity	1	0	0	0	0	0	1	0	0	0	1	1	0	0	1	0	0	0	0	0	0	0	0	0	0	0	0	1	0	1	0	0	0
	Operability	2	1	2	1	1	0	2	1	1	1	1	2	0	1	0	0	2	1	2	1	0	2	2	2	2	2	1	2	2	1	2	1	1
	Total	4	2	3	2	2	1	4	1	2	2	3	4	1	2	2	1	3	2	3	2	0	3	3	3	3	3	1	4	3	3	3	2	2
Counseling modalities	Existence	0	1	1	0	1	0	0	0	0	1	0	0	0	0	0	1	0	0	0	0	1	0	0	0	1	0	0	0	0	0	0	0	0
	Specificity	1	2	1	1	1	1	1	1	1	2	1	1	1	1	1	2	1	1	1	1	2	1	1	1	1	1	1	1	1	1	1	1	1
	Operability	1	1	2	1	1	1	1	1	1	1	1	1	0	1	1	1	1	1	1	1	1	1	1	1	2	1	1	1	1	1	1	1	1
	Total	2	4	4	2	3	2	2	2	2	4	2	2	1	2	2	4	2	2	2	2	4	2	2	2	4	2	2	2	2	2	2	2	2
Target population	Existence	1	0	1	1	1	1	1	1	1	1	1	1	1	1	1	1	1	1	1	1	0	0	1	1	1	0	1	1	1	1	1	1	1
	Specificity	2	1	0	1	0	0	0	0	2	1	1	1	1	0	1	1	0	0	0	2	0	0	1	1	1	0	0	0	0	0	0	0	1
	Operability	1	1	2	0	1	2	2	1	1	0	1	2	1	1	1	1	1	1	2	1	1	1	2	2	2	1	1	2	2	1	2	1	1
	Total	4	2	3	2	2	3	3	2	4	2	3	4	3	2	3	3	2	2	3	4	1	1	4	4	4	1	2	3	3	2	3	2	3
Total score	Existence	13	11	16	11	12	13	16	11	14	15	10	15	14	14	17	15	15	14	14	14	13	12	14	14	15	11	13	13	12	10	16	11	14
	Specificity	21	17	20	15	16	19	22	14	20	25	19	22	19	18	22	19	16	15	16	18	16	19	16	22	22	16	19	18	18	10	16	15	20
	Operability	21	15	33	18	18	19	34	17	18	19	18	34	10	18	17	18	31	19	33	18	13	29	28	33	35	22	21	33	30	19	34	22	18
	Total	55	43	69	44	46	51	72	42	52	59	47	71	43	50	56	52	62	48	63	50	42	60	58	69	72	49	53	64	60	39	66	48	52

Supplementary Data S2. Table of detailed ratings by expert 02 for all documents

Version	Indicator	Dimension	Document ID																																
			01	02	03	04	05	06	07	08	09	10	11	12	13	14	15	16	17	18	19	20	21	22	23	24	25	26	27	28	29	30	31	32	33
Original	Confidentiality	Existence	1	1	1	1	1	1	1	0	1	1	1	1	1	1	1	1	1	1	1	1	1	1	1	1	1	1	0	1	1	1	1	1	1
		Specificity	1	0	1	1	2	1	0	0	2	0	1	0	0	1	0	1	0	0	0	0	0	2	0	0	1	0	1	1	0	0	1	1	1
		Operability	1	1	2	1	1	1	2	1	2	0	1	2	1	1	1	2	2	2	2	1	1	2	1	2	1	1	1	2	2	1	1	1	0
		Total	3	2	4	3	4	3	3	1	5	1	3	3	2	3	2	4	3	3	3	2	2	5	2	3	3	2	2	4	3	2	3	3	2
	Exceptions to confidentiality	Existence	1	0	1	1	1	1	0	1	1	0	1	0	1	1	1	0	1	1	1	0	1	1	1	1	1	0	1	0	0	0	0	1	1
		Specificity	1	1	2	0	2	1	0	1	1	0	0	1	0	0	2	0	1	1	1	0	1	0	0	1	1	0	0	1	1	1	1	1	0
		Operability	0	1	2	1	1	1	1	1	1	1	1	1	1	1	2	1	2	1	2	0	1	2	1	2	1	1	1	1	1	0	1	1	1
		Total	2	2	5	2	4	3	1	3	3	1	2	2	2	2	5	1	4	3	4	0	3	3	2	4	3	1	2	2	2	1	2	3	2
	Client rights	Existence	1	1	0	1	1	0	1	1	1	1	1	1	1	0	1	1	1	1	1	1	1	1	1	1	1	1	1	1	1	1	1	1	1
		Specificity	1	1	1	1	0	1	1	1	2	0	0	0	1	1	2	1	1	1	1	0	1	1	1	1	2	0	1	0	1	1	1	2	1
		Operability	1	1	1	1	2	1	2	1	2	0	1	2	1	1	2	1	2	1	2	1	2	2	2	2	1	2	1	1	2	1	2	1	1
		Total	3	3	2	3	3	2	4	3	5	1	2	3	3	2	5	3	4	3	4	2	4	4	4	4	4	3	3	2	4	3	4	4	3
	Guardian consent	Existence	0	1	1	0	1	1	0	1	0	1	0	0	1	0	1	0	1	1	1	0	1	1	0	1	1	0	0	0	1	0	1	1	1
		Specificity	1	0	2	1	2	1	1	1	1	2	1	1	1	1	2	0	2	1	1	1	1	2	0	1	1	1	0	1	1	0	1	2	1
		Operability	1	1	2	1	1	0	1	1	1	1	1	1	0	1	0	1	2	2	2	1	1	2	1	2	1	1	1	1	2	0	2	1	1
		Total	2	2	5	2	4	2	2	3	2	4	2	2	2	2	3	1	5	4	4	2	3	5	1	4	3	2	1	2	4	0	4	4	3
	Goals and scope	Existence	0	0	1	0	1	0	1	1	0	1	0	1	1	0	1	0	1	1	1	0	1	1	1	1	0	0	1	0	1	0	1	1	0
		Specificity	1	1	1	1	2	1	0	2	1	0	0	1	0	1	2	0	1	1	1	0	1	0	2	1	0	0	0	1	1	0	2	0	1
		Operability	0	1	2	1	1	1	2	1	1	1	1	1	1	1	0	1	2	1	2	1	1	1	2	2	1	1	2	1	2	1	2	0	1
		Total	1	2	4	2	4	2	3	4	2	2	1	3	2	2	3	1	4	3	4	1	3	2	5	4	1	1	3	2	4	1	5	1	2
	Format and frequency	Existence	1	1	1	1	1	1	1	1	1	1	1	1	1	1	1	1	1	1	1	1	1	1	1	1	1	1	0	1	1	1	1	0	1
		Specificity	2	0	2	2	1	2	2	2	1	2	0	2	2	2	2	1	2	1	1	2	0	0	2	2	2	1	1	1	2	1	2	0	2
		Operability	2	1	2	2	1	1	2	1	1	1	1	2	1	1	1	1	2	0	2	2	2	2	2	2	1	2	1	2	2	1	1	1	1
		Total	5	2	5	5	3	4	5	4	3	4	2	5	4	4	4	3	5	2	4	5	3	3	5	5	4	4	2	4	5	3	4	1	4
	Fees and cancellation policy	Existence	1	1	1	1	1	1	1	1	1	1	0	1	1	0	1	1	0	1	1	0	0	1	1	1	1	1	1	1	1	0	0	1	0
		Specificity	1	1	1	1	1	1	0	1	0	1	1	0	2	1	1	1	1	1	1	0	0	1	1	0	1	0	0	1	1	1	1	1	1
		Operability	1	1	2	2	0	2	2	1	0	1	1	2	2	1	1	2	1	1	2	1	1	2	2	2	1	1	1	2	2	0	1	1	1
		Total	3	3	4	4	2	4	3	3	1	3	2	3	5	2	3	4	2	3	4	1	1	4	4	3	3	2	2	4	4	1	2	3	2
	Recording methods	Existence	1	1	1	1	1	1	1	1	1	1	0	1	1	1	0	0	1	1	1	0	0	1	1	1	1	1	0	1	1	0	1	1	1
		Specificity	2	0	1	1	2	0	1	2	1	2	0	1	2	2	1	1	1	1	2	1	1	2	1	2	0	0	1	1	2	1	1	1	1

	Operability	1	1	2	0	2	1	1	0	1	1	1	2	1	1	0	1	2	1	2	1	1	2	1	2	1	1	1	2	2	0	1	1	1
	Total	4	2	4	2	5	2	3	3	3	4	1	4	4	4	1	2	4	3	5	2	2	5	3	5	2	2	2	4	5	1	3	3	3
Authorization	Existence	1	0	1	1	1	0	1	1	1	0	0	1	0	0	0	1	1	1	1	1	0	0	1	1	1	1	1	0	1	0	1	1	0
and revocation	Specificity	1	0	2	0	1	0	0	0	2	0	1	0	0	0	1	1	0	1	0	0	0	1	0	1	1	0	0	0	0	0	0	1	0
	Operability	1	1	2	1	1	1	2	1	1	1	1	2	1	1	0	1	2	1	2	1	1	1	2	2	1	1	1	1	2	1	2	1	1
	Total	3	1	5	2	3	1	3	2	4	1	2	3	1	1	1	3	3	3	3	2	1	2	3	4	3	2	2	1	3	1	3	3	1
Crisis	Existence	1	0	1	1	0	1	1	1	1	1	1	1	1	1	1	1	1	1	1	1	0	1	1	1	1	1	1	1	1	1	0	1	1
procedures	Specificity	0	1	1	1	1	1	0	0	0	0	0	2	0	0	1	0	1	0	1	0	0	1	1	2	1	0	1	0	2	0	0	0	0
	Operability	1	1	2	1	1	1	2	1	1	1	1	2	1	1	1	1	2	1	2	1	0	2	2	2	1	1	1	2	2	1	1	1	1
	Total	2	2	4	3	2	3	3	2	2	2	2	5	2	2	3	2	4	2	4	2	0	4	4	5	3	2	3	3	5	2	1	2	2
Complaints	Existence	0	0	1	0	0	0	0	0	0	0	0	0	0	0	0	1	0	0	0	1	0	0	0	0	0	0	1	1	0	0	0	1	0
and appeals	Specificity	1	1	1	0	1	0	0	0	1	1	1	0	0	1	1	1	0	0	0	0	1	1	0	0	0	0	0	0	0	0	1	1	0
	Operability	1	1	2	1	1	1	1	1	1	0	1	1	1	1	1	1	1	1	1	1	1	1	1	1	1	1	1	2	1	0	1	1	1
	Total	2	2	4	1	2	1	1	1	2	1	2	1	1	2	2	3	1	1	1	2	2	2	1	1	1	1	2	3	1	0	2	3	1
Data	Existence	0	0	0	1	0	0	0	0	0	0	0	0	0	1	0	0	0	0	0	0	0	0	1	0	0	0	1	1	1	0	1	1	0
protection	Specificity	0	0	0	1	1	0	0	1	0	0	1	0	1	1	1	1	0	0	1	1	0	1	1	0	0	0	0	0	1	0	0	0	1
	Operability	1	1	1	1	1	1	1	1	1	1	1	1	1	2	1	1	1	1	1	1	1	1	2	1	0	1	0	2	2	1	2	0	0
	Total	1	1	1	3	2	1	1	2	1	1	2	1	2	4	2	2	1	1	2	2	1	2	4	1	0	1	1	3	4	1	3	1	1
Disclaimer of	Existence	1	0	0	0	0	0	0	0	1	0	1	0	0	0	0	0	0	0	0	0	0	0	0	0	0	0	0	0	0	0	0	1	0
boundaries	Specificity	2	1	1	0	1	1	1	1	2	0	0	1	1	1	1	1	1	1	1	1	0	1	1	1	0	1	1	1	1	0	1	0	1
	Operability	1	1	1	1	1	1	1	0	0	1	1	1	1	1	0	1	1	1	1	1	1	1	1	1	1	1	0	1	1	1	1	1	0
	Total	4	2	2	1	2	2	2	1	3	1	2	2	2	2	1	2	2	2	2	2	1	2	2	2	1	2	1	2	2	1	2	2	1
Language	Existence	0	0	0	0	1	0	0	1	0	0	1	0	0	1	1	0	0	0	1	1	0	0	0	0	0	0	0	0	0	0	0	0	0
clarity	Specificity	0	0	0	0	0	0	1	0	0	0	0	0	0	0	1	0	0	0	0	0	0	0	0	0	0	1	0	0	1	0	0	0	0
	Operability	1	1	1	0	2	1	1	1	1	1	0	1	1	1	1	1	1	1	1	1	1	1	1	1	1	1	1	1	1	1	1	1	1
	Total	1	1	1	0	3	1	2	2	1	1	1	1	1	2	3	1	1	1	2	2	1	1	1	1	1	2	1	1	2	1	1	1	1
Voluntariness	Existence	0	0	0	1	0	1	0	0	1	0	1	1	1	0	0	1	0	0	1	0	1	1	1	1	1	1	1	1	0	1	1	0	0
	Specificity	1	1	0	0	0	1	0	0	0	0	1	0	0	0	0	0	0	0	0	0	0	0	0	1	0	0	0	0	0	0	1	0	0
	Operability	0	1	1	1	1	1	1	1	1	1	1	2	1	1	1	1	1	1	2	1	1	2	2	2	1	1	1	2	1	1	2	1	1
	Total	1	2	1	2	1	3	1	1	2	1	3	3	2	1	1	2	1	1	3	1	2	3	3	4	2	2	2	3	1	2	4	1	1
Client	Existence	0	1	0	0	1	0	0	1	1	0	0	1	1	0	1	0	1	1	1	0	0	0	0	1	0	0	0	0	1	0	1	1	1
obligations	Specificity	0	1	0	0	1	0	0	1	0	0	0	0	1	0	0	0	0	0	0	0	0	0	0	0	0	0	0	0	0	0	0	0	0
	Operability	1	1	1	1	2	1	1	1	1	1	1	2	1	1	1	1	2	2	2	1	1	1	1	2	1	1	1	1	2	1	2	1	1

		Total	1	3	1	1	4	1	1	3	2	1	1	3	3	1	2	1	3	3	3	1	1	1	1	3	1	1	1	1	3	1	3	2	2
	Counseling limitations	Existence	0	0	0	0	0	0	0	1	0	0	0	0	0	0	0	0	0	0	0	0	1	0	0	0	0	0	0	0	1	0	0	0	0
		Specificity	0	0	0	0	0	0	0	0	0	0	0	0	1	0	0	0	0	0	1	0	0	0	0	0	0	0	0	0	0	1	0	0	0
		Operability	1	1	1	1	1	1	1	0	1	1	1	1	1	1	1	1	1	1	1	1	2	1	1	1	1	1	1	1	1	1	1	0	1
		Total	1	1	1	1	1	1	1	1	1	1	1	1	2	1	1	1	1	1	2	1	3	1	1	1	1	1	1	1	2	2	1	0	1
	Counselor qualifications	Existence	1	1	1	1	1	0	0	1	1	1	0	1	1	1	0	1	1	1	0	1	0	1	1	1	1	1	1	1	1	0	1	1	1
		Specificity	0	0	0	1	0	0	0	0	0	1	0	0	0	0	0	0	1	0	0	0	0	0	0	0	0	0	0	0	0	0	0	0	0
		Operability	2	1	2	1	1	1	1	2	1	1	1	2	1	0	1	1	1	1	1	1	1	2	1	1	1	1	1	2	2	1	2	1	1
		Total	3	2	3	3	2	1	1	3	2	3	1	3	2	1	1	2	3	2	1	2	1	3	2	2	2	2	2	3	3	1	3	2	2
	Counseling modalities	Existence	0	1	1	0	1	0	1	0	0	0	0	0	1	0	0	0	0	0	0	0	0	0	0	0	1	0	1	0	0	1	0	0	1
		Specificity	0	0	1	1	0	1	0	0	0	1	0	0	1	0	0	1	0	0	0	0	1	0	0	0	0	1	0	0	1	0	0	0	0
		Operability	1	1	2	1	1	1	2	1	1	1	1	1	2	1	1	0	1	1	1	1	1	1	1	1	1	0	2	1	1	1	1	1	1
		Total	1	2	4	2	2	2	3	1	1	2	1	1	4	1	1	1	1	1	1	1	2	1	1	1	2	1	3	1	2	2	1	1	2
	Target population	Existence	1	0	0	1	0	0	1	1	1	1	1	1	1	1	1	1	1	1	1	0	0	1	1	1	1	0	1	1	1	1	1	1	1
		Specificity	2	1	0	0	0	0	0	0	2	1	1	2	1	0	2	1	1	1	2	0	0	0	0	1	2	0	0	0	2	0	1	0	0
		Operability	1	1	1	0	1	1	2	2	1	1	1	2	1	2	1	1	1	1	2	1	1	2	2	2	2	1	2	2	2	1	2	1	1
		Total	4	2	1	1	1	1	3	3	4	3	3	5	3	3	4	3	3	3	5	1	1	3	3	4	5	1	3	3	5	2	4	2	2
	Total score	Existence	11	9	12	12	13	8	10	14	13	10	9	12	14	9	11	10	12	13	14	8	8	12	13	14	13	9	12	11	14	7	12	15	11
		Specificity	17	10	17	12	18	12	7	13	16	11	8	11	14	12	20	11	13	10	14	6	7	13	10	14	12	5	6	8	17	6	14	10	10
		Operability	19	20	32	19	23	20	29	19	20	17	19	31	21	21	17	21	30	22	33	20	22	31	29	33	20	21	21	30	33	15	29	17	17
		Total	47	39	61	43	54	40	46	46	49	38	36	54	49	42	48	42	55	45	61	34	37	56	52	61	45	35	39	49	64	28	55	42	38
ChatGPT-5	Confidentiality	Existence	1	0	0	1	1	1	1	1	1	1	1	1	1	1	1	1	1	0	1	1	1	1	0	1	0	1	1	1	1	1	1	1	0
		Specificity	2	1	1	1	0	2	0	2	2	1	2	1	2	2	1	1	2	1	1	2	2	2	1	1	1	2	1	2	0	1	2	2	1
		Operability	1	1	1	1	0	1	2	1	1	1	1	2	1	0	1	1	2	1	2	1	1	2	1	2	1	1	0	2	2	1	2	1	0
		Total	4	2	2	3	1	4	3	4	4	3	4	4	4	3	3	3	5	2	4	4	4	5	2	4	2	4	2	5	3	3	5	4	1
	Exceptions to confidentiality	Existence	1	1	0	1	1	1	1	1	1	1	1	0	1	0	1	1	1	0	1	1	1	1	1	0	1	1	1	1	1	0	1	1	1
		Specificity	1	2	1	0	2	2	2	1	2	0	2	1	2	1	1	1	2	1	2	1	2	2	2	1	0	1	0	1	2	1	2	2	2
		Operability	2	1	1	1	1	1	2	1	1	1	1	1	2	0	1	1	2	1	2	0	1	2	1	1	1	0	1	1	2	1	2	1	1
		Total	4	4	2	2	4	4	5	3	4	2	4	2	5	1	3	3	5	2	5	2	4	5	4	2	2	2	2	3	5	2	5	4	4
	Client rights	Existence	1	1	1	1	1	1	1	1	1	1	1	1	1	1	0	1	0	0	1	1	1	1	1	1	1	1	1	1	1	1	1	1	1
		Specificity	2	1	2	2	0	1	1	1	1	0	2	1	1	1	1	1	1	1	2	1	1	2	1	2	2	2	2	0	0	2	2	2	2
		Operability	1	1	2	1	0	1	2	2	1	1	1	2	1	0	1	1	1	1	2	1	1	2	1	1	1	1	1	2	2	1	2	1	1
		Total	4	3	5	4	1	3	4	4	3	2	4	4	3	2	2	3	2	2	5	3	3	5	3	4	4	4	4	3	3	4	5	4	4

Guardian	Existence	1	1	1	0	1	1	0	1	1	0	1	0	1	1	1	0	1	1	1	1	1	1	1	1	1	1	0	0	1	0	1	1	0
consent	Specificity	1	2	2	1	1	2	1	2	2	1	2	1	2	2	1	1	2	2	2	1	2	2	1	2	1	1	1	1	2	1	2	2	1
	Operability	1	1	1	1	1	0	1	1	1	0	1	1	1	1	1	0	2	2	2	1	0	2	2	2	1	0	0	1	2	1	2	1	1
	Total	3	4	4	2	3	3	2	4	4	1	4	2	4	4	3	1	5	5	5	3	3	5	4	5	3	2	1	2	5	2	5	4	2
Goals and	Existence	1	1	1	0	1	1	1	1	1	0	1	0	1	1	1	1	1	1	1	1	1	1	0	1	0	0	1	0	1	1	1	1	1
scope	Specificity	1	2	2	0	2	2	0	1	1	0	2	1	1	2	0	2	1	1	0	0	1	1	1	1	1	1	2	1	1	0	2	1	0
	Operability	2	1	2	1	2	2	2	1	1	1	1	1	1	1	1	1	2	1	2	1	1	2	1	2	0	1	1	1	2	1	2	0	1
	Total	4	4	5	1	5	5	3	3	3	1	4	2	3	4	2	4	4	3	3	2	3	4	2	4	1	2	4	2	4	2	5	2	2
Format and	Existence	1	1	1	1	1	1	1	1	1	1	1	1	1	1	1	1	1	1	1	1	0	1	1	1	1	0	0	1	1	0	1	1	1
frequency	Specificity	1	0	2	1	2	2	1	0	2	1	1	2	2	1	2	1	1	2	1	0	0	0	1	2	2	0	1	1	1	1	2	0	1
	Operability	1	0	2	1	1	1	2	1	1	0	1	2	1	1	1	1	2	2	2	1	1	2	2	2	1	1	1	2	2	1	2	2	1
	Total	3	1	5	3	4	4	4	2	4	2	3	5	4	3	4	3	4	5	4	2	1	3	4	5	4	1	2	4	4	2	5	3	3
Fees and	Existence	1	1	1	1	0	1	1	1	1	1	1	1	1	1	0	1	1	1	1	1	0	0	1	1	1	1	1	1	1	1	1	1	1
cancellation	Specificity	2	1	2	1	0	2	1	1	1	1	1	1	1	1	1	1	1	1	1	1	1	1	1	1	0	0	1	0	0	1	1	1	1
policy	Operability	2	1	2	1	0	1	1	1	1	1	1	2	0	1	1	0	2	2	1	1	1	1	2	2	1	0	1	2	1	2	2	1	1
	Total	5	3	5	3	0	4	3	3	3	3	3	4	2	3	2	2	4	4	3	3	2	2	4	4	2	1	3	3	2	4	4	3	3
Recording	Existence	0	1	1	1	1	1	1	1	0	1	1	1	1	0	1	1	1	1	1	0	1	0	1	1	0	1	0	1	1	0	1	1	1
methods	Specificity	1	1	2	0	2	1	1	2	1	1	2	0	0	1	2	2	1	1	2	0	1	1	2	0	1	1	1	1	2	0	1	0	0
	Operability	0	2	2	1	1	1	2	1	1	0	1	1	1	0	1	0	1	2	2	1	1	1	2	2	1	1	0	1	2	1	2	1	1
	Total	1	4	5	2	4	3	4	4	2	2	4	2	2	1	4	3	3	4	5	1	3	2	5	3	2	3	1	3	5	1	4	2	2
Authorization	Existence	1	0	1	1	1	0	1	1	0	1	1	1	1	1	1	1	1	1	1	1	1	1	0	1	1	0	0	1	1	0	0	0	1
and revocation	Specificity	2	1	1	1	1	1	0	1	0	2	1	0	1	1	0	1	1	1	1	0	1	1	1	2	0	0	0	0	1	0	1	1	1
	Operability	1	1	2	1	1	1	2	1	1	1	1	2	1	1	1	1	2	2	2	1	1	2	1	2	1	0	1	2	2	1	1	1	1
	Total	4	2	4	3	3	2	3	3	1	4	3	3	3	3	2	3	4	4	4	2	3	4	2	5	2	0	1	3	4	1	2	2	3
Crisis	Existence	1	1	1	1	1	1	1	1	1	1	1	1	1	1	1	1	1	0	1	1	1	1	1	0	1	1	1	0	1	1	1	1	1
procedures	Specificity	2	1	1	0	1	1	0	0	0	0	1	1	2	0	1	1	1	1	0	1	0	0	0	1	1	1	1	0	1	2	1	0	1
	Operability	1	1	2	0	1	1	2	1	1	1	1	2	1	1	0	1	2	1	2	1	0	1	1	1	1	1	1	1	2	1	2	1	1
	Total	4	3	4	1	3	3	3	2	2	2	3	4	4	2	2	3	4	2	3	3	1	2	2	2	3	3	3	1	4	4	4	2	3
Complaints	Existence	0	1	1	1	0	1	0	0	1	0	1	0	0	0	0	0	0	0	0	0	0	0	0	1	0	1	0	0	0	0	0	0	0
and appeals	Specificity	1	1	2	1	0	1	0	1	1	1	1	0	0	1	1	1	1	1	1	1	1	1	1	1	0	1	1	1	1	0	1	1	0
	Operability	1	1	2	1	1	1	1	1	0	1	1	1	1	1	0	1	1	1	1	0	1	1	1	2	1	0	1	1	1	0	1	1	1
	Total	2	3	5	3	1	3	1	2	2	2	3	1	1	2	1	2	2	2	2	1	2	2	2	4	1	2	2	2	2	0	2	2	1
Data	Existence	1	0	1	1	0	0	0	0	0	0	0	0	1	0	0	0	0	0	1	1	1	0	1	0	0	1	0	0	0	0	1	0	1

protection	Specificity	0	1	2	1	1	0	0	1	1	0	1	0	0	1	1	1	0	0	1	0	1	0	1	0	0	0	0	0	1	0	0	0	0
	Operability	1	0	2	1	0	1	1	1	1	0	1	1	1	1	1	1	1	1	1	1	1	1	2	1	1	1	1	1	1	1	2	1	1
	Total	2	1	5	3	1	1	1	2	2	0	2	1	2	2	2	2	1	1	3	2	3	1	4	1	1	2	1	1	2	1	3	1	2
Disclaimer of	Existence	0	0	0	0	0	0	0	0	0	0	0	1	0	0	1	0	0	0	0	0	0	0	0	0	0	0	0	0	1	0	0	0	0
boundaries	Specificity	1	1	1	0	1	1	1	1	1	1	1	2	1	1	2	1	1	1	1	1	1	1	1	1	1	1	1	1	1	1	1	1	1
	Operability	1	1	1	1	1	1	1	1	1	1	0	2	1	1	2	1	1	1	1	1	1	1	1	1	1	1	1	1	2	1	1	1	0
	Total	2	2	2	1	2	2	2	2	2	2	1	5	2	2	5	2	2	2	2	2	2	2	2	2	2	2	2	2	4	2	2	2	1
Language	Existence	0	0	0	0	0	0	0	0	0	0	0	0	0	0	0	0	0	0	1	1	0	0	0	0	0	0	0	1	0	0	0	0	0
clarity	Specificity	0	0	0	0	1	0	0	0	0	0	0	0	0	0	0	1	0	0	0	0	1	1	0	0	0	0	1	0	1	0	0	0	0
	Operability	1	1	1	1	1	1	1	1	1	1	1	1	0	1	1	1	1	1	2	1	1	1	1	1	1	1	0	2	1	1	1	0	1
	Total	1	1	1	1	2	1	1	1	1	1	1	1	0	1	1	2	1	1	3	2	2	2	1	1	1	1	1	3	2	1	1	0	1
Voluntariness	Existence	1	1	1	1	1	1	0	0	0	1	1	1	1	0	0	0	1	0	1	1	1	0	0	1	1	1	0	1	1	1	1	1	1
	Specificity	1	1	0	0	0	0	0	0	0	0	0	1	0	0	0	0	0	0	0	0	0	1	0	0	0	0	1	0	0	0	0	0	0
	Operability	1	1	2	1	0	0	1	0	0	1	1	2	2	1	1	0	2	1	2	1	0	1	1	1	1	1	1	2	1	1	2	1	2
	Total	3	3	3	2	1	1	1	0	0	2	2	4	3	1	1	0	3	1	3	2	1	2	1	2	2	2	2	3	2	2	3	2	3
Client	Existence	0	0	1	0	0	0	0	1	1	1	1	0	1	1	0	0	1	1	1	1	1	0	0	1	1	0	1	1	1	1	1	1	1
obligations	Specificity	1	0	1	0	0	0	0	1	1	0	1	1	2	2	0	0	0	0	0	0	0	0	0	0	1	0	1	0	1	0	0	0	1
	Operability	1	1	2	1	1	1	1	1	1	2	1	0	1	1	1	1	2	2	2	1	1	1	1	2	0	1	0	2	1	1	2	1	1
	Total	2	1	4	1	1	1	1	3	3	3	3	1	4	4	1	1	3	3	3	2	2	1	1	3	2	1	2	3	3	2	3	2	3
Counseling	Existence	0	1	0	0	0	0	0	0	0	1	0	0	0	0	0	0	1	1	0	0	0	0	0	0	0	0	0	0	0	0	0	1	1
limitations	Specificity	0	0	0	0	0	0	0	1	0	0	1	0	0	0	0	1	0	1	0	0	0	1	0	0	1	0	0	0	0	0	0	0	0
	Operability	0	1	1	1	1	1	1	1	1	2	1	1	1	1	1	1	2	1	1	1	1	1	1	1	1	1	1	1	1	1	1	1	1
	Total	0	2	1	1	1	1	1	2	1	3	2	1	1	1	1	2	3	3	1	1	1	2	1	1	2	1	1	1	1	1	1	2	2
Counselor	Existence	0	1	1	1	1	1	0	0	0	1	1	1	1	1	1	1	0	1	1	1	1	1	1	1	1	1	1	1	1	1	1	1	1
qualifications	Specificity	1	0	0	0	0	0	0	1	0	0	0	0	0	0	0	0	0	0	0	0	0	1	1	0	0	0	0	0	0	1	1	0	0
	Operability	1	1	2	1	2	1	1	1	1	1	2	2	1	1	0	1	1	1	2	0	0	2	2	2	1	0	1	1	2	0	2	1	1
	Total	2	2	3	2	3	2	1	2	1	2	3	3	2	2	1	2	1	2	3	1	1	4	4	3	2	1	2	2	3	2	4	2	2
Counseling	Existence	0	1	1	0	1	0	0	0	0	0	1	0	1	0	0	0	0	0	0	0	0	0	0	0	1	0	1	0	0	0	0	0	0
modalities	Specificity	1	2	2	1	2	1	1	1	1	0	2	1	2	1	0	1	0	0	1	0	1	0	0	0	2	1	2	0	1	1	0	1	0
	Operability	1	1	2	1	0	1	1	1	1	1	1	1	1	1	1	1	1	1	1	1	0	1	1	1	1	1	1	1	1	1	1	1	1
	Total	2	4	5	2	3	2	2	2	2	1	4	2	4	2	1	2	1	1	2	1	1	1	1	1	4	2	4	1	2	2	1	2	1
Target	Existence	1	0	1	1	0	0	1	1	1	1	1	1	1	1	1	1	1	1	1	0	0	1	1	1	1	0	1	0	1	1	1	1	0
population	Specificity	2	0	0	0	0	0	0	1	2	1	1	1	1	0	2	0	1	1	1	0	0	2	2	1	1	0	0	0	1	1	2	2	0

		Operability	1	1	2	1	1	0	2	1	1	2	1	2	1	1	1	1	2	2	2	1	1	2	2	2	1	1	0	1	1	1	2	1	1
		Total	4	1	3	2	1	0	3	3	4	4	3	4	3	2	4	2	4	4	4	1	1	5	5	4	3	1	1	1	3	3	5	4	1
	Total score	Existence	12	13	15	13	12	12	10	12	11	13	16	11	16	11	11	11	13	10	16	14	12	10	10	13	12	11	10	11	15	9	14	14	13
		Specificity	23	18	24	10	16	19	9	19	19	10	24	15	20	18	16	18	16	16	17	9	16	20	17	16	15	12	17	9	17	13	21	16	12
		Operability	21	19	34	19	16	18	29	20	18	19	20	29	20	16	18	16	32	27	34	17	15	29	27	31	18	14	14	28	31	19	34	19	19
		Total	56	50	73	42	44	49	48	51	48	42	60	55	56	45	45	45	61	53	67	40	43	59	54	60	45	37	41	48	63	41	69	49	44
Grok-4	Confidentiality	Existence	1	1	1	1	1	1	1	1	1	1	1	1	1	1	1	0	1	1	1	1	0	1	1	1	1	1	1	1	1	1	1	1	1
		Specificity	2	1	2	2	2	2	1	1	2	2	2	1	1	1	2	1	2	1	1	1	1	2	1	2	2	2	1	1	1	0	1	2	2
		Operability	0	1	2	1	1	1	2	1	1	1	1	1	1	2	1	1	2	1	2	1	1	2	2	2	2	1	1	2	2	1	2	1	1
		Total	3	3	5	4	4	4	4	3	4	4	4	3	3	4	4	2	5	3	4	3	2	5	4	5	5	4	3	4	4	2	4	4	4
	Exceptions to confidentiality	Existence	0	1	1	1	1	1	1	1	1	1	1	1	1	1	1	1	0	1	1	1	1	1	1	1	1	1	1	0	1	1	1	1	1
		Specificity	1	2	2	1	1	1	2	1	2	1	2	1	1	2	2	1	1	0	2	1	2	1	2	2	2	1	2	1	2	2	2	1	2
		Operability	1	1	2	1	0	1	2	1	1	0	1	2	1	1	1	0	1	2	2	1	1	2	2	2	2	1	1	1	2	1	1	1	1
		Total	2	4	5	3	2	3	5	3	4	2	4	4	3	4	4	2	2	3	5	3	4	4	5	5	5	3	4	2	5	4	4	3	4
	Client rights	Existence	1	1	1	1	1	1	1	1	1	1	1	1	1	1	1	1	1	1	1	1	1	1	1	1	1	1	1	1	1	1	1	1	0
		Specificity	2	2	2	2	1	2	2	2	2	2	2	1	2	2	2	1	2	2	2	1	2	1	2	1	2	2	2	2	2	2	2	2	1
		Operability	0	2	2	1	1	1	2	2	2	1	2	2	1	1	1	1	2	1	2	1	1	2	2	2	1	1	2	2	2	1	2	1	1
		Total	3	5	5	4	3	4	5	5	5	4	5	4	4	4	4	3	5	4	5	3	4	4	5	4	4	4	5	5	5	4	5	4	2
	Guardian consent	Existence	1	1	1	0	0	1	0	1	0	0	1	0	1	0	1	0	1	1	1	0	1	0	0	1	0	0	0	0	1	0	1	1	1
		Specificity	2	2	2	1	1	2	1	1	1	1	2	1	2	1	2	1	2	1	1	1	1	1	1	1	1	1	1	1	0	1	1	2	2
		Operability	1	1	2	1	1	1	1	0	1	1	1	1	1	1	1	1	2	1	1	0	1	1	1	2	1	1	1	1	2	1	2	0	1
		Total	4	4	5	2	2	4	2	2	2	2	4	2	4	2	4	2	5	3	3	1	3	2	2	4	2	2	2	2	3	2	4	3	4
	Goals and scope	Existence	1	1	1	1	1	1	1	0	1	0	1	0	1	1	0	1	1	1	1	1	1	1	1	1	1	1	1	1	1	1	1	1	1
		Specificity	2	1	2	2	2	2	2	1	2	1	1	1	1	2	1	2	2	2	1	1	2	1	2	2	0	1	1	2	2	2	2	0	2
		Operability	1	1	1	1	1	1	2	1	0	1	1	1	1	1	1	1	2	0	2	1	2	2	2	1	2	1	1	2	2	1	1	1	1
		Total	4	3	4	4	4	4	5	2	3	2	3	2	3	4	2	4	5	3	4	3	5	4	5	4	3	3	3	5	5	4	4	2	4
	Format and frequency	Existence	1	1	1	1	1	1	1	1	1	1	1	1	1	1	0	1	1	1	1	1	1	0	1	1	1	1	1	1	1	1	1	0	1
		Specificity	2	2	2	2	2	2	2	2	2	2	2	2	2	1	1	2	1	2	2	1	0	1	2	2	2	1	1	2	2	2	2	0	2
		Operability	1	1	2	1	2	1	2	1	1	1	1	2	1	2	1	1	2	2	2	1	1	1	2	2	2	1	0	2	2	1	2	1	0
		Total	4	4	5	4	5	4	5	4	4	4	4	5	4	4	2	4	4	5	5	3	2	2	5	5	5	3	2	5	5	4	5	1	3
	Fees and cancellation	Existence	0	1	0	1	1	1	1	1	1	1	1	1	1	1	1	1	1	1	1	1	1	1	1	1	1	1	1	1	1	1	1	1	1
		Specificity	1	1	1	1	1	2	1	1	1	1	1	1	2	0	1	1	1	1	1	1	0	1	1	1	1	2	1	2	1	1	1	1	1

policy	Operability	1	1	1	1	1	1	2	1	1	0	1	2	1	0	1	1	2	1	2	2	1	2	2	2	2	1	1	2	2	1	2	1	2
	Total	2	3	2	3	3	4	4	3	3	2	3	4	4	1	3	3	4	3	4	4	2	4	4	4	4	4	3	5	4	3	4	3	4
Recording methods	Existence	1	1	0	1	1	1	1	1	1	1	0	0	1	1	1	1	1	1	1	1	1	1	1	1	1	1	0	1	1	0	1	1	1
	Specificity	2	2	1	1	2	1	2	2	1	2	0	1	2	2	1	2	2	2	1	2	2	1	2	1	2	2	1	2	1	0	1	2	1
	Operability	1	1	1	1	1	1	2	0	1	1	1	1	1	1	1	2	2	1	2	1	1	2	2	2	1	1	1	2	2	1	2	1	1
	Total	4	4	2	3	4	3	5	3	3	4	1	2	4	4	3	5	5	4	4	4	4	4	5	4	4	4	2	5	4	1	4	4	3
Authorization and revocation	Existence	0	0	0	1	1	1	1	1	1	1	1	1	1	1	1	1	1	1	1	0	1	1	1	1	1	1	1	1	0	1	1	1	1
	Specificity	1	1	0	0	2	1	1	0	1	1	1	1	1	0	1	1	1	0	1	1	0	1	0	1	1	2	0	0	1	0	0	1	2
	Operability	1	1	1	1	1	1	2	0	1	1	1	1	1	1	1	1	2	1	2	1	1	2	1	2	2	1	1	2	1	1	1	0	1
	Total	2	2	1	2	4	3	4	1	3	3	3	3	3	2	3	3	4	2	4	2	2	4	3	4	4	4	2	3	2	2	2	2	4
Crisis procedures	Existence	1	1	1	1	0	0	1	0	1	1	1	1	1	1	1	1	1	1	1	1	1	1	1	0	1	1	1	1	1	1	1	1	1
	Specificity	0	0	1	1	1	1	1	1	0	1	1	1	1	0	0	1	1	2	1	0	0	2	0	1	0	1	1	0	1	1	2	2	2
	Operability	1	1	2	1	1	1	2	0	1	1	1	2	0	1	1	1	2	1	2	1	1	2	2	1	2	1	1	2	2	1	2	1	0
	Total	2	2	4	3	2	2	4	1	2	3	3	4	2	2	2	3	4	4	4	2	2	5	3	2	3	3	3	3	4	3	5	4	3
Complaints and appeals	Existence	1	1	0	1	0	1	0	0	0	0	0	0	0	0	1	0	1	0	0	1	1	0	1	1	1	1	1	0	0	0	1	0	0
	Specificity	2	1	1	0	1	1	1	0	1	1	1	0	0	0	2	1	1	0	1	1	0	0	1	1	2	1	0	0	0	0	0	0	1
	Operability	1	1	1	1	0	0	1	1	1	0	1	1	1	1	1	1	2	1	1	1	1	1	2	2	2	1	1	1	1	1	2	1	1
	Total	4	3	2	2	1	2	2	1	2	1	2	1	1	1	4	2	4	1	2	3	2	1	4	4	5	3	2	1	1	1	3	1	2
Data protection	Existence	0	1	1	1	1	1	1	0	1	1	0	0	0	1	1	1	1	1	0	0	1	0	1	1	1	0	1	0	1	0	1	1	0
	Specificity	0	0	0	0	2	1	1	1	0	2	0	1	1	1	1	2	0	0	0	1	2	0	1	0	1	1	2	1	1	0	0	1	0
	Operability	1	1	2	1	1	1	2	1	1	1	1	1	1	0	2	1	2	1	1	1	1	1	2	2	1	1	2	1	1	0	1	1	1
	Total	1	2	3	2	4	3	4	2	2	4	1	2	2	2	4	4	3	2	1	2	4	1	4	3	3	2	5	2	3	0	2	3	1
Disclaimer of boundaries	Existence	0	0	0	1	0	0	0	1	0	0	0	0	0	1	1	0	0	0	0	0	1	0	0	0	0	1	0	0	0	0	0	0	0
	Specificity	1	1	1	2	1	1	1	2	1	1	1	1	1	2	1	1	1	1	1	1	2	1	1	1	1	2	1	1	1	1	1	1	1
	Operability	1	1	1	1	0	1	1	1	1	0	1	1	1	0	0	1	1	0	1	1	1	1	1	1	1	1	1	1	1	1	1	1	1
	Total	2	2	2	4	1	2	2	4	2	1	2	2	2	3	2	2	2	1	2	2	4	2	2	2	2	4	2	2	2	2	2	2	2
Language clarity	Existence	0	1	0	0	0	0	1	1	0	1	0	0	0	0	0	0	1	0	1	0	0	1	0	0	0	1	0	1	1	0	0	0	1
	Specificity	0	0	1	1	0	0	0	0	1	0	1	0	0	0	0	0	0	0	0	0	0	0	1	0	0	0	0	0	0	0	0	0	0
	Operability	1	1	1	1	1	1	2	1	1	1	1	1	1	1	1	1	2	1	2	1	1	2	1	1	1	1	1	1	1	1	1	0	1
	Total	1	2	2	2	1	1	3	2	2	2	2	1	1	1	1	1	3	1	3	1	1	3	2	1	1	2	1	2	2	1	1	0	2
Voluntariness	Existence	1	0	1	0	0	1	1	0	0	1	1	1	1	1	0	0	0	1	0	0	0	1	1	1	1	1	1	1	0	1	1	0	0
	Specificity	0	1	1	0	0	0	0	0	0	0	0	0	0	0	0	0	0	0	0	0	0	0	0	0	0	1	0	0	0	0	0	0	0

	Operability	1	1	2	0	1	1	2	1	1	2	2	2	1	1	1	1	1	1	1	1	1	2	2	2	2	1	0	2	1	1	2	1	1
	Total	2	2	4	0	1	2	3	1	1	3	3	3	2	2	1	1	1	2	1	1	1	3	3	3	3	3	1	3	1	2	3	1	1
Client obligations	Existence	0	1	0	1	1	1	1	0	0	0	0	1	1	1	1	1	1	1	1	0	0	0	1	1	1	1	1	0	1	1	1	1	1
	Specificity	0	1	1	1	1	0	0	1	0	0	0	2	1	1	1	0	1	1	1	0	0	0	0	0	1	0	0	0	1	0	1	0	2
	Operability	1	1	1	1	2	1	2	0	1	1	1	2	1	2	1	1	2	2	1	1	1	1	2	2	2	1	0	1	2	1	2	1	2
	Total	1	3	2	3	4	2	3	1	1	1	1	5	3	4	3	2	4	4	3	1	1	1	3	3	4	2	1	1	4	2	4	2	5
Counseling limitations	Existence	0	1	0	0	0	1	1	0	1	0	0	1	1	0	1	0	0	0	0	1	0	0	0	0	0	0	0	0	0	0	0	0	0
	Specificity	0	0	1	0	0	0	0	0	1	0	0	0	0	0	1	0	1	0	0	0	0	0	1	0	1	0	1	0	0	1	0	0	0
	Operability	0	1	1	1	0	1	2	1	1	1	1	2	1	1	1	1	1	1	1	1	0	1	1	1	1	1	1	1	1	1	1	1	1
	Total	0	2	2	1	0	2	3	1	3	1	1	3	2	1	3	1	2	1	1	2	0	1	2	1	2	1	2	1	1	2	1	1	1
Counselor qualifications	Existence	1	1	0	1	1	1	1	1	1	1	1	1	1	1	1	1	1	0	1	1	1	1	1	1	1	1	1	1	1	1	1	0	1
	Specificity	0	0	1	0	0	0	1	0	0	0	0	0	0	0	0	0	0	1	1	0	1	0	0	0	0	0	1	0	1	0	0	1	0
	Operability	2	1	1	1	1	2	2	1	1	1	2	2	1	1	1	1	1	1	2	2	1	2	2	2	2	2	2	2	2	1	1	1	1
	Total	3	2	2	2	2	3	4	2	2	2	3	3	2	2	2	2	2	2	4	3	3	3	3	3	3	3	4	3	4	2	2	2	2
Counseling modalities	Existence	1	1	1	1	1	0	0	0	0	1	1	0	0	0	1	0	0	0	0	0	1	0	0	0	1	0	1	0	1	0	0	0	0
	Specificity	1	1	2	2	1	1	1	1	1	2	1	1	1	1	2	1	1	1	1	1	2	1	1	1	0	1	2	1	2	1	1	1	1
	Operability	1	1	2	2	1	1	1	1	0	0	1	1	1	1	2	1	1	1	1	1	2	1	1	1	2	1	1	1	2	1	1	1	1
	Total	3	3	5	5	3	2	2	2	1	3	3	2	2	2	5	2	2	2	2	2	5	2	2	2	3	2	4	2	5	2	2	2	2
Target population	Existence	0	1	1	1	1	1	1	1	1	1	1	1	1	1	1	1	1	1	0	1	0	1	1	1	1	0	0	1	1	1	1	1	1
	Specificity	1	2	0	1	0	0	0	0	1	2	1	0	1	0	1	1	0	0	0	0	0	0	2	1	1	0	0	0	0	0	0	1	0
	Operability	1	1	2	1	1	1	2	2	1	2	1	2	1	1	1	2	2	0	1	1	1	2	2	2	2	1	1	1	0	1	2	1	1
	Total	2	4	3	3	2	2	3	3	3	5	3	3	3	2	3	4	3	1	1	2	1	3	5	4	4	1	1	2	1	2	3	3	2
Total score	Existence	11	17	11	16	13	16	16	12	13	14	13	12	15	15	16	12	15	14	13	12	14	12	15	15	16	15	14	12	15	12	16	12	13
	Specificity	20	21	24	20	21	20	20	17	20	22	19	16	20	16	22	19	20	17	18	14	17	14	21	18	20	21	18	16	19	14	17	18	22
	Operability	18	21	30	20	18	20	36	17	19	17	23	30	19	20	21	21	34	20	31	21	21	32	34	34	33	21	20	30	31	19	31	17	20
	Total	49	59	65	56	52	56	72	46	52	53	55	58	54	51	59	52	69	51	62	47	52	58	70	67	69	57	52	58	65	45	64	47	55

Supplementary Data S3. Table of detailed ratings by expert 03 for all documents

Version	Indicator	Dimension	Document ID																																
			01	02	03	04	05	06	07	08	09	10	11	12	13	14	15	16	17	18	19	20	21	22	23	24	25	26	27	28	29	30	31	32	33
Original	Confidentiality	Existence	1	1	1	1	1	1	1	1	1	1	1	1	1	1	1	1	1	1	1	1	1	1	1	1	1	1	1	1	1	1	1	1	1
		Specificity	1	0	2	1	2	1	0	0	2	1	1	0	0	1	0	1	1	0	0	1	0	2	2	1	2	2	1	1	0	0	1	1	0
		Operability	1	1	2	1	1	1	1	0	1	1	1	2	1	0	1	1	1	0	2	0	1	2	2	2	1	1	1	2	2	2	2	2	2
		Total	3	2	5	3	4	3	2	1	4	3	3	3	2	2	2	3	3	1	3	2	2	5	5	4	4	4	3	4	3	3	4	4	3
	Exceptions to confidentiality	Existence	1	0	1	1	1	1	0	1	1	0	0	1	1	1	1	1	1	1	1	0	1	1	1	1	1	0	1	1	1	0	1	1	1
		Specificity	2	1	1	1	2	1	0	1	1	0	0	1	0	1	1	0	1	0	1	0	1	1	1	1	1	1	0	1	1	0	2	1	1
		Operability	1	1	2	1	1	1	1	1	2	1	1	1	1	1	2	1	2	1	2	1	1	2	2	1	0	1	0	1	2	1	2	1	2
		Total	4	2	4	3	4	3	1	3	4	1	1	3	2	3	4	2	4	2	4	1	3	4	4	3	2	2	1	3	4	1	5	3	4
	Client rights	Existence	0	1	1	1	1	1	1	1	1	1	1	1	1	0	1	1	0	1	1	1	1	1	1	1	1	0	0	1	0	1	1	1	1
		Specificity	1	2	1	1	0	2	1	1	2	0	0	0	1	0	1	1	1	2	1	0	1	1	1	1	1	0	1	1	1	2	2	2	1
		Operability	1	1	1	1	0	1	2	1	2	1	1	1	1	1	1	1	1	1	2	1	1	2	2	2	1	1	1	2	1	1	2	1	2
		Total	2	4	3	3	1	4	4	3	5	2	2	2	3	1	3	3	2	4	4	2	3	4	4	4	3	1	2	4	2	4	5	4	4
	Guardian consent	Existence	1	1	1	0	1	1	0	1	1	0	0	1	1	0	0	0	1	1	1	0	1	0	1	1	1	0	0	0	1	0	1	1	1
		Specificity	2	0	1	1	2	1	0	1	2	1	1	0	1	1	1	1	1	1	0	1	0	1	1	0	1	1	1	1	1	0	1	2	1
		Operability	1	1	2	0	1	1	1	1	1	1	0	2	1	1	0	1	2	1	2	0	1	1	2	2	1	1	1	1	2	1	2	2	1
		Total	4	2	4	1	4	3	1	3	4	2	1	3	3	2	1	2	4	3	3	1	2	2	4	3	3	2	2	2	4	1	4	5	3
	Goals and scope	Existence	1	0	1	0	1	0	0	1	0	0	0	1	1	0	1	0	1	1	0	0	1	1	1	0	0	0	1	0	1	0	1	1	1
		Specificity	1	1	1	0	2	0	0	2	1	0	1	1	0	1	0	0	1	1	1	0	0	0	2	1	0	0	0	1	1	0	2	0	1
		Operability	0	1	2	1	1	0	1	1	0	1	1	2	2	1	0	1	2	1	1	1	1	2	2	1	1	0	1	1	2	1	2	1	1
		Total	2	2	4	1	4	0	1	4	1	1	2	4	3	2	1	1	4	3	2	1	2	3	5	2	1	0	2	2	4	1	5	2	3
	Format and frequency	Existence	1	1	1	1	1	1	1	1	1	0	1	1	1	1	1	1	1	1	1	1	0	1	0	0	1	1	1	1	1	0	1	1	1
		Specificity	2	0	2	2	1	2	1	2	1	1	0	2	2	2	2	2	2	2	2	2	0	0	1	1	1	2	1	1	2	1	2	0	2
		Operability	1	1	2	0	1	0	2	1	1	1	2	2	1	1	1	1	2	0	2	1	1	2	1	1	1	1	0	2	2	1	2	1	1
		Total	4	2	5	3	3	3	4	4	3	2	3	5	4	4	4	4	5	3	5	4	1	3	2	2	3	4	2	4	5	2	5	2	4
	Fees and cancellation policy	Existence	1	1	1	1	1	0	1	1	1	1	1	1	0	1	1	0	1	1	1	0	0	1	1	1	1	0	1	1	1	1	1	1	1
		Specificity	1	1	0	1	0	1	1	1	0	1	0	1	1	1	2	1	1	1	1	0	0	0	1	1	2	0	0	1	1	1	1	1	1
		Operability	1	1	2	1	1	1	2	1	1	1	1	2	1	1	0	1	2	1	1	1	1	1	1	2	1	1	1	1	2	1	2	2	1
		Total	3	3	3	3	2	2	4	3	2	3	2	4	2	3	3	2	4	3	3	1	1	2	3	4	4	1	2	3	4	3	4	4	3
	Recording methods	Existence	1	0	1	1	1	0	0	1	1	1	1	1	1	1	0	0	1	1	1	1	0	1	1	1	1	1	0	1	1	0	1	1	0
		Specificity	1	1	2	1	2	0	1	2	1	1	0	1	2	2	1	1	2	0	1	1	0	2	2	1	0	1	1	2	2	0	0	1	1

	Operability	1	1	1	1	1	1	1	1	0	2	0	2	0	1	1	1	2	1	1	0	1	2	2	2	1	0	1	2	2	1	2	0	1
	Total	3	2	4	3	4	1	2	4	2	4	1	4	3	4	2	2	5	2	3	2	1	5	5	4	2	2	2	5	5	1	3	2	2
Authorization	Existence	1	0	1	1	1	0	1	1	1	1	0	1	1	0	0	1	1	1	1	0	0	1	0	1	1	1	1	1	1	0	1	0	0
and revocation	Specificity	1	0	1	0	0	1	0	0	1	1	1	0	0	0	0	1	0	0	1	1	0	2	0	0	1	0	0	0	1	0	0	0	0
	Operability	1	1	2	1	1	1	2	1	1	1	1	1	1	1	1	1	2	1	2	1	0	2	1	2	1	1	1	2	2	1	2	1	1
	Total	3	1	4	2	2	2	3	2	3	3	2	2	2	1	1	3	3	2	4	2	0	5	1	3	3	2	2	3	4	1	3	1	1
Crisis	Existence	1	1	1	1	1	1	1	1	1	1	1	1	1	1	1	1	1	1	1	1	1	1	1	1	1	1	0	1	1	1	1	1	1
procedures	Specificity	0	0	1	1	0	1	1	1	0	0	0	1	0	1	1	0	1	1	0	0	0	0	1	1	1	0	0	0	2	1	0	0	0
	Operability	1	1	2	1	1	1	1	1	1	1	1	2	1	1	1	1	2	1	2	0	1	2	2	2	0	1	1	2	2	0	2	1	1
	Total	2	2	4	3	2	3	3	3	2	2	2	4	2	3	3	2	4	3	3	1	2	3	4	4	2	2	1	3	5	2	3	2	2
Complaints	Existence	0	0	1	0	0	0	0	0	0	0	0	0	0	0	0	0	0	0	0	1	0	0	0	0	0	0	0	0	1	0	0	1	1
and appeals	Specificity	1	1	1	0	1	0	0	0	1	1	0	0	0	0	0	1	1	0	0	0	0	0	0	0	0	0	0	1	0	0	0	0	0
	Operability	1	1	2	1	1	1	1	0	1	1	0	1	1	1	1	1	1	0	1	1	0	1	1	1	1	1	1	1	2	1	1	1	1
	Total	2	2	4	1	2	1	1	0	2	2	0	1	1	1	1	2	2	0	1	2	0	1	1	1	1	1	1	2	3	1	1	2	2
Data	Existence	0	0	0	1	0	0	0	0	0	0	0	0	0	1	0	0	0	0	0	0	0	0	1	0	0	0	1	0	0	0	1	1	0
protection	Specificity	0	1	0	1	1	0	1	1	0	0	0	0	1	1	0	1	0	0	0	0	1	0	1	0	1	0	0	1	1	0	0	0	0
	Operability	1	1	1	1	1	1	1	1	1	1	1	1	0	1	1	1	1	1	1	1	1	1	2	1	1	1	1	1	1	1	2	0	1
	Total	1	2	1	3	2	1	2	2	1	1	1	1	1	3	1	2	1	1	1	1	2	1	4	1	2	1	2	2	2	1	3	1	1
Disclaimer of	Existence	0	0	0	0	0	0	0	0	0	0	0	0	0	0	0	0	0	0	0	0	0	0	0	0	0	0	0	0	0	0	0	0	0
boundaries	Specificity	1	1	1	1	1	1	1	1	1	0	0	1	1	1	1	1	1	1	1	1	1	1	1	1	1	1	1	1	1	0	1	1	1
	Operability	1	1	1	1	1	1	1	1	1	1	1	1	1	1	1	1	1	1	1	1	1	1	1	1	1	1	1	1	1	1	1	1	1
	Total	2	2	2	2	2	2	2	2	2	1	1	2	2	2	2	2	2	2	2	2	2	2	2	2	2	2	2	2	2	1	2	2	2
Language	Existence	0	1	0	1	0	0	0	0	0	0	1	0	0	0	1	0	0	0	0	0	0	0	0	0	0	0	0	0	0	0	0	1	0
clarity	Specificity	0	1	0	0	0	0	0	0	0	0	0	0	0	1	0	0	0	0	0	0	0	0	1	0	0	1	0	0	0	0	1	0	0
	Operability	1	1	1	2	1	1	1	1	1	1	1	1	1	1	1	1	1	1	1	1	1	1	1	1	1	0	1	1	1	1	1	2	0
	Total	1	3	1	3	1	1	1	1	1	1	2	1	1	2	2	1	1	1	1	1	1	1	2	1	1	1	1	1	1	1	2	3	0
Voluntariness	Existence	0	0	0	1	0	1	0	1	1	1	0	0	1	0	0	1	0	0	0	0	1	1	0	1	1	1	0	0	0	1	1	0	0
	Specificity	0	0	0	0	0	0	0	0	0	0	0	0	0	1	0	1	0	0	0	0	0	0	0	0	0	0	0	0	0	0	1	0	0
	Operability	1	1	1	1	1	1	1	1	1	1	1	1	1	0	1	0	1	1	1	1	1	2	1	1	2	1	1	1	1	1	2	1	0
	Total	1	1	1	2	1	2	1	2	2	2	1	1	2	1	1	2	1	1	1	1	2	3	1	2	3	2	1	1	1	2	4	1	0
Client	Existence	0	1	0	0	1	0	0	1	1	0	0	1	1	1	1	1	0	1	0	0	0	1	0	1	0	0	0	0	1	1	1	1	1
obligations	Specificity	1	1	0	0	1	0	0	0	0	0	0	1	1	0	0	0	0	1	1	0	0	0	1	0	1	0	0	0	0	0	0	0	0
	Operability	1	1	1	1	0	1	1	0	0	1	0	2	1	1	1	0	1	1	1	1	0	2	1	2	1	1	1	1	1	1	2	2	2

		Total	2	3	1	1	2	1	1	1	1	1	0	4	3	2	2	1	1	3	2	1	0	3	2	3	2	1	1	1	2	2	3	3	3
	Counseling limitations	Existence	0	0	0	0	0	0	0	0	0	0	0	0	0	0	0	0	0	0	0	0	0	1	0	0	0	0	0	0	0	0	0	0	0
		Specificity	0	0	0	0	0	0	1	0	0	0	0	0	1	0	0	0	1	0	0	0	0	1	0	0	0	0	0	0	0	0	0	0	0
		Operability	1	1	1	1	1	0	1	1	1	1	0	1	1	1	1	1	1	1	1	1	0	2	1	1	0	1	1	1	1	1	1	1	1
		Total	1	1	1	1	1	0	2	1	1	1	0	1	2	1	1	1	2	1	1	1	0	4	1	1	0	1	1	1	1	1	1	1	1
	Counselor qualifications	Existence	1	1	1	0	0	0	1	1	1	1	0	0	1	1	0	1	1	1	1	1	0	0	1	1	1	1	1	1	1	0	1	1	1
		Specificity	1	0	0	0	0	0	0	0	0	0	0	0	0	0	0	0	1	0	0	0	0	0	0	0	0	0	0	0	0	0	0	0	0
		Operability	1	1	2	1	0	1	2	1	2	1	0	1	1	2	0	1	2	1	2	1	0	1	2	2	1	1	2	2	2	1	2	1	2
		Total	3	2	3	1	0	1	3	2	3	2	0	1	2	3	0	2	4	2	3	2	0	1	3	3	2	2	3	3	3	1	3	2	3
	Counseling modalities	Existence	0	1	1	0	1	0	0	0	0	0	0	0	1	0	0	1	0	0	0	0	0	0	0	0	0	0	1	0	0	0	0	0	0
		Specificity	0	1	1	1	0	1	0	0	0	0	0	0	1	0	0	1	0	0	0	0	1	0	0	0	0	1	0	0	0	0	0	0	0
		Operability	1	1	1	1	0	1	1	0	1	1	1	1	1	1	1	1	1	1	1	1	1	1	1	1	0	1	1	1	1	1	1	1	0
		Total	1	3	3	2	1	2	1	0	1	1	1	1	3	1	1	3	1	1	1	1	2	1	1	1	0	2	2	1	1	1	1	1	0
	Target population	Existence	1	0	0	0	0	0	1	1	1	1	1	1	1	1	1	1	1	1	1	0	0	1	1	1	1	0	1	1	1	1	0	1	1
		Specificity	2	0	0	0	0	0	0	1	2	1	1	2	1	0	2	0	1	1	1	0	0	1	0	1	1	0	0	0	1	0	1	0	1
		Operability	1	1	1	1	1	1	2	1	1	1	1	2	1	0	1	1	2	0	2	1	1	2	2	2	1	1	1	2	2	1	1	0	2
		Total	4	1	1	1	1	1	3	3	4	3	3	5	3	1	4	2	4	2	4	1	1	4	3	4	3	1	2	3	4	2	2	1	4
	Total score	Existence	11	10	13	11	12	7	8	14	13	9	8	12	14	10	10	11	11	13	11	7	7	13	11	12	12	7	10	10	13	7	14	15	12
		Specificity	18	12	15	12	15	12	8	14	15	8	5	11	13	14	12	13	16	11	11	7	5	12	16	10	14	10	6	12	15	5	15	9	10
		Operability	19	20	30	19	16	17	26	16	20	21	15	29	19	18	17	18	30	16	29	16	15	32	30	30	17	17	19	28	32	20	34	22	23
		Total	48	42	58	42	43	36	42	44	48	38	28	52	46	42	39	42	57	40	51	30	27	57	57	52	43	34	35	50	60	32	63	46	45
ChatGPT-5	Confidentiality	Existence	1	0	1	1	1	1	1	1	1	1	1	1	0	1	1	1	1	1	1	1	1	1	1	1	1	1	1	1	0	1	1	1	1
		Specificity	1	1	2	2	2	2	1	2	2	2	2	1	1	2	1	1	2	2	1	2	2	2	2	0	2	2	1	2	1	1	2	2	0
		Operability	1	1	2	2	2	2	2	1	0	1	2	1	0	1	1	1	2	1	2	2	0	2	2	2	1	1	1	1	1	1	2	1	1
		Total	3	2	5	5	5	5	4	4	3	4	5	3	1	4	3	3	5	4	4	5	3	5	5	3	4	4	3	4	2	3	5	4	2
	Exceptions to confidentiality	Existence	1	1	1	1	1	1	1	1	1	1	1	1	0	1	0	1	0	1	1	1	1	1	1	1	1	1	1	1	1	1	0	1	1
		Specificity	2	2	2	0	2	2	2	2	1	1	2	2	1	2	1	1	1	2	2	2	2	2	2	1	0	2	1	1	2	1	1	2	2
		Operability	0	2	2	1	1	1	2	1	1	1	1	2	1	1	1	0	1	2	2	1	1	2	2	1	1	1	1	2	2	1	1	1	1
		Total	3	5	5	2	4	4	5	4	3	3	4	5	2	4	2	2	2	5	5	4	4	5	5	3	2	4	3	4	5	3	2	4	4
	Client rights	Existence	1	1	1	1	1	1	1	1	1	1	1	0	1	1	1	1	1	1	0	0	1	1	1	1	1	1	1	1	1	1	1	1	0
		Specificity	2	1	2	2	1	1	1	0	0	0	2	1	1	0	2	1	1	1	1	1	1	2	1	2	1	1	2	0	1	0	1	2	1
		Operability	1	1	2	1	1	1	2	1	1	1	0	1	0	0	2	1	2	2	1	1	1	2	1	2	1	1	2	2	2	0	2	1	0
		Total	4	3	5	4	3	3	4	2	2	2	3	2	2	1	5	3	4	4	2	2	3	5	3	5	3	3	5	3	4	1	4	4	1

Guardian	Existence	1	1	1	0	1	1	0	1	1	1	0	0	1	0	1	1	0	1	0	1	1	1	1	1	1	1	1	0	1	0	1	1	1
consent	Specificity	2	2	2	1	1	2	1	2	2	2	1	0	2	1	1	1	1	2	1	2	2	2	1	2	1	0	0	1	2	0	2	2	2
	Operability	1	1	1	1	1	2	1	0	1	1	1	1	1	1	1	1	1	1	1	1	1	2	2	2	1	0	1	1	2	1	2	2	0
	Total	4	4	4	2	3	5	2	3	4	4	2	1	4	2	3	3	2	4	2	4	4	5	4	5	3	1	2	2	5	1	5	5	3
Goals and	Existence	1	1	1	0	1	1	1	0	1	0	1	0	1	0	1	1	0	0	0	1	1	1	1	1	0	1	1	0	1	0	1	1	1
scope	Specificity	0	2	2	0	2	2	1	1	1	0	2	1	2	1	1	1	0	1	1	1	1	2	2	1	1	2	1	1	1	0	2	1	1
	Operability	0	1	2	1	1	2	2	1	1	1	0	1	1	1	1	1	1	1	1	0	1	2	1	2	1	1	1	1	2	1	2	2	1
	Total	1	4	5	1	4	5	4	2	3	1	3	2	4	2	3	3	1	2	2	2	3	5	4	4	2	4	3	2	4	1	5	4	3
Format and	Existence	1	1	1	1	1	1	1	1	0	1	1	1	1	1	1	1	1	0	1	1	1	1	0	1	1	1	1	1	1	0	1	1	1
frequency	Specificity	1	0	2	1	2	1	1	2	1	1	2	2	2	1	2	1	1	1	1	0	1	0	1	2	2	0	2	1	2	1	2	0	1
	Operability	1	1	1	1	1	1	2	1	1	1	1	2	1	1	1	1	2	1	2	1	1	2	1	2	1	1	1	2	2	1	2	1	1
	Total	3	2	4	3	4	3	4	4	2	3	4	5	4	3	4	3	4	2	4	2	3	3	2	5	4	2	4	4	5	2	5	2	3
Fees and	Existence	1	1	1	1	1	1	0	1	1	0	1	1	1	1	1	1	1	1	1	1	0	1	1	1	1	1	1	1	1	1	1	1	1
cancellation	Specificity	1	1	2	2	1	1	1	1	1	1	1	0	1	1	1	1	1	2	1	1	1	1	1	1	1	1	0	1	1	0	0	1	1
policy	Operability	1	0	2	1	1	1	1	1	2	1	1	2	1	1	2	1	2	2	2	1	1	2	2	2	1	1	1	1	2	1	2	2	1
	Total	3	2	5	4	3	3	2	3	4	2	3	3	3	3	4	3	4	5	4	3	2	4	4	4	3	3	2	3	4	2	3	4	3
Recording	Existence	1	1	1	1	1	1	1	1	0	1	1	1	1	1	1	1	1	1	0	1	1	1	1	0	1	1	0	1	1	0	1	1	1
methods	Specificity	0	2	2	1	2	2	0	2	1	1	2	1	1	2	1	2	1	1	1	1	2	1	2	1	1	1	1	1	2	0	1	1	0
	Operability	1	1	2	1	2	1	2	1	1	1	1	2	1	1	1	1	2	2	1	0	1	1	2	1	1	0	1	2	2	1	1	1	1
	Total	2	4	5	3	5	4	3	4	2	3	4	4	3	4	3	4	4	4	2	2	4	3	5	2	3	2	2	4	5	1	3	3	2
Authorization	Existence	1	1	1	0	1	1	1	1	1	1	1	0	1	1	0	1	1	1	1	1	0	1	1	0	1	1	0	1	1	0	1	1	1
and revocation	Specificity	1	1	2	1	1	1	0	1	1	2	2	0	1	1	0	1	1	1	2	0	1	1	0	1	0	1	0	1	1	0	1	1	0
	Operability	1	0	2	1	1	1	2	1	1	1	1	1	1	1	1	1	2	2	2	1	1	2	2	1	0	1	1	2	2	1	2	0	0
	Total	3	2	5	2	3	3	3	3	3	4	4	1	3	3	1	3	4	4	5	2	2	4	3	2	1	3	1	4	4	1	4	2	1
Crisis	Existence	1	0	1	1	1	1	1	1	1	1	1	0	1	1	1	1	1	1	1	1	1	1	1	1	1	1	1	1	1	0	0	1	0
procedures	Specificity	2	1	1	0	1	0	0	1	0	0	2	1	1	1	1	0	1	2	0	0	0	0	0	2	1	1	1	0	1	0	1	0	1
	Operability	1	1	2	1	1	1	1	0	1	1	1	1	1	1	1	1	2	2	1	1	1	2	2	2	1	1	1	1	2	0	1	0	1
	Total	4	2	4	2	3	2	2	2	2	2	4	2	3	3	3	2	4	5	2	2	2	3	3	5	3	3	3	2	4	0	2	1	2
Complaints	Existence	0	0	1	0	0	0	0	0	1	0	0	0	0	0	0	0	0	0	0	0	1	0	1	0	0	1	0	0	0	0	0	0	0
and appeals	Specificity	0	1	2	0	1	1	0	1	1	1	1	0	0	1	0	1	1	1	1	0	1	1	0	1	0	1	0	1	1	0	1	1	1
	Operability	1	1	2	1	1	1	1	0	1	1	1	1	1	1	1	1	1	1	1	1	1	1	2	1	1	1	1	1	1	0	1	1	1
	Total	1	2	5	1	2	2	1	1	3	2	2	1	1	2	1	2	2	2	2	1	3	2	3	2	1	3	1	2	2	0	2	2	2
Data	Existence	1	1	1	1	0	0	1	0	0	0	1	0	1	0	0	0	0	0	0	1	0	0	1	0	0	1	0	0	0	0	1	0	1

protection	Specificity	0	2	2	1	1	0	1	1	1	0	2	0	0	1	1	1	1	0	1	1	1	0	1	0	0	0	0	0	1	0	0	0	0
	Operability	1	1	2	1	1	1	2	1	1	1	2	1	1	1	0	1	1	1	1	1	0	1	2	1	1	1	1	1	1	1	2	1	1
	Total	2	4	5	3	2	1	4	2	2	1	5	1	2	2	1	2	2	1	2	3	1	1	4	1	1	2	1	1	2	1	3	1	2
Disclaimer of	Existence	0	0	1	0	0	0	0	0	0	0	0	0	0	0	0	1	0	0	0	0	0	0	0	1	0	0	1	0	0	0	0	0	0
boundaries	Specificity	1	1	2	0	1	1	1	1	1	1	1	1	1	1	1	2	1	1	1	1	1	1	1	2	1	1	2	1	1	1	1	1	1
	Operability	0	1	2	1	1	1	1	1	1	1	1	1	1	1	1	1	1	1	1	1	1	1	1	1	1	1	1	1	1	1	1	1	1
	Total	1	2	5	1	2	2	2	2	2	2	2	2	2	2	2	4	2	2	2	2	2	2	2	4	2	2	4	2	2	2	2	2	2
Language	Existence	0	0	0	0	0	0	0	0	0	0	0	0	0	0	0	0	0	0	0	1	0	0	0	0	0	0	0	1	0	0	0	0	1
clarity	Specificity	0	0	0	0	0	0	0	0	0	0	1	0	0	0	0	0	0	1	0	0	0	0	0	0	0	0	0	0	0	1	0	0	0
	Operability	1	1	1	1	1	1	1	1	1	1	1	1	0	1	1	1	1	1	1	1	1	1	1	1	1	1	1	2	1	1	1	0	2
	Total	1	1	1	1	1	1	1	1	1	1	2	1	0	1	1	1	1	2	1	2	1	1	1	1	1	1	1	3	1	2	1	0	3
Voluntariness	Existence	1	1	1	0	0	1	0	1	1	1	1	1	1	1	1	1	1	0	0	1	1	0	0	1	1	0	0	1	1	1	1	1	1
	Specificity	1	0	0	0	1	0	0	0	0	0	0	0	0	0	0	0	0	0	0	0	1	0	0	1	0	0	0	0	0	0	1	1	0
	Operability	1	0	2	1	0	2	1	1	1	1	2	2	1	1	1	1	2	1	1	1	1	1	1	1	2	1	1	2	1	1	2	1	1
	Total	3	1	3	1	1	3	1	2	2	2	3	3	2	2	2	2	3	1	1	2	3	1	1	3	3	1	1	3	2	2	4	3	2
Client	Existence	0	0	1	0	0	0	0	1	0	1	1	1	1	1	0	0	1	1	1	1	1	0	0	1	1	1	1	1	1	1	1	0	1
obligations	Specificity	0	0	1	0	0	1	0	1	0	0	0	1	1	1	1	1	0	0	1	0	0	0	0	0	1	0	1	0	1	0	1	0	0
	Operability	1	0	2	0	1	1	1	1	1	1	1	2	0	1	1	0	2	2	2	1	1	1	1	2	1	0	1	2	2	1	2	1	1
	Total	1	0	4	0	1	2	1	3	1	2	2	4	2	3	2	1	3	3	4	2	2	1	1	3	3	1	3	3	4	2	4	1	2
Counseling	Existence	0	1	0	1	0	0	0	0	0	0	1	0	0	0	0	0	0	1	0	0	0	0	1	0	0	1	0	1	0	0	0	1	0
limitations	Specificity	1	0	0	0	0	0	0	0	0	0	0	0	0	1	0	0	0	0	1	1	0	0	0	0	0	1	1	0	0	0	0	0	0
	Operability	0	1	1	0	0	1	1	1	1	1	0	1	1	0	0	1	1	2	1	1	1	1	2	1	1	2	0	2	1	1	1	2	0
	Total	1	2	1	1	0	1	1	1	1	1	1	1	1	1	0	1	1	3	2	2	1	1	3	1	1	4	1	3	1	1	1	3	0
Counselor	Existence	1	1	0	1	1	1	1	1	1	1	1	1	1	0	0	1	1	1	1	1	1	1	1	1	1	1	1	1	1	1	1	1	1
qualifications	Specificity	0	0	0	0	0	2	0	1	0	0	0	1	0	0	1	0	2	1	0	0	0	0	0	0	0	0	0	0	0	0	0	0	0
	Operability	1	1	1	1	1	1	2	1	1	0	2	2	1	1	1	0	2	2	1	1	1	2	2	2	0	1	0	2	2	1	2	1	1
	Total	2	2	1	2	2	4	3	3	2	1	3	4	2	1	2	1	5	4	2	2	2	3	3	3	1	2	1	3	3	2	3	2	2
Counseling	Existence	0	1	1	0	1	1	1	0	0	1	1	1	1	0	0	0	0	0	0	0	0	0	0	0	1	1	1	0	0	0	0	0	0
modalities	Specificity	1	1	2	1	1	2	2	1	1	0	2	1	2	0	0	1	1	0	1	1	1	0	0	0	2	2	0	1	1	0	0	0	0
	Operability	1	0	2	1	1	1	2	1	1	1	1	2	1	0	1	1	1	1	1	1	1	1	1	1	0	1	1	1	1	1	1	1	1
	Total	2	2	5	2	3	4	5	2	2	2	4	4	4	0	1	2	2	1	2	2	2	1	1	1	3	4	2	2	2	1	1	1	1
Target	Existence	1	0	1	0	0	0	1	1	1	1	1	1	0	1	1	1	1	1	1	0	0	0	1	1	1	0	1	0	1	1	1	1	1
population	Specificity	2	0	0	0	0	0	0	1	2	1	1	1	1	0	2	0	1	1	1	0	0	1	1	1	0	0	0	0	1	1	1	2	1

		Operability	1	1	2	1	0	1	2	1	1	2	1	2	1	1	1	2	1	2	1	1	1	1	2	2	0	1	1	1	2	1	2	1	1
		Total	4	1	3	1	0	1	3	3	4	4	3	4	2	2	4	3	3	4	3	1	1	2	4	4	1	1	2	1	4	3	4	4	3
	Total score	Existence	14	13	17	10	12	13	12	13	12	13	16	10	13	11	10	14	11	12	9	14	12	11	14	13	14	16	13	13	13	8	13	14	14
		Specificity	18	18	28	12	20	21	12	21	16	13	26	14	18	17	17	16	17	20	18	14	18	16	15	18	14	16	13	12	20	6	18	17	12
		Operability	16	16	35	19	19	24	31	17	20	20	21	29	16	17	20	18	30	30	26	19	18	30	32	30	17	18	19	30	32	17	32	21	17
		Total	48	47	80	41	51	58	55	51	48	46	63	53	47	45	47	48	58	62	53	47	48	57	61	61	45	50	45	55	65	31	63	52	43
Grok-4	Confidentiality	Existence	1	1	1	1	1	1	0	1	1	1	1	0	1	1	0	1	1	0	1	1	1	1	0	0	1	1	1	1	1	1	1	1	1
		Specificity	2	2	1	2	2	2	0	1	2	2	1	1	2	2	1	2	1	1	1	2	2	2	1	1	2	2	1	1	1	0	1	2	2
		Operability	1	1	2	1	1	2	1	0	1	1	1	1	1	1	1	2	2	0	2	1	1	2	1	1	1	0	2	2	2	1	2	0	1
		Total	4	4	4	4	4	5	1	2	4	4	3	2	4	4	2	5	4	1	4	4	4	5	2	2	4	3	4	4	4	2	4	3	4
	Exceptions to confidentiality	Existence	0	1	1	1	1	1	1	1	1	1	1	0	1	1	1	1	1	1	1	1	1	1	1	1	1	0	1	0	0	1	1	1	1
		Specificity	1	2	2	0	2	1	1	1	2	0	2	1	1	2	2	1	2	1	2	1	2	2	2	2	2	1	1	1	1	1	2	1	2
		Operability	1	1	2	0	2	2	2	1	1	1	1	1	1	1	1	0	2	1	2	1	2	2	2	2	2	1	1	1	1	0	2	1	2
		Total	2	4	5	1	5	4	4	3	4	2	4	2	3	4	4	2	5	3	5	3	5	5	5	5	5	2	3	2	2	2	5	3	5
	Client rights	Existence	1	1	1	1	1	1	1	0	1	0	1	1	1	1	1	0	0	1	1	1	1	1	1	1	1	1	0	1	0	0	1	1	0
		Specificity	2	2	2	2	1	2	2	1	2	1	2	2	2	2	1	1	1	2	1	1	2	2	2	1	2	1	1	2	1	1	2	1	1
		Operability	1	0	2	1	1	1	1	0	0	1	0	1	1	0	1	1	1	1	2	1	1	2	2	2	2	1	1	2	1	1	1	1	1
		Total	4	3	5	4	3	4	4	1	3	2	3	4	4	3	3	2	2	4	4	3	4	5	5	4	5	3	2	5	2	2	4	3	2
	Guardian consent	Existence	1	1	1	0	1	1	0	1	0	0	1	0	1	0	1	0	1	1	1	0	1	1	0	1	1	0	0	0	1	0	1	1	1
		Specificity	2	1	2	1	2	2	1	1	1	1	2	1	2	0	2	1	2	1	1	1	2	2	1	1	2	1	1	1	1	1	1	2	2
		Operability	1	1	2	1	1	1	1	2	1	1	1	1	1	0	2	1	2	1	1	1	1	2	1	2	2	1	1	1	2	1	2	1	1
		Total	4	3	5	2	4	4	2	4	2	2	4	2	4	0	5	2	5	3	3	2	4	5	2	4	5	2	2	2	4	2	4	4	4
	Goals and scope	Existence	1	1	1	1	1	1	1	1	1	1	1	1	1	1	1	1	1	1	1	1	1	1	1	1	1	1	1	0	1	1	1	1	1
		Specificity	1	1	1	2	2	1	2	0	2	1	2	2	1	2	2	2	1	1	0	2	2	1	2	2	1	1	1	1	1	2	2	0	2
		Operability	1	1	2	1	2	1	2	0	1	1	1	2	0	1	2	1	2	1	2	0	1	2	1	2	2	0	1	1	1	1	2	0	0
		Total	3	3	4	4	5	3	5	1	4	3	4	5	2	4	5	4	4	3	3	3	4	4	4	5	4	2	3	2	3	4	5	1	3
	Format and frequency	Existence	1	1	1	0	1	1	1	1	1	1	0	1	1	1	1	1	1	1	1	1	1	1	1	0	1	1	1	1	1	1	1	0	1
		Specificity	2	2	2	1	2	2	2	2	2	2	1	2	2	2	2	2	2	2	2	1	0	2	1	1	2	1	2	2	2	1	1	0	2
		Operability	1	1	2	1	1	1	2	1	1	1	1	2	1	1	1	1	2	1	2	2	2	2	2	1	2	0	1	2	2	1	2	1	1
		Total	4	4	5	2	4	4	5	4	4	4	2	5	4	4	4	4	5	4	5	4	3	5	4	2	5	2	4	5	5	3	4	1	4
	Fees and cancellation	Existence	0	1	1	1	1	1	1	1	1	0	1	1	1	1	1	1	1	1	1	1	1	1	1	1	1	1	0	1	1	1	1	1	1
		Specificity	1	1	1	0	1	1	2	1	1	1	1	1	2	1	1	1	1	1	1	1	0	1	1	1	1	0	1	1	1	2	1	1	0

policy	Operability	1	1	2	1	1	1	2	1	1	1	1	2	1	2	2	1	2	1	1	1	1	1	2	2	1	1	1	2	2	1	2	1	1
	Total	2	3	4	2	3	3	5	3	3	2	3	4	4	4	4	3	4	3	3	3	2	3	4	4	3	2	2	4	4	4	4	3	2
Recording methods	Existence	1	1	1	1	0	0	1	1	1	1	0	1	1	0	1	0	1	1	1	1	1	1	1	1	1	1	0	1	1	0	1	1	1
	Specificity	1	2	1	1	1	1	2	2	1	2	0	2	2	1	1	1	1	1	1	0	2	2	2	1	2	2	1	1	2	0	1	2	1
	Operability	1	1	2	1	1	1	2	0	1	1	0	2	0	1	1	1	2	2	2	1	1	2	2	1	2	1	1	2	2	1	2	2	1
	Total	3	4	4	3	2	2	5	3	3	4	0	5	3	2	3	2	4	4	4	2	4	5	5	3	5	4	2	4	5	1	4	5	3
Authorization and revocation	Existence	1	1	1	1	1	1	1	1	1	1	1	1	1	1	1	1	1	0	1	0	1	1	1	0	1	1	1	1	1	1	1	1	1
	Specificity	1	1	0	0	1	1	2	0	1	2	1	0	2	0	1	1	2	0	1	1	0	1	0	1	1	2	0	1	0	0	0	1	2
	Operability	1	1	1	1	1	0	2	1	1	1	1	2	1	1	1	1	2	0	2	1	1	2	1	1	2	1	1	2	2	1	2	0	1
	Total	3	3	2	2	3	2	5	2	3	4	3	3	4	2	3	3	5	0	4	2	2	4	2	2	4	4	2	4	3	2	3	2	4
Crisis procedures	Existence	1	1	1	1	1	1	1	1	1	1	1	1	1	0	1	1	0	1	1	1	1	1	1	1	1	1	1	1	0	1	1	1	1
	Specificity	0	0	0	1	1	1	1	1	0	1	1	1	1	0	2	0	0	2	1	0	0	1	0	2	2	1	0	0	1	1	2	1	2
	Operability	1	0	2	0	1	1	2	0	1	1	1	2	1	1	1	1	1	1	2	1	1	1	2	2	2	1	1	2	1	1	2	1	0
	Total	2	1	3	2	3	3	4	2	2	3	3	4	3	1	4	2	1	4	4	2	2	3	3	5	5	3	2	3	2	3	5	3	3
Complaints and appeals	Existence	1	1	1	0	0	1	0	0	0	0	0	1	0	0	1	0	1	0	0	0	1	0	1	0	0	1	1	1	0	0	1	0	0
	Specificity	2	1	1	0	1	2	0	0	1	1	1	0	0	1	1	1	1	0	1	1	0	1	0	0	1	1	0	0	1	0	0	1	1
	Operability	1	1	2	1	1	1	1	1	1	1	1	2	1	1	1	1	2	1	1	1	1	1	2	1	1	1	1	2	1	1	2	1	1
	Total	4	3	4	1	2	4	1	1	2	2	2	3	1	2	3	2	4	1	2	2	2	2	3	1	2	3	2	3	2	1	3	2	2
Data protection	Existence	0	1	0	1	1	1	1	0	1	1	0	1	1	1	1	1	1	1	0	1	1	0	0	0	1	0	0	0	1	0	1	1	0
	Specificity	0	1	0	0	1	1	2	0	1	2	0	2	1	2	0	2	0	0	0	1	1	0	1	0	2	1	1	1	1	0	0	1	0
	Operability	1	1	1	1	0	1	2	0	1	1	1	2	1	2	1	0	2	1	1	1	1	1	1	1	2	1	1	1	2	1	2	1	1
	Total	1	3	1	2	2	3	5	0	3	4	1	5	3	5	2	3	3	2	1	3	3	1	2	1	5	2	2	2	4	1	3	3	1
Disclaimer of boundaries	Existence	0	0	0	0	0	0	0	0	0	0	0	0	0	0	0	0	0	0	0	0	0	0	0	1	0	1	0	0	0	0	0	1	0
	Specificity	1	1	1	1	1	1	1	0	1	1	1	1	1	1	1	1	1	1	1	1	1	1	1	2	1	2	1	1	1	1	1	1	1
	Operability	1	0	1	1	1	1	1	1	1	1	1	1	0	0	1	1	1	1	1	1	1	1	1	1	1	1	1	1	1	1	1	1	1
	Total	2	1	2	2	2	2	2	1	2	2	2	2	1	1	2	2	2	2	2	2	2	2	2	4	2	4	2	2	2	2	2	3	2
Language clarity	Existence	0	1	1	0	0	0	1	1	0	1	0	0	0	0	0	0	0	0	1	1	0	0	1	0	0	0	0	1	0	0	0	1	1
	Specificity	0	0	0	0	0	1	0	0	0	0	0	0	0	1	0	0	0	0	1	0	0	0	0	0	1	0	0	1	0	0	0	0	1
	Operability	1	1	2	1	1	1	2	1	1	1	1	1	1	1	1	1	1	1	2	0	1	1	2	1	1	1	1	2	1	1	1	1	2
	Total	1	2	3	1	1	2	3	2	1	2	1	1	1	2	1	1	1	1	4	1	1	1	3	1	2	1	1	4	1	1	1	2	4
Voluntariness	Existence	1	0	0	0	0	1	1	0	0	1	1	1	1	1	0	1	0	1	1	0	1	1	1	1	1	1	1	1	0	1	1	0	0
	Specificity	0	0	1	0	0	1	0	1	0	0	1	0	0	0	0	0	0	0	0	0	0	0	0	0	0	0	0	0	0	0	0	0	0

	Operability	1	1	1	1	0	1	1	1	1	1	1	1	1	2	0	0	1	1	1	1	1	2	2	2	2	1	1	2	1	2	2	1	1
	Total	2	1	2	1	0	3	2	2	1	2	3	2	2	3	0	1	1	2	2	1	2	3	3	3	3	2	2	3	1	3	3	1	1
Client obligations	Existence	0	1	0	1	1	1	0	1	1	1	0	0	1	1	1	0	1	1	0	0	0	1	0	1	1	1	1	0	1	1	1	1	1
	Specificity	0	1	1	1	0	0	0	2	0	1	0	1	1	2	1	0	1	1	1	0	0	0	0	0	0	0	0	0	1	0	1	0	1
	Operability	1	1	1	1	0	1	1	1	1	0	1	1	0	2	1	1	2	2	1	1	1	2	1	2	2	1	1	1	2	2	2	2	1
	Total	1	3	2	3	1	2	1	4	2	2	1	2	2	5	3	1	4	4	2	1	1	3	1	3	3	2	2	1	4	3	4	3	3
Counseling limitations	Existence	0	1	0	0	0	1	1	0	1	0	0	0	1	0	1	0	0	0	0	1	0	0	0	0	0	0	0	0	0	0	0	0	1
	Specificity	0	0	0	0	0	1	1	0	2	1	0	0	0	0	1	0	1	0	0	1	0	0	0	0	1	0	0	0	0	0	0	0	0
	Operability	1	1	1	1	1	1	2	1	1	1	1	1	0	1	2	1	1	1	1	1	0	1	1	1	1	1	1	1	1	1	1	1	1
	Total	1	2	1	1	1	3	4	1	4	2	1	1	1	1	4	1	2	1	1	3	0	1	1	1	2	1	1	1	1	1	1	1	2
Counselor qualifications	Existence	1	1	1	1	1	1	1	1	0	1	1	0	1	1	1	1	1	1	1	1	0	1	1	1	1	0	1	1	1	1	1	1	1
	Specificity	0	1	0	0	0	0	1	0	0	0	1	0	0	0	0	0	0	0	0	0	0	0	0	0	0	0	0	0	0	0	0	0	0
	Operability	1	1	2	1	2	1	2	1	0	2	2	1	1	1	1	1	2	1	2	1	1	2	2	2	2	1	1	2	2	1	1	1	1
	Total	2	3	3	2	3	2	4	2	0	3	4	1	2	2	2	2	3	2	3	2	1	3	3	3	3	1	2	3	3	2	2	2	2
Counseling modalities	Existence	0	1	1	0	1	0	0	0	0	1	0	0	0	0	0	0	0	0	1	0	1	0	0	0	1	1	1	0	0	0	1	0	1
	Specificity	1	1	2	1	1	1	1	1	1	2	0	1	0	1	1	1	1	1	1	1	2	0	1	1	1	1	1	1	1	1	2	1	1
	Operability	1	1	2	1	1	1	1	1	1	1	1	1	1	1	1	1	1	1	2	1	2	1	1	1	2	1	1	1	1	1	2	0	1
	Total	2	3	5	2	3	2	2	2	2	4	1	2	1	2	2	2	2	2	4	2	5	1	2	2	4	3	3	2	2	2	5	1	3
Target population	Existence	1	0	1	1	0	1	1	1	1	1	1	1	1	1	1	1	1	1	1	1	0	1	1	1	1	0	1	1	0	1	1	1	1
	Specificity	2	1	0	1	0	1	0	0	2	1	2	1	1	1	1	1	0	0	0	1	0	1	2	2	1	0	1	0	0	0	0	0	1
	Operability	1	1	2	1	1	1	2	1	1	1	1	2	1	2	1	1	2	1	2	2	1	2	2	2	2	0	1	2	1	0	2	1	1
	Total	4	2	3	3	1	3	3	2	4	3	4	4	3	4	3	3	3	2	3	4	1	4	5	5	4	0	3	3	1	1	3	2	3
Total score	Existence	12	17	15	12	13	16	14	13	13	14	11	11	16	12	15	11	13	13	15	13	14	14	13	12	16	13	12	12	10	11	17	15	15
	Specificity	19	21	18	14	19	23	21	14	22	22	19	19	21	21	21	18	18	15	16	16	16	19	17	18	25	17	13	15	16	11	17	15	22
	Operability	20	17	34	18	20	21	32	15	18	20	19	29	15	22	23	18	33	20	32	20	22	32	31	30	34	16	21	32	29	20	35	18	20
	Total	51	55	67	44	52	60	67	42	53	56	49	59	52	55	59	47	64	48	63	49	52	65	61	60	75	46	46	59	55	42	69	48	57

Supplementary Data S4. Table of detailed ratings by expert 04 for all documents

Version	Indicator	Dimension	Document ID																																
			01	02	03	04	05	06	07	08	09	10	11	12	13	14	15	16	17	18	19	20	21	22	23	24	25	26	27	28	29	30	31	32	33
Original	Confidentiality	Existence	1	1	1	1	1	1	1	1	1	1	0	1	1	1	1	1	1	1	1	1	1	1	1	1	1	1	0	1	1	1	1	1	1
		Specificity	0	0	2	1	2	1	0	0	2	2	1	0	0	1	0	1	0	0	0	2	0	2	2	0	2	1	1	1	0	0	0	1	0
		Operability	1	0	2	1	0	1	2	1	2	1	1	2	1	1	1	1	2	1	1	2	2	2	2	2	1	1	1	2	2	1	2	0	1
		Total	2	1	5	3	3	3	3	2	5	4	2	3	2	3	2	3	3	2	2	5	3	5	5	3	4	3	2	4	3	2	3	2	2
	Exceptions to confidentiality	Existence	1	0	1	1	1	1	1	1	1	1	1	1	1	0	1	0	1	1	1	0	0	0	1	1	1	0	0	1	1	0	1	1	1
		Specificity	2	1	0	1	1	1	0	2	1	1	1	1	0	1	0	0	1	1	1	0	1	1	1	0	1	0	0	1	1	0	1	1	0
		Operability	1	1	2	2	1	1	2	1	0	0	2	2	1	1	1	1	2	1	2	1	1	1	2	2	2	1	1	1	2	0	2	1	2
		Total	4	2	3	4	3	3	3	4	2	2	4	4	2	2	2	1	4	3	4	1	2	2	4	3	4	1	1	3	4	0	4	3	3
	Client rights	Existence	1	1	1	0	1	1	1	1	0	1	1	1	1	1	1	1	1	1	1	1	1	1	1	1	1	1	1	1	1	1	1	1	1
		Specificity	0	1	1	1	0	1	1	1	1	0	0	0	0	2	1	1	0	1	1	0	2	1	1	1	1	1	2	0	1	2	2	2	1
		Operability	1	1	2	0	1	1	2	2	1	2	1	2	0	1	0	1	2	1	2	1	1	2	2	2	2	2	1	2	1	1	2	1	1
		Total	2	3	4	1	2	3	4	4	2	3	2	3	1	4	2	3	3	3	4	2	4	4	4	4	4	4	4	3	3	4	5	4	3
	Guardian consent	Existence	1	1	1	0	1	1	0	0	0	0	0	0	1	0	0	0	1	1	1	0	1	1	0	1	1	0	1	0	1	0	1	1	1
		Specificity	1	0	2	0	2	1	0	1	1	1	1	1	1	1	1	1	1	1	1	1	1	2	0	1	0	1	1	1	1	1	1	1	1
		Operability	0	1	2	1	1	0	1	0	1	1	1	1	1	0	1	0	2	1	2	1	2	2	1	2	2	1	0	1	2	1	2	1	2
		Total	2	2	5	1	4	2	1	1	2	2	2	2	3	1	2	1	4	3	4	2	4	5	1	4	3	2	2	2	4	2	4	3	4
	Goals and scope	Existence	1	0	1	0	1	0	0	1	0	0	0	0	1	0	1	0	1	1	1	0	1	1	1	1	1	0	1	0	1	0	1	1	1
		Specificity	1	0	1	0	2	1	0	1	1	0	0	0	0	0	1	0	0	1	1	0	0	0	2	1	0	0	0	1	1	0	2	0	2
		Operability	1	1	2	1	1	1	1	1	1	1	1	1	1	1	1	1	1	1	2	1	2	1	2	2	1	1	1	1	2	0	2	1	2
		Total	3	1	4	1	4	2	1	3	2	1	1	1	2	1	3	1	2	3	4	1	3	2	5	4	2	1	2	2	4	0	5	2	5
	Format and frequency	Existence	1	1	1	1	1	1	1	1	1	1	1	1	1	1	1	1	0	1	1	1	0	1	0	0	1	1	0	1	1	1	1	1	1
		Specificity	2	1	2	2	2	2	0	2	1	2	1	2	2	1	2	0	1	2	2	1	1	0	1	1	1	1	1	1	2	1	1	0	2
		Operability	2	1	2	2	1	0	2	1	1	1	1	2	1	0	2	1	1	1	2	1	1	2	1	1	1	1	1	1	2	1	2	1	1
		Total	5	3	5	5	4	3	3	4	3	4	3	5	4	2	5	2	2	4	5	3	2	3	2	2	3	3	2	3	5	3	4	2	4
	Fees and cancellation policy	Existence	1	1	1	1	0	1	1	1	1	1	1	1	1	1	1	1	1	0	1	0	1	1	1	1	1	0	1	1	1	0	0	1	1
		Specificity	1	1	1	0	0	0	1	1	0	1	1	1	2	2	1	1	2	1	1	0	0	1	1	1	1	0	0	1	2	1	1	1	1
		Operability	1	2	2	0	1	1	2	1	1	1	2	2	1	1	1	1	2	1	1	1	1	2	2	2	1	1	2	2	2	1	1	1	1
		Total	3	4	4	1	1	2	4	3	2	3	4	4	4	4	3	3	5	2	3	1	2	4	4	4	3	1	3	4	5	2	2	3	3
	Recording methods	Existence	1	0	1	1	1	0	0	1	1	1	0	1	1	1	1	1	1	0	1	1	0	1	1	1	0	0	0	1	1	0	0	1	1
		Specificity	1	1	1	1	2	1	1	2	1	1	0	2	2	2	2	2	1	1	2	0	1	2	2	1	1	1	1	1	1	1	1	1	0

	Operability	0	1	2	2	0	0	1	1	1	1	1	2	1	1	1	1	2	0	2	1	1	2	2	2	1	1	1	1	2	1	1	1	1
	Total	2	2	4	4	3	1	2	4	3	3	1	5	4	4	4	4	4	1	5	2	2	5	5	4	2	2	2	3	4	2	2	3	2
Authorization and revocation	Existence	0	1	1	1	1	0	1	1	1	1	0	1	1	0	0	1	1	0	1	1	0	1	1	1	1	1	1	1	1	0	1	1	1
	Specificity	1	0	1	0	1	0	1	0	1	1	1	1	0	0	0	0	0	0	0	0	0	1	0	0	0	1	0	0	0	1	0	2	0
	Operability	1	1	2	1	1	1	1	1	1	1	0	2	1	1	1	1	2	1	2	1	1	2	2	2	1	1	1	2	2	1	2	1	1
	Total	2	2	4	2	3	1	3	2	3	3	1	4	2	1	1	2	3	1	3	2	1	4	3	3	2	3	2	3	3	2	3	4	2
Crisis procedures	Existence	1	1	1	0	1	1	1	1	1	1	1	1	1	1	1	1	1	0	1	1	1	1	1	1	1	1	1	1	1	1	1	1	1
	Specificity	0	0	1	1	1	0	0	1	0	0	1	1	0	0	1	0	0	1	1	1	0	0	1	2	1	0	0	0	1	2	0	0	1
	Operability	1	1	2	0	1	0	2	1	1	1	0	2	1	0	1	1	2	1	2	0	1	2	1	2	0	1	1	2	2	1	2	1	1
	Total	2	2	4	1	3	1	3	3	2	2	2	4	2	1	3	2	3	2	4	2	2	3	3	5	2	2	2	3	4	4	3	2	3
Complaints and appeals	Existence	0	0	0	0	0	0	0	0	1	0	0	1	0	0	0	0	0	0	0	0	0	0	0	0	0	0	0	0	0	1	0	0	0
	Specificity	1	1	1	0	1	0	1	0	1	1	1	0	0	0	0	1	0	0	0	0	1	1	0	0	0	0	0	0	0	0	0	1	0
	Operability	1	0	1	1	1	1	1	1	1	1	1	2	0	1	1	1	1	1	1	1	1	1	1	1	1	1	1	1	1	1	1	1	1
	Total	2	1	2	1	2	1	2	1	3	2	2	3	0	1	1	2	1	1	1	1	2	2	1	1	1	1	1	1	1	2	1	2	1
Data protection	Existence	1	0	1	1	0	0	0	0	0	0	0	0	0	1	0	0	1	0	0	0	0	0	1	0	0	0	1	0	0	0	1	1	0
	Specificity	0	0	0	0	1	0	0	1	1	0	0	1	1	2	0	1	0	0	1	0	0	0	2	0	0	0	0	0	0	1	0	0	0
	Operability	1	0	2	0	1	1	1	1	1	1	1	1	1	1	1	1	2	1	1	1	1	1	2	1	1	1	1	1	1	1	1	1	1
	Total	2	0	3	1	2	1	1	2	2	1	1	2	2	4	1	2	3	1	2	1	1	1	5	1	1	1	2	1	1	2	2	2	1
Disclaimer of boundaries	Existence	1	0	0	0	1	0	0	0	0	0	0	0	0	0	0	0	0	1	0	0	0	0	0	0	1	0	0	0	0	0	0	0	0
	Specificity	2	1	1	0	2	1	1	1	1	0	0	1	1	1	1	1	1	2	1	1	1	1	1	1	1	1	1	1	1	0	1	1	1
	Operability	0	1	1	1	1	1	1	1	1	1	1	1	1	0	1	0	1	1	1	1	1	1	1	1	1	0	1	1	1	1	1	1	1
	Total	3	2	2	1	4	2	2	2	2	1	1	2	2	1	2	1	2	4	2	2	2	2	2	2	3	1	2	2	2	1	2	2	2
Language clarity	Existence	0	0	0	0	0	0	0	0	0	0	0	0	0	0	0	0	0	0	0	0	0	0	0	0	0	0	0	0	0	0	0	0	0
	Specificity	0	0	0	0	0	0	0	0	0	0	0	0	0	0	0	0	0	0	0	0	0	0	1	0	1	0	0	0	0	0	0	0	1
	Operability	1	1	1	0	1	1	1	0	1	1	1	1	1	1	1	1	1	1	1	1	1	1	1	1	1	1	0	1	1	1	1	1	1
	Total	1	1	1	0	1	1	1	0	1	1	1	1	1	1	1	1	1	1	1	1	1	1	2	1	2	1	0	1	1	1	1	1	2
Voluntariness	Existence	0	0	1	0	0	1	0	0	1	1	1	0	1	0	1	1	0	0	0	0	1	1	0	1	1	1	0	0	0	1	1	1	0
	Specificity	0	0	0	0	1	0	0	0	0	0	0	0	0	1	0	0	0	0	0	0	0	0	0	0	0	0	0	1	1	0	0	0	0
	Operability	0	1	2	1	1	1	1	1	2	1	1	1	2	1	1	1	1	0	1	1	1	2	1	2	2	1	1	1	1	0	2	1	1
	Total	0	1	3	1	2	2	1	1	3	2	2	1	3	2	2	2	1	0	1	1	2	3	1	3	3	2	1	2	2	1	3	2	1
Client obligations	Existence	0	1	1	0	1	1	0	1	0	0	0	1	1	0	1	0	0	1	1	0	0	1	0	1	0	0	0	0	1	0	1	1	1
	Specificity	0	0	0	0	0	1	0	1	0	0	0	0	1	0	0	0	0	0	0	0	0	0	0	0	1	0	0	0	0	0	0	0	1
	Operability	1	1	2	1	1	1	1	1	1	1	0	1	0	1	1	1	1	1	0	1	1	2	1	2	1	1	0	1	2	1	2	1	1

		Total	1	2	3	1	2	3	1	3	1	1	0	2	2	1	2	1	1	2	1	1	1	3	1	3	2	1	0	1	3	1	3	2	3
	Counseling limitations	Existence	0	0	1	1	0	0	0	0	0	0	0	0	0	0	0	0	0	0	0	0	0	1	0	0	0	0	0	0	1	0	0	0	0
		Specificity	0	1	1	0	0	0	0	0	1	1	0	1	0	0	0	0	0	0	0	0	0	0	0	0	0	0	0	0	0	1	0	0	1
		Operability	1	1	2	1	1	0	1	1	1	1	1	1	1	1	1	1	1	1	1	1	1	2	1	1	1	0	1	1	2	1	1	1	0
		Total	1	2	4	2	1	0	1	1	2	2	1	2	1	1	1	1	1	1	1	1	1	3	1	1	1	0	1	1	3	2	1	1	1
	Counselor qualifications	Existence	1	1	1	1	1	0	1	0	0	1	1	1	1	0	0	1	1	1	1	1	0	1	1	1	1	1	1	1	1	0	1	1	1
		Specificity	0	0	0	0	0	0	0	0	0	0	0	0	0	0	0	0	1	0	0	0	0	0	0	0	0	0	0	0	0	0	0	0	0
		Operability	1	1	2	2	1	1	2	1	1	2	1	2	1	1	1	1	2	1	2	1	1	2	2	2	1	1	1	2	2	0	2	1	1
		Total	2	2	3	3	2	1	3	1	1	3	2	3	2	1	1	2	4	2	3	2	1	3	3	3	2	2	2	3	3	0	3	2	2
	Counseling modalities	Existence	0	1	1	0	1	0	0	0	0	0	0	1	1	0	0	0	0	0	1	0	1	0	0	0	1	1	1	0	0	0	0	1	0
		Specificity	0	1	1	1	0	1	0	0	0	0	0	0	1	0	0	0	0	0	0	0	2	0	0	0	0	0	0	0	0	0	0	1	0
		Operability	1	0	2	0	1	1	1	1	0	1	1	2	1	1	1	1	1	1	2	1	1	1	1	1	1	1	2	1	1	1	1	1	1
		Total	1	2	4	1	2	2	1	1	0	1	1	3	3	1	1	1	1	1	3	1	4	1	1	1	2	2	3	1	1	1	1	3	1
	Target population	Existence	1	0	1	0	0	0	1	1	1	1	1	0	0	1	1	1	1	1	1	0	0	1	1	1	1	0	1	1	1	1	1	0	1
		Specificity	1	0	0	0	0	0	0	1	2	1	1	1	1	0	2	0	2	2	1	0	0	0	2	1	1	0	0	0	2	0	2	1	1
		Operability	0	1	2	1	1	1	2	1	1	0	1	1	0	1	1	1	2	1	1	1	1	2	2	2	1	1	0	1	2	1	2	1	0
		Total	2	1	3	1	1	1	3	3	4	2	3	2	1	2	4	2	5	4	3	1	1	3	5	4	3	1	1	2	5	2	5	2	2
	Total score	Existence	13	10	17	9	13	9	9	11	10	11	8	12	14	8	11	10	12	10	14	7	8	14	11	13	14	8	10	10	14	7	13	15	13
		Specificity	13	9	16	8	18	11	6	15	15	12	9	13	12	14	12	9	10	13	13	6	10	12	17	10	12	7	7	9	14	11	12	13	13
		Operability	16	17	37	18	18	15	28	19	20	20	19	31	17	16	20	18	31	18	29	20	23	33	30	33	23	19	18	26	33	16	32	19	21
		Total	42	36	70	35	49	35	43	45	45	43	36	56	43	38	43	37	53	41	56	33	41	59	58	56	49	34	35	45	61	34	57	47	47
ChatGPT-5	Confidentiality	Existence	1	0	1	1	1	1	0	1	1	1	0	1	1	1	1	1	1	1	1	1	1	1	1	1	1	1	1	1	1	1	1	1	1
		Specificity	2	1	2	2	1	2	1	2	1	1	1	1	2	2	0	1	2	2	1	2	2	2	2	0	2	2	1	2	2	1	2	2	0
		Operability	0	1	2	0	0	1	1	1	1	1	1	2	1	0	2	1	2	2	1	1	1	2	2	2	1	1	1	2	2	2	2	1	1
		Total	3	2	5	3	2	4	2	4	3	3	2	4	4	3	3	3	5	5	3	4	4	5	5	3	4	4	3	5	5	4	5	4	2
	Exceptions to confidentiality	Existence	1	1	1	1	1	1	1	1	1	1	1	1	1	1	1	0	1	1	1	1	1	1	1	1	1	1	0	1	1	1	0	1	1
		Specificity	2	2	2	0	2	2	2	2	2	1	2	2	2	2	2	0	2	2	2	1	2	1	2	1	1	2	1	2	2	1	1	1	1
		Operability	1	1	2	1	1	2	2	1	1	1	1	2	1	1	1	1	2	1	2	1	0	2	1	2	1	1	1	2	2	1	1	1	1
		Total	4	4	5	2	4	5	5	4	4	3	4	5	4	4	4	1	5	4	5	3	3	4	4	4	3	4	2	5	5	3	2	3	3
	Client rights	Existence	1	0	1	1	1	1	1	1	1	1	1	1	1	1	1	1	1	1	1	1	1	1	1	1	1	0	1	1	0	1	1	1	1
		Specificity	2	1	2	2	0	1	1	1	1	1	1	2	1	1	2	1	2	1	2	2	1	2	1	2	2	1	2	0	1	2	1	2	2
		Operability	1	0	2	0	1	1	2	2	1	1	1	2	1	2	1	1	2	2	2	1	0	2	2	2	1	1	1	2	1	0	2	1	1
		Total	4	1	5	3	2	3	4	4	3	3	3	5	3	4	4	3	5	4	5	4	2	5	4	5	4	2	4	3	2	3	4	4	4

Guardian consent	Existence	0	1	1	0	0	1	1	1	1	0	1	1	1	0	1	1	1	1	1	0	1	1	1	1	1	1	0	0	1	0	0	1	0
	Specificity	1	2	2	1	1	2	1	2	1	1	2	1	2	1	2	2	2	2	2	1	2	2	1	2	1	1	1	1	2	1	1	2	1
	Operability	1	1	2	1	1	1	2	1	1	1	1	2	2	0	1	1	1	2	2	1	1	1	1	2	1	1	1	1	2	1	1	1	1
	Total	2	4	5	2	2	4	4	4	3	2	4	4	5	1	4	4	4	5	5	2	4	4	3	5	3	3	2	2	5	2	2	4	2
Goals and scope	Existence	1	1	1	0	1	1	1	1	1	0	1	0	1	1	0	1	1	1	1	1	1	1	1	1	0	1	1	0	1	0	1	1	1
	Specificity	1	2	2	0	2	2	1	1	1	0	2	1	2	1	1	0	2	2	0	1	1	1	2	1	1	1	2	1	1	1	2	1	1
	Operability	1	1	2	1	1	1	2	1	1	1	2	1	2	1	1	1	2	2	2	1	1	0	2	2	1	1	2	1	2	1	2	1	1
	Total	3	4	5	1	4	4	4	3	3	1	5	2	5	3	2	2	5	5	3	3	3	2	5	4	2	3	5	2	4	2	5	3	3
Format and frequency	Existence	0	1	1	1	1	1	1	1	1	1	0	1	1	1	1	0	1	1	1	1	1	1	1	1	1	1	1	1	1	1	1	1	1
	Specificity	1	0	2	1	2	2	1	1	1	2	1	2	2	1	2	1	1	2	0	0	1	0	1	2	2	0	2	1	2	1	2	0	1
	Operability	1	0	2	1	1	1	2	1	1	1	1	2	1	1	1	1	2	1	2	1	1	2	2	2	2	1	1	2	2	1	2	0	0
	Total	2	1	5	3	4	4	4	3	3	4	2	5	4	3	4	2	4	4	3	2	3	3	4	5	5	2	4	4	5	3	5	1	2
Fees and cancellation policy	Existence	1	1	1	1	0	1	1	1	1	1	1	1	1	1	1	1	1	1	1	1	0	1	1	1	1	1	1	1	1	1	1	1	1
	Specificity	1	1	2	1	1	1	1	1	1	1	1	1	0	2	0	2	1	0	1	1	1	0	1	2	1	2	1	1	0	2	1	1	0
	Operability	1	1	2	1	0	1	2	1	0	1	2	2	2	0	1	1	1	1	2	1	1	2	2	2	1	1	2	2	2	1	2	2	1
	Total	3	3	5	3	1	3	4	3	2	3	4	4	3	3	2	4	3	2	4	3	2	3	4	5	3	4	4	4	3	4	4	4	2
Recording methods	Existence	1	1	1	1	1	1	1	1	1	1	0	1	1	1	1	1	0	1	1	1	1	1	1	1	1	1	1	1	1	0	1	0	1
	Specificity	1	2	2	1	1	0	1	2	2	1	1	1	1	2	0	2	1	1	0	1	1	0	2	2	2	2	0	1	2	1	1	1	1
	Operability	1	0	2	1	0	1	2	1	1	1	1	2	1	1	1	2	1	2	1	1	1	1	2	2	1	1	1	2	2	1	2	1	1
	Total	3	3	5	3	2	2	4	4	4	3	2	4	3	4	2	5	2	4	2	3	3	2	5	5	4	4	2	4	5	2	4	2	3
Authorization and revocation	Existence	1	1	1	0	1	1	1	0	1	0	1	0	1	1	0	1	1	1	0	1	1	1	1	1	1	1	1	1	1	0	1	1	1
	Specificity	1	1	2	1	1	1	0	1	1	1	2	0	1	0	0	1	1	0	1	0	1	2	0	0	1	0	0	2	1	1	0	1	1
	Operability	1	1	2	1	0	1	2	1	1	1	1	1	1	1	1	1	2	2	1	1	1	2	2	2	0	1	1	2	2	1	2	1	1
	Total	3	3	5	2	2	3	3	2	3	2	4	1	3	2	1	3	4	3	2	2	3	5	3	3	2	2	2	5	4	2	3	3	3
Crisis procedures	Existence	1	0	1	1	1	1	1	1	1	1	1	1	1	0	0	1	1	1	1	1	1	1	1	1	1	1	1	1	0	0	1	0	1
	Specificity	2	1	0	0	0	2	0	1	0	0	2	2	1	0	0	0	1	1	1	0	0	1	0	2	1	0	1	0	0	1	1	0	2
	Operability	1	1	1	1	1	1	2	1	1	1	1	2	1	1	1	1	2	2	2	1	1	2	2	2	1	1	1	2	1	1	2	1	1
	Total	4	2	2	2	2	4	3	3	2	2	4	5	3	1	1	2	4	4	4	2	2	4	3	5	3	2	3	3	1	2	4	1	4
Complaints and appeals	Existence	0	1	0	0	0	0	0	0	1	1	1	0	0	0	1	0	0	0	1	0	0	0	0	1	0	1	0	0	0	0	0	1	0
	Specificity	1	2	1	1	0	1	1	1	1	1	1	0	1	0	0	1	1	1	1	0	1	0	0	1	0	2	0	0	1	0	1	1	1
	Operability	1	1	1	1	0	1	1	1	0	1	1	1	1	0	1	1	1	1	1	1	0	1	1	1	1	1	0	1	1	1	1	1	1
	Total	2	4	2	2	0	2	2	2	2	3	3	1	2	0	2	2	2	2	3	1	1	1	1	3	1	4	0	1	2	1	2	3	2
Data	Existence	1	1	1	1	0	0	0	0	0	0	1	0	1	0	0	1	0	0	0	1	0	1	1	0	0	1	0	0	0	0	1	1	0

protection	Specificity	0	2	2	1	1	1	0	1	1	0	2	0	1	1	1	1	1	0	0	0	1	0	1	0	0	0	0	1	1	0	0	0	0
	Operability	1	1	2	1	1	1	1	1	1	1	2	1	1	1	0	1	1	1	1	2	1	2	1	1	1	1	1	1	1	1	2	1	1
	Total	2	4	5	3	2	2	1	2	2	1	5	1	3	2	1	3	2	1	1	3	2	3	3	1	1	2	1	2	2	1	3	2	1
Disclaimer of	Existence	0	0	0	0	0	0	0	0	0	0	0	0	0	0	1	0	0	0	1	0	0	1	0	0	0	0	0	0	0	0	0	0	0
boundaries	Specificity	1	1	1	0	1	1	1	1	1	0	1	1	1	1	2	1	1	1	1	1	1	2	1	1	1	1	1	1	1	1	1	1	1
	Operability	1	1	1	0	1	1	1	1	1	0	1	1	0	1	1	1	1	1	2	1	1	2	1	1	1	0	1	1	1	1	1	1	1
	Total	2	2	2	0	2	2	2	2	2	0	2	2	1	2	4	2	2	2	4	2	2	5	2	2	2	1	2	2	2	2	2	2	2
Language	Existence	0	0	0	0	1	0	0	0	0	0	0	0	0	0	1	0	0	0	0	0	0	0	1	0	0	0	0	1	0	0	0	1	1
clarity	Specificity	0	0	0	0	0	0	0	0	0	1	0	0	0	0	0	1	0	0	0	0	0	0	0	0	0	0	0	0	0	0	0	0	0
	Operability	1	0	1	1	1	0	1	1	0	0	1	1	1	1	0	1	1	1	1	0	1	1	2	1	1	1	1	2	1	1	1	1	1
	Total	1	0	1	1	2	0	1	1	0	1	1	1	1	1	1	2	1	1	1	0	1	1	3	1	1	1	1	3	1	1	1	2	2
Voluntariness	Existence	1	1	1	1	0	1	0	0	1	0	1	1	1	1	1	1	1	0	0	1	1	1	0	1	1	1	1	1	1	1	1	0	1
	Specificity	1	0	0	0	0	0	0	0	0	0	0	0	0	0	0	1	1	0	0	0	1	0	1	0	0	0	0	0	0	1	0	0	0
	Operability	1	1	2	1	1	1	1	1	0	1	1	2	1	1	1	1	2	1	1	1	1	2	1	2	1	1	1	2	2	1	2	1	1
	Total	3	2	3	2	1	2	1	1	1	1	2	3	2	2	2	3	4	1	1	2	3	3	2	3	2	2	2	3	3	3	3	1	2
Client	Existence	0	0	0	0	0	0	0	1	1	1	1	1	1	1	0	0	1	1	1	1	1	0	0	0	1	0	1	1	1	1	1	0	1
obligations	Specificity	0	0	1	0	0	0	1	1	0	0	2	1	1	1	0	0	0	0	1	0	0	0	0	0	1	0	1	0	1	0	2	0	1
	Operability	0	1	1	1	0	1	1	2	1	1	1	2	1	1	1	1	2	2	2	1	1	1	1	1	1	1	1	1	1	1	2	1	1
	Total	0	1	2	1	0	1	2	4	2	2	4	4	3	3	1	1	3	3	4	2	2	1	1	1	3	1	3	2	3	2	5	1	3
Counseling	Existence	0	1	0	0	0	1	0	1	0	0	0	0	0	0	0	0	1	1	0	0	0	0	0	0	0	0	0	0	0	0	0	0	0
limitations	Specificity	0	0	1	0	0	0	0	0	1	0	0	0	0	0	0	0	0	0	0	0	0	0	1	1	0	0	0	0	0	0	0	0	0
	Operability	1	1	1	1	1	1	1	1	0	1	1	1	1	1	1	1	2	2	1	1	1	1	1	1	1	1	1	1	1	1	1	1	1
	Total	1	2	2	1	1	2	1	2	1	1	1	1	1	1	1	1	3	3	1	1	1	1	2	2	1	1	1	1	1	1	1	1	1
Counselor	Existence	1	1	1	1	1	0	1	1	1	1	1	1	1	1	0	1	1	1	1	1	1	1	1	1	1	0	1	1	1	1	1	1	1
qualifications	Specificity	0	0	0	0	0	0	0	0	0	1	0	0	0	0	0	0	0	0	0	0	0	0	0	0	0	0	0	0	0	0	0	0	0
	Operability	1	1	2	1	1	1	2	2	0	1	1	2	1	2	1	1	2	2	2	1	1	2	1	0	1	1	1	2	2	1	2	1	1
	Total	2	2	3	2	2	1	3	3	1	3	2	3	2	3	1	2	3	3	3	2	2	3	2	1	2	1	2	3	3	2	3	2	2
Counseling	Existence	0	1	1	0	1	1	0	1	0	0	1	0	0	0	0	0	1	0	1	0	0	0	1	0	1	0	1	0	0	0	0	0	1
modalities	Specificity	1	2	2	1	2	2	1	1	1	0	2	1	1	1	0	1	1	1	0	1	1	0	0	0	2	1	1	1	1	0	0	0	0
	Operability	1	0	2	1	0	1	1	2	1	1	2	1	1	1	1	1	2	1	1	1	1	1	2	1	1	1	1	1	1	1	1	1	1
	Total	2	3	5	2	3	4	2	4	2	1	5	2	2	2	1	2	4	2	2	2	2	1	3	1	4	2	3	2	2	1	1	1	2
Target	Existence	1	0	1	1	0	0	1	1	1	1	1	1	1	1	1	1	1	1	1	0	0	1	1	1	1	0	1	1	1	1	1	1	1
population	Specificity	2	0	0	0	0	0	1	1	2	1	1	1	1	0	2	0	0	1	1	0	1	1	1	1	0	0	0	1	1	1	1	2	0

		Operability	1	1	1	1	1	1	2	1	2	1	1	2	1	1	1	1	2	2	2	1	1	2	2	2	1	1	1	2	2	2	2	2	1
		Total	4	1	2	2	1	1	4	3	5	3	3	4	3	2	4	2	3	4	4	1	2	4	4	4	2	1	2	4	4	4	4	5	2
	Total score	Existence	12	13	15	11	11	13	11	14	15	11	14	12	15	12	12	12	15	14	15	13	12	15	15	14	14	12	13	13	12	9	13	13	15
		Specificity	20	20	26	12	15	20	14	20	18	13	24	17	20	16	14	16	20	17	14	11	18	14	17	18	18	15	14	15	19	15	17	15	13
		Operability	18	15	33	17	13	20	31	24	15	18	24	32	22	18	19	21	33	31	31	20	17	31	31	31	20	19	21	32	31	21	33	21	19
		Total	50	48	74	40	39	53	56	58	48	42	62	61	57	46	45	49	68	62	60	44	47	60	63	63	52	46	48	60	62	45	63	49	47
Grok-4	Confidentiality	Existence	1	1	1	1	1	1	1	1	1	1	1	1	1	1	1	1	1	1	1	1	1	1	1	1	1	1	1	1	1	1	1	1	0
		Specificity	2	2	2	2	2	2	0	1	2	2	1	1	2	1	2	2	2	0	1	2	2	1	1	1	2	2	1	0	1	0	1	2	1
		Operability	1	1	2	0	0	1	2	1	1	1	2	2	1	2	1	1	2	0	2	1	2	2	2	2	2	2	2	2	2	1	2	1	1
		Total	4	4	5	3	3	4	3	3	4	4	4	4	4	4	4	4	5	1	4	4	5	4	4	4	5	5	4	3	4	2	4	4	2
	Exceptions to confidentiality	Existence	1	1	1	1	1	1	0	1	1	1	1	1	0	1	1	0	1	1	1	1	1	1	1	1	1	1	1	1	1	0	1	1	1
		Specificity	2	2	2	0	2	1	1	1	2	1	2	2	0	1	2	1	2	1	1	1	2	2	2	2	2	2	1	2	2	1	1	2	2
		Operability	2	1	2	0	1	1	1	1	1	1	2	2	1	0	1	1	2	1	2	1	1	2	2	2	2	1	1	1	2	1	2	1	1
		Total	5	4	5	1	4	3	2	3	4	3	5	5	1	2	4	2	5	3	4	3	4	5	5	5	5	4	3	4	5	2	4	4	4
	Client rights	Existence	1	1	1	1	0	1	1	1	1	1	1	1	1	1	1	0	1	1	1	1	1	1	1	1	1	1	1	1	1	1	1	1	1
		Specificity	2	2	2	2	1	2	2	2	2	2	2	2	2	2	2	1	2	1	2	1	2	2	2	2	2	2	2	2	2	2	2	2	2
		Operability	1	1	2	1	1	1	2	1	1	0	1	2	1	1	0	1	1	1	2	2	1	1	2	2	2	0	1	2	2	1	2	0	1
		Total	4	4	5	4	2	4	5	4	4	3	4	5	4	4	3	2	4	3	5	4	4	4	5	5	5	3	4	5	5	4	5	3	4
	Guardian consent	Existence	1	1	1	0	0	0	0	1	0	0	1	0	1	0	1	0	1	1	1	0	1	0	0	1	1	0	0	1	1	0	1	1	1
		Specificity	2	2	2	1	1	1	1	1	1	1	2	1	2	1	2	1	2	1	1	1	0	1	1	1	2	1	1	1	1	1	0	2	2
		Operability	1	1	2	1	1	1	1	2	0	0	1	1	1	1	1	1	2	1	1	1	1	1	1	1	2	1	1	2	2	0	1	0	0
		Total	4	4	5	2	2	2	2	4	1	1	4	2	4	2	4	2	5	3	3	2	2	2	2	3	5	2	2	4	4	1	2	3	3
	Goals and scope	Existence	1	1	1	0	0	1	0	1	1	1	1	1	1	0	1	1	1	1	1	1	1	1	1	1	1	1	1	1	1	1	1	1	1
		Specificity	1	1	2	1	1	1	1	0	2	1	1	2	1	1	2	2	2	1	1	2	0	1	2	2	1	1	1	2	2	2	1	0	2
		Operability	0	1	2	1	1	1	1	2	1	1	1	2	1	0	1	0	2	1	2	1	0	2	2	2	2	1	1	2	2	1	2	1	1
		Total	2	3	5	2	2	3	2	3	4	3	3	5	3	1	4	3	5	3	4	4	1	4	5	5	4	3	3	5	5	4	4	2	4
	Format and frequency	Existence	1	1	1	1	0	1	1	1	1	1	0	0	1	1	1	1	1	1	1	1	1	1	1	1	0	1	1	1	1	1	0	0	1
		Specificity	2	2	2	2	1	2	2	2	2	2	1	1	2	2	1	1	2	2	2	1	0	2	2	2	1	1	0	2	2	1	1	0	1
		Operability	1	1	2	1	1	1	2	0	1	1	1	1	1	2	1	1	2	1	2	1	1	2	2	2	1	1	1	2	2	1	1	1	1
		Total	4	4	5	4	2	4	5	3	4	4	2	2	4	5	3	3	5	4	5	3	2	5	5	5	2	3	2	5	5	3	2	1	3
	Fees and cancellation	Existence	1	1	1	1	1	1	1	1	1	1	1	1	1	1	1	1	1	1	1	1	1	1	1	1	1	1	0	1	1	0	1	1	1
		Specificity	1	1	2	1	0	1	2	0	1	1	1	1	2	1	1	0	0	1	2	1	0	1	1	1	1	1	1	2	0	1	1	1	0

policy	Operability	0	2	2	1	1	1	2	1	1	1	2	2	2	0	2	1	2	1	2	1	1	2	2	2	1	1	1	2	1	1	2	1	1
	Total	2	4	5	3	2	3	5	2	3	3	4	4	5	2	4	2	3	3	5	3	2	4	4	4	3	3	2	5	2	2	4	3	2
Recording	Existence	1	1	1	1	1	1	1	0	1	1	0	1	1	1	0	1	1	1	1	0	1	1	1	1	1	1	0	0	1	0	1	1	1
methods	Specificity	0	2	1	1	2	1	2	1	1	2	1	2	2	1	1	2	1	0	2	1	2	1	2	0	2	2	1	1	2	0	0	2	1
	Operability	1	1	2	1	1	1	2	1	0	1	0	2	1	1	1	2	1	1	2	0	1	1	1	2	2	1	1	1	1	1	2	1	1
	Total	2	4	4	3	4	3	5	2	2	4	1	5	4	3	2	5	3	2	5	1	4	3	4	3	5	4	2	2	4	1	3	4	3
Authorization	Existence	1	0	1	1	1	1	1	0	1	1	0	1	0	1	1	0	1	1	1	1	1	1	1	1	1	1	1	1	1	1	1	1	1
and revocation	Specificity	2	1	0	1	1	1	1	0	0	1	1	0	1	0	1	1	0	0	1	1	0	0	0	1	1	1	0	0	0	0	1	1	1
	Operability	1	1	2	1	1	1	2	1	0	1	1	2	1	1	1	1	1	1	2	1	0	2	1	2	2	1	1	2	2	1	2	1	1
	Total	4	2	3	3	3	3	4	1	1	3	2	3	2	2	3	2	2	2	4	3	1	3	2	4	4	3	2	3	3	2	4	3	3
Crisis	Existence	1	1	1	0	0	1	1	1	1	1	1	1	0	1	1	1	0	1	1	1	1	1	1	0	1	0	1	1	1	1	1	1	1
procedures	Specificity	0	0	1	1	1	1	0	1	0	1	1	1	1	0	1	0	1	2	1	0	0	1	1	1	1	1	2	1	2	1	2	1	2
	Operability	1	1	2	0	1	0	2	0	0	1	0	2	1	1	1	1	1	1	2	0	0	2	2	1	2	1	1	2	2	1	2	0	1
	Total	2	2	4	1	2	2	3	2	1	3	2	4	2	2	3	2	2	4	4	1	1	4	4	2	4	2	4	4	5	3	5	2	4
Complaints	Existence	1	1	1	0	0	1	0	0	0	0	0	1	0	0	1	0	1	0	0	1	1	0	0	1	0	1	1	0	0	0	1	0	0
and appeals	Specificity	2	1	1	1	1	1	1	0	1	0	1	1	1	0	1	0	2	0	1	1	1	1	0	1	1	1	0	1	1	1	0	1	1
	Operability	1	1	1	1	1	1	1	1	0	1	1	2	1	1	1	1	2	1	1	1	1	1	1	2	1	1	1	1	1	1	2	1	0
	Total	4	3	3	2	2	3	2	1	1	1	2	4	2	1	3	1	5	1	2	3	3	2	1	4	2	3	2	2	2	2	3	2	1
Data	Existence	0	1	0	1	1	1	1	0	1	0	0	1	0	1	1	0	1	1	0	1	1	0	1	0	1	1	1	0	1	0	1	1	0
protection	Specificity	0	1	0	0	1	2	1	1	0	1	0	2	1	2	0	1	0	0	0	2	1	0	1	0	1	1	2	0	1	0	0	1	1
	Operability	1	1	1	1	1	0	1	1	1	1	1	2	1	0	2	1	2	1	1	2	1	1	2	1	1	1	1	1	2	1	2	1	1
	Total	1	3	1	2	3	3	3	2	2	2	1	5	2	3	3	2	3	2	1	5	3	1	4	1	3	3	4	1	4	1	3	3	2
Disclaimer of	Existence	0	0	0	0	0	0	0	0	0	1	0	1	0	0	0	0	0	0	0	0	1	1	1	0	0	1	1	0	0	0	0	0	0
boundaries	Specificity	1	1	1	1	1	1	1	1	1	2	1	2	1	1	1	1	1	1	1	1	2	2	2	1	1	2	2	1	1	1	1	1	1
	Operability	1	1	1	1	1	1	1	1	1	0	0	2	1	1	1	1	1	1	1	1	1	2	2	1	1	1	2	1	1	1	1	1	1
	Total	2	2	2	2	2	2	2	2	2	3	1	5	2	2	2	2	2	2	2	2	4	5	5	2	2	4	5	2	2	2	2	2	2
Language	Existence	1	0	0	0	0	0	1	1	1	0	0	0	0	0	0	0	0	0	1	0	0	0	1	0	0	1	0	1	0	0	0	0	1
clarity	Specificity	0	1	0	0	0	1	1	0	0	0	0	1	0	0	0	1	0	1	0	0	0	0	0	0	1	0	1	0	0	1	0	0	1
	Operability	1	1	1	0	1	0	2	1	1	1	1	1	1	1	1	1	1	1	1	1	1	1	1	1	1	1	1	2	1	1	1	1	1
	Total	2	2	1	0	1	1	4	2	2	1	1	2	1	1	1	2	1	2	2	1	1	1	2	1	2	2	2	3	1	2	1	1	3
Voluntariness	Existence	1	0	1	0	0	0	1	0	0	1	1	1	1	1	0	1	0	0	1	0	1	1	0	1	1	1	0	1	0	1	1	1	0
	Specificity	0	0	2	0	1	0	0	0	0	0	0	0	0	0	0	1	0	0	0	0	0	0	0	0	0	0	0	0	0	0	0	0	0

	Operability	1	1	2	1	1	1	2	0	1	1	1	2	1	0	0	1	1	1	2	1	1	2	1	2	2	1	1	1	1	1	2	1	1
	Total	2	1	5	1	2	1	3	0	1	2	2	3	2	1	0	3	1	1	3	1	2	3	1	3	3	2	1	2	1	2	3	2	1
Client obligations	Existence	0	1	1	1	1	1	1	0	1	1	1	1	1	1	1	1	0	1	1	0	0	0	0	1	1	1	1	0	1	1	1	1	1
	Specificity	0	1	1	2	1	0	0	1	1	0	0	2	1	1	2	0	1	1	2	0	0	0	0	0	0	0	0	0	1	0	0	0	1
	Operability	0	1	2	1	1	0	2	1	1	1	1	2	1	0	1	1	1	1	2	0	1	1	1	2	2	2	1	1	2	1	2	1	1
	Total	0	3	4	4	3	1	3	2	3	2	2	5	3	2	4	2	2	3	5	0	1	1	1	3	3	3	2	1	4	2	3	2	3
Counseling limitations	Existence	0	1	0	0	0	1	1	0	1	0	1	1	1	0	1	0	0	1	0	1	0	0	0	0	0	0	0	0	0	0	0	0	0
	Specificity	0	0	0	0	1	0	1	0	1	0	0	0	0	1	0	0	0	0	0	0	1	0	1	0	0	0	0	0	0	0	0	1	0
	Operability	1	1	1	0	1	1	2	1	1	1	1	2	1	1	1	1	1	1	1	1	1	1	1	1	1	1	1	1	1	1	1	1	1
	Total	1	2	1	0	2	2	4	1	3	1	2	3	2	2	2	1	1	2	1	2	2	1	2	1	1	1	1	1	1	1	1	2	1
Counselor qualifications	Existence	1	1	1	1	1	1	1	1	0	1	1	0	1	1	1	1	1	1	1	1	0	1	1	1	1	0	1	1	1	1	1	1	1
	Specificity	0	0	0	1	0	0	0	0	1	0	1	0	0	0	0	0	0	0	0	0	0	0	0	0	0	0	0	0	0	0	0	0	0
	Operability	1	1	1	1	2	1	2	1	1	2	2	1	1	1	1	1	2	0	2	1	1	2	2	2	1	1	1	2	2	2	2	1	1
	Total	2	2	2	3	3	2	3	2	2	3	4	1	2	2	2	2	3	1	3	2	1	3	3	3	2	1	2	3	3	3	3	2	2
Counseling modalities	Existence	0	1	1	0	1	0	0	1	0	1	0	0	0	0	0	0	0	0	0	0	1	0	0	0	1	0	1	0	0	0	0	0	0
	Specificity	1	1	2	1	1	1	1	2	1	2	1	1	1	1	1	1	1	1	1	1	2	0	1	1	1	1	2	1	1	1	1	1	1
	Operability	1	1	1	1	2	1	1	2	1	1	1	1	1	1	1	1	1	1	1	1	1	1	1	1	2	1	1	1	1	1	1	1	1
	Total	2	3	4	2	4	2	2	5	2	4	2	2	2	2	2	2	2	2	2	2	4	1	2	2	4	2	4	2	2	2	2	2	2
Target population	Existence	1	1	1	1	1	1	0	1	1	1	1	1	1	1	1	1	1	1	1	1	0	1	1	1	1	0	1	0	1	1	1	1	1
	Specificity	2	2	0	1	1	0	0	0	2	2	1	1	0	0	1	1	0	0	1	1	0	0	2	1	1	0	0	1	0	0	0	0	1
	Operability	0	1	2	1	2	1	1	1	1	1	1	2	1	1	1	2	1	1	2	0	0	2	2	2	1	1	1	1	1	0	2	1	1
	Total	3	4	3	3	4	2	1	2	4	4	3	4	2	2	3	4	2	2	4	2	0	3	5	4	3	1	2	2	2	1	3	2	3
Total score	Existence	15	16	16	11	10	15	13	12	14	15	12	15	12	13	15	10	13	15	15	13	15	13	14	14	15	14	14	12	14	10	15	14	13
	Specificity	20	23	23	19	20	19	18	14	21	21	18	23	20	16	21	17	19	13	20	17	15	15	21	17	21	19	17	17	19	13	12	18	21
	Operability	17	21	33	15	22	16	32	20	15	18	21	35	21	16	20	21	29	18	33	18	17	31	31	33	31	21	22	30	31	19	34	17	18
	Total	52	60	72	45	52	50	63	46	50	54	51	73	53	45	56	48	61	46	68	48	47	59	66	64	67	54	53	59	64	42	61	49	52

Supplementary Data S5. Table of detailed ratings by expert 05 for all documents

Version	Indicator	Dimension	Document ID																																
			01	02	03	04	05	06	07	08	09	10	11	12	13	14	15	16	17	18	19	20	21	22	23	24	25	26	27	28	29	30	31	32	33
Original	Confidentiality	Existence	1	1	1	1	0	1	1	1	1	1	1	1	1	1	1	1	1	1	1	1	1	0	1	1	1	0	1	1	1	1	1	1	1
		Specificity	1	0	1	1	1	1	0	0	2	1	1	0	0	1	0	1	0	0	0	1	0	1	1	0	1	1	0	1	0	0	1	1	1
		Operability	1	1	2	1	1	1	2	1	1	1	1	2	2	1	1	1	2	1	1	1	1	1	1	2	1	1	1	2	2	1	2	1	1
		Total	3	2	4	3	2	3	3	2	4	3	3	3	3	3	2	3	3	2	2	3	2	2	3	3	3	2	2	4	3	2	4	3	3
	Exceptions to confidentiality	Existence	1	0	1	0	1	1	0	1	1	0	0	1	1	1	1	0	1	1	1	0	1	1	1	0	1	0	1	0	1	1	1	1	1
		Specificity	2	1	2	0	2	1	0	2	1	0	0	1	0	1	1	1	1	1	2	0	1	1	1	0	1	1	0	1	1	0	2	1	1
		Operability	1	1	1	1	1	1	1	1	2	1	1	2	0	1	1	1	2	1	1	1	1	2	2	1	1	1	1	1	2	1	2	0	1
		Total	4	2	4	1	4	3	1	4	4	1	1	4	1	3	3	2	4	3	4	1	3	4	4	1	3	2	2	2	4	2	5	2	3
	Client rights	Existence	1	1	1	1	0	1	1	1	1	1	1	1	1	0	1	1	0	1	1	1	1	1	1	1	1	1	1	1	1	1	1	1	1
		Specificity	0	1	1	2	0	1	1	1	1	0	0	0	1	0	1	1	1	1	1	1	1	2	1	2	1	0	1	0	2	2	1	2	1
		Operability	1	1	2	1	1	2	1	1	1	1	1	1	1	1	1	1	1	1	1	1	1	2	1	2	1	1	1	2	2	1	2	1	2
		Total	2	3	4	4	1	4	3	3	3	2	2	2	3	1	3	3	2	3	3	3	3	5	3	5	3	2	3	3	5	4	4	4	4
	Guardian consent	Existence	1	1	1	0	1	1	0	1	1	0	0	0	1	0	0	0	1	1	1	0	1	1	0	1	0	0	0	0	1	0	1	1	1
		Specificity	2	0	2	1	2	1	0	1	2	1	1	1	1	1	1	1	0	1	1	1	1	2	1	1	1	0	0	1	1	0	1	2	0
		Operability	1	1	2	1	1	2	1	2	1	1	1	1	0	1	0	1	2	1	2	0	1	2	1	2	1	1	1	1	1	0	2	1	2
		Total	4	2	5	2	4	4	1	4	4	2	2	2	2	2	1	2	3	3	4	1	3	5	2	4	2	1	1	2	3	0	4	4	3
	Goals and scope	Existence	1	0	1	1	1	0	0	1	0	0	0	1	1	0	1	0	1	0	1	0	1	1	1	1	0	0	0	1	1	0	1	1	1
		Specificity	1	1	1	0	2	0	0	1	1	0	0	1	1	1	1	0	1	1	1	0	0	0	2	2	0	0	0	2	1	0	1	0	1
		Operability	2	1	2	1	1	1	1	1	1	1	1	2	1	1	1	1	2	1	2	1	1	1	2	2	1	1	1	2	2	1	2	1	2
		Total	4	2	4	2	4	1	1	3	2	1	1	4	3	2	3	1	4	2	4	1	2	2	5	5	1	1	1	5	4	1	4	2	4
	Format and frequency	Existence	1	0	1	1	1	1	1	1	1	1	1	1	0	0	1	0	1	1	0	0	0	1	1	0	1	1	1	1	1	1	1	0	1
		Specificity	2	0	2	1	1	2	1	2	1	2	1	2	1	1	2	1	2	2	1	1	0	0	2	1	2	1	1	1	2	0	2	0	2
		Operability	1	1	2	1	1	1	2	1	2	1	1	2	1	1	1	1	2	1	1	1	1	2	2	1	1	2	1	2	1	1	2	1	1
		Total	4	1	5	3	3	4	4	4	4	4	3	5	2	2	4	2	5	4	2	2	1	3	5	2	4	4	3	4	4	2	5	1	4
	Fees and cancellation policy	Existence	1	1	1	1	1	1	1	1	1	1	1	1	1	1	0	1	0	1	1	0	0	1	1	1	1	0	1	0	1	0	1	1	1
		Specificity	1	1	2	0	0	1	2	1	0	1	1	1	1	1	1	1	0	1	1	0	0	0	0	1	1	1	0	1	1	1	1	1	1
		Operability	1	1	2	2	1	1	2	1	0	1	1	2	1	1	1	1	1	1	2	1	1	2	2	2	1	1	2	1	2	1	2	1	0
		Total	3	3	5	3	2	3	5	3	1	3	3	4	3	3	2	3	1	3	4	1	1	3	3	4	3	2	3	2	4	2	4	3	2
	Recording methods	Existence	1	0	1	1	1	0	1	1	1	1	0	1	1	1	0	1	1	1	1	1	0	1	1	1	1	1	0	1	1	0	1	1	1
		Specificity	0	1	1	0	2	0	1	2	1	1	0	1	2	2	1	2	1	1	1	0	1	1	2	1	1	1	0	2	2	0	1	1	1

	Operability	1	1	2	1	1	1	2	1	0	1	1	2	1	1	1	1	2	0	2	1	1	2	2	2	1	1	1	2	2	0	2	1	1
	Total	2	2	4	2	4	1	4	4	2	3	1	4	4	4	2	4	4	2	4	2	2	4	5	4	3	3	1	5	5	0	4	3	3
Authorization and revocation	Existence	1	1	1	1	1	0	1	1	1	1	0	1	1	1	0	1	1	1	0	1	0	0	1	1	1	1	1	1	1	0	1	1	1
	Specificity	1	0	0	0	1	0	0	0	1	1	1	0	1	0	0	2	0	0	1	0	1	1	1	0	0	0	0	0	0	0	0	2	0
	Operability	1	0	2	1	1	1	2	1	1	1	0	2	1	1	1	1	1	1	1	1	1	1	1	2	1	1	1	2	2	1	2	1	1
	Total	3	1	3	2	3	1	3	2	3	3	1	3	3	2	1	4	2	2	2	2	2	2	3	3	2	2	2	3	3	1	3	4	2
Crisis procedures	Existence	1	1	1	1	1	1	1	1	0	1	1	1	1	1	0	1	1	1	1	0	1	1	1	1	1	0	1	1	1	1	1	1	0
	Specificity	0	0	1	1	1	1	0	1	0	0	0	1	1	0	1	0	1	1	0	0	0	0	1	2	1	0	0	0	1	1	0	0	0
	Operability	1	1	2	1	1	1	2	1	0	1	1	2	1	1	1	1	2	1	2	1	1	2	1	1	1	1	1	1	2	1	2	0	0
	Total	2	2	4	3	3	3	3	3	0	2	2	4	3	2	2	2	4	3	3	1	2	3	3	4	3	1	2	2	4	3	3	1	0
Complaints and appeals	Existence	0	0	1	0	0	1	0	0	0	0	0	0	0	0	0	0	0	0	0	0	0	0	0	0	0	1	0	0	0	0	0	0	1
	Specificity	1	1	0	0	0	0	0	0	1	1	1	0	0	0	0	1	0	0	0	1	0	1	0	0	0	0	0	0	0	1	0	1	0
	Operability	1	1	2	1	1	1	1	1	0	1	0	1	1	1	0	1	1	1	1	1	1	1	1	1	1	1	0	1	1	0	1	1	1
	Total	2	2	3	1	1	2	1	1	1	2	1	1	1	1	0	2	1	1	1	2	1	2	1	1	1	2	0	1	1	1	1	2	2
Data protection	Existence	1	0	0	1	0	0	1	0	0	0	0	0	0	1	0	0	0	0	0	1	1	0	1	0	0	0	1	0	0	0	1	1	0
	Specificity	1	0	1	0	1	1	1	1	0	0	0	1	1	1	0	1	1	0	0	1	0	0	1	0	0	0	0	0	1	0	0	0	0
	Operability	1	0	1	0	1	0	2	1	1	1	1	1	1	1	1	1	1	1	1	1	1	1	1	1	1	1	1	1	1	1	2	1	1
	Total	3	0	2	1	2	1	4	2	1	1	1	2	2	3	1	2	2	1	1	3	2	1	3	1	1	1	2	1	2	1	3	2	1
Disclaimer of boundaries	Existence	1	0	0	0	0	0	0	0	0	0	0	0	0	0	0	0	0	0	0	0	0	0	0	0	0	0	0	0	0	0	0	0	0
	Specificity	2	1	1	0	1	1	1	1	1	0	0	1	1	1	1	1	1	1	1	1	1	1	1	1	1	1	1	1	1	0	1	1	1
	Operability	2	0	1	1	0	0	1	1	1	1	1	1	1	1	1	1	1	0	1	1	1	1	1	1	1	1	1	1	1	1	0	0	1
	Total	5	1	2	1	1	1	2	2	2	1	1	2	2	2	2	2	2	1	2	2	2	2	2	2	2	2	2	2	2	1	1	1	2
Language clarity	Existence	0	0	0	0	0	0	0	0	0	0	0	0	0	0	0	0	0	1	0	0	1	0	0	0	0	0	0	0	1	0	0	0	0
	Specificity	1	0	1	0	1	0	0	0	1	0	0	0	0	0	0	0	0	0	0	0	0	1	0	1	0	0	0	0	0	0	0	0	0
	Operability	1	1	1	1	1	1	1	1	1	1	1	1	1	1	1	1	1	1	1	1	1	1	1	1	0	1	0	1	2	1	1	1	1
	Total	2	1	2	1	2	1	1	1	2	1	1	1	1	1	1	1	1	2	1	1	2	2	1	2	0	1	0	1	3	1	1	1	1
Voluntariness	Existence	0	0	0	0	0	0	0	0	1	1	1	0	1	0	0	1	0	0	0	1	1	1	0	1	1	1	0	0	0	1	1	0	1
	Specificity	0	0	0	1	0	1	0	0	0	0	0	1	1	0	0	1	0	0	0	0	0	0	0	0	0	0	0	0	0	0	0	0	0
	Operability	0	1	1	1	1	1	1	1	1	1	1	1	2	1	1	1	1	1	1	2	1	1	1	2	1	1	1	1	1	1	2	1	0
	Total	0	1	1	2	1	2	1	1	2	2	2	2	4	1	1	3	1	1	1	3	2	2	1	3	2	2	1	1	1	2	3	1	1
Client obligations	Existence	0	1	0	0	1	0	0	1	1	0	0	1	1	0	1	0	1	1	0	0	0	1	0	1	1	0	0	0	1	0	0	1	0
	Specificity	0	0	0	0	1	0	0	0	0	0	0	1	2	0	0	0	0	0	0	0	1	0	0	0	0	1	0	0	0	1	0	1	0
	Operability	1	1	1	1	1	1	1	1	1	0	1	2	1	1	1	0	2	1	1	1	1	2	1	2	0	1	1	1	2	0	1	1	1

		Total	1	2	1	1	3	1	1	2	2	0	1	4	4	1	2	0	3	2	1	1	2	3	1	3	1	2	1	1	3	1	1	3	1
	Counseling limitations	Existence	1	0	0	0	0	0	0	0	0	0	0	0	0	1	0	0	0	0	0	0	0	0	1	0	0	0	0	0	0	0	0	0	0
		Specificity	0	0	0	0	0	1	0	0	0	0	0	1	0	0	1	0	0	0	0	0	0	0	0	1	0	0	1	0	0	0	0	0	0
		Operability	1	1	1	1	1	1	1	1	1	0	1	1	1	1	1	1	1	1	1	1	1	1	2	1	1	1	1	1	1	1	1	1	0
		Total	2	1	1	1	1	2	1	1	1	0	1	2	1	2	2	1	1	1	1	1	1	1	3	2	1	1	2	1	1	1	1	1	0
	Counselor qualifications	Existence	0	1	0	1	1	0	1	1	1	1	1	1	1	1	0	1	1	1	1	1	0	1	0	1	0	1	1	1	1	1	1	1	1
		Specificity	0	0	0	0	0	0	0	0	0	0	0	0	0	0	0	0	0	0	0	0	0	0	1	0	0	0	0	0	0	0	0	0	0
		Operability	1	0	1	1	2	1	2	0	0	1	2	1	1	1	1	1	2	0	2	1	1	2	1	2	1	1	1	2	2	1	2	1	0
		Total	1	1	1	2	3	1	3	1	1	2	3	2	2	2	1	2	3	1	3	2	1	3	2	3	1	2	2	3	3	2	3	2	1
	Counseling modalities	Existence	1	1	1	0	1	0	0	0	0	0	0	0	1	1	0	0	0	0	0	0	0	0	1	0	0	0	1	0	0	0	0	0	1
		Specificity	0	1	1	1	0	1	0	0	0	0	0	0	1	0	0	1	0	0	0	0	1	0	0	0	0	1	0	0	0	0	0	0	1
		Operability	1	1	2	1	0	1	1	1	1	1	1	1	1	2	1	1	1	1	1	1	1	1	2	1	1	0	2	1	1	1	1	1	1
		Total	2	3	4	2	1	2	1	1	1	1	1	1	3	3	1	2	1	1	1	1	2	1	3	1	1	1	3	1	1	1	1	1	3
	Target population	Existence	1	0	0	0	0	0	1	1	1	1	1	1	1	1	1	1	1	1	1	0	1	1	1	1	0	0	1	1	1	1	1	1	1
		Specificity	2	0	0	0	1	0	0	0	2	1	1	0	1	0	2	2	1	2	2	0	0	0	1	0	1	0	0	0	1	0	1	1	0
		Operability	0	1	1	1	1	1	2	1	1	2	1	2	1	1	1	1	2	1	2	1	1	2	1	2	1	1	1	2	2	2	2	1	1
		Total	3	1	1	1	2	1	3	2	4	4	3	3	3	2	4	4	4	4	5	1	2	3	3	3	2	1	2	3	4	3	4	3	2
	Total score	Existence	15	9	12	10	11	8	10	13	12	10	8	12	14	11	7	9	11	13	10	7	10	12	13	12	10	7	11	9	14	8	14	13	14
		Specificity	17	8	17	8	17	13	7	13	15	9	7	13	16	10	13	17	10	12	12	7	8	11	16	13	11	8	4	10	14	6	12	14	10
		Operability	20	16	31	20	19	20	29	20	17	19	19	30	20	21	18	19	30	17	27	20	20	30	27	31	18	20	20	28	32	17	33	17	18
		Total	52	33	60	38	47	41	46	46	44	38	34	55	50	42	38	45	51	42	49	34	38	53	56	56	39	35	35	47	60	31	59	44	42
ChatGPT-5	Confidentiality	Existence	1	1	1	1	1	1	0	1	1	0	1	1	1	1	1	1	1	1	1	0	0	1	1	1	1	1	1	1	1	1	1	1	1
		Specificity	2	2	2	2	1	1	1	2	2	1	2	0	2	2	1	1	2	2	1	1	1	1	2	1	2	2	1	2	2	1	2	2	1
		Operability	1	1	2	2	1	0	1	1	1	1	1	2	2	1	1	0	2	2	2	1	1	2	1	2	1	1	1	2	2	1	2	1	0
		Total	4	4	5	5	3	2	2	4	4	2	4	3	5	4	3	2	5	5	4	2	2	4	4	4	4	4	3	5	5	3	5	4	2
	Exceptions to confidentiality	Existence	1	1	0	1	1	1	1	1	1	1	1	1	1	0	0	1	1	1	1	1	1	1	1	1	1	1	1	1	1	1	1	1	1
		Specificity	2	2	1	1	2	2	2	2	2	1	1	2	2	1	1	1	2	2	2	1	2	2	2	1	1	2	1	1	2	1	2	2	2
		Operability	1	1	1	2	1	1	2	0	1	1	1	2	1	1	1	1	2	1	2	1	1	2	2	2	1	0	1	2	2	1	2	1	1
		Total	4	4	2	4	4	4	5	3	4	3	3	5	4	2	2	3	5	4	5	3	4	5	5	4	3	3	3	4	5	3	5	4	4
	Client rights	Existence	1	1	0	1	1	1	1	1	1	1	0	1	1	1	1	1	1	0	1	1	1	1	1	1	1	1	1	0	1	1	1	1	1
		Specificity	2	1	1	2	0	1	1	1	1	0	1	1	2	1	2	1	1	1	2	1	2	1	0	2	2	2	2	0	1	2	0	2	2
		Operability	1	1	1	2	1	1	2	1	1	1	1	2	1	1	1	1	1	1	2	1	1	2	2	2	1	1	1	1	2	1	2	1	1
		Total	4	3	2	5	2	3	4	3	3	2	2	4	4	3	4	3	3	2	5	3	4	4	3	5	4	4	4	1	4	4	3	4	4

Guardian consent	Existence	1	1	1	0	1	1	1	1	1	0	1	0	1	0	1	1	1	0	1	1	1	0	1	1	1	1	1	0	1	0	1	1	1
	Specificity	2	1	2	1	1	2	1	2	2	1	2	1	1	1	1	2	2	1	2	1	2	1	1	2	2	1	1	1	2	1	2	2	2
	Operability	0	1	2	1	1	1	2	0	1	1	1	1	2	1	2	2	2	1	2	1	2	1	2	2	1	1	1	1	2	1	2	1	1
	Total	3	3	5	2	3	4	4	3	4	2	4	2	4	2	4	5	5	2	5	3	5	2	4	5	4	3	3	2	5	2	5	4	4
Goals and scope	Existence	1	1	1	0	1	1	0	1	1	0	1	0	1	1	1	1	1	1	1	1	1	1	1	1	0	1	1	1	1	0	1	1	1
	Specificity	1	2	2	1	2	2	0	1	1	0	2	1	2	2	1	1	2	1	1	1	1	2	2	0	1	1	1	1	1	0	2	0	1
	Operability	2	2	1	1	2	0	1	1	1	1	0	1	1	1	2	1	2	2	1	1	1	2	1	2	0	1	1	2	2	1	2	1	1
	Total	4	5	4	2	5	3	1	3	3	1	3	2	4	4	4	3	5	4	3	3	3	5	4	3	1	3	3	4	4	1	5	2	3
Format and frequency	Existence	1	1	1	1	0	1	1	1	1	1	1	1	1	1	1	1	1	1	1	1	0	0	1	1	1	1	1	1	1	1	1	1	1
	Specificity	1	0	2	2	1	1	1	1	1	2	1	2	2	1	2	1	1	2	1	0	1	0	0	0	2	0	2	1	1	1	2	0	1
	Operability	0	0	2	0	0	1	2	1	2	2	1	2	1	1	1	1	1	2	2	1	1	1	2	2	1	1	2	2	2	1	2	2	0
	Total	2	1	5	3	1	3	4	3	4	5	3	5	4	3	4	3	3	5	4	2	2	1	3	3	4	2	5	4	4	3	5	3	2
Fees and cancellation policy	Existence	0	1	0	1	1	1	1	1	1	1	1	1	0	1	1	1	1	0	1	1	0	1	1	1	0	1	1	1	0	1	1	1	1
	Specificity	1	1	0	1	1	1	1	0	1	1	1	1	1	1	1	0	1	1	1	0	1	1	1	1	1	1	1	1	1	1	1	1	1
	Operability	1	1	1	2	1	1	2	1	1	1	0	2	0	1	1	1	2	1	2	0	1	2	2	2	0	2	2	1	1	1	2	0	1
	Total	2	3	1	4	3	3	4	2	3	3	2	4	1	3	3	2	4	2	4	1	2	4	4	4	1	4	4	3	2	3	4	2	3
Recording methods	Existence	1	1	1	1	1	1	1	1	0	1	1	1	0	1	1	1	1	1	1	1	1	1	1	1	1	1	0	1	1	0	1	1	1
	Specificity	1	2	2	1	2	1	1	2	1	2	2	1	1	2	1	2	1	1	1	1	2	1	2	2	1	1	1	1	2	0	1	2	2
	Operability	1	1	1	2	1	1	2	1	1	1	0	2	1	1	1	1	1	2	2	1	2	2	1	2	1	1	1	2	2	1	2	1	2
	Total	3	4	4	4	4	3	4	4	2	4	3	4	2	4	3	4	3	4	4	3	5	4	4	5	3	3	2	4	5	1	4	4	5
Authorization and revocation	Existence	1	1	1	1	1	1	1	1	1	1	1	1	1	1	0	1	0	1	1	1	1	1	0	1	1	1	1	1	1	1	1	1	1
	Specificity	1	1	2	2	1	0	0	0	1	2	1	0	1	1	0	1	1	1	1	0	2	1	0	1	0	0	0	1	1	0	1	0	1
	Operability	1	1	2	1	1	1	2	1	1	1	1	2	0	1	1	1	1	2	2	1	1	2	1	1	0	1	1	2	2	1	2	1	1
	Total	3	3	5	4	3	2	3	2	3	4	3	3	2	3	1	3	2	4	4	2	4	4	1	3	1	2	2	4	4	2	4	2	3
Crisis procedures	Existence	1	1	1	1	1	0	1	1	1	1	1	1	1	1	1	1	1	1	1	0	1	1	1	1	1	1	1	1	1	1	1	1	1
	Specificity	2	2	2	0	0	1	0	0	0	0	2	2	2	0	1	0	1	2	0	0	0	0	0	2	1	1	0	0	1	0	2	0	0
	Operability	1	1	2	0	1	1	2	1	1	1	1	2	1	1	1	1	2	2	2	0	1	2	2	2	1	1	1	2	2	1	2	1	1
	Total	4	4	5	1	2	2	3	2	2	2	4	5	4	2	3	2	4	5	3	0	2	3	3	5	3	3	2	3	4	2	5	2	2
Complaints and appeals	Existence	0	1	1	0	1	0	0	0	1	0	1	0	0	1	1	0	0	0	1	0	0	0	0	0	0	1	0	0	0	0	0	1	0
	Specificity	1	2	1	1	1	1	1	1	2	1	0	1	1	1	1	1	0	1	2	0	1	1	0	1	0	2	0	1	1	0	1	0	1
	Operability	1	0	2	1	1	1	1	1	0	1	1	1	1	1	1	1	1	1	2	1	1	1	1	1	1	1	1	1	1	1	1	1	1
	Total	2	3	4	2	3	2	2	2	3	2	2	2	2	3	3	2	1	2	5	1	2	2	1	2	1	4	1	2	2	1	2	2	2
Data	Existence	0	1	1	1	0	0	0	0	0	0	1	0	1	0	0	0	1	1	0	1	0	0	1	0	1	1	0	0	0	0	1	0	0

protection	Specificity	1	2	2	1	1	0	0	0	1	0	1	0	1	1	1	1	0	0	1	0	1	0	1	0	0	0	1	0	1	0	0	0	0
	Operability	1	1	2	1	0	1	1	1	1	1	1	1	1	1	1	1	2	2	1	1	1	1	1	1	0	1	1	1	1	1	2	0	1
	Total	2	4	5	3	1	1	1	1	2	1	3	1	3	2	2	2	3	3	2	2	2	1	3	1	1	2	2	1	2	1	3	0	1
Disclaimer of	Existence	0	0	0	0	1	0	0	0	0	0	0	0	0	0	0	0	1	1	0	0	0	0	0	0	0	0	0	0	0	0	0	0	0
boundaries	Specificity	1	1	1	0	2	1	1	1	1	1	1	1	1	1	1	1	2	2	1	1	1	1	1	1	1	1	1	1	1	1	1	1	1
	Operability	1	1	1	1	1	1	1	1	1	1	1	1	1	1	1	0	2	1	1	1	1	1	1	1	0	1	1	1	1	1	1	1	1
	Total	2	2	2	1	4	2	2	2	2	2	2	2	2	2	2	1	5	4	2	2	2	2	2	2	1	2	2	2	2	2	2	2	2
Language	Existence	1	0	0	0	0	0	1	0	0	0	0	0	0	1	0	0	0	0	0	0	0	1	0	0	0	0	0	0	0	0	0	1	1
clarity	Specificity	0	0	0	0	0	0	0	0	0	0	1	0	0	0	1	0	0	0	0	0	0	0	0	0	0	0	0	0	0	0	0	0	0
	Operability	1	1	1	1	1	1	2	1	1	1	1	1	0	1	1	1	1	1	1	1	1	2	1	1	0	1	1	1	1	1	1	1	1
	Total	2	1	1	1	1	1	3	1	1	1	2	1	0	2	2	1	1	1	1	1	1	3	1	1	0	1	1	1	1	1	1	2	2
Voluntariness	Existence	1	0	1	1	0	1	0	0	0	1	1	1	1	1	1	1	1	0	1	1	1	0	0	1	1	1	0	1	1	1	0	1	1
	Specificity	1	1	0	0	0	0	0	0	0	0	1	0	0	0	0	1	0	0	0	0	0	0	0	0	0	1	0	0	0	0	0	0	0
	Operability	1	1	1	1	1	1	1	0	1	1	1	2	1	2	1	2	2	1	2	1	1	1	1	2	1	2	1	2	2	1	1	1	0
	Total	3	2	2	2	1	2	1	0	1	2	3	3	2	3	2	4	3	1	3	2	2	1	1	3	2	4	1	3	3	2	1	2	1
Client	Existence	0	0	0	1	0	0	0	1	1	1	1	1	1	1	0	0	1	1	1	1	1	0	0	1	1	0	1	1	1	1	1	0	1
obligations	Specificity	0	0	1	0	0	1	0	2	0	0	0	1	2	1	0	1	0	0	0	0	0	0	0	0	1	0	1	0	1	0	1	0	1
	Operability	1	1	1	1	1	1	1	0	1	2	1	2	2	1	1	0	2	2	2	0	1	1	1	2	0	1	1	2	2	2	1	1	2
	Total	1	1	2	2	1	2	1	3	2	3	2	4	5	3	1	1	3	3	3	1	2	1	1	3	2	1	3	3	4	3	3	1	4
Counseling	Existence	0	1	0	0	1	0	0	0	0	0	0	0	0	0	1	0	1	1	0	0	0	0	0	0	0	0	0	0	0	0	0	1	0
limitations	Specificity	0	0	1	0	0	0	0	0	1	0	0	1	0	0	0	0	0	0	0	0	0	0	0	0	0	0	0	0	0	0	0	0	0
	Operability	1	1	1	1	2	0	1	1	1	1	1	1	1	1	2	0	2	2	1	0	1	1	1	1	1	1	1	1	1	1	1	1	1
	Total	1	2	2	1	3	0	1	1	2	1	1	2	1	1	3	0	3	3	1	0	1	1	1	1	1	1	1	1	1	1	1	2	1
Counselor	Existence	1	1	1	0	1	1	1	1	1	0	1	1	1	1	0	1	1	1	1	1	1	1	0	1	1	1	1	1	1	1	1	1	1
qualifications	Specificity	0	0	0	0	0	0	0	0	0	0	0	0	0	0	0	0	0	0	0	0	0	0	0	0	0	0	0	0	0	0	0	0	0
	Operability	1	1	2	0	0	1	2	1	1	1	1	2	1	0	1	1	2	2	2	1	1	2	1	2	1	1	1	1	1	1	2	1	1
	Total	2	2	3	0	1	2	3	2	2	1	2	3	2	1	1	2	3	3	3	2	2	3	1	3	2	2	2	2	2	2	3	2	2
Counseling	Existence	0	1	1	0	1	0	0	0	0	0	1	0	1	0	0	0	0	0	0	0	1	0	0	0	1	0	1	0	0	0	0	0	0
modalities	Specificity	1	2	2	1	1	1	1	0	1	0	2	0	2	1	0	1	1	0	1	1	2	0	0	0	2	1	1	1	1	0	0	0	0
	Operability	1	0	2	1	1	1	1	0	1	1	1	1	1	1	1	1	1	1	1	1	1	1	1	1	0	1	1	1	1	1	1	0	1
	Total	2	3	5	2	3	2	2	0	2	1	4	1	4	2	1	2	2	1	2	2	4	1	1	1	3	2	3	2	2	1	1	0	1
Target	Existence	1	0	1	0	0	0	1	1	1	1	1	1	1	1	1	1	1	1	1	0	0	1	1	1	1	0	1	1	1	1	1	1	1
population	Specificity	2	0	0	0	0	0	0	1	2	1	1	2	1	1	2	0	1	1	1	0	0	1	2	1	0	0	0	0	2	1	0	2	1

		Operability	1	1	2	1	1	1	2	1	1	1	1	2	1	1	1	1	2	2	2	0	1	1	2	2	1	0	0	2	2	1	2	1	1
		Total	4	1	3	1	1	1	3	3	4	3	3	5	3	3	4	2	4	4	4	0	1	3	5	4	2	0	1	3	5	3	3	4	3
	Total score	Existence	13	15	13	11	14	11	11	13	13	10	16	12	14	14	12	13	16	13	15	12	11	11	11	14	14	14	13	12	13	11	14	16	15
		Specificity	22	22	24	16	16	16	11	16	20	13	22	17	24	18	17	16	18	18	18	8	19	13	14	15	17	16	14	12	21	9	18	14	17
		Operability	19	18	30	22	19	17	31	15	20	22	17	32	20	20	23	18	33	31	34	15	22	30	27	33	12	20	21	30	32	21	33	18	19
		Total	54	55	67	49	49	44	53	44	53	45	55	61	58	52	52	47	67	62	67	35	52	54	52	62	43	50	48	54	66	41	65	48	51
Grok-4	Confidentiality	Existence	1	1	1	1	1	1	0	1	1	1	1	1	1	1	1	1	1	1	1	0	1	0	1	1	1	1	1	1	1	1	1	0	1
		Specificity	1	2	2	2	2	2	1	1	2	2	1	1	1	2	2	2	2	1	1	1	2	1	0	1	1	1	1	1	2	0	1	1	2
		Operability	2	1	2	1	2	1	1	1	1	1	2	2	1	1	1	1	2	2	2	1	1	1	2	2	2	1	0	2	2	1	2	1	2
		Total	4	4	5	4	5	4	2	3	4	4	4	4	3	4	4	4	5	4	4	2	4	2	3	4	4	3	2	4	5	2	4	2	5
	Exceptions to confidentiality	Existence	0	1	1	0	1	0	1	1	1	0	1	1	1	1	1	1	1	1	1	1	1	1	0	1	1	1	1	1	1	1	1	1	1
		Specificity	1	2	2	1	2	1	2	1	2	1	2	2	1	2	2	1	2	1	2	2	2	2	1	2	2	1	1	2	1	0	2	1	2
		Operability	1	0	2	1	1	1	2	1	2	1	1	1	1	1	1	1	2	1	2	0	1	2	1	2	1	0	1	2	0	1	2	1	1
		Total	2	3	5	2	4	2	5	3	5	2	4	4	3	4	4	3	5	3	5	3	4	5	2	5	4	2	3	5	2	2	5	3	4
	Client rights	Existence	1	1	1	1	0	1	1	1	1	1	1	1	1	1	1	1	1	1	1	1	1	1	1	0	1	1	1	1	1	1	1	1	1
		Specificity	0	2	2	2	1	1	2	2	2	2	2	2	1	2	2	2	2	2	2	1	2	1	1	1	2	2	2	2	2	2	2	2	2
		Operability	1	1	1	0	0	2	2	2	1	0	1	2	0	1	1	1	2	2	2	1	1	1	2	1	2	1	1	2	1	1	2	1	1
		Total	2	4	4	3	1	4	5	5	4	3	4	5	2	4	4	4	5	5	5	3	4	3	4	2	5	4	4	5	4	4	5	4	4
	Guardian consent	Existence	0	1	1	0	1	1	0	1	0	0	1	0	1	1	1	0	1	0	1	0	1	1	0	0	1	0	0	0	1	0	1	1	1
		Specificity	1	2	1	1	2	1	1	1	1	1	2	1	2	1	2	1	2	0	0	1	1	2	1	1	2	1	1	1	1	1	1	2	2
		Operability	1	1	2	1	1	1	1	2	1	1	1	1	1	1	1	1	2	1	1	0	1	2	1	1	2	1	0	1	2	1	2	1	1
		Total	2	4	4	2	4	3	2	4	2	2	4	2	4	3	4	2	5	1	2	1	3	5	2	2	5	2	1	2	4	2	4	4	4
	Goals and scope	Existence	1	1	1	1	0	1	1	1	1	1	1	1	1	1	1	1	1	1	1	1	1	1	1	1	1	1	1	1	1	1	1	1	1
		Specificity	1	1	2	2	1	2	2	0	1	1	1	2	1	2	2	2	2	1	1	2	2	1	1	2	1	1	0	2	2	2	2	0	2
		Operability	1	1	2	2	1	1	2	1	2	1	1	2	1	1	1	1	1	1	2	1	0	2	2	2	1	1	1	2	2	1	2	1	1
		Total	3	3	5	5	2	4	5	2	4	3	3	5	3	4	4	4	4	3	4	4	3	4	4	5	3	3	2	5	5	4	5	2	4
	Format and frequency	Existence	1	0	1	0	1	1	1	1	1	0	1	1	1	1	1	1	1	1	1	1	1	1	1	0	1	1	1	1	1	1	1	0	0
		Specificity	1	1	2	1	2	2	2	1	2	1	2	2	2	2	2	2	2	1	2	1	0	2	2	1	2	1	1	2	1	1	2	0	1
		Operability	2	1	2	1	1	2	2	1	1	1	1	1	1	1	1	1	2	1	2	1	1	2	2	1	2	0	1	2	2	1	2	1	1
		Total	4	2	5	2	4	5	5	3	4	2	4	4	4	4	4	4	5	3	5	3	2	5	5	2	5	2	3	5	4	3	5	1	2
	Fees and cancellation	Existence	0	1	1	1	1	1	1	1	1	1	0	1	1	1	1	1	1	1	1	1	1	0	0	1	1	1	1	1	1	1	1	1	1
		Specificity	1	1	1	0	1	1	1	1	1	1	1	0	2	1	1	0	1	1	1	1	0	1	1	0	1	1	1	1	1	1	1	2	0

policy	Operability	0	1	2	1	1	1	2	1	1	1	1	2	1	1	1	1	2	2	1	0	1	1	1	2	1	1	0	1	1	2	2	2	1
	Total	1	3	4	2	3	3	4	3	3	3	2	3	4	3	3	2	4	4	3	2	2	2	2	3	3	3	2	3	3	4	4	5	2
Recording methods	Existence	1	1	1	1	1	1	1	1	1	0	0	1	1	1	1	1	1	1	1	1	1	1	1	1	1	1	0	1	0	0	1	1	0
	Specificity	0	2	1	2	2	1	1	1	1	1	1	2	2	2	1	2	1	1	1	1	2	1	2	1	2	2	1	2	1	1	2	2	1
	Operability	1	1	0	1	1	1	2	1	2	1	1	2	0	1	1	1	2	1	1	1	1	2	2	2	2	1	0	2	1	1	2	1	1
	Total	2	4	2	4	4	3	4	3	4	2	2	5	3	4	3	4	4	3	3	3	4	4	5	4	5	4	1	5	2	2	5	4	2
Authorization and revocation	Existence	1	1	1	1	1	1	1	1	0	1	1	1	1	0	1	1	1	1	0	0	1	1	1	1	1	1	1	1	1	1	1	1	1
	Specificity	1	1	2	0	1	1	2	0	1	1	2	0	1	0	1	2	1	0	1	1	0	1	0	1	1	1	0	0	0	0	0	1	1
	Operability	1	1	2	1	1	1	2	1	1	1	1	2	1	1	1	1	2	1	1	1	1	1	1	2	1	1	0	2	2	1	2	1	1
	Total	3	3	5	2	3	3	5	2	2	3	4	3	3	1	3	4	4	2	2	2	2	3	2	4	3	3	1	3	3	2	3	3	3
Crisis procedures	Existence	1	1	1	1	1	1	1	1	1	1	1	1	1	1	1	1	1	1	1	1	1	0	1	1	1	1	1	1	1	1	1	1	0
	Specificity	1	0	1	1	1	2	0	1	1	0	1	0	0	0	0	0	1	2	1	0	0	1	0	2	1	1	0	0	2	1	2	1	1
	Operability	1	0	2	1	1	1	1	1	1	1	1	2	1	1	1	1	2	1	2	1	1	1	2	2	2	1	1	2	2	1	2	1	1
	Total	3	1	4	3	3	4	2	3	3	2	3	3	2	2	2	2	4	4	4	2	2	2	3	5	4	3	2	3	5	3	5	3	2
Complaints and appeals	Existence	1	1	1	0	0	1	0	0	1	0	0	1	0	0	1	0	1	0	0	1	1	0	1	1	1	1	0	0	0	0	1	0	0
	Specificity	1	1	1	0	0	2	0	0	1	1	0	0	0	0	1	1	2	0	0	1	0	0	0	1	1	1	0	0	1	0	0	1	0
	Operability	1	1	2	1	0	0	1	1	1	1	0	2	1	1	1	1	2	1	1	0	1	1	2	1	1	1	1	1	1	1	2	1	1
	Total	3	3	4	1	0	3	1	1	3	2	0	3	1	1	3	2	5	1	1	2	2	1	3	3	3	3	1	1	2	1	3	2	1
Data protection	Existence	0	1	0	1	1	1	1	0	1	1	0	1	0	1	1	0	1	1	0	0	1	0	1	0	1	1	0	0	1	0	1	1	0
	Specificity	0	2	1	0	1	1	1	1	0	2	0	2	1	2	0	1	0	1	0	1	1	0	1	0	2	1	1	1	1	0	0	1	1
	Operability	1	1	1	1	1	1	2	1	0	1	1	2	1	1	1	1	2	1	1	1	0	1	2	1	2	1	0	1	2	1	2	1	1
	Total	1	4	2	2	3	3	4	2	1	4	1	5	2	4	2	2	3	3	1	2	2	1	4	1	5	3	1	2	4	1	3	3	2
Disclaimer of boundaries	Existence	0	0	0	0	0	0	0	0	0	0	0	0	0	1	0	0	1	0	0	0	0	0	0	0	0	0	0	0	1	0	0	0	0
	Specificity	1	1	1	1	1	1	1	1	1	1	1	1	1	2	1	1	2	1	1	1	1	1	1	1	1	1	1	1	2	1	1	1	1
	Operability	1	1	1	1	1	1	1	1	1	1	1	1	1	1	1	1	1	0	1	1	1	1	1	1	1	1	1	1	2	0	1	1	1
	Total	2	2	2	2	2	2	2	2	2	2	2	2	2	4	2	2	4	1	2	2	2	2	2	2	2	2	2	2	5	1	2	2	2
Language clarity	Existence	0	0	0	1	0	0	1	1	0	1	1	0	0	0	0	0	0	0	1	0	0	0	1	0	0	0	0	1	0	0	0	0	1
	Specificity	0	0	0	0	0	1	1	0	0	0	0	0	0	0	1	0	0	0	0	0	0	0	0	1	1	0	0	2	0	0	0	0	1
	Operability	0	0	1	2	1	0	2	1	1	1	0	1	1	1	1	1	1	1	2	1	1	1	2	1	1	1	1	2	1	1	1	1	1
	Total	0	0	1	3	1	1	4	2	1	2	1	1	1	1	2	1	1	1	3	1	1	1	3	2	2	1	1	5	1	1	1	1	3
Voluntariness	Existence	1	1	1	0	0	0	1	0	0	1	1	1	1	1	0	1	0	1	1	0	1	1	0	1	1	1	1	1	0	1	1	0	0
	Specificity	0	0	1	0	0	0	0	0	0	0	0	1	1	0	0	0	0	0	0	1	0	0	0	0	0	0	0	0	0	0	0	1	1

	Operability	2	1	2	1	1	1	2	0	1	1	0	2	1	2	1	1	1	1	2	0	1	2	1	2	2	0	2	2	1	1	2	1	1
	Total	3	2	4	1	1	1	3	0	1	2	1	4	3	3	1	2	1	2	3	1	2	3	1	3	3	1	3	3	1	2	3	2	2
Client obligations	Existence	1	1	1	1	1	1	1	1	1	1	0	1	1	0	1	1	1	0	1	0	0	0	0	1	0	1	1	1	1	1	1	1	1
	Specificity	1	1	0	1	0	2	0	1	0	0	0	2	2	1	0	0	0	0	2	0	0	0	0	0	0	0	0	0	1	0	1	0	2
	Operability	1	0	2	1	1	2	2	1	1	1	1	2	1	1	0	2	2	1	1	1	1	1	1	2	1	0	1	2	2	1	2	2	2
	Total	3	2	3	3	2	5	3	3	2	2	1	5	4	2	1	3	3	1	4	1	1	1	1	3	1	1	2	3	4	2	4	3	5
Counseling limitations	Existence	0	1	0	0	0	1	1	0	1	0	0	1	1	0	1	0	0	0	0	1	0	0	0	1	0	1	0	0	0	0	0	0	0
	Specificity	1	0	0	1	0	0	0	0	1	0	0	0	0	0	0	0	0	0	0	0	0	0	0	0	0	0	0	0	0	0	1	0	0
	Operability	0	1	1	0	0	1	2	1	1	1	1	2	2	1	1	1	1	1	1	1	1	1	1	2	1	1	1	1	1	1	1	1	1
	Total	1	2	1	1	0	2	3	1	3	1	1	3	3	1	2	1	1	1	1	2	1	1	1	3	1	2	1	1	1	1	2	1	1
Counselor qualifications	Existence	1	1	1	1	1	1	1	1	0	0	1	1	1	0	0	1	1	1	0	1	0	1	1	1	1	1	0	1	1	1	1	1	1
	Specificity	0	0	0	0	0	0	1	0	0	0	0	0	0	0	0	0	0	0	0	0	0	0	0	0	0	0	0	0	0	0	0	0	0
	Operability	1	1	1	1	1	1	2	2	1	1	0	2	1	1	0	1	2	2	1	1	1	2	2	2	2	2	1	1	2	1	2	1	1
	Total	2	2	2	2	2	2	4	3	1	1	1	3	2	1	0	2	3	3	1	2	1	3	3	3	3	3	1	2	3	2	3	2	2
Counseling modalities	Existence	0	1	1	0	1	0	0	0	0	1	0	0	0	0	0	0	0	0	0	0	1	0	1	0	1	0	1	0	0	0	0	0	0
	Specificity	0	1	2	1	2	1	1	1	1	2	1	1	1	1	1	1	1	1	1	1	1	1	2	1	0	1	2	1	1	1	1	1	1
	Operability	0	1	2	1	1	0	1	1	1	0	1	1	1	1	1	1	1	1	1	1	1	1	2	1	2	0	1	1	1	1	1	0	1
	Total	0	3	5	2	4	1	2	2	2	3	2	2	2	2	2	2	2	2	2	2	3	2	5	2	3	1	4	2	2	2	2	1	2
Target population	Existence	1	1	1	1	0	1	1	1	1	1	1	1	1	1	1	1	0	1	1	1	0	1	1	1	1	0	1	1	1	1	1	1	1
	Specificity	2	2	0	1	0	0	0	0	2	1	1	1	1	0	1	1	0	0	0	1	0	1	2	0	1	0	0	0	0	0	0	0	1
	Operability	1	1	2	1	1	1	2	1	2	1	0	2	1	1	1	1	1	1	1	0	1	2	2	2	2	1	1	2	2	1	2	1	2
	Total	4	4	3	3	1	2	3	2	5	3	2	4	3	2	3	3	1	2	2	2	1	4	5	3	4	1	2	3	3	2	3	2	4
Total score	Existence	12	17	16	12	12	15	15	14	13	12	12	16	15	13	15	13	15	13	13	11	14	10	13	13	16	15	12	14	14	12	16	12	11
	Specificity	14	22	22	17	19	22	19	13	20	18	18	20	20	20	20	19	21	13	16	17	14	16	15	16	21	16	12	18	19	11	19	17	22
	Operability	19	16	32	20	18	20	34	22	23	18	16	34	19	21	18	21	33	23	28	14	18	28	32	32	31	16	15	32	30	20	36	21	23
	Total	45	55	70	49	49	57	68	49	56	48	46	70	54	54	53	53	69	49	57	42	46	54	60	61	68	47	39	64	63	43	71	50	56

Supplementary Data S6. Table of detailed ratings by Reader 01 for all documents

Version	Dimension	Document ID																																
		01	02	03	04	05	06	07	08	09	10	11	12	13	14	15	16	17	18	19	20	21	22	23	24	25	26	27	28	29	30	31	32	33
Original	Comprehensibility	3	2	1	3	3	3	3	4	1	3	5	4	4	2	3	3	3	3	3	2	4	2	3	3	3	2	3	3	3	2	2	3	3
	Clarity	4	2	1	3	3	3	3	4	2	3	5	2	4	3	3	4	3	3	3	3	3	3	3	5	4	3	4	2	4	3	3	3	3
	Trustworthiness	3	4	3	3	3	4	3	3	3	4	4	3	3	3	4	3	3	4	4	3	4	4	4	4	4	3	3	3	3	4	3	3	3
	Friendliness	4	2	3	3	4	4	3	3	3	3	3	2	3	2	2	3	3	3	3	3	4	4	3	3	4	3	4	3	3	3	2	3	3
	Professionalism	3	3	3	4	4	4	3	3	4	3	3	4	3	3	4	3	4	3	3	4	3	3	3	3	3	2	4	4	3	4	3	4	4
	Acceptability	3	3	3	4	3	3	3	3	4	4	3	3	4	3	3	5	4	4	4	4	3	3	4	3	3	4	3	5	4	3	4	4	4
CHatGPT-5	Comprehensibility	3	4	2	4	4	3	3	4	3	3	3	4	4	4	4	4	3	4	3	4	5	3	4	4	3	4	4	4	4	4	3	3	4
	Clarity	4	3	4	3	3	4	4	4	4	3	4	4	4	4	4	4	4	4	4	3	4	4	4	4	3	4	5	3	5	4	4	4	5
	Trustworthiness	4	3	4	4	3	5	4	4	4	4	4	4	4	4	4	3	3	3	3	4	4	4	4	4	4	4	4	3	4	4	4	4	4
	Friendliness	3	3	3	3	4	3	3	3	2	3	3	4	3	3	3	4	3	4	3	3	4	3	4	2	4	3	3	3	4	3	4	2	4
	Professionalism	3	4	3	3	3	4	4	3	3	2	4	3	3	3	3	3	4	3	4	4	4	4	3	4	3	4	3	4	3	4	3	3	4
	Acceptability	4	4	3	4	4	3	4	4	5	4	4	5	4	4	4	5	4	4	5	4	3	4	3	4	4	5	5	4	4	4	4	3	5
Grok-4	Comprehensibility	4	4	4	3	5	3	3	3	2	4	4	4	3	4	3	4	4	4	3	3	4	3	3	3	2	4	3	4	4	4	3	4	5
	Clarity	4	4	4	4	4	5	4	4	4	5	4	4	3	3	3	3	4	4	4	4	4	4	3	4	3	3	3	4	4	4	4	3	4
	Trustworthiness	4	4	4	3	3	2	4	4	3	4	4	3	4	3	3	3	5	4	4	3	3	4	4	4	4	3	3	4	3	4	3	4	3
	Friendliness	4	3	4	3	4	3	3	3	4	2	3	4	4	4	3	4	3	4	4	3	4	4	3	3	4	3	4	4	3	4	3	3	3
	Professionalism	4	4	4	4	3	4	3	3	3	2	4	3	3	3	4	3	3	3	4	3	3	4	3	4	5	3	4	4	3	5	4	4	3
	Acceptability	4	4	4	4	4	4	4	4	4	3	4	4	5	3	4	3	4	5	4	4	4	4	3	4	3	4	4	4	5	4	4	4	5

Supplementary Data S7. Table of detailed ratings by Reader 02 for all documents

Version	Dimension	Document ID																																
		01	02	03	04	05	06	07	08	09	10	11	12	13	14	15	16	17	18	19	20	21	22	23	24	25	26	27	28	29	30	31	32	33
Original	Comprehensibility	3	2	2	3	2	3	2	3	2	3	3	4	3	3	3	3	3	4	3	3	4	3	3	3	2	2	3	2	3	2	2	3	3
	Clarity	3	2	2	3	2	3	2	3	2	3	3	3	3	3	3	3	3	4	3	3	4	3	2	3	3	3	3	3	3	3	2	3	3
	Trustworthiness	3	3	3	3	3	3	3	4	2	3	3	2	3	3	4	3	3	3	4	3	4	4	4	4	3	3	3	3	3	4	3	3	2
	Friendliness	3	3	4	3	4	4	3	4	4	3	5	3	3	2	4	2	4	3	3	3	4	4	3	3	5	4	4	3	4	3	3	3	4
	Professionalism	3	3	2	3	2	3	4	3	3	3	2	2	3	3	2	3	4	3	2	3	3	3	2	3	3	2	4	4	2	2	3	3	3
	Acceptability	3	2	3	4	3	3	3	3	2	2	3	3	3	2	3	4	2	3	3	3	3	3	2	3	3	3	3	2	4	3	3	3	4
CHatGPT-5	Comprehensibility	4	4	4	3	4	3	4	3	3	4	3	4	4	4	4	4	4	4	3	4	4	3	3	4	4	5	3	3	4	4	4	3	4
	Clarity	4	2	3	4	4	3	4	4	4	4	4	4	4	4	4	3	4	4	3	2	3	4	4	4	4	3	4	3	4	4	3	3	5
	Trustworthiness	4	4	4	4	4	4	4	4	4	4	4	4	3	4	4	3	3	3	4	4	5	4	4	3	3	4	4	4	3	4	3	3	3
	Friendliness	4	4	4	4	5	3	4	4	3	4	4	5	3	4	3	4	4	4	4	3	4	4	4	3	4	5	4	4	3	4	3	4	4
	Professionalism	2	3	4	4	3	3	3	4	3	3	3	3	3	2	3	2	4	4	3	3	3	3	3	3	2	4	3	4	3	3	4	3	3
	Acceptability	3	4	4	3	3	3	4	4	4	4	3	3	3	3	4	4	3	4	5	4	2	4	3	4	3	4	4	3	3	4	4	3	5
Grok-4	Comprehensibility	3	3	2	3	5	3	3	4	2	4	4	4	3	4	3	3	5	4	4	3	3	4	3	4	3	4	3	4	4	4	3	4	4
	Clarity	4	3	3	3	4	5	4	3	4	3	4	4	4	4	4	3	4	4	3	3	4	5	3	3	4	4	3	3	4	3	5	4	4
	Trustworthiness	3	3	4	3	3	3	4	4	3	4	4	4	4	4	4	3	4	3	4	3	4	4	3	4	3	3	4	3	3	4	4	3	3
	Friendliness	3	4	4	4	5	4	4	4	4	4	4	4	4	4	4	4	4	4	3	5	4	4	3	4	4	4	4	4	3	4	4	4	4
	Professionalism	3	4	3	3	3	4	3	3	4	3	4	3	3	3	2	3	4	3	4	4	3	2	3	3	4	2	4	3	4	4	4	4	4
	Acceptability	3	4	2	4	5	3	4	3	4	2	3	3	3	3	3	4	3	4	4	3	3	3	2	3	3	3	4	4	3	3	3	4	5

Supplementary Data S8. Table of detailed ratings by Reader 03 for all documents

Version	Dimension	Document ID																																
		01	02	03	04	05	06	07	08	09	10	11	12	13	14	15	16	17	18	19	20	21	22	23	24	25	26	27	28	29	30	31	32	33
Original	Comprehensibility	3	2	2	3	3	4	3	3	2	3	4	3	3	3	4	3	3	3	4	3	3	2	3	4	2	3	3	3	3	2	3	4	3
	Clarity	2	2	3	3	2	3	3	3	3	3	3	2	4	2	5	4	3	3	4	2	3	3	2	3	2	3	3	3	3	3	2	3	3
	Trustworthiness	3	3	3	3	3	3	4	3	3	4	3	3	3	3	3	3	3	4	3	3	4	3	3	3	3	2	3	4	3	4	3	2	3
	Friendliness	4	3	3	4	4	3	3	3	3	3	4	4	4	3	3	4	4	4	4	3	4	3	4	3	4	5	3	3	3	4	3	3	4
	Professionalism	3	4	3	3	3	4	3	4	4	3	3	3	4	3	3	3	3	3	4	3	4	3	3	3	4	3	4	4	3	4	4	3	4
	Acceptability	4	2	3	3	2	3	3	4	3	3	3	3	4	2	3	4	2	3	4	2	3	3	3	3	3	3	3	3	4	3	2	3	4
CHatGPT-5	Comprehensibility	4	4	4	3	4	4	4	3	4	4	4	4	4	5	4	4	4	4	3	3	4	3	4	4	4	4	4	4	4	4	4	4	4
	Clarity	4	4	3	4	4	3	4	4	3	4	4	4	3	3	4	4	4	4	4	3	4	4	3	4	3	3	4	4	4	4	3	3	4
	Trustworthiness	4	4	3	4	2	3	3	4	4	4	3	3	3	4	4	4	4	4	4	4	4	5	4	4	2	4	3	3	3	4	4	3	3
	Friendliness	3	3	4	4	4	3	4	4	4	4	4	4	3	4	3	3	4	4	4	3	4	4	4	4	4	5	3	4	4	4	3	4	4
	Professionalism	3	4	3	3	4	4	4	4	4	4	4	4	4	3	4	4	4	4	4	3	4	3	3	4	4	4	5	4	3	3	4	4	4
	Acceptability	4	4	3	4	3	4	3	4	4	4	3	4	4	4	3	4	3	5	4	3	3	3	4	3	3	5	4	4	5	4	4	3	4
Grok-4	Comprehensibility	3	4	3	3	4	4	4	4	3	4	4	4	4	5	3	4	3	5	4	3	4	4	4	4	4	4	3	3	5	4	4	5	5
	Clarity	4	4	3	4	4	4	4	3	3	4	4	4	4	4	4	4	3	4	4	3	4	4	4	4	4	4	4	3	4	4	4	4	3
	Trustworthiness	4	4	4	3	4	3	4	3	4	3	4	3	4	4	4	3	4	3	4	3	4	5	3	4	3	4	3	3	4	4	4	3	3
	Friendliness	3	3	4	4	4	3	3	4	4	3	4	3	4	3	4	4	5	4	3	3	4	4	4	4	4	4	4	4	4	4	3	3	5
	Professionalism	4	4	4	4	3	4	5	3	3	4	4	3	4	4	3	4	4	4	4	3	4	4	3	4	3	4	4	4	4	4	4	3	4
	Acceptability	3	3	3	4	4	3	3	3	3	3	4	3	3	3	3	4	2	4	4	3	3	4	3	3	3	4	4	3	4	4	3	4	5

Supplementary Data S9. Table of detailed ratings by Reader 04 for all documents

Version	Dimension	Document ID																																
		01	02	03	04	05	06	07	08	09	10	11	12	13	14	15	16	17	18	19	20	21	22	23	24	25	26	27	28	29	30	31	32	33
Original	Comprehensibility	2	2	2	2	2	2	2	4	2	3	3	2	4	3	4	3	3	3	3	3	4	2	3	3	3	2	3	2	4	2	2	3	3
	Clarity	4	2	3	4	3	3	4	4	2	4	5	3	4	3	4	4	4	4	3	4	4	4	3	4	3	3	4	2	5	4	3	3	3
	Trustworthiness	4	4	4	3	3	3	4	4	3	4	4	4	3	2	3	3	4	3	4	3	4	4	3	4	4	4	4	4	3	4	4	3	3
	Friendliness	3	3	3	4	4	3	2	4	3	3	4	4	2	2	3	3	4	4	4	3	3	4	4	3	3	4	4	3	3	3	4	3	5
	Professionalism	3	4	4	5	5	4	4	4	4	4	4	4	4	3	3	4	4	4	4	5	3	3	4	4	4	4	4	4	4	3	5	3	4
	Acceptability	3	2	2	3	3	3	3	3	3	4	4	4	3	2	3	4	3	3	4	3	4	3	3	3	4	3	3	4	5	3	3	4	4
CHatGPT-5	Comprehensibility	4	4	4	4	3	5	3	4	3	3	3	4	4	4	4	4	3	4	3	3	5	4	4	4	3	4	3	3	3	4	3	3	4
	Clarity	4	3	4	5	5	4	4	4	4	4	5	4	4	4	4	4	5	5	4	3	4	4	4	5	5	5	4	3	4	5	3	4	3
	Trustworthiness	4	4	4	4	3	4	4	4	4	4	3	4	3	3	3	3	4	4	3	5	4	4	4	4	3	4	4	5	3	4	3	4	3
	Friendliness	3	5	4	4	4	4	4	4	4	4	4	5	3	4	3	3	4	5	3	4	4	4	4	3	4	5	3	4	4	4	4	4	4
	Professionalism	3	4	3	4	4	4	4	5	4	3	4	5	3	4	4	3	4	4	5	3	4	4	4	4	4	3	4	5	4	4	4	4	5
	Acceptability	3	4	3	3	5	4	4	4	4	4	3	4	4	4	4	5	4	4	4	4	4	4	3	3	3	4	4	3	4	4	4	4	5
Grok-4	Comprehensibility	3	4	3	3	4	4	3	3	3	4	4	3	4	4	4	3	4	4	4	3	3	3	3	4	4	3	3	3	4	3	5	4	4
	Clarity	4	4	4	3	5	4	4	5	3	4	4	3	4	4	4	4	4	5	3	4	4	4	4	4	4	4	5	5	4	5	5	3	4
	Trustworthiness	4	4	4	3	3	3	3	4	4	4	4	4	4	3	4	2	4	4	4	3	4	5	3	3	4	4	4	4	4	4	3	4	3
	Friendliness	4	4	4	3	5	3	3	4	4	3	3	4	3	4	2	4	4	4	4	3	4	3	4	4	4	4	4	5	4	3	3	3	4
	Professionalism	3	4	4	4	4	4	4	3	3	3	5	4	4	4	3	4	5	3	4	3	4	5	3	4	4	3	4	5	3	5	4	4	4
	Acceptability	4	4	3	3	4	4	3	4	4	5	4	4	3	4	4	4	3	4	4	3	3	4	3	3	4	4	4	4	4	3	4	5	4

Supplementary Data S10. Table of detailed ratings by Reader 05 for all documents

Version	Dimension	Document ID																																
		01	02	03	04	05	06	07	08	09	10	11	12	13	14	15	16	17	18	19	20	21	22	23	24	25	26	27	28	29	30	31	32	33
Original	Comprehensibility	2	2	2	3	3	2	2	3	2	2	3	3	3	3	3	3	4	3	3	4	4	3	2	3	3	2	4	3	3	3	3	3	3
	Clarity	3	2	2	3	3	3	3	4	2	2	4	3	3	2	3	3	4	3	3	3	3	3	2	3	4	3	3	3	4	3	2	2	2
	Trustworthiness	4	4	4	3	3	4	3	4	4	4	4	3	4	3	4	3	3	4	4	4	4	5	4	4	4	4	4	4	3	4	4	3	4
	Friendliness	4	3	4	3	4	5	3	4	4	4	3	4	3	3	3	4	4	3	4	3	4	4	4	3	4	3	4	4	2	3	3	3	4
	Professionalism	2	4	3	4	3	4	4	4	3	3	3	4	2	3	3	3	5	3	3	4	2	4	3	4	3	3	3	3	3	2	4	3	4
	Acceptability	4	3	3	3	3	4	3	3	3	3	3	3	4	2	3	4	3	3	4	3	3	4	3	3	4	4	4	3	3	4	3	4	5
CHatGPT-5	Comprehensibility	3	3	3	3	4	3	3	3	3	3	4	4	5	4	4	4	4	5	4	4	4	3	3	4	4	5	4	2	4	4	4	3	4
	Clarity	4	3	4	4	4	4	4	4	3	4	4	4	4	4	5	5	4	4	3	3	4	4	4	4	4	5	4	4	4	4	4	3	3
	Trustworthiness	5	4	3	4	5	4	5	5	5	4	3	3	4	4	5	3	4	4	5	4	5	5	5	4	4	4	4	4	4	4	4	5	3
	Friendliness	4	5	4	5	4	4	4	4	5	4	4	5	4	4	4	4	5	4	3	3	4	5	3	5	4	5	4	4	2	3	4	4	4
	Professionalism	4	4	3	4	4	3	4	3	3	4	4	4	3	3	3	2	4	3	3	4	3	3	4	4	4	4	3	4	3	4	4	4	4
	Acceptability	4	3	3	4	4	3	3	3	3	4	3	4	4	3	3	5	4	5	4	4	4	4	3	3	3	4	5	4	5	4	4	3	3
Grok-4	Comprehensibility	2	5	4	3	4	3	3	3	3	3	4	3	4	4	5	3	4	4	4	3	3	3	4	3	3	4	3	4	4	3	4	4	4
	Clarity	4	3	3	3	5	4	4	3	3	4	4	4	4	3	4	4	3	4	4	4	4	4	3	4	3	4	4	4	4	4	3	3	3
	Trustworthiness	4	4	4	3	3	4	4	4	4	4	4	4	4	3	4	4	3	4	4	4	5	5	4	4	3	4	4	4	4	4	3	4	3
	Friendliness	4	4	4	4	4	5	3	3	4	4	4	4	4	5	3	4	3	5	3	4	4	4	4	4	5	4	4	4	3	3	4	3	5
	Professionalism	3	4	4	4	4	4	3	4	4	3	4	4	3	4	4	4	4	4	4	4	4	4	4	4	4	4	4	4	3	4	4	4	4
	Acceptability	3	3	3	4	4	3	4	4	4	4	4	3	4	5	3	4	3	4	5	4	4	3	3	4	4	3	4	4	4	4	3	4	5

Supplementary Data S11. Table of detailed ratings by Reader 06 for all documents

Version	Dimension	Document ID																																
		01	02	03	04	05	06	07	08	09	10	11	12	13	14	15	16	17	18	19	20	21	22	23	24	25	26	27	28	29	30	31	32	33
Original	Comprehensibility	3	3	3	3	3	3	4	4	3	3	5	5	4	3	4	3	4	4	4	4	4	4	3	5	3	4	5	3	5	4	4	3	4
	Clarity	3	2	3	3	4	3	2	4	3	4	5	3	4	3	4	3	4	4	3	2	3	4	3	4	4	3	4	3	4	3	4	4	3
	Trustworthiness	3	3	2	4	4	3	3	3	3	3	5	3	3	3	3	3	3	3	2	3	4	4	3	3	3	2	2	3	3	4	2	2	3
	Friendliness	3	4	3	3	4	3	3	4	3	4	4	4	3	3	4	3	3	4	4	3	4	4	3	4	3	4	5	4	4	4	3	3	4
	Professionalism	3	4	3	3	3	3	4	3	4	4	3	3	4	4	3	3	3	4	4	3	4	4	3	3	4	4	4	4	4	4	4	3	3
	Acceptability	3	2	2	3	3	2	2	2	2	3	3	2	3	3	3	3	3	3	3	3	3	2	3	3	2	3	4	3	4	3	3	4	3
CHatGPT-5	Comprehensibility	4	5	4	4	5	5	4	4	4	5	4	5	4	5	5	4	5	5	5	5	4	4	5	5	4	4	5	4	5	4	4	5	5
	Clarity	5	4	4	4	4	4	4	4	4	4	5	5	4	5	4	5	5	4	4	5	4	5	4	4	5	5	4	4	5	4	4	4	5
	Trustworthiness	3	3	3	3	3	4	4	4	3	4	3	3	4	4	4	4	3	3	4	3	5	5	4	3	3	4	4	3	3	4	3	4	3
	Friendliness	4	4	4	4	5	5	4	4	4	3	3	4	4	4	2	3	4	4	3	3	4	4	5	4	4	5	4	4	4	4	4	4	4
	Professionalism	4	3	3	4	3	4	4	3	4	4	4	4	4	3	4	3	5	3	4	4	4	3	3	4	4	4	5	5	4	4	4	4	4
	Acceptability	3	3	3	4	3	4	2	4	3	3	3	3	3	3	3	4	4	4	3	3	3	3	3	3	3	4	3	4	4	3	3	4	4
Grok-4	Comprehensibility	4	4	4	4	5	4	3	5	4	5	5	5	5	5	4	4	4	5	3	5	4	5	5	5	4	5	5	4	5	5	4	5	5
	Clarity	5	4	4	3	5	4	4	4	4	4	5	4	4	5	5	4	5	5	4	4	4	5	3	4	5	4	4	4	5	4	4	4	4
	Trustworthiness	3	4	4	3	3	3	3	3	4	4	3	4	4	3	4	4	4	4	3	2	4	4	4	4	3	4	2	4	3	4	4	3	3
	Friendliness	4	4	4	4	5	4	4	3	4	4	4	4	5	4	4	4	3	4	4	4	4	5	5	4	4	4	4	4	4	3	4	4	5
	Professionalism	3	4	4	5	3	4	4	4	4	3	5	4	4	4	4	3	4	4	4	3	4	3	3	4	3	4	5	4	3	4	4	3	4
	Acceptability	3	2	3	3	4	3	3	3	3	4	4	3	4	4	4	4	3	3	4	3	3	4	3	2	3	4	4	3	4	3	4	4	4

Supplementary Data S12. Table of detailed ratings by Reader 07 for all documents

Version	Dimension	Document ID																																
		01	02	03	04	05	06	07	08	09	10	11	12	13	14	15	16	17	18	19	20	21	22	23	24	25	26	27	28	29	30	31	32	33
Original	Comprehensibility	3	2	2	3	2	3	2	4	3	3	4	4	4	3	4	3	3	3	3	2	3	3	2	4	2	3	3	3	4	3	3	3	3
	Clarity	3	1	2	4	3	3	3	3	2	3	5	3	4	2	3	4	4	4	3	4	2	2	2	4	2	3	4	2	3	3	3	3	3
	Trustworthiness	4	3	3	4	4	4	3	3	2	3	4	3	3	3	3	4	4	3	4	2	4	4	4	3	3	4	3	3	3	3	3	3	2
	Friendliness	4	3	3	3	4	3	3	4	4	3	3	3	3	2	3	4	3	4	3	3	4	4	3	3	4	4	4	4	3	3	2	3	4
	Professionalism	3	3	3	4	3	3	3	3	4	2	3	2	4	3	3	2	3	3	4	3	3	3	3	3	3	3	4	3	3	2	4	3	3
	Acceptability	3	3	3	4	3	3	4	3	4	2	4	4	3	3	4	5	3	4	5	4	3	4	3	4	3	3	4	3	5	3	4	3	4
CHatGPT-5	Comprehensibility	4	3	3	3	4	4	4	4	3	4	4	4	4	4	4	4	4	4	4	4	4	3	4	4	4	5	4	3	4	4	5	3	4
	Clarity	4	4	3	4	4	4	3	4	3	4	3	3	3	4	4	4	4	4	3	3	4	5	4	4	4	4	4	4	4	4	4	3	4
	Trustworthiness	3	4	3	4	4	4	4	4	3	4	3	4	3	4	4	4	4	4	4	3	4	4	3	4	3	3	3	4	3	4	4	4	3
	Friendliness	4	4	4	4	4	4	3	3	5	3	4	3	4	4	3	4	4	4	3	4	4	3	4	3	4	4	4	5	3	4	4	3	4
	Professionalism	3	3	3	3	4	5	3	4	4	3	4	5	4	3	3	3	4	3	4	3	3	3	2	3	4	3	4	4	4	4	4	4	4
	Acceptability	4	3	4	4	5	3	4	3	4	4	4	4	4	4	3	5	4	5	4	4	4	4	3	5	4	5	4	4	5	4	4	4	5
Grok-4	Comprehensibility	3	4	3	3	5	5	3	3	4	4	4	3	4	5	4	3	4	4	3	4	4	3	4	4	3	4	4	4	4	4	3	4	4
	Clarity	3	4	4	3	3	4	4	4	4	3	5	4	4	3	4	4	4	4	3	3	3	4	3	3	3	4	4	4	4	4	4	4	4
	Trustworthiness	4	4	4	4	3	3	3	4	4	3	3	2	4	3	3	3	3	3	4	3	4	4	5	3	3	3	3	5	3	4	3	3	4
	Friendliness	4	3	4	4	4	3	3	3	4	3	4	4	5	4	4	4	4	4	4	4	4	4	4	4	3	5	3	4	3	4	4	4	3
	Professionalism	3	4	3	5	4	5	4	3	4	3	3	3	3	3	4	4	4	3	4	3	4	4	4	4	4	3	4	4	3	3	4	3	4
	Acceptability	4	4	3	4	4	3	4	3	4	4	4	3	4	4	5	5	4	4	5	4	3	5	4	4	4	3	5	3	4	4	4	4	5

Supplementary Data S13. Table of detailed ratings by Reader 08 for all documents

Version	Dimension	Document ID																																
		01	02	03	04	05	06	07	08	09	10	11	12	13	14	15	16	17	18	19	20	21	22	23	24	25	26	27	28	29	30	31	32	33
Original	Comprehensibility	3	3	2	3	3	3	2	3	2	2	4	3	3	2	4	3	4	4	2	3	4	3	3	3	3	2	3	3	3	3	3	2	3
	Clarity	3	2	2	2	2	2	3	4	2	3	3	3	2	2	4	3	4	4	3	2	3	3	2	3	2	3	4	3	3	3	3	1	3
	Trustworthiness	3	4	3	3	2	3	2	3	3	4	3	3	3	3	2	3	3	3	3	3	4	3	3	3	2	3	3	3	3	4	3	3	3
	Friendliness	3	3	3	3	4	2	3	4	3	3	3	2	2	3	3	3	3	4	4	2	2	2	3	4	4	4	3	2	3	3	3	2	4
	Professionalism	3	3	4	3	3	4	3	3	2	2	2	4	3	2	3	2	4	3	3	3	2	2	3	3	2	3	4	3	4	3	3	3	3
	Acceptability	2	2	3	3	3	3	2	3	2	3	3	3	3	3	4	4	2	4	3	3	3	3	2	3	2	3	3	3	4	3	3	3	4
CHatGPT-5	Comprehensibility	3	4	2	3	4	4	3	4	4	3	4	4	4	3	4	4	4	4	4	4	5	3	4	4	3	3	4	4	3	4	3	4	4
	Clarity	3	3	4	3	4	3	4	4	4	3	4	3	4	3	4	3	4	4	4	4	4	4	3	3	3	4	4	3	4	3	4	4	3
	Trustworthiness	4	3	3	3	3	4	4	3	3	4	3	3	3	3	4	3	3	3	3	3	3	4	4	4	2	3	3	3	4	4	4	4	3
	Friendliness	3	4	3	3	3	3	3	3	4	3	3	3	3	3	2	3	3	4	2	3	4	4	3	3	4	4	3	4	3	3	3	3	4
	Professionalism	3	4	3	4	4	4	4	3	4	3	4	3	3	3	4	3	4	2	4	3	3	3	3	3	4	4	4	5	3	4	4	4	4
	Acceptability	4	4	3	4	3	3	3	3	3	4	4	4	4	3	3	4	4	4	4	3	3	4	3	3	3	4	4	4	5	4	5	4	4
Grok-4	Comprehensibility	4	3	3	2	4	4	3	4	4	4	5	3	4	4	4	3	4	4	4	4	4	4	3	4	4	4	4	3	4	3	3	4	4
	Clarity	4	4	4	3	5	3	3	2	2	3	4	3	3	4	5	3	4	3	3	3	3	4	3	4	4	4	4	3	4	3	4	3	3
	Trustworthiness	4	4	4	3	3	3	4	3	4	3	3	3	4	3	3	3	3	3	4	4	3	5	4	4	3	3	3	3	4	3	3	3	3
	Friendliness	3	4	3	3	4	2	2	3	3	3	3	3	3	3	2	4	3	3	4	3	3	4	3	4	3	3	3	4	4	3	4	3	4
	Professionalism	3	4	3	3	4	3	3	3	4	3	4	4	3	3	3	3	3	3	4	4	3	4	4	3	4	2	4	3	3	3	4	4	3
	Acceptability	4	4	3	4	4	3	3	3	4	4	4	3	3	5	3	3	4	4	4	3	3	3	3	4	3	4	4	3	4	3	3	3	4

Supplementary Data S14. Table of detailed ratings by Reader 09 for all documents

Version	Dimension	Document ID																																
		01	02	03	04	05	06	07	08	09	10	11	12	13	14	15	16	17	18	19	20	21	22	23	24	25	26	27	28	29	30	31	32	33
Original	Comprehensibility	2	3	2	3	3	1	2	3	2	2	3	2	3	2	3	4	3	3	2	2	3	2	3	3	3	3	3	1	3	2	3	3	3
	Clarity	3	1	2	2	3	2	3	3	2	3	3	2	3	2	3	3	2	3	2	3	3	3	3	3	2	2	3	3	3	3	3	3	3
	Trustworthiness	4	3	3	4	4	4	4	3	3	4	4	4	3	3	4	4	3	4	3	3	4	4	4	4	4	4	3	3	3	4	3	3	3
	Friendliness	3	3	3	3	3	4	2	4	3	4	3	4	4	2	3	3	4	3	4	4	2	3	4	3	4	4	3	3	2	2	3	4	4
	Professionalism	3	4	3	4	4	3	4	3	4	3	3	3	3	3	3	4	3	3	3	3	3	3	4	4	3	3	4	4	3	3	3	4	3
	Acceptability	3	2	3	4	2	2	3	4	3	3	3	3	4	3	3	4	4	4	4	3	2	3	3	4	3	4	4	3	3	2	3	4	3
CHatGPT-5	Comprehensibility	3	4	3	3	4	3	2	4	4	3	3	3	4	3	4	3	3	4	3	3	4	3	3	3	3	4	4	3	4	3	3	4	3
	Clarity	4	2	4	3	4	3	3	3	3	4	3	3	4	3	3	3	4	3	3	3	4	4	3	4	3	4	3	3	4	4	3	3	3
	Trustworthiness	4	4	4	4	4	4	4	4	4	4	4	5	4	4	4	4	4	4	4	4	4	4	4	5	4	3	4	3	4	4	4	3	3
	Friendliness	4	4	4	4	4	4	3	3	4	2	4	4	4	4	3	3	4	4	4	4	5	4	4	3	5	4	3	3	3	3	4	3	5
	Professionalism	4	4	4	3	3	5	4	4	4	4	4	4	4	3	3	3	4	3	4	3	4	4	3	4	4	3	4	4	3	4	4	4	5
	Acceptability	4	3	3	4	4	3	3	3	3	4	4	4	4	3	4	5	3	4	4	4	4	2	4	4	3	5	4	4	4	4	4	4	5
Grok-4	Comprehensibility	4	3	3	3	3	4	3	4	3	3	3	3	4	4	3	4	3	4	2	3	4	3	3	3	3	3	3	4	3	2	3	2	4
	Clarity	3	3	3	2	4	3	5	4	2	3	5	3	4	4	4	4	4	4	3	3	3	5	3	4	3	3	4	3	3	3	4	4	4
	Trustworthiness	5	4	4	3	3	4	4	5	4	4	4	4	4	3	3	4	4	3	4	4	4	5	4	4	4	4	3	4	3	5	4	3	5
	Friendliness	4	4	4	4	4	3	4	4	4	4	4	3	4	3	4	3	4	4	3	3	4	4	3	3	4	5	3	4	3	3	3	3	4
	Professionalism	3	4	4	4	4	4	4	4	4	4	5	4	4	4	4	3	4	3	4	3	3	4	4	3	4	4	5	5	3	3	4	4	4
	Acceptability	3	3	3	3	4	3	4	4	3	3	3	3	4	4	4	4	4	4	4	4	4	4	3	4	3	4	4	5	4	3	3	3	4

Supplementary Data S15. Table of detailed ratings by Reader 10 for all documents

Version	Dimension	Document ID																																
		01	02	03	04	05	06	07	08	09	10	11	12	13	14	15	16	17	18	19	20	21	22	23	24	25	26	27	28	29	30	31	32	33
Original	Comprehensibility	3	3	2	3	2	3	2	3	1	2	3	3	4	3	3	3	3	4	3	3	4	3	3	3	2	3	3	3	3	3	2	3	3
	Clarity	2	2	2	3	4	2	3	3	2	3	3	3	3	2	3	4	3	3	3	2	2	3	2	4	3	3	3	2	3	4	3	3	2
	Trustworthiness	3	3	3	3	3	2	3	4	3	3	4	4	4	3	3	2	3	3	3	2	4	4	3	3	2	3	3	4	4	5	4	3	3
	Friendliness	3	3	3	4	4	4	3	3	3	4	3	4	3	3	4	4	3	4	4	3	4	4	3	3	3	4	4	3	3	4	3	3	4
	Professionalism	2	3	2	3	2	3	3	3	3	3	3	3	3	3	2	3	3	3	3	3	3	3	3	3	3	3	3	3	2	3	3	4	2
	Acceptability	4	2	3	3	3	3	3	4	4	3	3	4	4	3	3	4	3	4	5	3	3	3	3	4	3	3	4	3	5	2	4	4	4
CHatGPT-5	Comprehensibility	3	4	3	4	4	3	3	5	4	4	2	4	4	3	4	5	4	4	4	3	4	4	4	4	4	4	4	3	4	4	3	4	5
	Clarity	3	3	3	3	4	3	4	4	3	3	3	4	3	4	4	3	4	5	3	3	4	4	4	4	4	4	4	4	4	4	3	3	4
	Trustworthiness	4	4	4	3	4	4	4	3	3	4	4	3	4	4	4	3	4	3	4	3	5	4	3	4	4	4	3	4	4	3	3	3	4
	Friendliness	4	4	3	5	5	4	4	4	4	4	4	5	4	4	4	4	5	4	3	5	4	4	4	5	4	4	4	4	3	4	3	4	4
	Professionalism	3	4	3	3	3	3	4	3	3	3	3	3	4	2	3	3	3	3	4	4	3	3	2	3	4	3	4	3	3	3	3	3	2
	Acceptability	3	4	5	4	4	4	4	3	4	4	3	4	4	4	4	5	3	4	4	3	4	4	4	3	3	5	5	4	5	3	4	5	5
Grok-4	Comprehensibility	3	4	4	4	4	4	3	4	3	3	4	3	4	4	5	4	4	4	3	4	3	3	3	4	3	4	4	4	4	4	4	5	4
	Clarity	4	3	4	4	4	3	3	4	3	4	4	3	4	4	4	2	4	3	3	3	3	3	3	4	4	3	4	3	3	3	3	4	4
	Trustworthiness	4	4	3	3	4	3	4	3	4	4	4	4	3	3	4	4	3	3	3	3	4	5	4	4	4	4	3	4	4	4	4	3	4
	Friendliness	4	4	3	3	4	4	3	3	4	4	4	4	4	4	3	4	4	5	4	4	4	4	4	4	5	5	3	4	4	4	4	3	4
	Professionalism	3	4	3	5	3	4	4	3	4	4	4	4	3	4	3	3	4	3	4	3	3	3	3	4	2	4	4	4	3	3	3	3	3
	Acceptability	3	3	3	4	4	4	4	4	4	4	3	4	4	3	4	5	4	5	4	3	3	4	4	3	4	4	4	4	4	3	4	4	4
